# Supplementary material for: Effects of Lifestyle Modification Interventions to Prevent and Manage Child and Adolescent Obesity: A Systematic Review and Meta-Analysis
Source: Nutrients. 2020 Jul 24;12(8):2208. doi: 10.3390/nu12082208 (PMC7468898; doi:10.3390/nu12082208)
Supplement: Supplementary file 1 [file nutrients-12-02208-s001.pdf]

Supplementary File

Table S1: Characteristics of included studies

Intervention group	Study	Country	Intervention	Comparison	Outcomes
**Diet, Exercise and Behavioral Therapy**	1. Alexander 2013 [1]	USA	Nutrition, exercise, motivational and educational messages	Standard of care	BMI change
	2. Arlinghaus 2019 [2]	USA	Sessions on diet, PA and behavior modification 1 day/ wk, 3 day/wk, 5 day/wk	Control	BMI z score
	3. Bartelink 2014 [3]	Netherlands	RealFit: 1:Exercise lessons; 2: Nutrition lessons; 3: Psychological lessons	No intervention	BMI z score, aerobic fitness dietary and PA behaviors, waist circumference, aerobic fitness, height, weight
	4. Bean 2015 [4-6]	Not specified	T.E.E.N.S—T.E.E.N.S. is a multidisciplinary treatment that includes physical activity, dietary intervention, and behavioral support. Parents attended bi-weekly groups (independent from adolescents). Participants met on alternating weeks with a dietitian and behavioral support specialist throughout T.E.E.N.S.' initial six months. Adolescents performed supervised physical activity ≥3x/week at the T.E.E.N.S. gymnasium, and were encouraged to exercise on additional days<br>MI Values— Adolescents in the MI treatment participated in two 30 minute, individual MI sessions at weeks 1 and 10 of T.E.E.N.S., led by interventionists independent from T.E.E.N.S.	Participants viewed 30 minute health education videos at identical time periods, proctored by interventionists, to match on attention without confounding content	Treatment adherence and attrition, BMI, height, weight
	5. Bocca 2012 [7-11]	Netherland	Children and parents in the multidisciplinary intervention program received dietary advice, physical activity sessions and, for parents only, psychologic counselling. Dietary advice consisted of 6 sessions of 30 minutes each, guided by a dietician. In addition, education and advice to improve eating behavior was given. The physical activity sessions consisted of 12	Children and parents in the usual-care group were followed up by a paediatrician, also during a period of 16 weeks. In this period, they were seen 3 times for 30 to 60 minutes each time. Information on healthy eating behavior was provided, and they were advised to perform physical activity for 1 hour	Physical activity, body composition (SCF and VF), BMI

			group sessions of 60 minutes each and were supervised by a physiotherapist.	per day, according to the Dutch Standard of Healthy Activities	
	6. Boutelle 2014 [12, 13]	Not specified	Regulation of Cues (ROC) program for four months	No intervention	Child and parents anthropometry, diet (Kcal per day), treatment acceptability, eating in the absence of hunger (EAH) scale, child eating behaviour questionnaire (CEBQ) scale, child usual dietary intake
	7. Braet 1997 [14]	Belgium	Intervention included cognitive strategies, behavioral strategies, educational and motivation components	Waiting list controls	Height, weight, percentage overweight
	8. Burrows 2012 [15-25]	Australia	Healthy Dads: Healthy Kids (HDHK) family lifestyle intervention. Five sessions were for fathers only, and 3 physical activity sessions involved fathers and children.	Waiting list controls	Body weight of fathers (kg and percentage), BMI, waist circumference, blood pressure and resting heart rate, physical activity, dietary intake, process evaluation
	9. Chen 2010 [26]	United States of America	Active Balance Childhood (ABC) study intervention: The intervention is based on the social cognitive theory.	Waiting list controls	Body mass index, waist-to-hip ratio, systolic blood pressure, diastolic blood pressure, caltrac count, fat, %, sugar, g, vegetables and fruit,number of servings, food choice, physical activity knowledge, nutrition knowledge, physical activity, self-efficacy, nutrition self-efficacy
	10. Chen 2011 [27, 28]	United States of America	Web based Active Balance Childhood (ABC) program consisted of activities to enhance self-efficacy of adolescents and facilitated their understandingand use of problem-solving skills related to nutrition, physical activity, and coping. Information related to nutrition	Participants in the control group also logged on to the Web site. Every week for 8 weeks, adolescents received general health information that was not tailored adapted from the American Academy of Pediatrics, the CDC, and the American Heart Association, related to nutrition, dental care, safety, common dermatology care, and risk-taking behaviors. Parents also received three internet sessions related to general information on	Body mass index, waist-to-hip ratio systolic blood pressure, diastolic blood pressure, caltrac count, fat, %, sugar, g, vegetables and fruit,number of servings, food choice, physical activity knowledge, nutrition knowledge, physical activity, self-efficacy, nutrition self-efficacy

				the topics taught in the control group.	
	11. Colorado LEAP study [29, 30]	Colorado, USA	classroom activities to try new foods and different physical activities	school's standard curriculum	Children's consumption of food, likings, anthropometry
	12. De Bar 2012 [31]	United States of America	The multicomponent intervention included the following:<br>1. change in dietary intake and eating patterns; increasing physical activity by using developmentally tailored forms of exercise (eg, exergaming);<br>2. addressing issues ssociated with obesity in adolescent girls (eg, depression, disordered eating patterns,poor body image); and<br>3. training participants' PCPs to support behavioral weight management goals collaboratively	Usual care	BMI z score, weight, BMI percentile, metabolic measures (mg/dL), total cholesterol, HDL, LDL, TGs, fasting glucose
	13. Dubuy 2014 [32]	Belgium	The key intervention strategy and the program consisted of three components: a start clinic, a school program, and an end clinic.<br>The intervention had two main topics which included healthy diet and PA	Usual care	Healthy diet and physical activity
	14. Dunker 2018 [33]	Brazil	1) group physical education sessions<br>2) Inter-active group educational sessions<br>3) motivational interviewing<br>Additionally, students were provided lunch on the days of the New Moves Program activities	Usual classes	Self-esteem, Body satisfaction, Physical activity, Healthy eating, Self-worth and feelings about the self, BMI, Unhealthy Weight-Control Behaviors (UWCB)
	15. Dzewaltowski 2010 [34]	United States	The HOP'N intervention model included three levels: a community/government/human service agency (County Cooperative Extension), after-school staff training, and after-school program quality elements. The quality elements included an organized daily PA session for at least 30 minutes, a daily	Not specified	Body Mass Index Z Scores (BMIz), Physical activity and Sedentary Behavior

			healthful snack that included a FV, and a weekly nutrition and PA education experience (HOP'N Club)		
	16. Elinder 2012 [35, 36]	Sweden	Stockholm County Implementation Programme in school (SCIP-school), a model project which allows schools to create their own solutions to healthy eating and physical activity	No intervention	Height, weight, BMI, BMI-SDS, health behaviours, validation of diet and physical activity questions, global self-esteem, well being and dieting
	17. Ellis 2010 [37-39]	The United States of America	Multi-systemic therapy (MST) (i.e., making changes in eating and activity level)	Shapedown, a family-based outpatient group weight management program, for 6 months	Family support for healthy eating and exercise, changes in adolescent diet, height, weight, percent body fat
	18. Family partners for Health Study [40-42]	North Carolina, USA	nutrition and exercise education and coping skills training	Wait list controls	BMI percentile, improvements in adiposity, nutrition and exercise health behaviors, and self-efficacy
	19. Figueroa- Colon 1996 [43]	The United States of America	Dietary-activity (high protein and very low calorie diet followed by a hypo-caloric diet) and behavioral weight loss program	No intervention	Blood pressure, weight, height, total cholesterol, triglycerides, lipoproteins
	20. Foster 2010 [44-50]	United States of America	HEALTHY Study (The intervention consisted of four integrated components denoted nutrition, physical education, behavior and communications)	Not specified	Prevalence of overweight and obesity (BMI ≥85th percentile), obesity (BMI ≥95th percentile), BMI z score, Continuous and categorical measurements of waist behavioral therapcircumference, Adverse events
	21. Fulkerson 2010 [51-56]	The United States of America	The HOME program: Each session included a healthy snack, separate parent and child group time, family meal preparation, interactive nutrition education activities, a group meal, homework assignment, take home materials, and session evaluations	Families randomized to the control condition participated in home assessments only and were sent written intervention materials at the end of the study	child and parent satisfaction, program dose, intervention fidelity, family dinner frequency and source of foods, parental self-efficacy, child food preparation skill, height, weight, home food availability, nutrition quality of foods served at family meals, dietary assessment
	22. LA health study [57, 58]	Louisiana, USA	Group 1 primary prevention,<br>Group 2 primary prevention plus secondary prevention,	No-intervention control	dietary measure, anthropometry

	23. Gallagher 2011 [59]	The United States of America	8-weekly psycho-educational groups over telemedicine	Meetings with primary care physician to discuss a standardized list of topics	Height, weight, BMI, daily intake of calories, percent fat from calories, fruit and vegetable servings, sweetened beverages, red foods, physical activity, parental report of child competencies and behavioral or emotional problems
	24. Gatti 2015 [60]	French Polynesia	Ressources Alimentaires et Santé aux Australes (RASA) intervention included two major components: a school nutrition program and a physical activity facility	No intervention	Fasting plasma glucose, Hyperinsulinemia, Waist circumference(WC), BMI
	25. Gee 2014 [61]	NA	Intervention group participants received individual sessions teaching and practicing problem-solving skills. Sessions included 5-minute videos introducing the steps of problem-solving, case examples, handouts, and worksheets.	Standard of care	Height, Weight, Confidence to Improve/Maintain Habits and Problem-Solving Skills
	26. Gerards 2015 [62, 63]	Netherland	Lifestyle Triple P intervention, a 14-week intervention comprising ten 90-minute parental group sessions and four individual 15–30 minute telephone sessions.	Two brochures (one on healthy nutrition and physical activity, and one on positive parenting), web-based tailored advice on setting a good example to their child, and suggestions for exercises to increase active play at home.	Children's BMI z score, waist circumferences, and biceps and triceps skinfolds, children's diet and physical activity level, parenting behaviors, process evaluation
	27. Germann 2006 [64]	The United States of America	FitMatters involved cognitive-behavioral therapy, nutritional counselling, exercise therapy, and medical management	Successful and Less Successful groups were delineated based on change in body mass index z-scores. Medical record review provided weight and height data one year before treatment for a subset of participants (comparison group)	Overall weight change, predictors of success, correlates of success rate of weight gain
	28. Goldfield 2001[65]	The United States of America	Mixed treatment whereby subjects received a mixture of individualized plus group treatment (mixed). The sessions for participants in the mixed treatment consisted of 15 – 20 min individual sessions with a therapist and 40 min of group therapy	Group sessions and with no individual attention	BMI, costs of recruitment and treatment, cost-effectiveness

	29. Golley 2007 [66, 67]	Australia	P Group: Parenting-skills training-parents participated in the Positive, Parenting Program (Triple P)<br><br>PDA Group: Parents in the PDA arm completed the Triple P program and the group participated in an additional 7 intensive lifestyle support group sessions	Wait list control	BMI z score, blood pressure, fasting glucose, total cholesterol, high-density lipoprotein cholesterol, and triacylglycerol levels,parent satisfaction with the intervention programs
	30. Graef 2014 [68-71]	United States of America	Extension Family Lifestyle Intervention Project for Kids (E-FLIP for Kids): Family-Based Behavioral Group Intervention (b) Parent-Only Behavioral Group Intervention	Education Control Condition	BMI z-scores, Height, Pediatric sleep duration
	31. Guo 2015 [72]	China	Psychological fitness included a 12-month education and consultation of diet, exercise and psychology	No intervention	Height, weight, BMI, waist circumference, hip circumference, waist-to-hip ratio and waist-to-height ratio, levels of fasting plasma glucose, cholesterol, triglycerides, low-density-lipoprotein cholesterol, and high-density-lipoprotein cholesterol
	32. Haines 2013 [73, 74]	The United States of America	The Healthy Habits, Happy Homes, a home based intervention that tailored individual counselling to encourage behavior change. Major components of the intervention included motivational coaching, mailed educational materials and incentives, and weekly text messages on adoption of household routines.	Received 4 monthly mailed packages that included educational materials on reaching developmental milestones during early childhood and low-cost incentives (e.g., colorings books).	Eating meals together as a family, child's sleep duration, child's TV viewing time, and presence of a TV in the room where the child slept
	33. Handel 2017 [75-79]	Denmark	Children were invited to see a health consultant on a regular basis over a 15 months period with a maximum of 10 visits, and most families received 4 to 5 consultations. The families in the intervention group were also invited to participate in optional monthly play events as a supplement to the consultations	Children met with a health consultan	Physical activity assessment, maternal BMI
	34. Hofsteenge 2013 [80-82]	Netherlands	Go4it is a multidisciplinary group treatment consisted of 7 sessions with two-week intervals, the adolescents received education	Regular care	Physical and psychosocial wellbeing, BMI-SDS

			on healthy dietary behaviour, screen behaviour and physical activity.		
	35. Hughes 2008 [83]	Scotland	The program consisted of 8 appointments (7 outpatient visits and 1 home visit) during 26 weeks with a total patient contact time of 5 hours. The program used a family-centered approach whereby the child (and family) took control of his or her own lifestyle changes	Typical dietetic care with 3 to 4 outpatient appointments with paediatric dietitians	BMI z Score, fat distribution, physical activity and sedentary behavior, quality of life
	36. Israel 1994 [84]	The United States of America	Children received comprehensive training in self-management skills. Training components included instructionin<br>self-goal setting,<br>formulating and implementing a plan to change behavior,<br>selfevaluation<br>self-reward,<br>training in problem-solving behaviors appropriate for high-risk or tempting situations.<br>Each component was presented to the children didactically and through therapist modeling, and the children were assigned homework exercises to practice the new skills. In addition, parents in this condition also rewarded their children for engaging in self-management skills	Standard treatment	Height, weight, triceps skinfold thickness, self-regulation and self-control, children's problem solving skills, parents' impressions of the percentage of responsibility their children assumed for completing specific weekly homework assignments
	37. Jelalian 2008 [85-89]	The United States of America	Aerobic: Activities for the exercise intervention included use of treadmills, stationary bicycles, and brisk walking within the hospital setting.<br>Peer enhanced adventure therapy: The peer-based activity session consisted of an initial 'warm-up' activity that included physical	Standard of Care	Weight, height, psychosocial variables

			activity, followed by the primary challenge for the group. 16 weekly sessions (one session of parents and children separate,one PEAT or EXER session), Biweekly dyad sessions; 4 monthly maintenance session.		
	38. Jensen 2015 [90]	Australia	Participants were given diet plans (reduced carbohydrate/ low fat) and physical activity related advice	Wait list control	Diet, physical activity,<br><br>glucose, insulin, leptin, adiponectin, total amylin, acylated ghrelin, active GLP-1, height, weight, BMI z-score, pubertal staging, body fat, appetite
	39. Johnson 1997 [91]	Not specified	Sessions on nutrition and eating-habit change followed by exercise (NE), exercise followed by nutrition and eating-habit change (EN)	Information control (INFO).	Weight, body-fat estimation, lipid profiles, adherence to the nutritional component, adherence to the exercise component, activity level
	40. Kalarchian 2009 [92]	The United States of America	Stoplight Eating Plan was provided and families were counselled about diet and exercise	Usual care	Percent overweight, changes in blood pressure, body composition, waist circumference, health-related quality of life
	41. Khan 2014 [93]	United States of America	After-school physical activity	Provided with written information	Dietary intake, Sexual Maturation, BMI, VO2max, Fat mass index, Body composition
	42. Knowlden 2015 [94-96]	The United States of America	The EMPOWER program was designed to reify and improve five social cognitive theory (SCT) constructs in mothers: environment, emotional coping, expectations, self-control, and self-efficacy.	Delivery of general health knowledge	Four child behaviours associated with prevention of obesity: physical activity, fruit and vegetable consumption,sugar-free beverage consumption, and screen time, Scores of five maternal-facilitated constructs of SCT: environment, emotional coping, expectations, self control,and self-efficacy for each behavior
	43. Lopera 2016 [97]	Brazil	Multidisciplinary intervention based on water-based exercises (WB)<br><br>Multidisciplinary intervention based on land-based exercises	Regular daily routine	Height, weight, waist and hip circumferences, body composition, health-related quality of life, physical fitness

	44. Maatoug 2015 [98, 99]	Tunisia	The 3-year school-based intervention program had 2 main components: educational lessons and environmental change. Content of classroom sessions consisted of interactive lessons. Teachers were asked to present at least 1 session to promote physical activity and 1 session to promote a healthy diet	No behavior modification program	Weight, nutritional intake, physical activity, sedentary behavior
	45. Macdonell 2012 [100]	Not specified	Four sessions focusing on motivational interviewing, diet and exercise	Nutritional counselling- For the control condition, a manual was developed for four sessions of nutritional counselling with caregivers and adolescents together, based on recommendations of the Expert Committee	Food consumption, physical activity, height, weight, BMI, reasons for healthy behaviour
	46. Magarey 2011 [101-103]	Australia	Healthy lifestyle education with parenting skills training intervention	Healthy lifestyle education without specific parenting skills training intervention	BMI z score, height, weight, waist circumference
	47. Mauriello 2010 [104]	The United States of America	Students self-directed through the 30-minute program in which they completed a series of TTM-based assessments and received stage-matched and tailored feedback messages based on their responses. Physical activity was the largest component of the intervention.<br><br>Multimedia sessions aimed at improving diet and physical activity	No treatment	Physical activity, fruit and vegetable consumption, limited tv viewing, physical activity (readiness), fruit and vegetable consumption (readiness), height, weight, BMI
	48. Moens 2012 [105]	Belgium	The training program involves six group meetings of 2 hours each over a 5-month period. Each family also received a comprehensive parent treatment work book. In the program the dietician educates the parents on the different food groups by providing detailed product information and child-friendly recipes	Wait list control	Height, weight, BMI,<br><br>children's eating behavior,<br><br>parental behavior, familial socio-economic situation

	49. Moore 2019 [106, 107]	The United States of America	HealthyCHANGE: The Healthy Change intervention consisted of behavior change strategies commonly used in cognitive behavioral and motivational interviewing interventions, such as problem-solving, goal setting, self-monitoring, and relapse-prevention skills.<br><br>SystemCHANGE: The System Change intervention was based on process improvement techniques and emphasized restructuring family daily routines (systems) to establish new healthy living habits.<br><br>The intervention mode of delivery consisted of small group sessions of 12 to 15 families who met in 25 face-to-face sessions in Year 1	Parent and child participants in this arm received 1 hour of private coaching from a registered dietitian on healthy eating and physical activity in Year 1 as well as a social telephone call and social event in all study years to enhance study retention	BMI, healthy weight behaviours (diet, physical activity, sedentary activity, sleep, and perceived stress) cardiometabolic risk factors
	50. Nemet 2014 [108]	Israel	All subjects and their parents were invited together for a series of four evening lectures along with exercise training and balanced hypocaloric diet	Children who did not participate in the structured program served as controls	Body weight, BMI, and fitness
	51. Nowicka 2008 [109]	Sweden	Therapy sessions for counselling on the need for parental cooperation, communication skills, mutual support, consistency, and establishment of appropriate limits	Wait list control	Weight, height, pubertal status
	52. Nyberg 2016 [110, 111]	Sweden	The intervention consisted of health information given to parents via a brochure that was sent home, motivational interviewing sessions provided to parents to promote healthy eating and physical activity amongst children, and classroom activities for children. Parents received 2 MI sessions lasting for 45 minutes. Children	Wait list control	Physical activity,<br><br>dietary indicators (fruit, vegetables and energy-dense products), physical activity habits, sedentary behavior, sleep, height, weight, waist circumference, BMI

			received 10 classroom sessions lasting for 30 minutes		
	53. Patsopoulou 2017 [112]	Greece	Diet & Activity,' and a Physical Activity Skill Development Program (Activity) and a combination of a Dietary Information and a Physical Activity Skill Development Program (Diet & Activity) . The activity intervention included a 45-minute, 3-day per week supervised training program, while the Diet & Activity intervention included a supplementary 15 minutes of group-based sessions attended by the parents	No intervention	Mean BMI, waist circumference, systolic and diastolic blood pressure, pulses per minute, and 50 m sprint run test at 3 months
	54. Po'e2013 [113]	The United States of America	Sessions, phone calls and event participation to promote a healthy diet and exercise. 12 weekly 90 minute sessions followed by 9 monthly 45 minute calls and then monthly events for 24 months.	Six 45 minute sessions	BMI, body fat percentage, Waist circumference
	55. Phaitrakoon 2014 [114]	Thailand	Multicomponent Obesity Control Program	Normal school curriculum	children's perception of obesity, the calories of dietary intake per day, the energy expenditure per day and the body fat %
	56. Reinehr 2003 [115-124]	Germany	Sessions focusing on nutrition, exercise and eating behaviour. Once weekly session on physical training lasting for 1.5 hours. First 3 months= group session: 1.5 hours each, evening sessions for parents: 1.5 hours each, physical training: 1.5 hours each. Next 3 months= nutritional counselling: 30 minutes, physical training: 1.5 hours each.	Wait list control	BMI-SDS BMI, skin fold thickness, waist circumference, bio-impedance analyses, blood pressure, physical activity, dietary records
	57. Resniscow 2005 [125, 126]	The United States of America	Once weekly group sessions and motivational interviewing phone calls focused on diet and exercise.	The moderate intensity group received 6 sessions of the same program	BMI, percentage body fat, waist, hip circumferences, blood pressure, serum measures of lipids, insulin, and glucose, cardiovascular fitness
	58. Robertson 2017 [127-130]	The United Kingdom	The FFH V2 manualised programme comprises 10 weekly 2½-hour sessions, with children and parents from 8 to 12 families	Usual care	Change in BMI z-score, Changes in children's physical activity, fruit and vegetable consumption and

			attending parallel groups. The programme combines information on parenting skills, social and emotional development as well as healthy eating including portion size and physical activity. The plan was to run six FFH courses (two in each site).		quality of life, parents' bmi and mental well-being, family eating/activity, parent-child relationships, parenting style
	59. Rosenbaum 2007 [131]	New York, USA	classroom sessions on diet and PA	No intervention	body fatness (bioelectrical impedance), insulin sensitivity, lipid profiles, and circulating concentrations of IL-6, C-reactive protein, adiponectin, and TNF
	60. Sabet Sarvestani 2009 [132]	Iran	The program involved behavior modification, dietary instruction and yoga therapy. The program covered standard behavioural strategies such as self-assessments and monitoring; additional measures taught were stimulus control, cognitive restructuring problem-solving skills, stress management (yoga), teaching healthy eating and physical behaviour and parent consultation and social support.	Attended 3 sessions of the intervention program	Body weight, BMI, Dutch eating behaviour questionnaire score, arm circumferences
	61. Savoye 2007 [133-136]	The United States of America	Counselling and exercise sessions to reduce weight	Diet and exercise counselling	Change in weight, BMI, body fat, homeostasis model assessment of insulin resistance (HOMA-IR), blood pressure, plasma glucose, insulin, total cholesterol, high-density lipoprotein cholesterol, low-density lipoprotein cholesterol, triglyceride levels
	62. Sharma 2019 [137]	United States of America	CATCH EC has three main components: (1) It's Fun to be Healthy! a nutrition and gardening-based curriculum; (2) developmentally appropriate structured, indoor and outdoor physical activities; and (3) parent tip-sheets including recipes, meal plans, parent-child activities, and recommendations for preschoolers' diet, physical activity, and screen time.	Not specified	BMI, Height, Weight, Process evaluation
	63. Shelton 2007 [138]	Australia	Components included an initial assessment session attended by the parent and child, followed by four group sessions at weekly	Wait list control	Height, weight, BMI, waist circumference, child's physical activity, sedentary electronic

			intervals of 2-hour duration each attended by the parent only. Each family received a comprehensive parent treatment manual developed by the project team		media time, food intake, parental behaviour, social validity
	64. Stark 2018 [139-144]	The United States of America	Family-based behavioral weight management intervention: Motivational interviewing (MI) was a parent only intervention consisting of 18 sessions over 6months. At the initial 60-minute session parents met with a paediatrician trained in motivational interviewing	Standard care	BMI z-score, child dietary intake, child physical activity, child sleep, home food environment, caregiver eating, caregiver activity, parenting and child eating behaviour
	65. Steinberg 2017 [145]	Israel	GRP1— participated in a 6-month multidisciplinary childhood obesity treatment program<br><br>GRP2— participated in a locomotion-emphasis 6-month multidisciplinary childhood obesity treatment program that included a similar program as GRP1, with additional specific exercises for improving their movement characteristics	No intervention	Movement Characteristics, Temporal Parameters, Foot Pressure
	66. Story 2003 [146, 147]	United States of America	After-School Program: focused on increasing physical activity and healthy eating. A family component was also included	GEMS Club ‘‘active placebo,’’ non-nutrition/physical activity condition	BMI, Diet, Physical activity, psycho-social measures
	67. Todd 2008 [148]	The United States of America	Sessions/Meetings, newsletters, TV allowances, ENUFF software, follow up phone calls and a seminar. Both groups attended meeting 1,2, 4 and 5 but only the intervention group attended meeting 3. Intervention group also attended a seminar.	No intervention	Height, weight, step counts, electronic media time and foods consumed while using electronic media, body fat composition, moderate and vigorous activity, food frequency data, electronic media access
	68. Vandongen 1995 [149]	Australia	1. Fitness: Included 6x 30 min sessions for activity programs and general exercise. They were planned to be conducted 15 min every school day throughout the year<br>2. fitness + school nutrition,<br>3. school-based nutrition: consisted of 10 1 hour lessons which aimed to improve attitude, knowledge and eating habits	No intervention	Nutrient intake,<br>Fitness,<br>Anthropometry,<br>Blood pressure,<br>Blood cholesterol

			4. school + home nutrition,<br>5. home-based nutrition: presented 5 nutrition based messages using comics delivered through school		
	69. Vos 2012 [150, 151]	Netherlands	First three months: 3 sessions<br><br>Next 3 months: biweekly sessions (7 for children, 5 for parents, and one together)	Advice on physical activity and nutrition	BMI-SDS, health-related quality of life
	70. Wafa 2011 [152, 153]	Malaysia	Sessions (8 sessions) for behavioral change to manage childhood obesity	Wait list control	Height, weight, habitual physical activity and sedentary behaviour, Health-related Quality of Life (QoL)
	71. Weber 2017 [154]	Germany	Physical and nutritional intervention	Usual care	Body height and weight, Body mass index (BMI), Physical fitness and motor abilities, Dietary behavior
	72. Wesnigk 2016 [155]	Belgium	Intervention program (10-month duration) included dietary restriction (1500–1800 kcal/day), supervised physical activity, and psychological support.	Usual care	Cholesterol efflux, HDL-Mediated eNOS Phosphorylation, Cell Culture
	73. West 2010 [156]	Australia	Group and telephone sessions for 12 weeks. Nine 90-min group sessions and three 20-min telephone sessions.	Wait list control	BMI z-score, weight-related problem behaviour, parenting self-efficacy, ineffective parenting
	74. Wilfey 2007 [157-160]	The United States of America	Group 1: behavioral skills maintenance (BSM) treatment<br><br>Group 2: social facilitation maintenance (SFM) treatment	No intervention	BMI z score and percentage overweight
	75. Williams 2010 [161]	United States of America	Team Positive Lifestyles for Active Youngsters	Standard care condition	Height, Weight, BMI, Attendance, Family functioning, Child Emotional/Behavioral Problems
	76. Williamson 2007 [162]	Not specified	The HEE program was designed with the goal of preventing inappropriate weight gain by modifying the school environment to improve healthy eating habits, increase physical activity, and decrease sedentary behavior at school and to encourage these same behavioral changes outside the school environment	Alcohol/Drug/Tobacco use/abuse prevention (ADT) program	BMI, weight gain prevention, percent body fat, digital photography of food selections and food intake, physical activity, self-esteem

	77. Wright 2013 [163]	The United States of America	Telephone counselling sessions	Wait list control	Initial efficacy, acceptability, and feasibility of the intervention
	78. Østbye 2012 [164, 165]	The United States of America	Mailed interactive kits, telephone coaching sessions using motivational interviewing and group sessions to promote obesity prevention. 20-30 minute calls every month and one group session	Received monthly newsletters emphasizing prereading skills	Change in child diet, physical activity, sedentary behavior, child weight change, change in parenting behaviors, mother's dietary intake and physical activity, mother and child weight
**Exercise and Behavioral Therapy**	79. Amaro 2006 [166]	Naples, Italy	KALEDO: Participated every week in one play session (15–30 min); Four "kaleidoscopes", by which the player can add energy for food intake or subtract energy expenditure for physical activity to the basal metabolic rate (BMR	No kaledo	BMI, BMIz, nutrition knowledge, physical activity and dietary intake
	80. Amini 2016 [167]	Iran	The intervention included nutrition education and increased physical activity (PA) for the pupils, lifestyle modification for parents, and a change in food items sold at the schools' canteens	No nutrition education	Waist circumference, physical activity, body mass index Z-score and hip circumference
	81. Anderson 2015 [168, 169]	Taranaki, New Zealand	home visits and weekly contact for PA sessions or psychology sessions	standard care of home visits but no sessions	Cost effectiveness
	82. Annesi 2013 [170, 171]	The United States of America	Physical activities, behavioral skills training. The Start For Life intervention incorporates primarily gross motor skills (e.g., running, jumping, hopping) with behavioral skills training interspersed.	Typical physical activity in preschools	Height, weight, BMI, physical activity intensity, physical activity
	83. Annesi 2015 [172]	USA	PA along with cognitive behavioural therapy for goal setting for diet and PA	Mandated PA	Self-efficacy, BMI, CV fitness
	84. Annesi 2017 [173]	USA	PA along with cognitive behavioural therapy for goal setting for diet and PA	Mandated PA	BMI, self-regulation for physical activity, overall negative mood, exercise self-efficacy (i.e. feelings of ability),11 cardiovascular endurance and strength
	85. Backlund 2011 [174, 175]	Umea, Sweden	received a 14-session intervention during 1 year	a single information session only before baseline measurement,	energy expenditure/day, (ii) time spent being physically active each

					day, (iii) steps/day and (iv) screen time, BMI
	86. Bianchini 2014 [176]	Brazil	Physical education +CBT	Control	Body composition, glucose, insulin, lipids and BP
	87. Butte 2017 [177]	USA	Diet and Exercise therapy (active participation and personnel directed learning with sport facilities)	Diet and Exercise therapy (self-directed learning via primary care clinical visits and printed material)	%BMI, body composition, blood pressure, and psychosocial status
	88. Bryant 2011 [178]	UK	Diet and exercise therapy (WATCH IT)	Waiting list	BMI, waist circumference and change in body fat %, 2 hour oral glucose tolerance test, lipid level, liver function test, blood pressure, fitness and physical activity over a 7-day period, parental BMI
	89. Chae 2010 [179]	Korea	Diet and exercise therapy	Conventional counselling	BMI, percent body fat, total body fat, plasma glucose, high-sensitivity C reactive protein (hs-CRP), insulin, adiponectin, total cholesterol (TC), high-density lipoprotein cholesterol (HDL), low-density lipoprotein cholesterol (LDL), and triglyceride levels (TG)
	90. Cliff 2011 [180]	Australia	Both PA and DIET	Child centered physical activity (PA), Parent Centered Diet program (DIET)	total physical activity, locomotor standard score, object- control standard score, gross motor quotient, perceived athletic competence, total screen time
	91. Cong 2012 [181]	USA	Exercise and diet therapy	No therapy	No outcomes reported
	92. COPE Healthy TEEN program [182-184]	Istanbul, Turkey	15 sessions which included educational information on leading a healthy lifestyle and cognitive-behavioral skill building.	Health topics other than physical activity	Weight, BMI, steps, knowledge of diet and physical activity, anxiety and depression
	93. Davis 2011 [185]	The United States of America	Circuit training (CT; aerobic + strength training) program, with and without motivational interviewing (MI) behavioral therapy	Wait list control	Baseline strength, physical activity/fitness, dietary, physical and metabolic characteristics

	94. De Heer 2011 [186]	USA	Health education and physical activity	Received health workbooks and incentives	BMI, Aerobic capacity, Dietary intentions and dietary knowledge
	95. del Río 2019 [187]	Spain	Educational program	Did not participate in intervention program	Knowledge of nutrition throughout the study, Adherence to the Mediterranean diet, Healthy habits in nutrition
	96. Dias 2018 [188, 189]	Australia	High-intensity interval training (HIIT)<br><br>Moderate-intensity continuous training (MICT)	Nutrition Advice	Left ventricular function peak systolic tissue velocity, cardiorespiratory fitness; abdominal adipose tissue; whole body composition; lipids, triglycerides, glycaemic control and insulin resistance, ferritin, haemoglobin, and leisure-time physical activity and nutrition.
	97. Eliakim 2007[190]	Israel	Nutritional education and physical intervention	Regular preschool schedule.	BMI, Physical activity
	98. Energy Balance for Kids with Play [191, 192]	USA	School-based multicomponent program- Energy Balance for Kids with Play (EB4K with Play)	Standard school curriculum	Dietary intake, moderate-to vigorous physical activity (MVPA) minutes, nutritional knowledge, BMI z scores
	99. Farah 2014 [193]	Brazil	High-intensity aerobic exercise training (HIT)	Low-intensity aerobic exercise training (LIT)	Pubertal maturation, Height, Weight, Peak oxygen uptake (VO2peak), Blood pressure, heart rate and heart rate variability Plasma insulin and leptin levels Blood glucose levels
	100. Farpour-Lambert 2019 [194]	Switzerland	Moderate-intensity individually delivered intervention (treatment A) or a high-intensity group (treatment B) plus physical activity	Standard care	Primary outcomes: Body weight and BMI, Secondary outcomes: Total body fat, Abdominal fat, Fat-free mass, blood pressure, Cardiorespiratory fitness, Physical activity level, carotid intima-media thickness
	101. Fetter 2018 [195]	United States of America	The 5 integrated Shaping Healthy Choices Program (SHCP) components are: (1) nutrition education and promotion; (2) family and community partnerships; (3) supporting regional agriculture; (4) foods available on the school campus; and (5) school wellness committees and policies	Not specified	Physical activity

	102. FitKid Program [196-199]	USA	FitKid after school program - The intervention consists of (a) academic enrichment, (b) a healthy snack, and (c) physical activity in a mastery-oriented environment	control	body composition, cardiovasctflar fitness, and heart period variability
	103. Freira 2018 [200]	Portugal	Motivational interviewing group (MIG)	Conventional intervention group (CIG)	HRQoL
	104. Freitas 2017[201]	Brazil	Multidisciplinary treatment	No intervention	Pubertal maturation, BMI, Stature, Triceps, subscapular, and medial calf skinfolds, Quality of life (QoL)
	105. Garcia 2019 [202]	Spain	multidisciplinary obesity treatment program (diet exercise and behavioral therapy)	No therapy	health-related quality of life (HRQoL), anthropometric, body composition, and cardiorespiratory fitness parameters
	106. Głabska 2019 [203]	Poland	Athletics for All (in Polish Lekkoatletyka Dla Kaz˙dego—LDK), a nationwide after-school physical activity program plus nutrition education	No intervention	Height, BMI, Body Weight, Waist-to-Height Ratio (WHtR), Waist circumference (WC), Body composition
	107. Gortmaker 2012 [204]	Pacific Northwest, Midwest, South, and Eastern US	program practice changes in the areas of physical activity and nutrition	Food and Fun After School was available at central childcare offices, but control sites were not notified they were available and did not receive training.	Physical activity
	108. Graf 2008 [205]	Cologne area in Germany	The school-based Children's Health Interventional Trial (the CHILT Project)	Normal school curriculum	overweight and obesity
	109. Greening 2011 [206]	Mississippi, USA	The intervention program included monthly family events that alter-nated between nutrition and physical activities/contests. Focus groups	standard curriculum	children's nutritional knowledge, number of different physical activities, fitness level, dietary habits, waist circumference, BMI percentile, and percentage body fat
	110. Grillich 2016 [207]	Lower Austria	create a healthy and positive learning environment and to improve the quality of physical education classes	Wait list control	motional and Social Experience in School, Physical Activity, Well-being, Motor Skills, and Attention Performance
	111. Gundogdu 2018 [208]	NA	Guidance about age-appropriate sport and exercise behaviors was also given to the	No intervention	Ealthy nutrition, exercise attitudes and behaviors

			adolescents. The adaptation behavior of the adolescents to the exercise type selected, such as basketball, volleyball, swimming, running, walking, or arm and leg exercises, was monitored.		
	112. Heino 2019 [209-211]	Finland	Let's Move It programme	Usual curriculum	Moderate to vigorous PA(MVPA) Sedentary behaviour (SB)
	113. Heo 2018 [212]	New York, USA	Health Corps	Control group	Weight, height, BMI
	114. Herget 2016 [213]	Leipzig, Germany	both groups received the same kind of high intensity interval training (HIIT) for 60 min twice a week that could be chosen from three available sessions per week (on weekdays) at the same time each afternoon (with approximately 8 to 15 participants each session) ALONG with text messages	both groups received the same kind of high intensity interval training (HIIT) for 60 min twice a week that could be chosen from three available sessions per week (on weekdays) at the same time each afternoon (with approximately 8 to 15 participants each session)	Anthropometry and metabolic outcomes
	115. Hoffman 2018 [214]	North Carolina, USA	Clinic – community partnership	Standard of care	BMI, clinic visits, waist circumference, health behaviors
	116. Hrafnkelsson 2014 [215]	Iceland	integrated nutrition and PA education	control	weight, fat percentage, cardiovascular risk factors, and blood pressure
	117. Ickovics 2019 [216]	New Haven, Connecticut, USA	Nutrition only, physical activity only, nutrition and physical activity (dual),	Received other health-relevant training (e.g., oral health, cold/ influenza prevention) during the study period, with obesity-related materials delivered after data collection was completed.	Anthro, nutrition and PA HABITS
	118. Kain 2009 [217]	Chile	Physical activity and nutritional program	Not specified	BMI Z-score (BMIZ) and obesity prevalence, waist circumference and triceps skinfold thickness.
	119. Kalantari 2017 [218]	Tehran, Iran	Educational session regarding healthy meals, high intensity physical activity along with parental involvement	No intervention	BMI and body fat
	120. Karczewski, 2016 [219]	NA	Urban Initiatives Work to Play health intervention	Wait list control	BMI
	121. Li 2019 [220, 221]	China	CHIRPY DRAGON	Usual care	BMI, Proportion of children with overweight or obesity; Waist

					circumference; Body fat percentage; Consumption of fruit and vegetables, unhealthy snacks and sugar-sweetened beverages; Time spent in moderate to vigorous physical activity (MVPA); Sedentary behaviors; Blood pressure; Cost-effectiveness analysis (CEA)
	122. Madsen 2013 [222]	Na	SCORES an after-school soccer program	Not specified	Change in after-school minutes of MVPA, body mass index (BMI)
	123. Magnusson 2012 [223]	Iceland	Physical exercises and dietary sessions	Received two compulsory 40-min sessions of physical exercise at the control schools	BMI, skinfolds, waist circumference, percentage lean mass, percentage fat mass and cardiorespiratory fitness
	124. Melnyk 2013 [224]	United States of America	The COPE program (educational and cognitive–behavioral skills-building program)	Healthy Teens program (attention control)	Healthy lifestyle behaviors, BMI, Mental health, Alcohol and drug use, Social skills, and Academic performance
	125. Meyer 2014 KISS trial [225-227]	Switzerland	Multi-component physical activity program of nine months including daily physical education (i.e. two additional lessons per week on top of three regular lessons), short physical activity breaks during academic lessons, and daily physical activity homework	No intervention	Body fat (sum of four skinfolds), aerobic fitness (shuttle run test), physical activity (accelerometry), and quality of life
	126. Nayak 2016 [228]	India	School based multicomponent intervention	Delayed dispatch of reading material	BMI, quality of life
	127. Nemet 2011 [229, 230]	Israel	Dietary- behavioral-physical intervention	No intervention	BMI percentiles, nutritional and physical knowledge and preferences, shuttle run test for fitness assessment.
	128. Nemet 2013 [231]	Israel	Dietary-behavioral-physical intervention	Ambulatory nutritional consultation	BMI, triceps and subscapular skinfold thickness, percentage body fat, physical activity, fitness assessment

	129. Odense overweight intervention study [232-235]	Denmark	Day camp of 6 weeks for nutrition and PA followed by 46 weeks of parent supported intervention	Once/week PA for 6 weeks	BMI, CVD risk factors and cognitive and motor skills
	130. Pablos 2017 [236]	Spain	Healthy Habits Program- HHP (exercise and diet therapy)	No therapy	lipid profile, blood pressure, BMI, cardiac output, blood glucose level
	131. Pbert 2016 [237]	USA	Nurse lead diet and exercise therapy	Normal school curriculum	BMI, BMI z score, waist circumference, body fat %, dietary intake, physical activity, sedentary behavior, total energy intake.
	132. Plasvic 2019 [238]	Serbia	High intensity interval training (HIIT) and nutritional advice-Diet and exercise therapy	Diet therapy	Biochemical and hormonal markers, cardiorespiratory fitness, BMI, BMI standard deviation scores (BMI-SDS), blood pressure, waist circumference.
	133. Reilly 2006 [239]	UK	Exercise therapy	Not intervention	BMI standard deviation score, physical activity levels and sedentary behavior
	134. Saraf 2014 [240]	North India	school component (policies), a classroom component (activities) and a family component [Information Education & Communication (IEC) material].	control	knowledge and behavioral changes in physical activity, diet and tobacco
	135. Sepulveda 2019 [241]	Spain	Psychosocial family based intervention	Without family intervention	Anthro, diet, physical activity
	136. Sgambato 2019 [242]	Rio de Janeiro, Brazil	The intervention aimed to improve diet quality, to enhance regular physical activity, and to reduce sedentary behaviors by combining primary prevention of obesity at schools and secondary prevention of obesity at home among adolescents with overweight or obesity	Routine activities for healthy behavior of the school.	body mass index (BMI) and percent body fat (%body fat)
	137. Shaw 2008 [243]	Connecticut, USA	Bright bodies	Clinic care	Height, weight, BMI, and body fat, glucose
	SHOT trial<br>138. Daley 2006 [244, 245]	UK	Exercise therapy: Participants attended 3 one-on-one sessions per week for 8 weeks and then completed a home program for another 6 weeks	Usual care	Self-perceptions (self-esteem), depression, affect, physical activity, aerobic fitness, and BMI

	139. Siegel 2014 [246]	USA	The program includes two 1-hour group exercise sessions per week, and families are invited to attend group nutrition education sessions once per month with a CBHN dietitian.	Not specified	BMI, and BMI Z-score
	140. Siegrist 2018 [247]	Germany	JuvenTUM 3 intervention	Usual activities	Body height, Weight, Fasting blood levels: Serum lipids and glucose, Adiponectin and leptin, concentrations, Retinal vascular diameters, Physical activity and physical fitness
	141. Silva 2018 [248]	Lisbon	Replacing traditional seating vs standing desks and motivation sessions	No intervention	Sedentary behavior and physical activity
	142. Simons 2015 [249, 250]	Netherlands	Received Play Station Move: received active video games and encouragement to play	Continued normal gaming behavior	BMI-SDSs, Waist circumference-SDSs, Hip circumferences, Skin fold thicknesses, Secondary outcomes, Self-reported sedentary screen time, Consumption of sugar-sweetened beverages and snacks, Physical activity behaviors
	143. Singh 2007 [251, 252]	Netherlands	11 lessons for the subjects of biology and physical education	regular curriculum.	Body height and weight, hip and waist circumference, 4 skinfold thickness measurements, and aerobic fitness
	144. Slusser 2013 [253]	Los Angeles, USA	Catch Kids Club Curriculum on nutrition and physical activity	Usual diet and PA curriculum	changes in student nutrition and physical activity knowledge, attitudes, and behaviors associated with the program
	145. Sousa 2018 [254-256]	Portugal	TeenPower mHealth Program	Waitlist control	Cognitive-behavioral indicators, User experience, adherence to the TeenPower intervention program Impact of program
	146. Spencer 2014 [257]	Nova Scotia, Canada	Heart Healthy Kids (H2K) program	In control schools, H2K included a physical activity challenge and education sessions	physical activity (measured by school day pedometer use) and aerobic fitness (maxi-mal oxygen uptake [VO2 max]

	147. Speroni 2007 [258]	USA	Kids Living Fit (KLF),	No intervention	body mass index (BMI) percentiles for age and gender and waist circumference
	148. Vajda 2007 [259]	NA	Of the study group (S) 21 volunteer obese children completed the exercise program (Participating in at least 90% of the sessions).	The members of group C had only 2 curricular PE classes a week.	Height, Body mass, Biceps, triceps, subscapular, suprailiac, and medial calf skinfolds (on both sides), relative body fat content, Peak oxygen uptake, Heart rate and minute ventilation, Anaerobic ventilator threshold
	149. van Leeuwen 2018 [260, 261]	Netherlands	Kids4Fit (multidisciplinary intervention program); includes exercise sessions and exercise and nutrition advice	Wait list control	Anthropometric measurements, BP measurements, Shuttle-run test (SRT), BMI-z scores
	150. Viggiano 2015 [262]	Campania, Italy	KALEDO : participated every week in one play session (15–30 min); Four “kaleidoscopes”, by which the player can add energy for food intake or subtract energy expenditure for physical activity to the basal metabolic rate (BMR	No kaledo	The primary outcomes were (i) score on the Adolescent Food Habits Checklist (AFHC) [20], (ii) scores on six sections of the dietary questionnaire and (iii) BMI z score.
	151. Viggiano 2018 [263]	Campania, Italy	KALEDO - Participated every week in one play session (15–30 min); Four “kaleidoscopes”, by which the player can add energy for food intake or subtract energy expenditure for physical activity to the basal metabolic rate (BMR	No kaledo	The primary outcomes were (i) score on the Adolescent Food Habits Checklist (AFHC) [20], (ii) scores on six sections of the dietary questionnaire and (iii) BMI z score.
	152. Wang 2018 [264, 265]	China	Routine health education was provided to all schools, whereas the intervention schools additionally received a 1-year tailored multi-component PA intervention program, including classroom curricula, school environment support, family involvement and fun programs/events.	Both control and intervention students received their routine health education programs regulated by educational authority	BMI z-score, BMI, Weight status (overweight and obesity), Physical activity
	153. WAVES study [266-271]	UK	Multifaceted intervention on diet and PA	Existing Educational resources	Height, weight, body fat, waist and arm circumference, dietary assessment, physical activity, blood pressure, economic evaluation
	154. Widhalm 2018 [272]	Austria	Lifestyle, nutrition and exercise lessons	No intervention	Weight, Height, Body Composition

					Nutritional Knowledge, Food preferences, Physical Fitness Psychological Measurements
	155. Xu 2017 [273]	Shanghai, Chongqing, Guangzhou, Jinan, Harbin and Beijing, China	Comprehensive nutrition and PA intervention	control	BMI, waist circumference, lipid profile
	156. Yusop 2018 [274]	Malaysia	Stage-based lifestyle modification	Standard treatment	BMI-for-age z-scores, Waist circumference (WC), Body fat percentage, Physical Activity, Dietary intake
**Exercise only**	157. Ackel Delia 2017 [275]	Brazil	aerobic + resistance training,	Aerobic training, leisure PA	Body weight, BMI, Insulin resistance, leptin levels
	158. ABC Study [276, 277]	Canada	ABC intervention, designed to increase PA and reduce sedentary behaviour.<br>ABC program delivered at a child care (CC) centre only<br>ABC program delivered at child care with a home/parental education component	Regular daycare curriculum	Height, weight, body composition, physical activity, sedentary time
	159. Aggeloussi 2012 [278]	Greece	Swimming >=1 year; >=3/wk and >=1hr/session	Inactive	BMI, PA and blood markers such as glucose, adiponectin, resistin
	160. Alberga 2013 [279]	Canada	High repetition, moderate-intensity resistance exercise training sessions twice a week	No intervention	Height, weight, body composition, muscular strength
	161. Almas 2013 [280]	The United States of America	Physical activities, behavioral skills training.	Typical physical activity in preschools	Height, weight, BMI, physical activity intensity, physical activity
	162. Andrade 2016 [281]	The United States of America	Educational classes for adolescents, workshops for parents, organization of social events, providing walking trails, standard school physical education curriculum, posters	Standard school curriculum	Diet (energy intake and food group consumption), physical fitness, sedentary time, physical activity, BMI, waist circumference
	163. Balagopal 2005 [282-286]	The United States of America	Weight management program (Shapedown). The subjects were advised to perform physical activity for 45 min three times per week for 3 months. Each session	Usual care	BMI, adiponectin concentration, % Body fat, insulin concentration, HOMA-IR, C- reactive protein (CRP), LDL/HDL, IL-6

			included warm-up/flexibility exercises and aerobic activity consisting of progressive stretching techniques for 5 min.		
	164. Barbosa Filho 2017 [287-289]	Brazil	The intervention program was structured into four main components:<br><br>training and activities in the general curriculum;<br><br>training and activities in PE classes;<br><br>active opportunities in the school environment;<br><br>health education in the school community	No intervention	Height, weight, PA determinant
	165. Barkin 2011 [290]	The United States of America	Six sessions over the course of 6 months; the initial session at the community-based primary care clinic and the subsequent five sessions at the YWCA recreational centre. At the initial clinic visit, each family received behavior modification counselling in brief principles of motivational interviewing. During this visit, both parent and child completed a goal setting contract and participated in a 45-minute group health education session, led by a trained bilingual study member, on increasing daily physical activity	Consisted of 2 sessions (baseline and 6 months later), all located at the community-based primary care clinic. Families received standard of care counselling These sessions were enhanced by a subsequent 45-minute group health education session	Adiposity, BMI
	166. Bastian 2015 [291]	Canada	Alberta Project Promoting active Living and healthy Eating in Schools; health facilitator	Standard IE material	Physical activity, BMI
	167. Benjamin 2015 [292]	North Carolina, USA	The Mebane on the Move volunteers promoted PA through (1) walking and running clubs in the elementary schools for children and in the community for families, (2) portable play equipment provided to low-income families through home delivery food assistance programs, and (3) sidewalks, crosswalks, and walking trails installed throughout town	Neighboring community 8.4 miles away was compared	child PA and BMI

	168. Berge 2016 [293]	Minnesota, USA	create and implement a family focused childhood obesity prevention intervention using the Citizen Health Care model of CBPR	Control	weight and weight-related behaviors (e.g., physical activity, sedentary behavior, dietary intake).
	169. Bibiloni MM 2019 [294]	Spain	Nutritional intervention and physical activity	Nutritional intervention alone	Prevalence of obesity and overweight, abdominal obesity
	170. Bohlin 2017 [295]	Sweden	Group activities along with individualised sessions. The children participated in an educational and physical activity group. After this initial group treatment programme, treatment was individualized for each patient and involved visits to a medical doctor (normally 1–2 times/year), a nurse (1–8 times/year) and, if necessary, a dietician and physiotherapist.	Usual care	Change in BMI SDS, degree of obesity, average time spent by the treating nurse for each patient, parental weight status,<br>family living situation
	171. Borjesson 2019 [296]	Sweden	Providing mothers with a pedometer	Only Children provided with pedometers	Steps per day
	172. Camhi 2011 [297, 298]	The United States of America	8-month life skills-oriented physical exercise class	Standard physical exercise	Physical fitness, physical activity measure
	173. Carlin 2018 [299]	UK	Peer-led walking in school intervention – WISH	Standard school physical education	sedentary behavior, light physical activity, moderate physical activity, vigorous physical activity, total physical activity, social support, self- efficacy, perceived benefits to physical activity.
	174. Chang 2008 [300]	China	Exercise intervention	No intervention	BMI, Triglyceride level, fasting insulin, exercise performance
	175. Chen 2016 [301]	Taiwan	Physical activity program	Wait list control	Physical fitness, obesity status, executive function, and HRV
	176. Chen 2017 [302]	Taiwan	Exercise therapy	Reading course	Cardiovascular fitness, muscular endurance, power, flexibility, obesity and metacognition
	177. Chritison 2016 [303]	USA	Exercise and behavioral therapy	Didactic curriculum	BMI, BMI z scores, cardiovascular, behavioral and psychosocial measurement

	178. Coimbra 2017 [304]	Portugal	Exercise therapy	No therapy	BMI z scores, body fat mass, CRP, IL-6, ferretin, hepcidin and sTfR, iron concentration, waist circumference, waist-to-hip ratio
	179. Cvetkovic 2018 [305]	Serbia	Behavioral therapy	Usual PE exercises	BMI, body fat %, cardiorespiratory fitness, muscular fitness, body composition
	180. Damaso 2014 [306]	Brazil	Exercise and behavioral therapy	Exercise and behavioral therapy	No relevant outcomes reported
	181. Davis 2006 [307]	USA	Low-dose aerobic exercise (20 minutes per day), High-dose aerobic exercise (40 minutes per day)	No-exercise control condition	Pediatric Sleep Questionnaire scores
	182. Davis 2011 [308]	NA	Low dose (20 minutes/day) or high dose (40 minutes/day) aerobic exercise	No exercise	Standardized psychological evaluations (Cognitive Assessment System and Woodcock-Johnson Tests of Achievement III) assessed cognition and academic achievement. Functional magnetic resonance imaging measured brain activity during executive function tasks
	183. Davis 2012 [309]	USA	Low dose training, high dose training-exercise therapy	control	BMI, body fat
	184. Deforche 2005 [310]	Belgium	Monthly checkups, behavioral therapy sessions and phone calls by therapists along with calorie restriction and physical activity	No intervention	Height, weight, physical activity, frequency of watching television and playing computer games
	185. De Greeff 2016 [311]	Netherland	Physical activities integrated into regular classroom learning : program focusing on integrating physical activity into the routine academic lessons such as mathematics, spelling, and reading	No intervention	Height, weight, physical fitness
	186. Delgado-Floody 2018 [312]	Chile	high-intensity interval training (HIIT)	low-intensity interval training	BMI, Waist circumference (WC), Body fat (BF), Waist-to-height ratio (WHR), Cardiorespiratory capacity

					Heart rate (HR), systolic blood pressure (SBP), and diastolic blood pressure (DBP)
	187. Dennis 2013 [313]	NA	Two different doses of exercise	No exercise	BMI, waist circumference, percent body fat, insulin resistance and b-cell function and several lipid markers
	188. Donnelly 2009 [314, 315]	The United States of America	Extra 90 minutes per week of physical activity	Regular classroom instructions	Height, weight, daily PA, academic achievement, implementation and fidelity of paac, direct observations of classroom PA
	189. Eichner 2016 [316]	The United States of America	Run or walk 1 mile each day and then involve in activities like soccer, basketball, football, volleyball	No intervention	Height, weight, BMI
	190. Eiholzer 2010 [317]	NA	Twice weekly in guided resistance training for 4 months	Unchanged training schedules	Spontaneous physical activity energy expenditure (SpAEE; 3-axial accelerometry for 7 days), muscle strength, and body composition (dual energy x-ray absorptiometry
	191. Epstein 2008 [318]	Not specified	The group received ideas for alternatives to sedentary behavior, a tailored monthly newsletter with parenting tips to reduce sedentary behavior,and information about how to rearrange the home environment to reduce access to sedentary behavior	Children were provided free access to television and computers and received $2.00 per week for participating, independent of their behavior change. Control families received a newsletter to provide parenting tips, sample praise statements, and child-appropriate activities and recipes	Age- and sex-standardized BMI (zBMI), television viewing, energy intake,physical activity
	192. Erfle 2015 [319]	The United States of America	Physical exercise program for 30 minutes every school day	Normal schedule of non daily physical exercise	Height, weight, physical fitness
	193. Escobar-chaves 2010 [320]	The United States of America	Workshop and newsletters to reduce sedentary behaviour	No intervention	Media usage
	194. Faith 2001 [321]	The United States of America	TV viewing contingent on pedaling a stationary cycle ergometer	Inactive group	Weight, height, percent total body fat, pedaling and TV viewing times

	195. Farias 2015 [322]	Brazil	Physical activity programme with aerobics, sports games and stretching twice per week for 60 minutes	Conventional physical education classes	Heart rate, maturation stage, age of menarche, weight, height, subscapular and triceps skinfold, sum of skinfolds, waist circumference
	196. Farmer 2017 [323]	New Zealand	Intervention schools were given funds to help with altering school play spaces	No change in play spaces	Height, weight, physical activity, play space evaluation
	197. Fiorilli 2017 [324]	NA	Moderate-intensity Resistance Training (RT) and a high-intensity RT	Aerobic Training (AT)	Height, weight, BMI, waist, circumference and waist–hip ratio, Heart rate, % fat mass
	198. Goldfield 2006 [325-327]	Canada	Open-loop feedback plus reinforcement- Children were provided objective feedback on their physical activity by wearing a physical activity monitor. The physical activity that they accumulated was rewarded with access to television.	Open-loop feedback- Children were required to wear activity monitors and, therefore, were provided feedback on physical activity but had free access to television independent of physical activity. Families in this group were not given any activity goals to avoid confounding goals with the effects of the feedback from activity monitors	Physical activity and television viewing, body composition
	199. Graves 2010 [328]	North West England	jOG encourages step-powered video gaming of light-to-moderate intensity	continue playing their video games as normal	anthropometric variables, body fat and steps
	200. Gutin 1999 [329]	Georgia, USA	Controlled physical training	control	body composition, PA and diet
	201. Hagstromer 2009 [330]	Sweden	The exercise sessions consisted of a variety of group activities and lasted over 13 weeks	Wait list control	BMI, aerobic capacity (VO2max and VO2max/kg/min), physical activity
	202. Ham 2016 [331]	South Korea	The experimental group was offered a TTM-based exercise counseling consisting of eight sessions during a 3-month period	For students in the control group, were provided counselling booklets and one session of counselling for each student and a weekly 60-minute session of music skipping rope exercise was provided to students in both groups for 3 months	Stages of change in exercise, pros and cons of exercise, self-efficacy, BMI

	203. Hayes 2015 [332]	West Virginia USA	P group = intervention led by principal, in entire school<br><br>CT = intervention led by class teachers	No instruction to modify student daily activity.	heart rate and laps completed
	204. Herrick 2012 [333]	San Francisco, USA	Sports, Play, and Recreation for Youth (SPARK) program	Wait list control	moderate-to-vigorous physical activity (MVPA), BMI z-score, and cardiorespiratory fitness (VO2)
	205. Hollis 2016 [334-338]	Denmark	Camp Intervention Arm. The intervention for the DCIA comprised two parts—an intensive 6-week day camp intervention and a subsequent 46-week family-based intervention program (52 weeks in total). In the camp, children achieved about 90 min of moderate to vigorous physical activity per day measured by accelerometry	The standard intervention consisted of one weekly fun-based physical activity session (2-h duration) for 6 weeks.	Children's executive function, visuospatial construction and nonverbal memory, height, weight, BMI, body fat, parental education level and ethnicity, pubertal development
	206. Hull 2018 [339]	Tennessee, USA.	active intervention (weight gain prevention)	Oral health	BMIZ, BMI, child's waist circumference, physical activity, screen time, dietary behaviours and preferences for fruits and vegetables
	207. Ildiko 2007 [340]	Budapest, Hungary	weekly, two physical education classes of 45min. plus three extra-curricular activity sessions of 60min. duration)	wo curricular physical education classes every week	height, body weight, biceps, triceps, subscapular, suprailiac and medial calf skinfold
	208. Ingul 2018 [341]	Brisbane, Australia	High intensity interval training (HIIT moderate intensity continuous training (MICT)	Nutrition only	Cardiac and vascular function
	209. Jago 2019 [342]	South West England.	The Action 3:30 after-school clubs were scheduled to run twice per week for 15 weeks in each school and last 60 min per session.	CONTROL	MVPA, BMI, health related QOL
	210. Jones 2015 [343]	Wollongong, Australia	The PA programs were delivered biweekly (Tuesday/ Thursday for boys and Monday/Wednesday for girls)	HL programs were delivered weekly (Monday for both boys and girls	Implementation, acceptability, percentage body fat and BMI z-score
	211. Klakk 2013 [344]	Denmark	School based PE program consisting of PE lessons for 4.5 hours per week	Regular PE curriculum which includes lessons for 1.5 hours per week	BMI, total body fat percentage

	212. Krafft 2014 (a) [345]	Georgia	Aerobic exercise	Sedentary attention control	Resting state fMRI, Children's cognitive processes, Salience network, Default mode network, Motor network
	213. Krafft 2014 (b) [346]	Georgia	Aerobic exercise	Sedentary attention control	Resting state fMRI, Antisaccade tasks, Flanker tasks, Task performance, Correlations between change in health variables (percent body fat or VO2 peak) and change in brain activation
	214. Krombholz 2012 [347]	Germany	Children had more opportunities for unstructured and structured physical activities. Children received at least one weekly 45-minute- session of physical education and sessions of physical activities (mainly organized games, at least 20 min. per day) on the other days	Regular curriculum which included one session of physical activities per week of 45 min duration	Motor skills, physical fitness, manual dexterity,<br><br>height, BMI, weight, skinfold-thickness, information about the children’s family circumstances
	215. Lazzer 2009 [348]	Italy	High-intensity (HI) equicaloric exercises	Low intensity (LI) equicaloric exercises	Body mass, BMI, Fat-free mass (FFM), Basal metabolic rate (BMR), Peak oxygen uptake and maximal fat oxidation, Maximal oxygen uptake (VO2max), Substrate oxidation rate, Protein oxidation rate, Energy supply (kJ/min)
	216. Lee 2010 [349]	Korea	Aerobic exercise training	No exercise sessions or dietary restriction	Plasma total cholesterol, HDL cholesterol, Triglycerides, Plasma glucose, Dietary-intake, Total energy expenditure
	217. Liu 2007 [350]	China	Happy 10 program is a classroom-based physical activity program	No intervention	Metabolic rate (MET), Body mass index (BMI), Prevalence of overweight and obesity, Daily energy expenditure, Physical activity
	218. Liu 2008 [351]	The United States of America	Happy 10 programme is a classroom-based physical activity programme for primary-school students. Many safe and age- and space-appropriate physical activities are included in the programme materials. Classroom based physical activity program	No intervention	Height, weight, BMI, energy expenditure, prevalence of overweight/obesity,<br><br>duration and intensity of physical activity

			at least once every school day for ten minutes		
	219. Liu 2017 [350]	Taiwan (China)	Coordination exercise program	Wait list control	Cognitive function and inhibitory control, Physical fitness, BMI, Cognitive function measures (i.e., Tower of London task)
	220. Maddison 2012 [352-354]	New Zealand	Participants received an upgrade (hardware and games) of existing gaming technology enabling them to play Sony PlayStation EyeToy™ active video games at home. Children were encouraged to meet current physical activity recommendations (60 minutes of moderate to vigorous physical activity on most days of the week)	Normal video game play	Change in BMI, BMI, including aerobic fitness (VO2Max), time spent in moderate-to-vigorous physical activity (MVPA), and food snacking at 24 weeks
	221. Manley 2014 [355]	The United States of America	Pedometer intervention along with a daily health class	Wait list control	Height, weight, BMI, BMI percentile, relative body mass index (RBMI), physical activity assessment, aerobic fitness assessment, self-efficacy assessment
	222. Martinez Vizcaino 2008 [356, 357]	Spain	The program consisted of three 90-min sessions per week for 24 weeks. The physical activity sessions sessions included sports with alternative equipment (pogo sticks, frisbees, jumping balls, parachutes, and so on), cooperative games, dance and recreational athletics.	The standard physical education curriculum	BMI, triceps skin-fold thickness, and percent body fat, blood lipid level and blood pressure
	223. Martínez-Vizcaíno 2014 [358]	Spain	MOVI KIDS (physical exercise sessions)	Standard physical education curriculum	Waist circumference (WC), Triceps skinfold thickness (TST), Body fat %, Fat-free, Mean arterial pressure (MAP), Triglycerides, HDL-cholesterol and LDL-cholesterol, insulin and C-reactive protein mass, Energy expenditure, Daily physical exercise
	224. Maud 2019 [359, 360]	France	High Intensity Interval Training (HIIT)	Moderate Intensity Continuous Training (MICT)	Maturation status, Eating behavior traits, Individual aerobic capacity, Anthropometric measurements,

					Appetite sensations, Daily energy intake, Body composition, Maximal oxygen uptake test (VO2)
	225. Moller 2014 [361]	Denmark	Sport schools with physical exercise	Normal schools	Habitual physical activity, organized leisure time sports participation and bicycling, Fitness, BMI, Waist circumference
	226. Monteiro 2015 [362]	Brazil	Aerobic training was performed three times per week, which consisted of walking and running<br>The concurrent training was performed at three times per week, with each session consisted of 60 min of 50 % of resistance training time followed by 50 % of the aerobic training	The adolescents were instructed not to change their usual eating or physical activity behavior during the entire 20 weeks	Body mass, height, waist circumference, body composition and fat distribution, intra-abdominal adiposity tissue (IAAT), subcutaneous adiposity tissue (SAT), and hepatic steatosis, total cholesterol (TC), triacylglycerol (TG), high density lipoprotein (HDL), low density lipoprotein (LDL), very low density lipoprotein (VLDL), glucose, non-ester fatty acid (NEFA), cytokines (IL-6, IL-10 and TNF-α), PAI-1, maximum stress test, prediction test of a maximum repetition
	227. MOPO study [363-365]	Oulu, Finland	Interactive gamification methods on PA in young men	Control	Body composition, WC, PA
	228. Morgan 2012 [366]	Australia	PALs was a multi-component school-based intervention that included enhanced school sport sessions with a focus on resistance training, physical activity and nutrition handbooks with home-based challenges, interactive seminars addressing key lifestyle physical activity and nutrition behaviours, leadership principles and self-directed lunch-time exercise sessions.	No intervention	BMI, BMI z-score and % body fat
	229. Muller 2019 [367]	South Africa	Physical activity lessons (PA), health and hygiene (HE) and nutritional education (NU)	Normal school based physical education	Height, weight and skinfolds; the latter including triceps and subscapular and cardiorespiratory fitness, socioeconomic status (SES), self-reported physical activity, haemoglobin (Hb) and infection

					with soil-transmitted helminths and intestinal protozoa
	230. Nascimento 2016 [368]	Portugal	Exercise therapy	No therapy	BMI, BMI z scores, total fat percentage, lipid profile, glucose metabolism, adipokines and inflammatory markers
	231. Neumark Sztainer 2003 [369-371]	The United States of America	High-school physical education class 4 times per week as well nutrition and social support sessions	Written materials on healthy nutrition and exercise	Weight, height, diet, physical activity, sedentary activity, self-acceptance, self-worth, athletic competence, physical appearance, media internalization, socio-environmental factors
	232. Nobre 2017 [372]	Brazil	Exercise twice a week - The progressive plyometric training program consisted of a set of exercise on nonconsecutive days twice per week for 12 weeks under monitored and controlled conditions.	No intervention	Weight, height, tricep and subcapsular skin fold measurement, body fat, health-related physical fitness, gross motor coordination
	233. Nogueira 2014 [373]	Australia	High-intensity exercise regimen	Usual physical education	years to age peak height velocity, weight, standing height, sitting height, waist circumference, resting heart rate, blood pressure, vertical jump, estimated maximal oxygen consumption, stiffness index, vertical jump, bone mineral content at different sites, fat mass, lean mass, tibial bone metrics, radial bone metrics
	234. Nowicka 2009 [374]	Sweden	One-week sports camp and six-month support system	Wait list control	Weight, height, body composition, measurement of lifestyle
	235. Olvera 2010 [375, 376]	The United States of America	Exercise sessions (three weekly), nutrition sessions (two weekly) and a behavioral counselling session (one weekly)	Exercise session (once a week)	Height, weight, aerobic fitness (daughters), duration and intensity of physical activity (daughters), aerobic fitness (mothers), peak oxygen consumption, average activity level (mothers)

	236. Oreskovic 2016 [377, 378]	The United States of America	Counselling on working towards physical activity goals (2 meetings)	Handout with standard-of-care diet and physical activity recommendations	Change in mean daily minutes of MVPA, change in percent of adolescents achieving the recommended 60 min of daily MVPA
	237. Pate 2005 [379]	USA	Physical activity	Regular PE classes	percentage of moderate-vigorous activity per day, prevalence of overweight, prevalence of obesity
	238. Prado 2015 [380]	Brazil	Exercise therapy- high intensity<br><br>+diet and behavioral therapy	Exercise therapy- low intensity<br><br>+diet and behavioral therapy	BMI, body mass, fat mass, tricep skin fold thickness, subscapular skin fold thickness, biochemical markers, total energy intake
	239. Racil 2016 [381]	Tunisia	Plyometric exercise + high intensity interval training (P+HIIT)	HIIT and no exercise	BMI, body fat%, lean body mass, BMI z-scores, waist circumference, blood glucose level, plasma insulin, plasma leptin, adiponectin, energy intake
	240. Riiser 2014 [382]	Norway	12-weeks access to an online program providing tailored physical activity counselling based on principles from Self-determination Theory and Motivational Interviewing	Standard follow-up by the school nurses	Cardiorespiratory fitness (shuttle run test), HRQoL, leisure time exercise, body image and self determined motivation for physical activity and exercise, Age- and gender-adjusted body mass index (BMI), height and weight
	241. Robbins 2006 [383, 384]	The United States of America	Computer program and counseling targeted at increasing physical activity	Age specific PA recommendations	Height, weight, PA frequency, intensity, duration, and readiness; information regarding the HPM constructs of interpersonal influences, activity-related affect (PA enjoyment), self efficacy, perceived benefits and barriers of PA
	242. Robinson 1999 [385, 386]	The United States of America	The intervention, which was based in Bandura's social cognitive theory, consisted of incorporating 18 lessons of 30 to 50 minutes into the standard curriculum. These lessons were followed by a television	Only assessments	Height, weight, triceps skinfold thickness, waist and hip circumferences, cardiorespiratory fitness, self reported media use, physical activity, dietary intake,

			turnoff, during which children were challenged to watch no television or videotapesand play no video games for 10 days. After the turnoff, children were encouraged to follow a 7-hour per week budget.		parental report of child and family behaviors, sedentary behavior
	243. Robinson 2010 [387-389]	The United States of America	The GEMS Jewels After School Dance Intervention was offered five days per week, 12 months per year (excluding school holidays), at community centers in selected neighborhoods.<br><br>Sisters Taking Action to Reduce Television (START) is a home-based screen time reduction intervention designed to incorporate African or African-American history and culture,including up to 24 lessons over two years	It consisted of state-of-the-art, culturally-tailored, authoritative, information-based health education on nutrition, physical activity, and reducing cardiovascular and cancer risk. It included 24 monthly newsletters for the girls and their parents/guardians, and quarterly community center health lectures	Height, weight, BMI, waist circumference, triceps skinfold thickness (R arm), resting blood pressure and resting heart rate, fasting serum insulin, glucose, and lipids, physical activity, television viewing, videotape viewing, dietary intake, eating meals with the television,<br><br>self-reported psychosocial measures, depression inventory (CDI), sexual maturation
	244. Rolland- Cachera 2004 [390]	France	Diet therapy<br><br>PROT-diet	Diet therapy<br><br>PROT+diet	BMI, BMI, z scores, waist circumference, hip circumference, waist/hip ratio, skin fold thickness (triceps and subscapular).
	245. Roth 2015 [391, 392]	Germany	Prevention through Activity in Kindergarten Trial (PAKT)	Routine schedule	Change in percentage of (wearing), time spent in moderate to- vigorous physical activity, Change in a composite motor skills score, Changes in percentage of time spent in MVPA and the composite motor skills score, BMI, Blood pressure, Sum of four skinfolds (triceps, biceps, subscapular, and suprailiac), Frequency of accidents and infections
	246. Sacchetti 2013 [393]	Italy	Exercise sessions: 60 min/daily of moderate to rigorous physical activity. The physical activity program that was enhanced in terms of duration, intensity, and frequency	Standard program of physical education involving 2 lessons of around 50 minutes a week in the gym	BMI, overweight/obesity, physical activity

	247. Salmon 2008 [394-396]	Australia	Two intervention components: 1) a behavioural modification (BM) condition and 2) Fundamental movement skills (FMS) condition	Usual curriculum	Weight (kg), height (cm), BMI; physical activity, screen behaviours, enjoyment of physical activity, fundamental movement skills, unintended consequences (like adverse body image-related consequences from the interventions), food intake: food-frequency
	248. Schaeffer 2014 [397]	Georgia USA	Aerobic exercise.	sedentary attention control	BMI, body fat, V02, MRI changes
	249. Scheffler 2007 [398]	Berlin, Germany	Regular exercise program	No intervention	Anthropometry and body fat
	250. Schranz 2014 [399]	Australia	Components included an initial assessment session attended by the parent and child, followed by four group sessions at weekly intervals of 2-hour duration each attended by the parent only. Each family received a comprehensive parent treatment manual developed by the project team	Wait list control	Height, weight, BMI, waist circumference, child's physical activity, sedentary electronic media time, food intake, parental behaviour, social validity
	251. Schwanke 2016 [400]	Brazil	30 minute sessions 3/week on stretching and strengthening	No intervention	Body posture, abdominal strength
	252. Seabra 2016 [401]	Portugal	S = SOCCER PROGRAM<br>TA = Traditional PA program	Control	body composition, cardiometabolic risk factors, inflammatory and oxidative markers, cardiorespiratory fitness and perceived psychological status
	253. Seo 2019 [402]	Korea	Exercise intervention	Usual care	Anthro, body composition, BP, dietary intake, PA
	254. Shaibi 2006 [403]	Los Angeles, USA	Resistance training	Control group	anthro, body fat, VO2
	255. Siegrist 2011 [404, 405]	Germany	JuvenTUM intervention	Usual activities	BMI, Moderate to vigorous physical activity index, Physical fitness
	256. Sigal 2014 [406]	Ottawa (Ontario) and Gatineau (Quebec), Canada,	aerobic training (n = 75), resistance training (n = 78), combined aerobic and resistance training (n = 75); all received dietary counseling	Non-exercising control (n = 76); all received dietary counseling	body fat %, Weight, height, BMI, waist and hip circumferences and blood pressure

	257. Sigmund 2012 [407]	Czech republic	School based PA intervention	Traditional PA programs	Over weight/ obese prevalence and physical activity
	258. Silva 2015 [408]	Pernambuco (northeast of Brazil)	high-intensity (HIT) aerobic training	low-intensity (LIT) aerobic training	Fatness, fitness, lipid profile, glucose
	259. Simon 2004 [409-411]	France	Sessions on physical activity and sedentary behaviours. 3 sessions per week	Regular school curriculum	Changes in overweight prevalence, body composition, PA and SB, and attitudes toward PA
	260. Smith 2016 [412-417]	Australia	Teacher professional learning (2 × 5 h workshops); provision of fitness equipment to schools (1 × pack/school valued at ~ $1500); researcher-led seminars for students (3 × 20 min); face-to-face physical activity sessions (20 × ~90 min, in addition to regular PE lessons); lunch-time physical activity leadership sessions (6 × 20 min); pedometers for physical activity self-monitoring (17 weeks); parental strategies for reducing recreational screen-time (4 × newsletters); and a purpose-built web-based smartphone application (15 weeks)	Not specified	Weight, height, physical activity, sedentary behavior, sugar-sweetened beverage consumption, muscular fitness, resistance training skill, motivational regulations for school sport
	261. Son 2017 [418, 419]	Korea	Combined Exercise Training	No intervention	Heart rate, Blood glucose concentrations, Serum insulin Systolic BP (SBP) and diastolic BP (DBP), BMI, Endothelin-1, Body fat (%) and lean body mass (%)
	262. Spruijt Metz 2008 [420]	The United States of America	Education about decrease in sedentary behavior and increase in physical activity	Not specified	Physical activity, meanings of physical activity, motivation for physical activity, height, weight, body fat
	263. Staiano 2013 [421-425]	The United States of America	Active video games every school day	Usual activities	Height, weight, self-reports of self-efficacy, self-esteem, and peer support
	264. Tkacz 2008 [426]	NA	Low dose exercise condition (20 min/d)<br><br>High dose exercise condition (40 min/d)	No-exercise control condition	Body weight, Height, BMI z-score, Anger Expression, Aerobic Fitness, Percent Body Fat
	265. Thivel 2011 [427]	France	Sessions for physical education (once a week) and physical activity (twice a week)	Regular physical education	Body mass, Height, Waist, Skinfold thickness, Aerobic

					performance, Anerobic performance
	266. Tsang 2009 [428]	NA	Kung Fu sessions	Tai Chi sessions (placebo training)	Metabolic outcomes, Fat and lean mass, Waist circumference, Height, Body mass, Muscle strength, Dietary intake, Habitual physical activity
	267. Van Stralen 2012 [429, 430]	Netherlands	The JUMP-in intervention, targeted sport participation, and outdoor play.. The JUMP-in is a school-based multicomponent intervention, including<br><br>Pupil followup system, a yearly monitoring instruments of PA, BMI and motor skills;<br><br>School sport activities<br><br>Calendars offering recurrent breaks for PA, relaxation and posture exercises during regular lessons;<br><br>Personal workbooks for children and their parents with assignments to perform in class and at home and an instruction book for the school staff;<br><br>Parental information services including information meetings, courses and sport activities for parents; and<br><br>Extra care for children at risk, wherein children detected by the pupil follow-up system receive additional adapted physical education lessons or motor remedia lteaching.	Usual curriculum	Sports participation, unorganized outdoor play,<br><br>screen behaviors, personal and environmental mediators
	268. Vasconcellos 2015 [431]	Brazil	Soccer intervention: The RSP was performed three times a week during 12 weeks	Non-exercise control groups	Body mass, Height, BMI, Blood pressure, Hip circumference, and waist circumference (WC), Fat percentage and fat-free mass, Biochemical markers, Cardiorespiratory fitness, Endothelial function, Heart rate

					variability (HRV), Inflammatory biomarkers
	269. Velez 2010 [432]	The United States of America	Resistance training sessions for 3 days a week	Regular program	Total body strength, body composition (BF%, lean body mass (LBM), fat mass (FM), and BMI), self-concept, participants' perception of the intensity of the workout
	270. Villa Gonzalez 2017 [433]	Mexico	Activities of Parents in the Intervention Group:<br><br>Website. An interactive workshop to learn and use the project website was developed.<br><br>Messages by mobile phone. A short message was sent to the parents' mobile phone, every week. The message was related to the current topic of the website that motivated and reinforced behavior changes. A total of 40 messages were sent<br><br>In-person activities. Parents were invited to 3 sessions of 1 hour each, once every 2 months, for the purpose of giving them feedback on topics of eating and physical activity that were on the website, to participate in some project activities, and to answer their questions and receive comments to modify or improve the website.<br><br>Activities of Children in the Intervention Group<br><br>Workshops. The workshops were integrated with both board games and physical games and with educational materials to reinforce healthy eating habits and physical activity.<br><br>Educational materials. They were provided with board games and plastic place mats with pictures of a healthy dish, the pyramid	No intervention	Weight, height,<br><br>waist circumference, BMI, BMIZ

			of physical activity, and a picture of a healthy drink pitcher.<br><br>Visit to Universum Museum.<br><br>Posters. Each month, in a visible area and an area with large influx of children inside the school a new poster alluding to the project and the current website topic was placed.		
	271. Walther 2009 [434]	Germany	According to German standards, 2 units (each 45 minutes) of physical education per week are mandatory in all schools. Intervention classes were assigned to 1 unit of physical exercise (45 minutes) with at least 15 minutes of endurance training per school day	The control classes continued to receive 2 units of exercise per week. In addition, lessons on healthy lifestyle were included in the regular schedule once monthly for all pupils	Body composition, blood pressure, heart ratebody coordination test,<br><br>treadmill exercise test with spirometry, laboratory analyses such as lipid profile and the amount and the migratory function of CPCs
	272. Weintraub 2008 [435]	United States of America	After-school soccer program	The “active placebo” control intervention consisted of an after-school health education program.	Implementation, acceptability, body mass index, physical activity measured using accelerometers, reported television and other screen time, self-esteem, depressive symptoms, and weight concerns
	273. Whooten 2018 [436]	United States of America	Students participated in BOKS for 12 weeks. 2 days/week program and 3 days/week program. BOKS sessions lasted ≅60 minutes and started with a warm-up game, transitioned into running, relay races, or obstacle courses, and included a skill of the week (e.g., plank, running, jumping).	Usual care	BMI<br>BMI z-score<br>Social–emotional wellness
	274. Wong 2008 [437]	Singapore	Aerobic Exercise Training Programme (n=12) + typical 2 sessions of 40-minute physical education (PE) per week in schools	typical 2 sessions of 40-minute physical education (PE) per week in schools	BMI, resting heart rate, systolic BP, triglycerides, serum CRP concentration
	275. Wright 2019 [438, 439]	United States of America	100 Mile Club walking/running program	CHALK/Just Move classroom activity break PA program	Receptivity and sustainability, Program reach, Dose received, Implementation and fidelity, Dose delivered
	276. Yildrim 2014 [440]	Australia	PA-I: 18 lessons on the use of pedometers; children were provided with sports equipments. Additionally, 9 family	Usual practice	Sedentary time and physical activity, anthropometry: height,

			newsletters were sent home that included project updates and tips to increase children's PA at home, and teachers assigned active homework tasks<br>SB-1: Teachers delivered a 30 min standing lesson daily; during 2 hr teaching blocks teachers asked the children to stand after 30 min for a 2 min light intensity activity break<br>By mid-intervention 9 family newsletters were sent home, and weekly homework tasks to decrease sitting time at home were set by teachers<br>PA+SB-I: This group received a blended version of the PA-I and SB-I		weight and waist circumference, blood pressure, serum bio-markers
	277. Zehsaz 2016 [441, 442]	NA	Exercise (Ex) group participated in additional 2 sessions per week of 45 to 60 minutes per session of exercise training	Both the ExG and the CG participated in the typical 2 sessions of 40-minute physical education (PE) per session per week	Height, Weight, BMI, Serum chemerin concentration, Whole-body fat percentage (%), Fat mass, Lean body mass, Fasting glucose and insulin concentrations
**Behavioral Therapy Only**	278. Abraham 2015 [443]	Hong Kong	Simplified Lifestyle Modification Programme Group (simplified LMP) vs Internet Group (IT)	received usual care visits with a physician	Physical activity level, dietary intake, stress level, and knowledge related to nutrition/physical activity, as well as weight and blood pressure.
	279. Ahmad 2018 [444]	Malaysia	Social media and Facebook sessions	Control	BMI z score, weight circumference and % body fat
	280. Alkon 2014 [445]	USA	Child care center policy revisions but parent involvement as well through workshops and flyers	Standard of care	BMI
	281. Arlinghaus 2017 [446]	USA	Physical education with compañeros	Physical education without compañeros	zBMI
	282. Angelopoulos 2009 [447]	Greece	Diet and PA Sessions	Control	Anthro, diet, BP, PA
	283. Armstrong 2017 Text-MI study [448]	NORTH CAROLINA, USA	usual care plus daily (Monday– Friday) text messages on their designated mobile device for 12 weeks; GOAL SETTING, DIET AND pa	Monthly lifestyle counseling visits by a physician and dietician. Standard care participants received	BMIZ, child health behaviors and cardiovascular fitness,

				text message reminders for the 3-month study outcomes visit.	
	284. BacardÃ--Gascon 2012 [449]	Mexico	Nutrition and physical activity professionals interacted with parents, children, and teachers. Its purpose was to show how food choices and physical activity depend on personal behavior, individual health and school and family environment.	Delayed intervention	BMI s z-core
	285. Bagherniya 2017 [450, 451]	Shahinshahr,, Iran	evidence-based psychological (i.e., self-efficacy, outcome expectation) behavioral (i.e., knowledge and skills), and environmental (i.e., parents, teachers support, and situation) components was conducted over 7 months (30 weeks) using the SCT.	we held six lectures for students and their parents in the control group and gave them two nutritional books.	Wt, ht , waist circumference, psychological variables
	286. Ball 2011 [452]	Canada	Youth Lifestyle program; healthy initiatives program	Waitlist control	BMI s z-core, height, weight, BMI, waist circumference, glucose, lipids
	287. Baranowski 2003 [453]	United States of America	Interactive multimedia Girls health Enrichment Multisite Studies (GEMS) Fun, Food, and Fitness Project (FFFP) activities	Usual camp activities	Body mass index (BMI), consumption of fruit, 100% fruit juice, and vegetables (FJV), physical activity
	288. Barkin 2018 [454]	USA	Growing Right Onto Wellness Healthier	Growing Right Onto Wellness Smarter	BMI, obesity, diet and PA variables
	289. Berkowitz 2013 [455]	USA	Intensive lifestyle modification sessions (23)	Brief self-guided sessions (6)	Weight, height, BMI, BMI z-score, waist ciorcumference
	290. Black 2010 [456]	USA	Behavioral therapy	No intervention	Body fat percentage, BMI, z-BMI, fat mass and fat-free mass, average daily physical activity counts per minute and minutes per day in play-equivalent physical activity
	291. Bolton 2017 [457, 458]	Australia	Health Promoting Communities: Being Active<br>Eating Well (HPC:BAEW) initiative	No intervention	Differences in anthropometry (weight, waist, body mass index (BMI) and standardized BMI (BMI-z score), differences in health-related behaviours, quality of life, and school environment assessments

	292. Boodai SA 2014 [459]	Kuwait	Behavioral therapy	No treatment	BMI z scores, waist circumference and blood pressure
	293. Boutelle 2013 [460]	USA	Guided self-help treatment for pediatric obesity (Behavior therapy)	Delayed Behavioral therapy	BMI, BMI z scores and percentage overweight
	294. Broccoli 2015 [461, 462]	Italy	Motivational Interviewing (MI)- behavioral therapy	Usual care by family pediatrician	BMI, BMI z scores, change in BMI z scores, subgroup analysis using gender and mother's education, physical activity habits and dietary habits
	295. Byrd-Banner 2017 [463-465]	USA	Behavioral- HomeStyle	No treatment	Meal related behaviors, household improvements
	296. Casazza 2007 [466]	USA	Behavioral therapy	No therapy	BMI, 24 hour recall, knowledge retained, physical activity, dietary habits, meals skipped per day, food frequency questionnaire
	297. Cason 2006 [467]	USA	Behavioral therapy	No therapy	A knowledge and behavior questionnaire covering nutrition and physical activity
	298. Chawla 2017 [468]	Bangkok, Thailand	Diet and Exercise therapy	No therapy	Overall knowledge, overall attitude, diet practice, Physical activity practice, overall practice, physical activity, screen time, sleep, , prevalence of obesity/overweight, lipid profile
	299. Chen 2019 [469]	USA	Behavioral therapy	General health education	BMI, sedentary activity,, pediatric quality of life adolescent scores, Physical activity self-efficacy and healthy eating self-efficacy
	300. CHOOSE HEALTH program [470-474]	Australia	Cognitive Behavioral therapy- CHOOSE HEALTH	No treatment	Tanner stage, height, weight, BMI, number of overweight/obese, waist circumference, hip circumference, fat mass, abdominal fat, lean tissue, bone mineral density, total energy intake, physical activity (average weekly steps)

	301. CHOPPS (Christchurch obesity prevention programme in schools) [475, 476]	Southwest England	Focused on discouraging children from consuming carbonated drinks and involved one hour of additional health education during each of the four school term	No intervention	height, weight, BMI, waist circumference
	Cloutier 2018 [477, 478] .302	USA	an enhanced program (NFN+) that incorporated behavioral change strategies	(Nurturing Families Network, NFN	Breastfeeding extent , duration, suitability, infant sleep pattern, maternal diet and behavior, maternal activity, infant screen time, tummy time, BMI, infants' nocturnal awakenings, weight – for-length
	303. Cox 2017 [479]	Australia	Diet and behavioral therapy	On waiting list for intervention	No outcomes of interest reported
	304. Crespo 2018 [480]	USA	Behavioral therapy	No therapy	No outcomes of interest reported
	305. CRETE study [481-483]	Crete	'Health and Nutrition Education programme	No intervention	Diet, anthropometry, skin folds, body fat, blood pressure, lipid profile
	306. Croker 2012 [484]	UK	'Family-based behavioural treatment' (FBBT) for childhood obesity	Wait list control	Body mass index (BMI), BMI SD. scores (SDSs),weight, weight SDSs, height, height SDSs, waist, waist SDSs, FM index, FFM index, blood pressure (BP) and psychosocial measures
	307. Da Silva 2019 [485]	Brazil	Diet and behavioral therapy	No therapy	Food consumption over last 7 days, BMI, waist circumference, hip circumference, arm circumference, tricep skin fold thickness, subscapular skinfold thickness. Physical activity level
	308. Davis 2012 [486]	NA	4-month nutrition and strength training intervention (group class maintenance)	Newsletter maintenance	Height, weight, blood pressure, body composition via BodPod™, lipids and glucose/insulin indices
	309. Davis 2013 [487]	USA	Behavioral therapy	Baseline primary physician visits	BMI, BMI z scores, BMI percentile, 24-hour dietary recall, physical activity, children behaviour
	310. Davoli 2013 [488]	Italy	Motivational Interviewing	Usual Care	Primary outcomes: Individual BMI score variation (ΔBMI), Secondary outcomes:

					Percentage of positive changes in parent reported dietary behaviors and in PA
	311. Day, 2008 [489]	Not specified	Action Schools! BC – Healthy Eating intervention	HE usual practice	Fruit and Vegetable Consumption, Fruit and Vegetable Knowledge, Attitudes and Perceptions Willingness to Try New Fruits and Vegetables, Fidelity to the Whole-School Model, Fidelity to Classroom Dose, Fidelity to Model
	312. De Lepeleere 2017 [490, 491]	Belgium	Online parenting videos	Waitlist control	Demographic variables, parent reported child's PA, screen-time and healthy diet, Specific parenting practices and parental self-efficacy concerning these practices
	313. De Moraes 2017 [492]	Brazil	Healthy lifestyle educational program	Session on general health issues	Change in fasting glucose, Total cholesterol, Dietary intake, BMI
	314. Dennison 2004 [493]	United Stated of America	Program designed to reduce television viewing	Received curriculum, materials, and ideas for activities about health and safety were provided to day care or preschool staff and information and materials or at-home activities were mailed to parents	Number of hours per week of television/videos viewed and the number of hours per week of computer/video games played Changes in children's growth
	315. de Villiers 2016 [494]	South Africa	HealthKick(HK), a healthy lifestyle intervention	Not specified	Nutrition knowledge, Nutrition behavior score, Self-efficacy score
	316. de Visser 2016 [495]	United States of America	Project Healthy Schools (PHS) intervention plus environmental activities	Non- Activity Group: Project Healthy Schools (PHS) intervention without activities	Frequency of physical activity and sedentary behavior, Dietary intake Outcomes related to environmental activities
	317. de Vries 2015 [496]	Netherlands	Parents received recommendations on activity	Standard of care	Anthropometry, Skinfold thicknesses, Bioelectrical impedance analyses, Motor development, Daily physical activity
	318. Doring 2016 [497-501]	Sweden	Over ~39 months, families<br>participated in 1 group session and 8 individual sessions in motivational interviewing, focusing on healthy food habits and physical activity	Usual care	BMI, overweight prevalence, waist circumference, children's and mothers' food and physical activity habits, and mothers' BMI, prevalence of overweight, and waist circumference

	319. Dunn 2012 [502]	United States of America	Move-To-Improve (MTI), a classroom-based physical education program with trained teachers	Move-To-Improve (MTI), a classroom-based physical education program without trained teachers	Physical activity
	320. Early 2019 [503]	United States of America	5-2-1-0 Let's Go! Program: Motivational Interviewing and home visits	Usual care	BMI, Health Behaviors
	321. Eldridge 2016 [504]	United States of America	Received face-to-face intervention during 10 ninety-minute face-to-face meetings, attended by parents only, over an 8-month period	The minimal intervention Control group consisted of parents and children who received printed materials mailed to their homes approximately every 3 weeks over the same 8 months	Attitudes and behaviors related to all aspects of the interventions including physical activity, food and nutrition, body image, appearance, body esteem, self-esteem, and quality of life
	322. Epstein 1995 [505]	NA	Reinforcing decreased sedentary activity and increased physical activity (combined)	Reinforcing decreased sedentary activity (sedentary)Reinforcing increased physical activity (exercise)	Physical work capacity, Height, Weight, Preference, compliance, and choice
	323. Epstein 2004 [506]	NA	Reinforced reduced sedentary behavior:	Stimulus control of sedentary behaviors	BMI, Physical activity, Dietary intake
	324. Epstein 2008 [507]	NA	Diet and Exercise intervention: Treatment meetings included group and individual sessions for parents and children totaling approximately one and a half hours	Usual meeting families were weighed, were seen as a family by their case manager, and then went to separate parent and child group meetings	BMI, Dietary Measures: Servings per day of fruits and vegetables, high energy-dense (RED) foods and low-fat dairy, Active behaviors, Parental feeding practices
	325. Epstein 2014 [508]	United States of America	Family-based behavioral treatment (FBT)	Parent and child (PC) treatment	BMI, Payer cost, Parent and child costs
	326. FABO Study [509-511]	Norway	CBT for families	Waiting list	body mass index standard deviation scor, self-esteem, symptoms of depression and blood parameters indicative of cardiovascular risk
	327. Fidancı 2017 [512]	Turkey	Health Promotion Model (HPM) -based individual and group education	Received routine follow-up only	Individual characteristics and experiences, Behavioral outcomes (commitment to a plan of action, immediate competing demands and preferences, and health promoting behavior), Healthy eating habits, Average daily time spent in front of the television or computer, Self-confidence scores, BMI

	328. Fisher 2019 [513]	NA	Food, Fun, and Families (FFF) attended weekly group intervention sessions for twelve weeks	Delayed intervention	SoFAS (kcal), Child daily energy, Height, Weight, BMI, Authoritative food parenting
	329. Flodmark 1993 [514]	NA	Family therapy, Conventional treatment	No treatment	BMI, Skin fold thickness
	330. Foster 2008 [515]	United States of America	School Nutrition Policy Initiative	Not specified	Incidence of Overweight and Obesity, Prevalence of Overweight and Obesity, Remission of Overweight and Obesity, Dietary Intake and Physical Activity, Potential Adverse Effects, Sedentary Behavior
	331. French 2018 [516]	United States of America	NET-Works Intervention Program	Usual Care	BMI, Dietary intake, Physical activity, Screen time
	332. FRESH (Family, Responsibility, Education, Support, & Health) study [517-519]	USA	Behavioral therapy- parent based therapy (PBT)	Family based therapy (FBT)	BMI, BMI z score, parent BMI, child and parent energy intake and physical activity, parenting style and feeding behavior.
	333. Gholami 2014 [520]	NA	Received theory-based instructional leaflets to promote self-regulatory skills for providing a healthy nutrition for children	No intervention	Vegetable intake, Planning, Self-efficacy
	334. Gomez 2018 [521, 522]	Spain	Thao-Child Health Program (TCHP) a community based program	Followed usual health care policy	Waist circumference, BMI, Waist-to- height ratio (WHtR), Physical activity and adherence to the Mediterranean diet
	335. Gong 2014 [523]	China	PA and nutrition education, parents sessions, food service personnel	No intervention	Anthropometric, dietary intake, and physical activity data
	336. Gourlan 2013 [524]	France	Standard weight loss program + motivational interviewing	Standard weight loss program + motivational interviewing	PA and BMI
	337. Graf 2006 [525]	Cologne, Germany	school- and family-based intervention consisting of extra lessons, healthy nutrition and physical education for overweight and obese children	control	Height, weight, blood pressure, waist circumference
	338. Greve 2015 [526]	Denmark	HSN program	No intervention	Waist circumference, BMI, Obesity, Total body fat, Percentage body fat, Fat-free mass

	339. Guelph Family Health Study [527-529]	Ontario, Canada	Group 1 = four home visits (HV) with a health educator, emails, and mailed incentives<br><br>Group 2 = two HV, emails, and mailed incentives	general health advice through emails	fat mass, MVPA, change in food groups, sedentary time
	340. Haire-Joshu 2010 [530]	United States of America	PARADE mentors delivered 8 lesson plans, 8 child-focused computer-tailored storybooks, and 8 parent action support newsletters addressing positive diet and activity behavior patterns.	Usual care	Primary outcomes: child nutrition and physical activity knowledge, daily caloric intake, percent of calories consumed from fat, daily servings of fruits and vegetables, percent of time spent in physical activity, BMI z-score, and parental daily caloric intake, percent of calories consumed from fat, daily servings of fruits and vegetables, and minutes walked per week. Secondary outcomes: change in child reported attempt to challenge self to eat 5 fruits or vegetables a day or to be active for at least one hour each day.
	341. Hakanen 2006 Special Turku Coronary Risk Factor Intervention Project for Children (STRIP) [531]	Turku, Finland	The families of the intervention group received individualized dietary and lifestyle counselling at 1–3-month intervals until the child was 2 years old and twice a year thereafter	The families of the control group were seen by the counselling team twice a year until the child's age was 7 years and once a year after that age, but they only received similar basic health education as routinely given at Finnish well-baby clinics and school health care	Prevalence of overweight and obesity
	342. Hakanen 2009 Special Turku Coronary Risk Factor Intervention Project for Children (STRIP) [532]	Turku, Finland	The families of the intervention group received individualised dietary and lifestyle counselling at 1–3-month intervals until the child was 2 years old and twice a year thereafter	The families of the control group were seen by the counselling team twice a year until the child's age was 7 years and once a year after that age, but they only received similar basic health education as routinely given at Finnish well-baby clinics and school health care	Prevalence of overweight and obesity

	343. Harvey Barino 2003 [533]	St. Regis Mohawk community of Akwesasne, USA	The peer educator was instructed to focus exclusively on how improved parenting skills could facilitate the development of appropriate eating and exercise behaviors in children	Parenting support only	weight and height (WHZ score and weight-for-height percentile for children), dietary intake (3-day food records), physical activity (measured by accelerometers), parental feeding style (Child Feeding Questionnaire), and maternal outcome expectations, self-efficacy, and intention to change diet and exercise behaviors
	344. Healthy Beginnings Trial [534-536]	Sydney, Australia	1 hour visit by nurse to monitor feeding and physical practices	Usual care	Anthropometry, time sent watching TV, time spent in active play
	345. Healthy Eating and Lifestyle Programme (HELP) [537-542]	UK	The Healthy Eating and Lifestyle Programme-HELP	Standard care	BMI, BMI z scores, fat mass and fat mass percentage, health related quality of life , eating attitudes Test, Rosenberg Self- Esteem Scale, Psychological health using Development and well-being Assessment (DAWABA) online interview, Physical activity, cardio-metabolic risk factors, health economic outcomes.
	346. Hidayanty 2016 [543]	Makassar city, Indonesia	12 weekly 75-min nutrition education group sessions, which focused on behavioral modification; furthermore, their parents received weekly nutrition education leaflets.	Adolescents from the control schools, but not their parents, received leaflets on evidenced-based nutrition information.	BMI z-scores, waist circumference, snacking habits, sedentary activity, and the adolescents' self-efficacy data
	347. Horodynski 2005 [544]	Michigan USA	nutrition lessons and structured reinforcement	control	meal time behaviors, parents self-efficacy
	348. Hu 2017 [545]	Guangzhou, China	training of staff, initiation of a health curriculum, collaboration with families	Routine health care provision	BMIz
	349. Huang 2007 [546]	San Diego, USA	The Patient-Centered Assessment and Counseling for Exercise Plus Nutrition Project (PACE +)	Standard care	Body image and self esteem
	350. Hystad 2013 [547]	Trondheim, Norway	therapist-led groups (TLG)	self-help groups (SHG) for parents	Body fat, BMIz, dietary intake

	351. Infant Feeding Activity and Nutrition Trial (InFANT) Extend Program [548, 549]	Australia	Infant Feeding Activity and Nutrition Trial (InFANT) Extend Program	General child care	Anthropometry, dietary intake, physical activity
	352. INSIGHT Trial [550-554]	USA	Responsive parenting	Child safety message	screen time, television exposure, and interactive play
	353. Jansen 2011 [555, 556]	Netherlands	Sessions for parents focusing on how to manage obesity in children. 2 hour sessions for 8 weeks	Wait list control	Weight, height, eating psychopathology, eating behavior, physical activity, self-esteem, negative thought, knowledge test, motivation
	354. Jancey 2014 [557]	Perth, Australia	PA and nutrition program; face to face workshops at playgroups	No contact	intake of various food groups
	355. Jansen 2017 [558, 559]	USA	POPS+ Incredible Year Series (IYS)- behavioral therapy<br><br>Pre-school obesity prevention series (POPS)- school based curriculum. (behavioral therapy)	usual Head Start program (HS)	Externalizing behavior, diet measures, BMI, race/ethnicity, education of parent//guardian, income-to-needs ratio, screen time and physical activity.
	356. Johansson 2015 [560, 561]	Sweden	Early STOPP (STockholm Obesity Prevention Program): The intervention consists of two components, one educational and one individually targeted coaching	Usual care	Physical activity, Sedentary behavior, Height, Weight, BMI, Motor skills, Family-related factors
	357. Jones 2008 [562]	Idaho and California USA	StudentBodies2-BED	wait-list control group	BMI, binge eating, mood changes
	358. Kaufman-Shriqui 2016 [563]	Israel	Nutrition education and physical activity lesson	Physical activity lessons only	Nutritional habits, Packed Lunch Score, Physical Activity and Sedentary Behaviors, Children's nutritional knowledge, BMI
	359. Kalavainen [564-567]	Eastern Finland	Family-based group treatment (15 separate sessions for parents and children). These sessions included nutrition education, physical activity education and behavioral therapy	routine counseling (two appointments for children)	Anthropometry
	360. Kebaili 2014 [568]	Tunisia	Received special courses and actions that promoted healthy nutrition.	No intervention	Students' knowledge, behaviors, and intentions about healthy nutrition and dietary habits

	361. KEYS trial [569, 570]	North Carolina, USA	The Keys intervention included three modules addressing Family child care homes (FCCH) provider health, the FCCH environment, and FCCH business practices	business-focused intervention	Diet quality, PA, height, weight, and waist circumference, BMI percentile and z-score
	362. Kharofa 2015 [571]	United States of America	Wellness Action Plan	Diet and activity plans were also recorded within the patient note in the electronic medical record	BMI
	363. Klesges 2010 [572-576]	United States of America	Memphis GEMS (Girls health Enrichment Multi-site Studies): Group behavioral counseling to promote healthy eating and increased physical activity (obesity prevention intervention)	Alternative intervention (Self-esteem and social efficacy)	BMI at 2 years, Anthropometric, body composition, dietary assessment, physical activity
	364. Kokkvoll 2014 [577]	Norway	Multiple-family intervention (MUFI) comprised a 3-day inpatient programme at the hospital with other families and a multidisciplinary team, individual and group-based follow-up visits in their hometown, weekly group based physical activity and a 4-day family camp	Single-family intervention (SIFI) comprised clinical examination and individual counselling by paediatric nurse, paediatric consultant, nutritionist at the hospital and follow-up by a local public health nurse	Height, weight, BMI, waist circumference, skin fold thickness body composition
	KOPS study<br>365. Plachta-Danielzik 2007 [578-580]	Germany	Behavior and educational messages	No intervention	BMI, triceps skinfold (TSF) and waist circumference (WC)
	366. Kubik 2008 [581]	United States of America	Clinic-based primary prevention intervention	Usual care	Relevant lifestyle practices, BMI, Children's physical activity, Sedentary practices, Eating practices
	367. Kulendran 2016 [582]	The United Kingdom	Short messaging service (SMS) sent to participants.<br>All 50 camp attenders received fortnightly calls from a MoreLife counsellor who had no stake in the experiment's outcome and would want all camp attenders to succeed. During these calls the counsellor discussed the campers' progress, social support and any potential barriers to weight loss, for about 10 min	Received three text messages per week the first on Sunday. The first text message each week asked them to commitment to act in a specific way, based on the same information given to the information group	BMI, response of counselling calls
	368. Kulik 2015 [583]	NA	Standard cognitive behavioral	Standard cognitive behavioral weight loss program	Family and friend support for healthy eating behaviors and

			weight loss program enhanced with peer support		exercise, Total energy intake, Body weight, BMI, Adherence, Dietary intake, Physical activity behavior
	369. Lee 2018 [584, 585]	United States of America	Out-of-School Nutrition and Physical Activity intervention	Received after intervention	Increased moderate and vigorous physical activity, Improved quality of food and beverage consumption
	370. Lloyd 2011 [586-593]	United Kingdom	HeLP is a multicomponent four-phase programme using a range of school-based activities including lessons, assemblies, parents' evenings, interactive drama workshops and goal setting to engage and support schools, children and their families in healthy lifestyle behaviours	Not specified	Body mass index, Waist circumference, Body fat, Physical activity, Television (TV), viewing/screen time, Food intake
	371. Lochrie 2013 [594]	NA	Family based lifestyle intervention	No intervention	Oral glucose tolerance test (OGTT) and lipid panel, BMI, Blood pressure (BP), Child depressive symptoms, Harter Self-Perception Profile (SPP), Pediatric quality of life, Behavioral Assessment
	372. Loeb 2019 [595]	NA	Family-based treatment (FBT) for paediatric obesity (FBT-PO)	Nutritional education counselling (NEC) condition	BMI
	373. Looney 2014 [596]	United States of America	Newsletter and Growth Monitoring plus Family-Based Behavioral Counseling Condition (N + GM + BC)	Newsletter Condition (N).<br><br>Newsletter and Growth Monitoring Condition (N +GM).	Child z-BMI, Child dietary intake (sugar sweetened beverages [SSBs], fruits and vegetables [FVs], energy and percentage energy from fat)<br>Leisure-time behaviors (moderate to vigorous-intensity physical activity [MVPA] and television [TV] viewing)
	374. Love-Osborne 2014 [597]	NA	The educator met with the IG during the academic year, utilizing motivational interviewing techniques to set lifestyle goals	Medical care only	BMI, Fitness Testing, Physical Education Class, and Sports Participation
	375. Luca 2014 [598]	Canada	SickKids Team Obesity Management Program (STOMP)	No intervention	Homeostatic measurement, assessment-insulin resistance (HOMA-IR), nsulin resistance (IR), Pediatric Quality of Life Teen, Report (PedsQL 4.0), BMI, BMI z-

					scores, Health behavior, Children's Depression Inventory (CDI)
	376. Lumeng 2017 [599]	United States of America	Head Start (HS) + Preschool Obesity Prevention Series (POPS) + Incredible Years Series (IYS) (HS enhanced by the POPS [program targeting evidence-based obesity-prevention behaviors] and the IYS [program to improve children's self-regulation]) HS+POPS	Head Start (HS) only	BMI z score, BMI, Self-regulation, Dietary intake, Physical activity, Sedentary behavior, Parent nutrition knowledge
	377. Mameli 2016 [600]	Italy	Smartphone applications (APP)<br><br>Wristband (WB)	Received only the Mediterranean diet and instruction to practice PA and minimize sedentary activity to follow during the 3 months study period	Difference between body weight at 3 months; BMI change; Changes in energy and macronutrients; Changes in the level of commitment to the intervention; Burden of the child with the intervention; Satisfaction with the intervention, burden of the Parents with the intervention, awareness on the Importance of lifestyle changes and change of Habits using the intervention
	378. Markert 2014 [601, 602]	Germany	Computer-aided telephone counseling	No intervention	BMI SDS, Dietary intake, Level of physical activity, Psychosocial well-being, Health-associated quality of life
	379. Marlid 2012 [603]	Sweden	Nurse–dietician managed treatment (NDT)<br><br>Nurse–dietician–physiotherapist managed treatment	Wait list (No intervention)	Puberty, BMI, Glucose, insulin, triglycerides, low- and highdensity lipoproteins, and apolipoprotein
	380. Martínez-Andrade 2014 [604]	Mexico	Educational intervention sessions (High Five for Kids Intervention)	Usual care	Physical activity, BMI, Adherence
	381. McEachan 2016 [605]	United Kingdom	Healthy and Active Parenting Programme for Early Years (HAPPY) (6 antenatal, 6 postnatal sessions)	Usual care	BMI, Home food environment, Physical activity, Parenting practices, Infant diet, Physical activity and development
	382. McGarvey 2004 [606]	United States of America	Fit Women, Infants, and Children "Fit WIC" childhood overweight prevention program	Standard WIC	Active play item, Family activity item, Television viewing item, Mealtime behavior scale, Water-

					consumption item, Fruits and vegetables item, Efficacy belief item, Outcome expectancy scale, Risk perception item, Readiness-to-change item, Client satisfaction scale
	383. Meng 2018 WAVE~Ripples for Change: Obesity Prevention in Active Youth (WAVE) program [607]	Oregon USA	encourage PA outside of sport, and teach sport nutrition and life-skills (e.g., meal planning, shopping on a budget, food preparation/cooking skills, and gardening) to support sustainable healthy eating and life-long physical activity among high school soccer players	control	Food groups intake and physical activity
	384. Moore 2009 [608]	USA	Diet therapy	Normal diet evaluation	Children's nutrition knowledge, nutrition self-care practices, physical activity, and nutrition status
	385. Mora 2015 [609]	Spain	Intervention to promote healthy diet and increased physical activity	Basic dietary advice	BMI
	386. Morandi 2019 [610, 611]	Italy	PROBIT trial (The encouraged behaviors were breast-feeding, feeding on demand, responsive feeding, timely complementary feeding, giving portions based on the child's appetite, alternating protein sources correctly, and playing active games with the child)	Usual care	Rates of overweight/obesity, Rate of beverage consumption
	387. Morshed 2018 [612]	USA	Parent led behavioral therapy + added intervention	Parent led behavioral therapy	weight, BMI, waist circumference, blood pressure, added sugars, food quantity, sedentary time on a weekday, physical activity
	388. Munsch 2008 [613]	Switzerland	CBT of mother and child (condition A)	CBT of mother only (condition B)	Children's socio-demographics and mental disorders were assessed at baseline. Children's percent overweight, depression, measures of anxiety; behavioural problems

	389. Muth 2008 [614]	USA	Improving Meals and Physical Activity in Children and Teens- IMPACT	Standard school curriculum	Change in self- reported nutrition and physical activity, BMI, BMI percentile
	390. Nardo Junior 2017 [615]	Brazil	Multidisciplinary obesity program (PMTO)-behavioral therapy	No treatment	BMI, BMI z scores, absolute body fat, absolute lean mass, cardiorespiratory fitness, adolescent quality of life, pubertal development.
	391. Nawi 2015 [616]	Malaysia	Internet based diet intervention	Printed material on dietary advice	BMI
	NiÃ±os Sanos, Familia Sana<br>392. de la Torre 2013 [617, 618]	USA	The three-year intervention included parent workshops on nutrition and physical activity; school-based nutrition lessons and enhanced physical education program for children; and a monthly voucher for fruits and vegetables	Not specified	BMI z-score, obesity
	393. Norman 2016 [619]	USA	Behavioral therapy -step-down care (SDC)	Enhanced usual care (EUC)	BMI, BMI z scores, percentage body fat, lipid profile, blood pressure, plasma glucose
	394. Nyberg 2016 [620, 621]	Sweden	Healthy School Start intervention	Waiting-list	Physical activity, dietary habits, physical activity habits, sedentary behavior and sleep, BMI standard deviation score, socio economic status.
	395. Ochoa-Aviles 2017 [622, 623]	Ecuador	Diet therapy	Normal school curriculum	dietary intake, waist circumference, change in body fat %, change in dietary habits
	396. Omourou 2015 [624]	France	Screening and care strategy –behavioral therapy	Education and environmental change	Physical activity and sedentary behavior, eating habits
	397. Poeta 2019 [625]	Italy	preschool-age prevention program (3P)	Normal school curriculum	BMI, BMI z scores, waist circumference, physical activity, dietary preferences and knowledge, screen time
	398. Rerksuppaphol 2017 [626]	Thailand	Internet based programme (diet therapy)	Verbal advice provided	Change in % of overweight and/or obese children, BMI, BMI z score, waist and hip circumference

	399. Resnicow 2016 [627, 628]	USA	motivational interviewing (MI) by primary care physician + dietician review (Group 3)<br><br>Motivational interviewing by primary care physician (Group2)	Usual care by primary care physician (Group 1)	BMI percentile, actual MI dose
	400. Reyes- Morales 2016 [629]	Mexico	Multicomponent intervention	Standard nursery practices	changes in obesogenic behaviors, such as the frequency of consumption of sugary drinks and foods with high energy density and low nutritional value, in addition to physical activity, and changes in the eating environment and physical activity in home
	401. Rohde 2017 [630]	Denmark	Children in the intervention group were assigned to a health consultant trained in nutrition and dietetics, and most children were followed by the same consultant throughout the 15 months of follow-up	Children allocated to the control group met with a health consultant twice; at the beginning of the study and at follow-up approximately 15 months after their first visit.	BMI, diet quality index (DQI)
	402. Saelens 2013 [631]	Seattle USA	motivational and autonomy-enhancing intervention (self-directed) for behavioral family-based pediatric obesity	standard prescription of uniform behavioral skills use and interventionist goal assignment (prescribed)	BMI z-score
	403. Santos 2015 [632]	Pelotas, Brazil	Nutrition counseling (breastfeeding promotion and increased intake of micronutrient-rich and energy-dense foods)	(receiving routine nutrition advic	Anthropometric measurements; body composition (air-displacement plethysmography); body shape (3-dimensional photonic scan); and plasma total, LDL, and HDL cholesterol, triglycerides, C-reactive protein, and glucose
	404. Satoh 2007 [633]	Japan	received dietary guidance using the model nutritional balance chart (MNBC)	No dietary advice given	Intake of food groups and overweight/obesity
	405. Sen 2018 [634]	Turkey	Kaledo game	Behavioral intervention	BMI, Prosocial behavior, Physical activity, Dietary assessment
	406. Schwatrz 2007 [635]	USA	Minimal intervention (pediatrician only); or intensive intervention (pediatrician and registered dietitian).	No intervention	BMI percentile

	407. Sharifi 2017 [636, 637]	United States of America	Electronic health records (EHRs) –based CDS and training for pediatricians at the point of care to promote recognition and recommended management of obesity	Usual care	Start-up intervention costs , Ongoing costs, Estimated intervention costs
	408. Sousa 2015 [638, 639]	Portugal	Participants were invited to get restricted access to the e-therapeutic platform (Next.Step), which included a diverse set of resources, such as educational resources, self-monitoring, social support, interactive training modules and motivational tools.	Standard clinical treatment	Program effectiveness, BMI, Adolescent Lifestyle Profile (ALP), Impact of Weight on Quality of Life (IWQOL)
	409. Spiegel 2006 [640]	USA	Multidisciplinary, school based intervention entitled Wellness, Academics & You (WAY)	Not exposed to the WAY program	BMI, consumption of fruits and vegetables, and physical activity
	410. Small 2017 [641]	Colorado USA	Parents attended four intervention sessions at their child's primary health care office	Age-specific health and safety education intervention.	Behavioral outcomes
	411. Stock 2007 [642]	Canada	Students in 4th through 7th grade then acted as teachers for their younger "buddies." All lessons included 3 components of healthy living: nutrition, physical activity, and healthy body image	No intervention	Healthy-living knowledge, behavior and attitude, a 9-minute fitness run, self-competence, body satisfaction, disordered eating symptoms, and anthropometry (BMI, blood pressure, and heart rate)
	412. Stookey 2017 [643]	United States of America	Child Care Health Program (CCHP) + Healthy Apple Program (HAP)	Child Care Health Program (CCHP) + Healthy Apple Program (HAP) Delayed Program	Primary outcome: Change in child, BMI percentile, BMI z-score, incidence of overweight or obesity, Secondary outcome: child's relative odds of exposure
	413. Tanofsky-Kraff 2017 [644-647]	The United States of America	Weekly psychotherapy sessions	Health education- domestic violence, alcohol, drug and tobacco, basic information on nutrition, body image, symptoms and prevalence of depression and suicide, gang violence, non-violent conflict resolution, and sun safety	BMIz, change in adiposity, eating disorder, anxiety, depressive symptoms
	414. Taveras 2015 [648, 649]	United States of America	Clinics receiving CDS only<br><br>Clinics receiving CDS + coaching intervention	Usual care	Height, Weight, BMI z score, BMI
	415. Thompson 2015 [650]	NA	All groups played the 10-episode online videogame. The groups varied only on type	Children assigned to the "Control "group played the game, but only	Child FV intake, Servings of F and regular V (i.e., non-fat, non-fried)

			of implementation intention created (Action, Coping, Both) after setting a goal to eat FV	set a goal to eat FV i.e., they did not create an action or coping implementation intention	
	416. Thakur 2016 [651]	India	Group sessions on topics such as food and nutrition, environment, physical fitness and lifestyle disorders such as obesity, hypertension and stroke	The control group did not attend any lectures at the school; however, they received information about diet and physical activity	Change in weight, Body mass index (BMI), Waist circumference Biceps and triceps skin fold thickness, Biochemical parameters, Dietary energy and fat intake, Physical activity
	417. Taylor 2015 [652]	New Zealand	Tailored package (TP) condition consisted of a single multidisciplinary consultant session (usually both parents, mentor, dietitian, exercise specialist, and clinical psychologist all together) followed by regular, brief contact (predominantly mothers only) with a MInT mentor (1 nutritionist, 1 exercise trainer)	Usual care	Height, Weight, Waist circumference, BMI, Waist to height ratio (WHtR), Motivation, and parental feeding and discipline, Level of household chaos, Quality of life, Child behavior, Dietary intake, Home food availability, Moderate-vigorous physical activity
	418. Tomayko 2019 Healthy children, strong families [653-655]	USA	The Wellness Journey consisted of 12 monthly mailed healthy lifestyle lessons, items, and children's books addressing 6 intervention targets: increase fruit and vegetable (F/V) consumption, decrease sugar consumption, increase physical activity, decrease screen time, improve sleep habits, and decrease stress (adult only)	Active control group focused on child safety (Safety Journey)	Physical measurements (height, weight, and waist circumference), sex-specific BMI percentiles, health behaviors, adult and child diet and physical activity, family home environment, adult stress and other psychosocial measures, and adult cultural identity
	419. Tucker 2019 [656]	United States of America	Behavioral counseling	Usual care	Demographics, BMI z-scores, Child/Family Behaviors, Family nutrition and Physical Activity, (FNPA), Feeding Practices
	420. Tyler 2016 [657]	USA	The participants at the intervention clinic received the five NWM intervention visits, which consisted of nurse-delivered counseling that focused on weight-related behaviors.<br>The intervention group made seven clinic visits (baseline visit with provision of weight management information, five intervention visits, and one follow-up/termination visit).	The comparison group made four clinic visits: one at baseline for data collection and weight management information and then three subsequent visits for data collection only.	Demographic, Health behavior knowledge, Attitudes about eating, Quality of life, BMI, Waist circumference, Blood pressure (BP), Clinical measures of lipid, insulin and glucose.

	421. van Grieken 2017 [658, 659]	Netherland	E-health4Uth Healthy Toddler intervention group	Usual care	Primary Outcome: Health-related behaviors, Daily breakfast, Daily consumption in glasses per day of sweetened beverages, Screen time Other outcomes: Sociodemographic characteristics, Parents' BMI
	422. Wadolowska 2019 [660]	Poland	The educated group, a diet-related and lifestyle-related school-based education program lasting three weeks was implemented	Usual care	Nutrition knowledge, Sedentary and Active Lifestyle, Diet quality, Waist-to-height ratio (WHtR), Body mass index
	423. Wald 2011 [661]	USA	Family-based behavioral weight management treatment program	Wait list control (quasi control)	Change in weight, BMI, BMI z score.
	424. Wald 2018 [662]	United States of America	Family-based behavioral model in which healthy eating and activity was encouraged and authoritative parenting was supported.	Usual care	Dietary intake, Weight, Family Eating and Activity Habits, Physical activity, Parenting Sense, Height, BMI, BMI z score, Child's degree of overweight
	425. Waling 2012 [663]	Sweden	Intervention sessions concerning food habits, physical activity and behavioral change	No intervention	Food habits, Food intake, Total energy expenditure, Anthropometrics
	426. Walpole 2013 [664]	Canada	Motivational Interviewing (MI)	Social skills training	Self-efficacy, height, weight, and waist circumference
	427. Warren 2003 [665]	United Kingdom	'Eat Smart Play Smart' Physical activity and nutrition lessons. For all intervention groups, an activity book, designed for use at home, accompanied each term's lessons.	Be Smart: Children learnt about food in a non-nutrition sense. The topics covered were food traditions, food in different countries and food processing. On alternate weeks, children learnt about the human body, using an interactive CD-Rom. Children had an activity book, which had a related homework, but it did not have weekly messages.	Physical activity, Dietary assessment, Anthropometry Nutrition knowledge, social and medical history
	428. Wylie-Rosett 2018 [666]	United States of America	Kid-WAVE introduction To foster early engagement children were given the Kid-Weight, Activity, Variety, and Excess (WAVE) Get Healthy card game	Standard care pediatrician visits	Height, Weight, Systolic and diastolic blood pressures, Fasting glucose, triglyceride (TG), total cholesterol (TC), low density lipoprotein (LDL) cholesterol, and high-density lipoprotein (HDL) cholesterol concentrations

	429. Yackobovitch-Gavan 2018 [667]	Isreal	In the parents-only group, at least one parent attended the meetings. In the parents–child group, at least one parent and child attended separate group meetings	The control group did not participate in group meetings.	Height, Blood pressure, BMI, Serum glucose, total cholesterol, triglyceride and high-density lipoprotein cholesterol levels Serum insulin concentrations
	430. Zimmerman 2012 [668]	United States of America	Participants received written materials (distributed at initial interview) and monthly newsletters about their respective behavior changes. The intervention lasted for 4 months and targeted two television viewing-related behaviors. A case manager for each group used in-person conferences, monthly newsletters, and e-mail contact to motivate behavior change around child television viewing (n=33 families)	Parents were asked to promote their child's safety in several areas, including regular use of bike helmets, regular and appropriate use of child car seats, appropriate asthma management, home fire safety, timely flu shots, gun safety, smoking cessation, safe level of hot water heater temperature, and developmental screening. A case manager for each group used in-person conferences, monthly newsletters, and e-mail contact to motivate behavior change around child safety	Amount of total television viewing Amount of commercial television/DVD/video viewing of the child
**Diet and Exercise**	431. Aceves Martin 2017 [669]	Reus, Catalonia, Spain	D and E	No intervention	>1 portion/day of fruits and >1 portion/day vegetables
	432. Alberga 2015 (HEARTY Study) [670-681]	Canada	Exercise sessions (aerobic, resistance or combined) and dietary guidance (n= aerobic training: 75, resistance training: 78, combined aerobic and resistance training: 75)	No intervention	Weight, height, waist circumference, body composition, percent body fat, Abdominal SAT and VAT Apolipoproteins, HSCRP
	433. Aperman Itzhak 2018 [682]	Israel	Physical activity and dietary prevention	Control	Food frequency questionnaire, height, weight, body fat
	434. Arauz Boudreau 2013 [683]	United States of America	Power Up classes that educated children and caregivers about healthy behaviors surrounding nutrition, activity, and stress management and culturally sensitive coaching to empower families to incorporate learned behaviors and address both family and social barriers to lifestyle changes.	No intervention	HRQoL, metabolic markers of obesity, BMI, physical activity

	435. Ariza 2019 [684]	Spain	PA + Nutrition	Control	Body composition, nutrition and PA
	436. Banks 2012 [685]	England	Primary care clinic (PCC): session with nurses that discussed overall progress, focusing on factors that facilitated or inhibited weight reduction. For diet and exercises: Age-specific approaches were used in recognition of developmental stage, with parents being the key determinants	Bristol Royal Hospital for Children (BRHC): an established service that uses a multi-component team approach in consultations with children and families	BMI SDS, quality-of life scores using the Pediatric Quality of Life Scale (PedsQL), satisfaction with care
	437. Barkin 2012 [686]	United States of America	Twelve weekly 90-minute skill-building interactive sessions for parent and child	Brief school readiness program	Height, weight, BMI, fidelity
	438. Barnett 2017 [687]	USA	Evidence Based Quality Imprvement Project	Routine care	BMI, weight
	439. Bayer 2009 [688]	Germany	The ''TigerKids'' behavioral intervention program: The program and its contents were based on the concept of age-appropriate social learning of health promoting behavior by imitation of superiors and peers and adoption the behavior of these role models	No intervention	Food frequency, high fruit and vegetable consumption, Low consumption of high caloric drinks, overweight/obesity: BMI, high consumption of low caloric drinks, low consumption of energy dense sweets, purchase of low fat milk products, infrequent consumption of unhealthy snacks in front of TV
	440. Bernsten 2010 [689]	Norway	Guided active play	Waitlist control	BMI, body composition, Physical activity
	441. Bharath 2018 [690]	USA	Combined resistance and aerobic exercise	No exercise	Braichial-ankle pulse wave velocity, blood pressure, heart rate, leptin levels, adiponectin levels and body composition
	442. Bhave 2016 [691]	India	Physical activity, diet and general health, and including increased extracurricular and intra-curricular physical activity sessions; daily yoga based breathing exercises; making physical activity a 'scoring' subject; nutrition education; healthier school meals; removal of fast-food hawkers from the	No intervention	BMI, waist circumference, physical fitness, diet and lifestyle indicators (time watching TV, studying and actively playing).

			school environs; and health and nutrition education for teachers, pupils and families		
	443. Bonis 2014 [692]	United States of America	The intervention consisted of workshops given to facility staff. The workshop topics included overweight, nutrition, physical activity, and growing healthy kids Parents/guardians also received information to implement at home	Wait list control	Physical activity, BMI, waist circumference
	444. Brandstetter 2012 [693, 694]	Germany	URMEL-ICE focused on health-promoting behaviour change in three areas: drinking sugar-sweetened beverages, spending time with screen media and being physically active. The URMEL-ICE-intervention consists of material for 1 school year including 29 teaching units (each 30–60 min), 2 short blocks of physical activity exercises a day (each 5–7 min), 6 family homework lessons and materials for the training and information of the parents	No intervention	BMI, waist circumference, skinfold thickness
	445. Bruyndonckx 2015 [695]	Belgium	dietary restriction (1500–1800 kcal/day), physical activity, and psychological support under medical supervision	Participants on the waiting list were treated by their general practitioner or paediatrician, focusing on caloric restriction and encouragement to participate in sports activities	BMI, peak oxygen uptake (VO2peak), blood pressure, markers of arterial stiffness, Reactive Hyperemia Index (RHI), Platelet poor plasma (PPP), Lipid profile
	446. Burguera 2011 [696]	Spain	ACTYBOSS (Activity, Behavioral Therapy in Young Subjects) is an incentive-driven physical activity and nutrition education program for children. The intervention involved nutritional advice, behavioral modification, and free supervised physical activity sessions, where children received points as a reward for the hours they spent exercising and also for their attendance to nutritional lectures. Participants were also offered 2 nutritional and 2 behavioral modification workshops during the intervention period.	Abandoned the study before the first day of activities	Waist circumference, Height, % BF, BMI, fasting glucose and hormonal determinations: blood count, insulin, TSH, GH, and testosterone (in boys) or estrogens (in girls)

			The ACTYBOSS exercise tool box included team sports, racket games as well as dancing and music games, it also involved parental conferences		
	447. Caballero 2003 (Pathways Study) [697-706]	United States of America	The Pathways Study intervention consisted of 4 components: classroom curriculum, food service, physical activity, and family involvement.	No intervention	Percentage body fat, dietary intake, physical activity, and knowledge,attitudes, and behaviours.
	448. Campbell 2013 [707]	Australia	The intervention comprised of six sessions delivered at three month intervals during the regular meeting time of the first-time parents' group . Based on an anticipatory guidance framework the intervention will incorporate a range of modes of delivery and educational strategies including brief didactic sessions, use of group discussion and peer support, exploration of perceived barriers, use of visual and written messages, follow-up delivery of messages by text-messaging and mail-outs.	Usual care	Child's dietary intake, physical activity, television viewing time, economic analysis, process evaluation, BMI
	449. Centis 2012 [708]	Italy	Children: 3 fortnightly meetings with children to improve nutritional practice of breakfast - with every child preparing his breakfast (in a fun manner). Provision of step counters to stimulate their daily activity.<br>Parents: Invited to 3 motivational meetings for diet & PA Counseling; Weekly telephone calls.	No intervention	Triceps skin-fold thickness, BMI, open-air games and TV watching
	450. BEACHES Study [709, 710]	United Kingdom	The intervention programme included both dietary and physical activity components, targeting children and their families. These included family cooking workshops and a range of additional physical activity opportunities within and outside of school, all delivered to varying degrees by each school	No intervention	BMI, skin fold, waist circumference
	451. Cohen 2014 [711]	United States of America	CHANGE communities- Students were primarily exposed to the intervention while	No intervention	Fruits (cups) per 1,000 kcal, vegetables (excluding potatoes)

			at school through daily access to a food service component and to an educational curriculum every week on average. The cafeteria changes included offering whole grains daily; providing five different fruit and vegetable options weekly (with a fresh fruit or vegetable option daily, and a dark green or orange vegetable or fruit at least three times per week); providing beans or peas weekly; supplying 1% and nonfat milk daily; limiting ice cream sales; and encouraging a healthier à la carte portfolio Shape Up- During- and after-school curricula, the Eat Well Keep Moving curricula (both curricula were based on the social-cognitive theory), and the 5-2-1 messages. CHANGE study also included parent and community outreach components throughout the school district.		(cups) per 1,000 kcal, fruits and vegetables combined (cups) per 1,000 kcal, whole grains (oz)per 1,000 kcal,s legumes (cups) per 1,000 kcal dairy (cups) per 1,000 kcal. potatoes (cups) per 1,000 kcal energy from saturated fat (%)added sugars (tsp) per 1,000 kcal fiber (g) per 1,000 kcal glycemic index
	452. Cohen 2016 [712-716]	Canada	The dietitian guided families to either provide their child with two servings (StnTx) or four servings (ModTx) of milk and alternatives/day, preferably consuming products with lower percentage of milk fat (%MF) Both groups were encouraged to meet current PA guidelines (60 minutes of moderate-to-vigorous activity/day) and limit screen time (<2 hours/day); daily weightbearing activities (i.e., skipping rope, jumping types of activities) were emphasized in ModTx. (n=Standard group (StnTx): 25 , Modified group(ModTx): 25)	No intervention	Weight, height, BMI, BMI-for-age z-scores (BAZ) and height-for-age z-scores (HAZ), body composition, fat mass,lean mass, percent body fat, trunk fat mass, android/gynoid ratio and fat mass index (FMI)
	453. Coppins 2011 [717]	The United States of America	The intervention involved two Saturday morning workshops and attendance at two physical activity sessions throughout the 1-year intervention. Workshops took place in a school (4–12 participants plus 2–10 parents/guardians and siblings involved) and focused on healthy eating, physical activity, reducing sedentary behaviour,	Received no active input	Change in BMI SDS, waist circumference, body fat, lifestyle outcomes

			behaviour change and psychological well being		
	454. Crespo 2012 [718, 719]	The United States of America	Sessions, home visits, Newsletters, recipe cards, goal setting, Booster phone calls (4 calls over 2 years), Teachers' discipline and classroom practices, School playgrounds and salad bars; community parks	The control condition consisted of measures only. Participants<br><br>in the control condition were asked to maintain their regular lifestyles and to complete the yearly measurements	Parent andc BMI, children's physical activity, children's sports participation, active transportation to and from school, children's dietary intake, tv viewing, availability and use of active toys, parental support for child physical activity, family meals together, away from home eating
	455. da Silva 2013 [720]	Brazil	The intervention group participated in curricular and extracurricular activities for nutrition education (50 min once a week) and physical activity (50 min twice a week)	Participapted in regular curricular activities	BMI, total body fat percentage,physical activity, type of physical activity (light, moderate and intense); hours spent watching TV, playing video games or using the computer; four physical fitness tests: PACER—progressive aerobic cardiovascular endurance run; the push-up test; the curl-up test; the back-saver sit and reach test
	456. Davis 2009 [721]	The United States of America	Group class: Participants in the group class met monthly (classes lasted 90 minutes) at the Veronica Atkins Lifestyle Intervention Laboratory (VALIL) and received a<br>monthly class that was similar to their 4-month intervention classes (i.e., either N only or N+ST). They included a cooking component, a snack, a nutrition lesson (focused on reducing sugar and increasing fiber intake), and a 45-minute strength training session (for those subjects in the N+ST group)	Newsletter group: Participants in the newsletter group received a monthly<br>newsletter in the mail that matched their 4-month intervention group assignment (i.e., either a nutrition specific newsletter or a nutrition + strength training newsletter). The newsletter covered basic tips on how to continue to eat foods and drink beverages low in sugar and high in fiber and included one or two new low-sugar or high fiber recipes.	Height, weight, blood pressure, body composition via BodPod™, lipids and glucose/insulin indices via frequently sampled intravenous glucose tolerance test (FSIVGTT)
	457. Davis 2016 [722-727]	The United States of America	The six components of the CHILE intervention are<br><br>nutrition and physical activity curriculum	No intervention	BMI z-score

			quarterly professional development training<br><br>a component focused on integrating policy and behavior change in food purchasing, preparation, and serving by HS food service staff;<br><br>a family component consisting of take-home materials about nutrition and physical activity and family events reinforcing these messages twice during the school year;<br><br>a local grocery store component<br><br>a component that asked local healthcare providers to emphasize healthy eating and physical activity during routine patient visits and invited health professionals to attend CHILE family events to show support for the intervention.		
	458. Davison 2015 [728-732]	The United States of America	Behavioral counselling<br>MA-CORD intervention communities: Intervention activities implemented in health centers include:<br><br>1. advanced training on clinical quality improvement and obesity prevention, assessment, and management through learning communities;<br><br>2. computerized, point-of-care decision support tools for clinicians through electronic health records (EHRs);<br><br>3. multidisciplinary weight management programs housed within the health center (i.e.,healthy weight clinics); and<br><br>4. environmental changes within the health centers to support behavior change	No intervention	Mean child BMI, and rates of child overweight and obesity, along with five evidence-based behaviours that support obesity prevention and control in children, including increased consumption of fruits and vegetables and decreased consumption of sugar-sweetened beverages (SSBs), increased PA, decreased screen time, and improvements in sleep duration and quality, quality of life and quality of care

	459. De Coen 2012 [733]	Belgium	Sessions for guiding and training teachers. The intervention was based on the 'Nutrition and Physical Activity Health Targets' of the Flemish Community clustered into: 1. increasing daily consumption of water and decreasing soft drinks consumption; 2. increasing daily milk consumption; 3. increasing daily consumption of vegetables and fruit; 4. decreasing daily consumption of sweets and savoury snacks; and 5. increasing daily PA and decreasing screen-time behaviour.	No intervention	BMI and BMI z-score
	460. De Henauw 2015 (IDEFICS) [734-764]	Belgium, Cyprus, Estonia, Germany, Hungary, Italy, Spain and Sweden	A coherent set of intervention modules were developed by a team of experts, focusing on diet, physical activity and stress-coping capacity (including sleep quality)	No intervention	BMI z-score, body fat (peripheral/ central), body fat (central/peripheral), body fat % (slaughter), body fat mass (kg), waist/hip, waist/height, weight/height ratio, BIA (bioelectrical impedance analys
	461. de Niet 2012 [765, 766]	Netherlands	Group sent weekly self-monitoring data on exercise and eating behaviour and their mood via mobile phones. In return, they received tailored feedback messages.	No intervention	Weight, eating behaviour and psychological well-being, i.e. competence, self-esteem and quality of life, adherence to the SMSMT
	462. de Silva-Sanigorski 2010 [767, 768]	Australia	(CoGG) and (BoQ) 2 year olds : 1587 and 3.5 year olds: 1194): Sessions, dietary plan and education, behavioural management, Structured Active Play Program	No intervention	Weight (in kg), BMI (in kg/m2), zBMI, weight status
	463. Dewar 2013 [769-777]	Australia	NEAT Girls4- Intervention components included enhanced school sport sessions, lunchtime physical activity sessions, nutrition workshops, interactive educational	No intervention	BMI, BMI z-score; percentage body fat (bioelectrical impedance analysis); physical activity (accelerometers); dietary intake;

			seminars, pedometers for self-monitoring, student handbooks, parent newsletters, and text messages to reinforce and encourage targeted health behaviors		and recreational screen-time (self-report).
	464. Diaz 2010 [778]	Mexico	Family-centered program consisting of 12 sessions of behavioral curriculum, dietary advice from a registered dietitian (weekly for the first 3 months and monthly thereafter), and monthly consultations with a primary care physician.	Participants attended monthly consultations with a primary care physician	BMI, BMI z-score, body weight, waist circumference, body fat, blood pressure, glucose, triglycerides, total cholesterol, high-density lipoprotein cholesterol, and low-density lipoprotein
	465. Dietrich 2008 [779, 780]	Austria	lessons consisted of 12 sessions, one hour per week in each class (9 nutrition and 2 health related lessons) and one final session with a healthy school lunch. A playful, experience-oriented learning, through sensory exercises was preferred to its cognitive counterpart.	No intervention	Knowledge about nutrition eating habits, consumption of food, BMI
	466. Donnelly 1996 [781]	The United States of America	Changes in the school lunch program, and increasing structured physical activity concentrating on large muscle groups (3 days per week)	No intervention	Body weight, body composition, metabolic fitness, and aerobic capacity
	467. Duggins 2010 [782]	The United States of America	Nutrition classes (4 sessions); healthy snack preparation; Free YMCA family membership and encouragement for its use	No intervention	BMI percentile<br><br>BMI, food type scores, servings
	468. Economos 2007 [783-787]	The United States of America	Newsletters, monthly media piece, before school breakfast program, walk to school campaign, professional development, school food service, classroom curriculum, enhanced recess, school wellness policy, after school curriculum, professional development for all program staff, walk from school campaign, parent outreach and education, family events, parent nutrition forums, community advisory council, walking training	No intervention	BMI, weight change

	469. Elder 2014 [788]	The United States of America	Telephone survey about the families' recreation centre use followed by an introductory group workshop at the recreation centre, and a home visit. Tip sheets offered simple strategies for parents to use to promote healthy eating and physical activity in their children. Motivational interviewing, self-monitoring and goal setting	No intervention	BMI
	470. Eliakim 2002 [789]	Israel	Evening lectures, dietitian visits (once a month), training sessions for PA (twice weekly)	Outpatient nutritional consultation at least once every 3 months and were instructed to perform physical activity three times per week on their own	BMI
	471. Epstein 1994 [790]	Not specified	Experimental family-based treatment: Written information was provided in a manual that was divided into five units (Introduction and Self-Monitoring, Diet, Exercise, Parenting, and Maintenance). Each unit contained smaller modules, with a total of 22 modules. The behavioral skills for parents and children were divided into five levels. Parents in the experimental treatment were also asked to master parenting skills of praise and stimulus control within a separate level system. Common components of treatment were diet: exercise and behavioral change	The same lectures were given to parents and children, for behavior change. Parents were given the list of suggested parenting skills and self-monitored their use of praise and stimulus control techniques. However, they were not praised by program staff for changes in child and parent behavior. Parents in the control group were told that parent-child nightly meetings were important for child weight loss but were not instructed to institute specific contingencies during evening meetings. Lottery chances were provided in this group for attendance, rather than changes in parenting	BMI, adherence to treatment and knowledge of parenting
	472. Epstein 2000 [791]	The United States of America	The 6-month treatment program included 16 weekly meetings followed by two monthly meetings. Families were seen at 12 and 24 months after randomization for follow-up treatment and data collection.	No intervention	Height, weight, fitness, physical activity

	473. Epstein 2005 [792]	The United States of America	Session for nutrition and physical activity counselling with reward points	No reward points	Height, Weight, BMI, BMI z score, physical activity, eating episodes and patterns, socioeconomic status
	474. Epstein 2015 [793]	The United States of Americas	Both groups attended the sessions and were given same treatment.<br>Families in FBT + Variety group developed meal plans that repeated entrees and included leftover foods, reduced variety of RED foods, and were encouraged to repeat weekly meal plans over the month.	Same as intervention but the foods were not repeated	Weight, Height, BMI, z BMI, percent overweight, Parent BMI, adherence
	475. Epstein 1990 [794]	The United States of America	Sessions on Diet and Nutrition Education, exercise and behavioral procedures (contracting, self monitoring, social reinforment and prompts, therapist contact and contingency management) In group 1, the intervention to loss weight and change habit was for both the parents and the children; in group 2, the intervention was only for the child	Families attended meetings but had no intervention	Height, Weight, Percent overweight
	476. Fairclough 2013 [795, 796]	The United Kingdom	The CHANGE! Project is a school-based physical activity and healthy eating intervention study delivered through the PSHE strand of the primary school curriculum. Teacher-led curriculum, learning resources, and homework tasks (weekly lessons)	No intervention	BMI
	477. Fitzgibbon 2005 [797-804]	The United States of America	Teachings sessions, physical activity sessions, Newsletters with homework (for parents). Children in the six WCI schools participated in a 14-week healthy eating and exercise intervention. Thrice Weekly session covering:<br>1. 20-minute healthy eating and exercise lessons<br>2. 20-minute exercise activity	Children in the six GHI schools received a 14-week class in which they learned about a variety of general health concepts such as dental health, immunization, seat belt safety, and 911 procedures. Parents received weekly newsletters that mirrored the GHI. No information on diet or physical activity was presented.	BMI

			Parents received weekly newsletters on healthy eating and exercise. it also included homework assignment		
	478. Flattum 2011 [805]	The United States of America	Program components included the New Moves physical education class (4 days/week for one semester), which incorporated nutrition and social support sessions (1 day/week); individual counselling sessions that incorporated motivational interviewing strategies; weekly lunch get-togethers after the physical education class ended; and minimal parent outreach	No intervention	Physical activity, fruit and vegetable intake, breakfast consumption, portion control, satisfaction with individual sessions
	479. Foster 2016 [806, 807]	United States of America	Parent mentors trained in positive-deviance behaviors and conducted follow-up phone calls at least monthly to encourage healthy habits and behaviors identified as foci for that particular parent. Intervention parents also participated in monthly community meetings at Head Start centers	Monthly community meetings at Head Start centers	Body mass index z-score change, feeding behaviors and practices, health-related quality of life, dietary intake participation levels.
	480. Fotu 2011 [808, 809]	Tonga	Nutrition education; exercise promotion, policy modification. Media reports and promotions (TV and Radio); vegetable gardens; sports competition; trees planting; sports training sessions; provision of sports equipment	No intervention	Overweight and obesity prevelance
	481. Francis 2010 [810, 811]	Trinidad	Nutrition and physical activity lessons	No intervention	BMI, Soda intake, vegetable intake, physical activity, television viewing
	482. French 2011 [812]	The United States of America	6 monthly face-to-face group sessions (week day), monthly newsletters, and 12 home-based activities	No intervention	HH mean BMI z-score, changes in food choices, PA, self-weighing (adults only), and TV viewing behaviors.
	483. French 2016 [813]	The United States of America	Home environment intervention to reduce television viewing with locking devices and displace availability of sugar sweetened	No intervention	Television viewing, beverage intake, BMI

			beverages with home delivery of non-caloric beverages		
	484. Gallotta 2015 [814]	Italy	Traditional Physical activity (PA)<br><br>Coordinative PA	Traditional physical exercise school curriculum	Children's weight, height, BMI z-score, lean body mass and body fat mass percentage (FM%), Sedentary time, Eating habits of children, sustained attention and concentration under stress
	485. Gallotta 2016 [815]	Italy	Physical exercise and Nutrition intervention	Traditional physical exercise school curriculum	Children's weight, height, BMI z-score, lean body mass and body fat mass percentage (FM%), Sedentary time, Physical activity, Eating habits of children
	486. García-Hermoso 2014 [816]	Spain	Diet (low-calorie diet) and Exercise (It comprised a warm-up (15-20 minutes), a main part consisting of pre-sports and multi-sports games with a moderate to vigorous intensity aerobic component (60-65 minutes), and a cool-down (5-10 minutes))	Exercise only	Eating habits, Daily physical activity, Pubertal status, Body height, Body weight, Body fat mass, Physical fitness
	487. Garipağaoğlu 2008 [817]	NA	Family-based group treatment	Individual treatment (standard group)	BMI, Energy quantity
	488. Gentile 2009 [818, 819]	The United States of America	The Switch program promoted healthy active lifestyles by encouraging students to 'Switch what you Do, Chew, and View'. The specific DO, VIEW, and CHEW goals were to be active for 60 minutes or more per day, to limit total ST to 2 hours or fewer per day, and to eat five fruits/vegetables or more per day. The intervention utilized overlapping behavioral and environmental strategies employed at multiple ecological levels	Control schools did not receive any school materials	Physical activity, standing height and weight, BMI, screen time, fruit and vegetable consumption
	489. Gillis 2007 [820]	Jerusalem	Both groups were given a basic discussion on healthy diet and exercise. The intervention group was asked to also record the contents of food ingested and amount of exercise performed on one day of each week. These children received a weekly telephone call. During the call, diaries were reviewed and the children were encouraged to improve their adherence to the prescribed	Controls were given a basic discussion on healthy diet and exercise	BMI, lifestyle changes, physical fitness, biochemical parameters

			plan. After 3 months, children from both groups were again seen in the clinic for a talk on healthy diet and exercise and for physical examination. Six months after enrolment, participants from both groups were invited for a third clinic visit.		
	490. Gortmaker 1999 (Planet Health Study) [821-824]	The United States of America	Schools received the Planet Health Program	Usual health curricula	Obesity, television viewing, physical activity, total percentage of dietary intake from fats, servings of fruits and vegetables, total energy intake
	491. Gray 2015 [825]	The United States of America	The intervention was a science and nutrition education curriculum designed to impact middle school students' EBRBs: eating more fruits and vegetables, drinking more water, increasing physical activity and decreasing intakes of sweetened beverages and packaged snacks, eating at fast food restaurants and leisure screen time. Social cognitive theory and self-determination theory together served as the behavioral change theoretical framework. The curriculum addressed theory constructs such as self-efficacy, autonomy and competence.	The control schools received regular science curriculum during the same period, and received the delayed intervention in spring 2007	Behavioral and psychosocial outcomes.
	492. Grydeland 2013 [826-833]	Norway	Teachers were responsible for holding one structured lecture on energy balance for the students, initiating "HEIA-breaks" - a 10 minute physical activity break during class at least once a week, hanging up "HEIA-posters" in the classrooms, carrying out active commuting campaigns, handing out fact sheets to parents once a month (including student-parent tasks in 7th grade), and implementing a computer tailored program (in 7th grade only) for the students. The intervention schools received an "Activity box" with sports equipment and toys (such as balls, hockey-sticks, jump	No intervention	School activity, physical activity, sedentary time

			ropes, Frisbees, etc.) to promote physical activity during recess.		
	493. Habib-Mourad 2014 [834-836]	Lebanon	The intervention specifically targeted obesity-related behaviours in 9–11 year olds including: increasing consumption of fruits and vegetables, favouring healthy over high energy dense snacks and drinks, increasing the habit of having breakfast daily, increasing moderate-to-vigorous physical activity (MVPA), and decreasing overall sedentary behaviour.	Usual curriculum	Height, weight, and waist circumference, dietary habits, nutrition knowledge, physical activity
	494. Harder-Lauridse 2014 [837]	Denmark	The intervention consisted of<br>1. 60 minutes weekly group training session<br>2. 90 minutes weekly group training session of the children, their parents, and siblings at a municipal fitness club<br>3. individual nutritional guidance and coaching of the children and their families<br>4. common cooking and dining with the children and their families	No intervention	BMI, physical activity, blood pressure, waist circumference, waist-height ratio (WHtR)
	495. Hasson 2012 [838]	USA	Nutrition and strength training	Nutrition only	strength, dietary intake, body composition (dual-energy X-ray absorptiometry/magnetic resonance imaging) and glucose/insulin indexes (oral glucose tolerance test (OGTT)/intravenous glucose tolerance test (IVGTT)) and inflammatory markers
	496. Hendy 2011 [839]	The United Sates of America	The KCP group (called the 'LIONS'') received stars punched into their nametags for each of three ''Good Health Behaviors'' that included eating 1/8 cup FV (''the size of a ping pong ball'') first during their meal	The control group (called the ''TIGERS'') received stars punched into their nametags for each of three ''Good Citizenship Behaviors'' that included talking quietly during	Changes in BMI%, changes in children's EXERCISE, changes in children's FVFIRST and HDRINK, height, weight

			(FVFIRST), choosing a low-fat and low sugar healthy drink (HDRINK), and having 5000 exercise steps recorded on their pedometers (EXERCISE)	meals, keeping their meal area clean, and respecting others by not touching them or their things	
	497. Hollar 2010 [840-843]	The United Sates of America	HOPS included a set of interventions: provision of nutritious ingredients and whole foods in breakfasts, lunches, and extended day snacks, which modelled nutrition education in the classrooms; the incorporation of a holistic curricula that taught children, parents, and school staff about good nutrition, healthful lifestyle management, and increased levels of physical activity; and the implementation of other school-based wellness activities such as fruit and vegetable gardens.	No intervention	BMI percentile, academic performance (math scores, reading scores)
	498. Iaia 2017 [844]	Itaky	Motivational interviews, information tools (leaflets, manuals, posters), learning experiences at childcare centres for parents and learning experiences and active play for children	Routine healthcare	Children's eating habits and activity behaviours, active play and TV watching time, height, weight, parent's height, parent's weight, parental and teacher degree of appreciation for intervention
	499. Janicke 2008 (Project STORY) [845-847]	Not specified	Changes in dietary habits were addressed via a modified version of the Stoplight Diet. Increased physical activity was promoted through a pedometer-based step program FB: Parent and child dyads participated in simultaneous but separate groups.<br>PO: Only the participating parent(s) attended group meetings	Wait list control	Height, weight, dietary intake, BMI
	500. Jiang 2007 [848]	China	Nutrition education, traffic light food item for overweight and obese children. Meeting for oveweight/obese children and their parents separately. Nutrition and PA counselling overweight/ obese children who failed to pass routine school physical	No intervention	height, weight, BMI, % overweight/obese

			education tests were asked to run for 20 min after class (50-70% compliance)		
	501. Jiang 2005 [849]	China	Psycho: family based behavioural therapy; goal definition; interval behaviour definition; exercise counselling (20–30 minutes per day for four days per week (three weekdays and one day on weekends) was advised).<br><br>Diet: Diary, counselling, traffic light diet, family dietary practice monitoring and counselling	No intervention	Height, weight, BMI, BMI z-score, SBP (mm Hg), DBP (mm Hg), cholesterol (mmol/l), triglyceride (mmol/l)
	502. Johnston 2010 [850-853]	The United States of America	All children at the school received a snack during this period, though the study staff provided ILI children with peanuts/peanut butter and a fruit or vegetable to enhance satiety and to provide an opportunity for fruit/vegetable consumption. During the 12-week intervention, participants received nutrition instruction and physical activity training. In addition, parents were invited to attend monthly meetings to teach them how to adapt family meals and activities to facilitate healthy changes. Nutrition instruction focused on teaching participants to make healthier food choices from the options available to them by reading labels and controlling the portion sizes of their foods	Self Help (SH): Children in the SH condition used a parent-guided manual intended to promote child weight loss and long-term maintenance of changes. Participants and their parents were provided Trim Kids, a book to be used for weight management in children and their families. The parent-led program consisted of 12 weekly sessions followed by maintenance activities for improving diet and level of physical fitness of children	Weight, height, bmi, tricep skinfold, cholesterol analysis (total cholesterol, triglycerides, high-density lipoprotein cholesterol, and calculated low density lipoprotein cholesterol), blood pressure, heart rate
	503. Kain 2004 [854]	Chile	Nutrition education for children and parents, 'healthier' kiosks, 90 min of additional physical activity (PA) weekly, behavioral PA program and active recess.	No intervention	Adiposity indices (BMI, BMI Z-score, triceps skinfold thickness (TSF), waist circumference and physical fitness (20m shuttle run test and lower back flexibility)
	504. Kain 2014 [855]	Chile	Nutrition education, 8 sessions of 90 min each on health eating for the children (6 hrs of training), exercise counselling, kiosk counselling	Regular curriculum	BMI z-score and obesity prevalence, children's knowledge on healthy eating, types of foods brought to school, and degree of implementation by teachers of the

					educational contents on healthy eating and physical education
	505. Kipping 2014 (Active for Life Year 5 Study) [856-862]	England	Teacher training, provision of lesson and child-parent interactive homework plans, all materials required for lessons and homework, and written materials for school newsletters and parents.	No intervention	Mean time per day spent doing moderate/vigorous physical activity, sedentary activity, servings of fruit and vegetables consumed per day, mean time spent screen viewing, servings of snacks consumed per day, high fat foods consumed per day, high energy drinks consumed per day, BMI, waist circumference, overweight/obesity
	506. Kobel 2014 [863-868]	Germany	The intervention is based on teaching materials offering action alternatives for recreational activities (without screen media), physical activity, and a healthy diet (focusing on breakfast and soft drinks) which are integrated into the primary school curriculum.	No intervention	Physical activity (PA), screen media use (SMU), regular breakfast, and consumption of soft drinks (SDC)
	507. Kocken 2016 [869]	Netherlands	Theory lessons and practical assignments), attitude (group discussions, food diaries), social norm (group discussions and homework assignments) and perceived behavioral control (modeling through assignments e.g., preparing a healthy meal and physical activity games).	No intervention	Nutrition, physical activity, sedentary behavior (inactivity and screen time) and behavioral determinants, BMI (prevalence of overweightness/obesity), waist and hip circumference
	508. Kong 2013 [870]	The United States of America	ACTION consisted of eight visits using motivational interviewing. 3 primary components:<br>1. clinical encounters with the SBHC clinician<br>2. use of motivational interviewing (MI),<br>3. obesity risk reduction strategies from a toolkit. The toolkit	Standard Care Group (SCG)- Participants received one clinic visit at the beginning of the trial that was similar in content to the first visit of the intervention group except they were not given the DVD or DVD player	Weight, height, waist circumference, blood pressure, insulin resistance, behavioral measures (dietary, physical activity, and television viewing), process evaluation.

			included a DVD and print materials to provide a "menu of options" during clinical encounters<br><br>Students brought home a newsletter to their caregivers that included obesity risk reduction strategies for the home. After each visit, telephone updates were given to the caregiver, during which the SBHC clinician used MI to encourage caregivers to adopt the risk reduction strategies		
	509. La Rowe 2010 [871]	The United States of America	Families received 12 lessons on nutrition and physical activity. In addition, they receive three group-activity lessons designed to help intervention families support one another in making and sustaining healthy lifestyle choices. Year 2 of the intervention consists of monthly newsletters for the intervention and control groups and monthly group-activity lessons for the intervention families	Families received the same 12 lessons by mail	Dietary intake of children, physical activity, time spent in sedentary activity
	510. Larsen 2018 [872]	NA	Small-sided ball game group (SSG)<br>Circuit strength training group (CST)	Not specified	Lean body mass (LBM), Weight, Height, Postural balance, Maximal horizontal jump, Gross motor skill, Player load
	511. Leach 2008 [873]	The United States of America	The minimum core nutrition intervention applied equally to all participants involved a parent/guardian and the child and included a handout, Tips for Families, as well as a 15-minute DVD lesson.	Wait list control	BMI, impact evaluation
	512. Lee 2014 [874]	China	Intervention group received 4 months intensive intervention. The program consisted of ten 75-minutes after school sessions and 3 hours weekend session of practical interactive and fun activities covering the topics on healthy eating, exercise and positive self-image. The sessions included 5 nutrition education sessions, 1 session on body image and self-	Wait list control	BMI z-score and body fat percentage, attitude and behavioural variables.

			esteem, 1 session on sport safety and 4 sessions of supervised physical activity.<br><br>Parents of intervention group received an introductory seminar on the basic principles, skills and knowledge on weight management at the beginning of the programme		
	513. Lee 2012 [875-880]	The United States of America	Aerobic exercise and resistance exercise	No intervention	Visceral fat
	514. Leme 2016 [881-884]	Brazil	Healthy Habits, Healthy Girls–Brazil (H3G-Brazil): The H3G-Brazil program was a 6-month multi component school-based intervention guided by the social cognitive theory (SCT). Accredited dietitians delivered the nutrition workshops and the interactive seminars, and they were responsible for sending the WhatsApp® messages and newsletters	Control school received a condensed version of the program after follow-up assessments. This included professional learning workshops for control school teachers and the H3G-Brazil intervention materials	BMI, BMI z score, waist circumference, various sedentary and dietary health-related behaviours.
	515. Li 2014 [885]	China	Multi component physical activity intervention that included Physical education (PE) improvement, extracurricular Physical Activity (PA) for overweight/obese students, PA at home, and health education lectures for students and parents.	Usual care	Changes in BMI and overweight/obesity prevalence
	516. Lioret 2012 [886-892]	Australia	The intervention focused on parenting skills and strategies, including parental modeling, and aimed to promote development of healthy child and parent behaviors from birth, including healthy diet, increased physical activity and reduced TV viewing time.	Usual care, and newsletters<br><br>regarding generic issues in child health were sent<br><br>to participating families three monthly.	Healthy eating, increased physical activity,and reduced TV viewing time.
	517. Lison 2012 [893]	Spain	Study volunteers belonging to GRX and HOX groups and their parents jointly attended two 1-hour educational sessions. The topics covered included the importance of weight loss and its maintenance, a therapeutic nutritional approach to childhood obesity, and the role of PA in	Control group participants were instructed about diet and other lifestyle changes during their regular visits to the hospital, but neither received the exercise nor the nutrition educational sessions as for the intervention groups. Control	Height, weight, BMI, BMI z score, %BF, waist circumference

			cardiovascular fitness. The dietary intervention focused on the promotion of the Mediterranean diet. This diet, in addition to regular PA, emphasizes the consumption of abundant vegetables, fresh fruit for dessert, olive oil as the principal source of fat, regular consumption of dairy products (principally cheese and yogurt), fish and poultry consumed in low to moderate amounts, 0 to 4 eggs consumed weekly, and a reduced intake of red meat. Families were provided with additional nutritional instruction, including interpretation of food labels and shopping, and were taught stimulus control to reduce access to high-calorie foods and increase access to healthy lower-calorie foods. Participants were also encouraged to reduce sedentary behavior.<br><br>Exercise: GRX subjects were provided with 5 supervised exercise sessions. The participants and their parents were strongly advised to attend a minimum of 3 sessions per week (minimum attendance rate). HOX group participants were instructed to complete all exercises in their home environment. Their program also consisted of 5 sessions per week.	group participants maintained their usual levels of daily activity, with no additional exercise components	
	518. Llargues 2011 [894-896]	Spain	Every classroom used 3 h a week to develop activities related to health food habits and/or physical activity. This time was part of regular classes math, science, language, knowledge of the environment developing posters, food tables, games, crafts, cooking workshops and promotion of games in the playground. The intervention group of each school was given educational material on healthy food, as well as educational material on games to promote physical activity during break time. Each school was also offered the necessary equipment for these games. Each family in the intervention	No intervention	Difference in BMI progression, Weight, Height, Food habits

			group received monthly recipes for a balanced diet taking into account traditional food habits. The families also received a guide of the local areas and paths to exercise during weekends and books about balanced eating were recommended		
	519. Lopes 2016 [897]	Brazil	The combined training protocol was composed of resistance training and aerobic training performed in the same session, three times a week, during approximately 60 min and divided into three stages, each consisting of 4 weeks of training	No intervention	Height, weight, BMI, blood samples (total cholesterol, triglycerides and high-density lipoprotein levels,c-reactive protein, interleukin-6, tumour necrosis factor alpha, interleukin-10, resistin, leptin and adiponectin levels), cardiorespiratory fitness and maximal strength assessments, dietary assessment
	520. Lubans 2012 [898-900]	Australia	The PALs program10 was a multi-component school based intervention that included school sport sessions, physical activity and nutrition handbooks, interactive seminars, lunch-time activities, leadership sessions, and pedometers for self-monitoring. The program was guided by SCT13 and the intervention components involved a variety of behavior change strategies focused on the promotion of lifetime and lifestyle activities	No intervention	BMI, physical activity
	521. Lynch 2016 [901]	The United States of America	Healthy Habits Survey: designed to address health habits of children including physical activity, screen time, takeout or fast-food intake, frequency of eating meals together as a family, fruit and vegetable intake, sugar containing beverage intake, and the presence of a television or computer in the bedroom	No intervention	Health behaviour, BMI, serving of fruit/day

			Educational Curriculum :The curriculum involved 8 sessions anchored around the 5-2-1-0 curriculum		
	522. Malakellis 2017 [902]	Australia	Food at School policy: The following strategies were used to address this objective:<br>1. increased collaborations with local food producers and Nutrition Australia to create healthy food policies;<br>2. commitment to increasing healthy food consumption among staff and students;<br>3. a focus on the relationship between health and food across all areas of the curriculum;<br>4. traffic light colour coding of food sold at the school canteen by nutrition content;<br>5. provision of healthy foods and the reduction of unhealthy foods at school events;<br>6. healthy morning teas for staff to encourage positive role modelling;<br>7. cooking classes outside school hours<br>8. increased access to water fountains within the schoolyard	No intervention	Weight and Height, BMI, health behaviour, dietary and physical activity behaviours, mental well being
	523. Manger 2012 [903]	The United States of America	The VITAL curriculum consists of 8 different weekly lessons that teach young children healthy eating and appropriate physical activity. Participation in a variety of instructive fun games and physical activity captures the attention of children and is largely responsible for acceptance of	Usual curriculum	BMI

			the program. Each child received ‘’The Berenstain Bears and Too Much Junk Food’’ book, which is read in class and at home with their parents. A central and extremely valuable component of VITAL is the opportunity for constructive discussion between children and teachers about a healthy lifestyle. Each lesson ends with a question-and-answer session		
	524. Marcus 2009 [904]	Sweden	Physical activity and dietary intervention	Usual care	Physical activity, BMI, Eating habits, Prevalence of overweight and obesity
	525. Moir 2016 [905-912]	New Zealand	- Food, activity, and breastfeeding (FAB): The FAB intervention focused on breastfeeding and timely introduction of solids, healthy family foods, and physical activity.<br>- Sleep: The sleep intervention focused on antenatal and early postnatal education about preventing sleep problems with optional behavioral strategy interventions from 6 months of age for those parents who considered their infant to have a sleep problem<br>- Combination (FAB and sleep)	Control participants received usual care from the government-funded ‘’Well Child’’ care service, which typically includes seven visits beginning from 2 to 4 wk postnatally until 2 yr of age and covers a wide variety of issues including standard growth and development assessments, interventions where indicated, and support and guidance on a range of health issues including physical activity	Tummy time, maternal and partner physical activities, physical activity
	526. Mo-suwan 1998 [913]	Thailand	Exercise program included a 15-min walk before the morning class began and a 20-min aerobic dance session after the afternoon nap 3 times/wk.	Regular school schedule which included 1 h of physical education per week	Weight, height, BMI, triceps skinfold thickness
	527. Murphy 2009 [914, 915]	The United States of America	Exercise intervention (EX) using an active video game 5 days a week.Subjects were encouraged to use DDR 5 days per week. EX subjects recorded daily DDR use and steps they took while playing DDR	Delayed treatment-DTC subjects were instructed to maintain current PA levels for the intervention period.	Weight, height, BMI, tanner growth staging, step counts/physical activity, vascular testing, flow-mediated dilation, total cholesterol, high-density lipoprotein cholesterol, triglycerides, insulin, glucose, no2+no3, asymmetric dimethylarginine, aymmetric

					dimethylarginine, i-arginine, tumor necrosis factor alpha, interleukin-6, c-reactive protein, adiponectin, assessment of aerobic exercise capacity
	528. Natale 2014 [916-923]	The United States of America	Treatment or intervention arm schools received a combination of<br>1. implementing a daily curricula for teachers/ parents (the nutritional gatekeepers);<br>2. implementing a daily curricula for children;<br>3. technical assistance with meal and snack menu modifications<br>4. creation of a centre policy for dietary requirements for meals and snacks, physical activity and screen time	Schools received an attention control safety curriculum delivered by character "Safety Sam," which provided parents and teachers with home, car, and child seat safety information. They received also received the same incentives as the intervention arms to foster involvement and ensure retention/reduce loss to follow up.	Child measures included consumption of fruit/ vegetables, consumption of junk food, and sedentary behavior.<br>parents' and teachers' measures include consumption of fruits/vegetables, consumption of junk food, and physical activity. consumption of fruits/vegetables and consumption of junk food
	529. Nemet 2005 [924, 925]	Israel	Lecture, dietary counselling, dietary plans, exercise (training program) for 3 months.	Referral to an ambulatory nutritional consultation at least once and instructions to perform physical activity 3 times per week on their own.	BMI, body fat, television viewing, habitual physical activity, fitness, parental obesity
	530. Nerud 2017 [926]	The United States of America	The Make a Move intervention consisted of four sessions. The ''WE CAN'' curriculum is comprised of activities that encourage families and caregivers to make healthy food choices, engage in physical activity, and reduce youth screen time. Parents were asked to select one goal each week related to healthy eating or activity and were encouraged to track their progress toward achieving this goal.	No intervention	Parent knowledge, attitudes, and behaviours of healthy eating and physical activity
	531. Nguyen 2012 [927-932]	Australia	In the first 2 months (phase 1), participants received adolescent and parent weekly sessions focused on lifestyle modification. From 2 to 24 months (phase 2), adolescents	No intervention	Changes in BMI z score, waist to height ratio, changes in metabolic, psychosocial, behavioral variables.

			attended booster sessions. During phase 2, adolescents randomized to the additional therapeutic contact arm also received telephone coaching and electronic communications.		
	532. Nollen 2014 [933]	The United States of America	Both conditions included three 4-week modules that targeted fruits/vegetables (FVs; Weeks 1–4); sugar-sweetened beverages (SSBs; Weeks 5–8); and screen time (Weeks 9–12).	Girls randomized to the control condition received manuals at Weeks 1 (FV); 5 (SSB); and 9 (screen time).	device utilization and effect size estimates of FVs, SSBs, screen time, and BMI
	533. Novotny 2015 [934, 935]	The United States of America	At baseline, children in the treatment group received a welcome letter, handout on recommended (DASH) eating pattern, DASH of Aloha cook book, Farmers Market locations, and a PA location/map in the study informational packet	Child and parent received a welcome letter and attention control mailings on unrelated health topics, such as importance of hand washing, sun protection, and dental hygiene. At the end of the maintenance measure of the study (15 months), children in the control group received the full package of materials received by the treatment group	Body size and BP, BMI
	534. Nystrom 2017 [936-941]	Sweden	The intervention included 12 themes (healthy foods in general, breakfast, healthy small meals, physical activity and sedentary behavior, candy and sweets, fruits and vegetables, drinks, eating between meals, fast food, sleep, foods outside the home, and foods at special occasions), with a new theme being introduced biweekly. Each theme consisted of general information, advice, and evidence-based strategies on how to change unhealthy behaviours. According to personal preferences and at least once a week, parents were encouraged to register information within the application on their child's intake of fruits, vegetables, candy, and sweetened beverages as well as time spent being sedentary. At the end of every week the parents received graphic feedback and automated comments	The control group received a pamphlet on healthy eating and physical activity in preschool-aged children based on the existing guidelines, which is similar to what parents receive from the Swedish child health care system.	Body fat, intake of fruits, vegetables, candy, and sweetened beverages and the amount of time spent sedentary and in moderate-to-vigorous physical activity (MVPA)

			based on the submitted information. Within the application parents could contact a dietitian and/or a psychologist to ask questions pertaining to their child.		
	535. Obert 2013 [942]	France	Aerobic exercise; Total daily calorie intake was controlled at about 2300-2500 kcal, according to age and French recommendations for daily allowances	No intervention	longitudinal strain, peak and time to peak strain rate and twist/untwist rate,Peak and time to peak longitudinal and circumferential strains and rotation/twist, conventional echocardiography and tissue doppler data
	536. O'Connor 2013 [943]	The United States of America	Sessions and telephone calls to target dietary and physical activity behaviour. Once a month session and call	Wait list control	Height, weight, BMI z-score, physical activity, dietary intake, dietary data, TV viewing, parenting practices related to the targeted child behaviours
	537. Parra-Medina 2015 [944]	The United States of America	Behavioral intervention: Parents and children assigned to INT received all elements of SC, plus face-to-face counselling, telephone counselling, and newsletters. Face-to-face counselling: at the clinic. Telephone counselling: monthly telephone counselling calls - from health educator. Newsletters: The newsletters also featured age appropriate examples of fun, interactive family activities, and healthy food and snack recipes.	Standard care	Weight, waist circumference, and zBMI, sedentary behavior, sugar sweetened beverage (SSB) consumption, and fasting glucose and insulin
	538. Poeta 2013 [945]	Brazil	Physical exercises with recreational activities, and nutritional counselling to children and parents, lectures, meetings	Conventional treatment	Health-related domains of quality of life of the PedsQL
	539. Pope 2016 [946]	The United States of America	The intervention group received education on good nutrition and active lifestyle.	No intervention	BMI, blood pressure, fitness levels,
	540. Puder 2011 (Ballabeina Study) [947-950]	Switzerland	Children: PA Program - 4 x 45minute sessions per week; availability of sports equipment; 22 sessions on healthy nutrition, media use, and sleep. Every other week	Regular curriculum	Aerobic fitness, BMI, motor agility, balance, percentage body fat, waist circumference,<br>physical activity, eating habits,

			children received a new funny physical activity or nutrition activity card to take home. A CD with specific music for most physical activity cards was created to increase leisure and define the minimal time the activity should be performed. Teachers: two workshops before the intervention to learn about the content and the practical aspects of the intervention. Parents: three interactive information and discussion evenings about promotion of physical activity, healthy food, limitation of TV use, and importance of sufficient sleep. Information leaflets, speakers available to answer questions Environmental factors: Fixed and mobile equipment such as climbing walls, hammocks, balls, cords, or stilts were installed or provided in and around classrooms poster of the "Ballabeina track" "Ballabeina game"		media use, sleep, psychological health,and cognitive abilities.
	541. Quattrin 2014 [951-953]	United States of America	Sessions emphasized on parenting and behavioral strategies to promote child and parent behavior change, including parenting-related techniques (selective ignoring, time out, praising, rewarding, contracting) and strategies aimed at changing parental behavior that would facilitate parent and child change (pre-planning, stimulus control, shaping, modeling, self-monitoring, changing the home environment, social support, and changing black and white thinking).	60-minute sessions over 6 months in which a consistent group leader delivered dietary/physical and sedentary activities education, and trained staff engaged the children in active games.	BMI, z-BMI, height, weight
	542. Rifas-Shiman 2017 [954-958]	The United States of America	The overarching model for this intervention was the Chronic Care Model. Intervention practices received primary care restructuring, and families received motivational interviewing by clinicians and educational modules targeting television viewing and intakes of fast food and sugar-sweetened beverages. During the 1-year intervention period, we aimed for	Usual care	BMI z-scores, weight, height, age- and sex-specific BMI z-scores, changes in duration of television viewing and intakes of fast food and sugar-sweetened beverages

			participants to complete four in-person visits and two phone calls with clinicians. During the subsequent 1-year maintenance period, we aimed for participants to complete two in-person intervention visits.		
	543. Rodearmel 2006 [959]	The United States of America	EXP families were asked to maintain their usual eating and step patterns for the first week of the study to establish a baseline and then to make two specific lifestyle changes. First, each participating family member was asked to in- crease walking by at least 2000 steps/d above his or her individual baseline level. They were encouraged to continue to gradually increase steps per day as much as possible. Second, each participating family member was asked to consume 2 servings cereal/d, one at breakfast and one for a snack	CON families were asked to maintain their usual eating and step patterns throughout the 14-week study. All CON families attended three group meetings (at the beginning, middle, and end of the study) with a member of the research team. These sessions were used to take measurements, collect family data on steps and cereal servings, answer questions, and encourage continued participation in the study.	Percentage BMI-for-age, body weight, percentage body fat, cereal consumption, dietary intake
	544. Rodearmel 2007 [960]	The United States of America	AOM Group: Families were asked to make 2 small lifestyle changes.<br>- Physical Activity Changes: All AOM families were asked to wear pedometers during the first 2 weeks of baseline measures and throughout the 6-month intervention period<br>- Dietary Changes: Each AOM family participant was instructed to eliminate 420 kJ/day (100 kcal/day) from his or her usual diet; replacement of dietary sugars alone or in food products with Splenda no-calorie sweetener packet or granular products or sucralose-containing beverages was emphasized	Self monitor group (SM)	BMI for age,change in the following anthropometric measurements: BMI (children), weight, percentage of body fat, and waist circumference
	545. Rooney 2005 [961]	The United States of America	Pedometer only (P): received Digi-walker pedometer + instruction to walk 10,000 steps daily.	No intervention	Change in weight, BMI

			Pedometer plus education (PE): same as group P + sessions (nutrition, PA, and other parenting issues)		
	546. Rosenkranz 2010 [962, 963]	The United States of America	The intervention consisted of three main components:<br>1. An interactive educational curriculum<br>2. Troop meeting policies implemented<br>3. Badge assignments completed at home with parental assistance.<br>Troop meeting policies included: 1) Providing 15 minutes per meeting for physically active recreation; 2) Troop leaders participating in physically active recreation with girls; 3) Provision of a FV snack prepared by girls; 4) Troop leaders eating FV snack with girls; 5) Troop leaders verbally promoting PA, FV consumption in troop meetings and for home, and verbally promoting FM for home; and 6) Prohibition of SSB, candy, and TV watching during meetings.	Standard care	Troop Environmental Observations, Study's reliability, Body mass index (BMI), Accelerometry, Fruits and vegetables consumption, Sugar-sweetened beverage consumption, Physical activity, Family meals, Fruit servings and vegetable servings
	547. Rush 2012 [964-968]	New Zeland	Classes modelled included fundamental movement skill training, ideas for 'huff and puff' fitness activities, modified games, and ball activities and sport-related games, where keeping children moving as much as possible throughout each session was the focus. Energizers promoted active transport, lunchtime games, bike days and leadership training for students to be leaders of physical activities before and after school. Energizers were also available to assist each school with a range of healthy-eating initiatives. There was also a home–school link programme that provided opportunities for parents to attend three	Control schools were given no additional resourcing or information; however, no restrictions were placed on initiatives they may have pursued for themselves.	Height, BP, body fat (%BF), BMI, overweight/obesity

			information-based sessions, which included a 45 min practical nutrition class.		
	548. Saelens 2002 [969]	The United States of America	HH intervention: The intervention included interaction with a computer program designed for overweight adolescents and physician counselling in the paediatric primary care clinic, followed by 4 months of telephone- and mail-based behavioral counselling to achieve dietary and physical activity changes. Telephone calls would last 10 to 20 minutes.	Typical care: a single session of physician weight counselling	BMI z-score, physical activity, sedentary behavior, problematic eating and weight-related behaviors and beliefs, physician counselling, behavioral skills use, and participant satisfaction
	549. Saelens 2011 [970]	The United States of America	Children and parents were encouraged to complete daily food logs. In both conditions, children and parents were instructed in the use of a modified Stoplight Eating Plan. Children and parents in both conditions worked toward meeting 3 specific eating/weight goals introduced in week 2 and continuing through the end of treatment: 1) reducing calories to 1000–1200/day for children and 1200–1400/day for parents on at least 5 days/week, 2) reducing red foods to ≤ 15 servings/week, and 3) reducing weight (0.3kg/week). Children and parents in the ADDED condition were instructed to increase physical activity up to at least 90 minutes of moderate-to vigorous physical activity per day on at least 6 days each week. To help families in the ADDED condition meet this recommendation, children and parents were each 1) provided with pedometers, 2) asked to set weekly and long-term physical activity goals and contingent rewards based on physical activity goal attainment, 3) instructed in ways to increase both lifestyle and structured physical activity.	In the STANDARD condition, children and parents were encouraged to get at least 60 minutes of physical activity on most days of the week, and were simply asked to check off in their daily logs if they obtained this level of physical activity each day.	Child body composition, change in parent body anthropometrics and child and parent physical activity and dietary behaviours
	550. Saelens 2013 [971]	Not specified	Prescribed approach- Weekly session with the first 5 weeks about behavioral modification for a better diet. After 5 weeks,	Self directed approach- Standard of care: first 5 weeks were identical to the intervention group	Child and parent BMI z-score

			participant autonomy and self-efficacy were encouraged.		
	551. Safdie 2013 [972, 973]	United States of America	The basic program focused on improving norms related to nutrition and physical activity at the schools and was limited to using existing school infrastructure and resources. The plus program implemented all the components incorporated in the basic program and included additional financial investment and human resources.	No changes were made to existing nutrition or physical activity practices in control schools	Food and beverage availability at school, food intake at recess, physical activity, BMI (for obesity and overweight), weight, height
	552. Sahota 2001 [974, 975]	England	The intervention schools received the active programme promoting lifestyle education in schools (APPLES) The programme consisted of teacher training, modifications of school meals, and the development and implementation of school action plans designed to promote healthy eating and physical activity over one academic year	The comparison schools continued with their usual health curriculum, without the intervention	Height, weight, BMI, physical activity, psychological well being
	553. Salazar 2014 [976]	Chile	a) Educational material: Educative Guidelines in Nutrition and Physical Activity. Further counselling was carried on weekly basis to clarify doubts during the intervention.<br>b) Educational, playful motivational strategy for the parents and families, called "Healthy Days". Educational leaflets containing information on growth, nutritional factors and physical activity needs, were designed and provided to parents, in order to minimise additional consumption of energy dense food to the normal pattern (breakfast, lunch, milk and bread plus dinner) and to avoid excess TV time (> one hour daily), and encouraging daily play time	No intervention	Body fat, physical activity pattern and energy, food intake
	554. Sanigorski 2008 [977-979]	Australi	Be Active Eat Well intervention program<br><br>1. Nutrition strategies: dietitian support, nutrition strategies, training for canteen staff; Canteen menu changes; Healthy lunch	No intervention	Differences in the increases in anthropometry (weight, waist and body mass index (BMI)-z score) over time and the relationship between baseline indicators of

			packs; Interactive, glossy, children's newsletters; Teacher fliers, Promotional materials; Happy healthy families program; Parent tips sheets; Healthy lunchbox tip sheets; Community garden; Choice chips program<br>2. Physical activity strategies: After-school activities program; Be Active Arts program; Walking school buses; Walk to school days; Promotional materials; Sporting club equipment; Two class sets of pedometers for rotation between schools<br>3. Screen time: TV power-down week, including a 2-week curriculum; Interactive, glossy, children's newsletters; Teacher fliers		children's household socioeconomic status and changes in children's anthropometry, Safety measures
	555. Santos 2014 [980]	Canada	The program content focused on physical activity, promoting healthy foods, and having a healthy body image using the slogans: "Go Move!" (activity), "Go Fuel!" (nutrition), and "Go Feel Good!" (body image). In schools randomized to the intervention, an older class was paired with a younger class. Each week, the older students received a 45-minute healthy living lesson from their classroom teacher. Later that week, the older students acted as peer mentors, teaching a 30-minute lesson to their younger "buddies." The "Go Move!" aspect included two 30-minute structured aerobic fitness sessions per week, called fitness loops, with the student pairs. The"GoFuel!"component included lessons about distinguishing nutritious from unhealthy (nutrient poor, energy-rich) foods and beverages. As part of the "Go Feel Good!" component, students were taught to value themselves and classmates based on individual traits rather than peer influence.	Waiting list control group receiving a regular curriculum	BMI z score, waist circumference, physical activity, cardiorespiratory fitness, self-efficacy, healthy living knowledge, self-reported dietary intake
	556. Schwingshandl 1999 [981]	Austria	Dietary intervention: Both groups participated in the same dietary education programme. General dietary advice was given by group teaching. This included:	Dietry advice alone	Changes in BMI-SDS and fat free mass

			energy requirements; relation of the different nutrients (protein, fat, and carbohydrates) in a balanced diet (20%/30%/50% of total energy); and the importance of fibre, vitamins, minerals, and fluids. Energy intake was restricted to 4180 kJ/day in both groups; children older than 14 years were restricted to 5016 kJ/day (girls) and 5852 kJ/day (boys).<br>Training programme: Training sessions took place twice weekly in a public gym, with a duration of approximately 60–70 minutes for each session		
	557. Serra Paya 2015 [982, 983]	Spain	The NP is an intensive, family-based multi-component, behavioural intervention in primary care settings, consisting of 4 components: (a) Supervised physical activity sessions for children, (b) Family theoretical and practical sessions for parents, (c) Behaviour strategy sessions for both children and parents, and (d) Weekend activities.	Counselling group: Eight monthly, 10-minute, structured, family meetings were scheduled with the child's paediatrics unit. The content of these family sessions was the same as the Nereu Program intervention	Height, BMI, waist circumference, waist-to-height ratio, dietary intake
	558. Shalitin 2009 [984]	Israel	or both diet and exercise – a combination of the program of the other two groups (D+E),	exercise only (E), diet only (D);	anthropometric variables, cardio-metabolic profile and psychological outcome
	559. Shamah Levy 2012 [985, 986]	Mexico	The intervention was implemented in the 30 schools in the IG for a period of 3 weeks in each school; implementation of the strategy was conducted for 6 months.<br>The ongoing activities in schools in the IG were:<br>a) Nutrition and physical activity workshops.<br>b) Puppet Theatre, based on the theory of peer learning<br>c) Two-day workshops in each school to raise awareness about healthy eating and physical activity	No intervention	BMI, food intake, physical activity, knowledge, self-efficacy

			d) Sale of fruits, vegetables and pure water in the school's store cooperative<br><br>e) To promote the consumption of pure water, spots were broadcast using the schools' PA systems, and water bottles were delivered to children and teachers to encourage water consumption<br><br>Activities with the educational community included:<br>f) Physical activation<br><br>g) Broadcasting of audio spots on the schools' PA systems.<br><br>h) Organized games during break (once per week).<br><br>i) Placement of banners at the entrance of the school<br><br>Activities with parents included:<br>j) Delivery of recipe calendars		
	560. Sherwood 2013 [987-989]	The United States of America	Counselling session and phone coaching to prevent obesity by making dietary changes and exercise. Six biweekly calls for the first 3 months and then 8 monthly calls for the remainder of the year	Safety/Injury Prevention Contact Control arm	Child BMI percentile, dietary intake, accelerometry, diet-related factors, television and media use, parent/guardian BMI, caregiver's feeding styles, child feeding, children's eating behavior, parental healthy eating, parent physical activity, media availability and household media rules, parental importance and enjoyment of physical activity, parental support for physical activity, play equipment availability, parent physical activity with child, restaurant behavior, parent weight loss, three-factor eating questionnaire, parenting styles and dimensions, children's sleep habits, pediatric quality of life inventory, parent

					report for young children, fire prevention, injury prevention, safety equipment ownership and use, distracted driving, vehicle safety, parental support for safety, parental safety self-efficacy, pediatric gastrointestinal symptoms, carbon monoxide safety, water/ice safety, sun safety, secondhand smoke, family disaster plan, firearm/gun safety, internet safety, intervention cost
	561. Shofan 2011 [990]	Israel	double hours of weekly, physical education lessons and additional nutritional advice, regular parents meetings, which occurred once a month for one hour per session for 10 months a year, the paediatrician and the dietitian encouraged healthy dietary habits for 2 years; eight nutritional education lessons and double the physical education hours as compared with the control group	Standard physical education lessons only	BMI percent, weight, height
	562. Singhal 2010 [991]	India	Nutrition education, physical activity and counselling sessions, lifestyle intervention included 24 weeks (6 months) of nutrition education; subsequent 18 weeks were used for intensive and repetitive nutrition education to all the eleventh-grade students.	No intervention	BMI, waist-to-hip ratio (W-HR), waist-to thigh ratio (W-TR) and waist-to-height ratio (W-Ht ratio), body composition, knowledge, attitude and practices and body image satisfaction surveys, biochemical measures
	563. Skouteris 2016 [992]	Australia	Guided active play; healthy snack time, supervised creative play activities for the children, interactive education and skill development session. 10 weekly 90-min workshops relating to nutrition, physical activity, parenting and lifestyle behaviours	Wait list control	Child daily dietary intake, child eating habits, physical activity and sedentary behaviours, child BMI z-score, child food neophobia
	564. Slawson 2015 [993]	The United States of America	Posters, self monitoring forms, pedometers,self-efficacy, social support,weight teasing and weight perceptions including self-reported body	Standard curriculum	Body mass status, dietary behaviors, PA, and sedentary behaviors, attitudes and beliefs on weight control, perceived behavior control/self-efficacy, perceived

			weight and weight concerns, and HRQoL are also key elements		behavioral control (PBC) for healthy eating and physical activity,<br>attitude towards healthy eating and physical activity, perceived group norms of eating and physical activity, social support, weight teasing and weight perception, self-reported body weight and weight concern,<br>unhealthy dieting,<br>health-related quality of life (HRQoL), and<br>dental health
	565. Small 2014 [994]	The United States of America	Educational information about the establishment of healthy habits in young children, nutritional information, information regarding increasing physical activity and decreasing sedentary time, and age-specific information regarding the child's behavior in response to change	Parents were encouraged to make health and safety goals for their family (e.g., development of first-aid materials and identification of a fire escape plan).	Body mass index percentile,<br>waist circumference,<br>waist/height ratio
	566. Sosa 2016 [995-997]	The United States of America	The ¡Míranos! program includes both center- and home-based activities. Center-based activities include health education, gross motor activities, and parent newsletters. Home-based activities include peer-led parent obesity education and take-home activities.	Usual care	Weight-based z-scores, raw scores of gross motor skills, children's responses to míranos, evaluation of fidelity and feasibility
	567. Stettler 2015 [998]	The United States of America	Multiple behavior interventions included: imparting knowledge and skills; behavioural changes: sessions / conference calls;<br>Combined beverage-only and multiple behaviours interventions group<br>Beverage-only intervention group<br>Multiple behavior intervention group	No intervention	Difference in BMIz incidence of obesity, changes in unadjusted BMI and changes in skinfold thickness

	568. Story 2012 [999]	United States of America	1. Physical activity sessions<br>2. healthy eating interventions<br>3. Family-focused intervention	No intervention	Mean BMI, mean percent body fat, prevalence of overweight children (BMI ≥85th percentile and <95th percentile for age and gender), and the prevalence of obese children (BMI ≥95th percentile for age and gender), percentage of calories from fat and nutrient content in school meals, duration of physical activity at school, and food intake at home.
	569. Suarez-Balacazar 2014 [1000]	United States of America	Twice a month for 5 months the team came into the kindergarten and first grade classroom to deliver an education session about specific fruits and vegetables. These sessions lasted about 30 to 45 minutes and covered the following: (a) identification of fruits and vegetables (F=V), (b) importance of F=V to health by highlighting one food item each month in an age-appropriate manner, and (c) a container gardening project in the classroom in which the students learned how to plan tomatoes which were placed by a large sunny window in the classroom. At one of the sessions, researchers partnered with an African American rural community farmers co-op to visit with the students and demonstrate farming skills and vegetable crops. Team members used handouts with large and colourful pictures, actual fruits and vegetables, and mostly visual material to introduce the F=V.	No intervention/nutrition education in schools. The team delivered the same nutrition education initiative to the comparison school once this project was completed, except for a visit from the farmers coop	Changes in knowledge pre and post nutrition education
	570. Tarro 2014 [1001-1004]	Spain	The EdAl program consisted of interventional activities of educational value focusing on eight lifestyle topics. These topics are based on scientific evidence indicating the value of improving nutritional food item selection; healthy habits such as teeth brushing and hand washing, and, overall, adoption of activities that encourage physical activity (e.g.,	No intervention	Obesity prevalence, Changes in BMI z-score, Waist circumference, Hip circumference, BMI incidence and remission (i.e., the participant's change from OB status to OW or to normal weight) of excess weight

			walking to school, playground games); and avoidance of sedentary behavior. These topics were covered in 12 activities (1 hour/activity/ session) over three school academic years.		
	571. Taylor 2006 [1005-1010]	New Zeland	APPLE (A Pilot Programme for Lifestyle and Exercise) is a community-based demonstration project. Wherever possible, the focus was on encouraging lifestyle-based activity rather than structured sports only, both to widen exposure to a variety of activities and to engage those children not interested in traditional sporting activities. Such activities included golf, taekwondo, community walks, beach hikes, school triathlons, line dancing, children's games from other countries, and parent and child team sports. ACs were employed for 20 hours per week, providing activity programmes for 8 hours and promoting activity in the community or undertaking administrative duties in the remainder of their time. Additional initiatives introduced in the second year include activities promoting reduction of intake of sugary drinks; increase in fruit and vegetable consumption; reduction of television time, and short activity breaks in class. In addition, physical activity was enhanced by providing sport and play equipment during lunchtime.	No intervention	Height, waist circumference, pulse rate, Blood pressure, BMI, physical activity, sedentary behavior
	572. Taylor 2017 [1011]	New Zeland	Lactation consultation along with advice on breast feeding and weaning	Standard care	Body weight, length, BMI, BMI z-score, infant temperament, fine and gross motor skills, parental feeding behaviour and perception of "picky eating", adverse events, eating behaviour, acceptability to parents of the complementary feeding (amongst other issues), dietary assessment, energy intake,

					complete blood count, plasma ferritin
	573. Toulabi 2012 [1012]	Iran	The ‘‘behavior modification’’ interventional program was implemented as follows:<br>1. it comprises a 24-hour diet record for the students and their parents;<br>2. it provides face to face nutritional instructions for the parents supported by an educational booklet;<br>3. it provides face-to-face nutritional instructions for the students regarding dietary modification and techniques for increasing physical activity supported by an educational booklet;<br>4. it provides exercises demonstrated by the physical education expert at school in a group<br>The exercise program consisted of warming up for 10 minutes, performing aerobic exercises for 40-45 minutes, and cooling down for 5-10 minutes, respectively, and included rapid and vigorous walking, running, rope jumping, zigzag movements, high-knees, butterfly movements, stepping exercises, and exercises for strengthening important muscles.	Provided with educational booklets after data collection.	Body weight, height, BMI, waist and hip circumferences, WHR
	574. Tucker 2011 [1013, 1014]	The United States of America	All children (control and intervention) received classroom delivery of the Let’s Go 5-2-1-0 Program curriculum by the PHN. Intervention children also received 1:1 student nurse coaching, parent evening offerings, and reinforcement incentives.	No intervention	BMI, BMI percentile, Healthy habits, Physical activity

	575. Van Grieken 2014 [1015, 1016]	Netherlands	The intervention protocol is based on theories and models of behavioral change, i.e. the ASE model. It aims to promote the development of a healthy weight: i) playing outside for at least 1 h a day, ii) having breakfast daily, iii) drinking no more than 2 glasses of sweet beverages, and iv) limiting television time to a maximum of 2 h a day.Together with the parents, youth health care professionals chose one or two behaviors to target during the well-child visit and/or the additional intervention sessions. Parents drew up a family-oriented action plan on the health behavior change they wanted to achieve in their family	Usual care was given: i.e. the well-child visit, during which parents were offered general information about healthy nutrition and physical activity.	BMI and waist circumference, child health behaviours
	576. van Nassau 2014 [1017, 1018]	Netherlands	DOiT effectiveness programme: daily breakfast consumption as an additional potential target behaviour, and a parental component to the programme.<br><br>A 5-minute instruction video to guide teachers through the programme and materials. (1) reducing intake of SCB; (2) reducing intake of high-energy snacks/sweets; (3) reducing screen time; (4) increasing levels of physical activity (i.e., active transport to school and sports participation) and (5) consuming a daily breakfast	No intervention	Body height and weight, skinfold thickness, and waist circumference, dietary and physical activity behaviours
	577. Vanhelst 2011 [1019]	France	CEMHaVi consisted of 2 h per week of physical activity and a 2 h session every 3 months for health education.Health education manuals included the following four chapters: (i) How My Heart Works, (ii) Physical Activity, (iii) Nutrition, and (iv) Stress.Physical activity consisted of a 2-h exercise session every week in a gymnasium, primarily in the form of games. The exercises were of moderate intensity, that is, 40–45% of peak maximum oxygen uptake.During the 2-week school holiday,	Controls received normal care of a specialist physician in pediatrics.	Age, height, weight, BMI, resting systolic and diastolic blood pressures

			one exercise session of 2 h was provided to be in contact with children and parents.		
	578. Verbestel 2014 [1020]	Belgium	Posters, feedback forms: increasing daily consumption of water (instead of soft drinks), milk, fruit and vegetables, increasing daily physical activity and decreasing daily consumption of sweets and savoury snacks and daily screen-time behaviour.	standard care of therapy	BMI, BMI-z score, water consumption (ml/d), fruit consumption (g/d), vegetable consumption (g/d), unsweetened milk consumption (ml/d), sweetened milk consumption (ml/d), soft drink consumption (ml/d), sweets and savoury snacks (g/d), physical activity (h/d), screen-time behaviour (min/d)
	579. Veugelers 2005 [1021]	Canada	Two categories of schools were created with nutrition programs. The first included schools reporting that they had policies or practices in place to offer healthy menu alternatives. The second included 7 schools that are part of a coordinated program incorporating aspects of each of the CDC recommendations for school based healthy eating programs.This initiative developed into Annapolis Valley Health Promoting Schools Project (AVHPSP)	No nutrition program	Number of daily servings of fruits and vegetables, percentage of calorie intake from dietary fat, overall dietary quality, dietary adequacy, variety, moderation, and balance, physical and sedentary activity, BMI
	580. Vilchis-Gil 2016 [1022, 1023]	Mexico	The intervention was implemented in 2 schools,1 governmental and 1 private.<br><br>Activities of Parents in the Intervention Group:<br>1. Website. An interactive workshop to learn and use the project website was developed. Parents were invited to access the site at least once every 2 weeks and review the current topic.<br>2. Messages by mobile phone. A short message (25 words on average) was sent to the parents' mobile phone, every week. The message was related to the current topic of the website that motivated and reinforced behavior changes. A total of 40 messages were sent	No intervention	Weight, height, waist circumference, BMI, BMI z-score

			3. In-person activities. Parents from the intervention schools were invited to 3 sessions of 1 hour each, once every 2 months, for the purpose of giving them feedback on topics of eating and physical activity that were on the website, to participate in some project activities, and to answer their questions and receive comments to modify or improve the website.<br>Activities of Children in the Intervention Group<br>1. Workshops. The workshops were integrated with both board games and physical games and with educational materials to reinforce healthy eating habits and physical activity.<br>2. Educational materials. They were provided with board games and plastic place mats with pictures of a healthy dish, the pyramid of physical activity, and a picture of a healthy drink pitcher.<br>3. Visit to Universum Museum. Children and parents visited the Life in Balance room of Universum, the Science Museum of the UNAM.<br>4. Posters. Each month, in a visible area and an area with large influx of children inside the school (eg, at the entrance of the school and in the schoolyard) a new poster alluding to the project and specifically the current website topic was placed.		
	581. Wake 2009 [1024, 1025]	Australia	GPs used a brief, solution focused approach to set and record appropriate, healthy lifestyle goals, assisted by a 16 page "family folder" written at a 12 year old reading level to be sure that virtually all parents could understand it.	Standard of care	BMI, child waist circumference, maternal and paternal BMI, physical activity (accelerometer, 4 day activity diary; parent report), nutrition (4 day activity diary), health status (PedsQL 4.0), Body dissatisfaction (body figure perception questionnaire), physical appearance and self worth

	582. Waling 2010 [1026]	Sweden	Group sessions (total 14: 5 x food habits, 9 x physical activity behavioural changes & self esteem)	Controls were given an 1 hr session at beginning in which they were told about the study and its measurements	Effect of the intervention on dietary intake was change in EI
	583. Wang 2015 [1027]	China	1. Comprehensive intervention group: combination of PA and diet interventions<br>2. PA only intervention group based on the Happy 10 exercise program<br>3. Diet only intervention group based on nutrition education program	No intervention	Height, weight, BMI and waist circumference (WC), body composition, blood pressure, biochemical indicators
	584. Waters 2018 [1028]	Australia	Interventions to improve physical activity, healthy eating, health promotion, and well being	Normal school activities and programs for health promotion	Change in adiposity, BMI, Intakes of calories, fat, and fruits and vegetables, Television-viewing time
	585. White 2004 [1029-1031]	Not specified	The HIP-Teens program-<br><br>The Internet-based behavioral intervention included the provision of nutrition education plus an internet counselling behavior modification program that targeted the lifestyle eating and physical activity habits of the overweight adolescent and obese parent.<br><br>Weight Management Programs: The two secured web sites were designed to present culturally specific information, including links to other web sites containing culture-specific information such as recipes for foods commonly eaten by African Americans. Each family was assigned a counsellor who conducted face-to face sessions and corresponded regularly using e-mail.<br><br>Internet-Based Behavioral Intervention. This intervention was based on the family treatment methods. The web site provided	Internet-Based Control Intervention: Participants were provided health education in a coordinated program between face-to-face sessions and links to a variety of web sites promoting healthy lifestyle. The control web site did not provide explicit prescriptions for behavior change, behavioral contracts, or opportunities for self-monitoring. Instead, the control web site was designed to be a passive (non-interactive) program that provided useful health education for the parents and the adolescents by electronic links to other health-related web sites.	Change in body fat, dietary self-efficacy, program adherence, multipass 24-hour recall psychological indices

			nutrition education and behavior modification for adults and adolescents using a family-oriented format. Counseling for behavior modification was accomplished primarily by asynchronous e-mail communications. Along with e-mail, the web site contained a variety of interactive components. Participants could also self-monitor their food intake by entering foods consumed into the web site. They also received feedback on it. Participants were also instructed to read 52 lesson plans during the 1st year of the program.		
	586. Wright 2014 [1032]	United States of America	The Kids N Fitness intervention group (KNF) intervention had two components, a family-centered educational lifestyle program, providing physical activity and nutrition education, and school-level environmental activities at the school site. Sessions consisted of three components: physical activity, nutrition education=behavior modification, and family involvement. During the exercise portion parents were taught the implications of obesity in children and adults, and the importance of healthy lifestyles to prevent obesity. In addition parents participated in a parent support group. Following the exercise, children and parents were given a 45-minute nutrition education behavioral modification session.<br><br>Environmental Activities: At each intervention school participating children received health services, including a full physical exam. A School Health Advisory Council (Advisory Council) was created at each intervention school to discuss the overall issues of wellness, health promotion, and disease prevention for their school population.	The general education group participated in the standard physical activity program given by their respective schools and did not receive any physical or nutrition education.	Body mass index (BMI), child physical activity behavior, including: daily physical activity, team sports participation, attending PE class, and TV viewing computer game playing, Intervention Fidelity

	587. Xu 2014 [1033-1036]	China	Four components: 1) classroom curriculum (including physical education and healthy diet education); 2) school environment support; 3) family involvement; and 4) fun programs/events.	They followed their usual health education curriculum without additional intervention	Body weight, height, waist circumference, dietary intake, physical activity, sedentary behavior, obesity related knowledge
	588. Yang 2017 [1037]	Korea	The participants in the intervention group received environmental intervention in addition to the usual school curriculum. Based on the pre-intervention results, personalized suggestions for improving physical strength and dietary habits and information on the daily caloric requirement were provided to individuals in the form of a handout. In each classroom, educational dietary and exercise videos and the daily lunch menu were presented 5–10 min a day by internet protocol television service for 1 academic year. Various design materials were also painted along the school staircase and hallway to encourage physical activities by the students.<br>During the vacation period, for students who are already overweight or obese (BMI 85th percentile), a once-weekly obesity care program was held during the 12-week summer vacation.	No intervention	BMI z score, dietary habits, weight-related outcomes (for example, weight, WC, percent body fat, and prevalence, incidence, and remission rates of overweight or obesity), BP, Physical fitness
	589. Tooty Fruity Vegie [1038-1040]	Australia	Fundamental movement skill development through prescribed games; Workshop for parents; newsletters; posters food and nutrition policies	No intervention	Weight status, fundamental movement skills (FMS), lunch box audits of parents, children's food intake, physical activity and sedentary behaviours
**Diet Only**	590. Akdemir 2017 [1041]	Turkey	Nutrition intervention	Routine activities	Weight, BMI, nutrition, exercise
	591. Alaimo 2013 [1042]	The United States of America	A modified experimental<br>intervention and a natural experiment, whereby schools<br>were encouraged to make self-selected nutrition policy and practice changes and followed over time	No intervention	Food Frequency Questionnaires outcomes

	592. Albala 2008 [1043]	Chile	Substituting SSBs with milk	None	Height, weight, lean mass, body fat
	593. Andersen 2016 [1044]	Denmark	Participants consumed 1l/d of skim milk, whey, casein or water for 12 weeks	No intervention	Total energy intake, dietary intake, BMI
	594. Ask 2010 [1045]	Norway	Free lunches-The offered lunch consisted of whole-meal bread, different kinds of cheese, cold cuts of lean meat, fish and jam. Low-fat milk and fresh fruit and vegetables were also served	No intervention	Height, weight, BMI, food score, self-perceived school behaviour
	595. Bean 2012 [1046-1049]	The United States of America	NOURISH focused on parenting skills and role-modelling to improve family-based health behaviours. Specific strategies to enhance nutrition were integrated throughout the group-based intervention, including those focused on label reading, reducing sugar-sweetened beverages and increasing fruit and vegetable intake, and portion control	A single group session addressing the roles of diet and exercise in paediatric overweight.	Child fiber and parent protein intake, parent and child dietary intake
	596. Beets 2016 [1050-1053]	The United States of America	Intervention ASPs received a multi-step adaptive framework intervention i.e. Strategies To Enhance Practice for Healthy Eating (STEPs-HE) conceptual framework. Programs had to provide a snack, homework assistance/ completion time, enrichment, and opportunities for physical activity.	The control ASPs did not receive any formal trainings related to snack purchasing during this time	Fruit and vegetable servings, overall energy (kcals) and macronutrient content, micronutrients for foods, dips, beverages, total snack between intervention and control
	597. Berkowitz 2011 [1054]	Philadelphia USA	4 months of an isocaloric meal replacement (MR) approach, followed by consumption of an isocaloric CD from months 5–12 (MR/CD) 4 months of MR with continued use of MR from months 5–12 (MR/MR)	12 months of a 1300–1500 kcal/d conventional diet (CD);	Weight, height, BMI, Serum lipids, glucose, and insulin levels, blood pressure
	598. Blum 2008 [1055]	Denmark	Schools aimed to reduce availability of sugar-sweetened beverages (SSB) and diet soda in food venues for 1 school year	No intervention	Subjects' beverage servings/day
	599. Casazza 2012 [1056]	USA	Reduced carbohydrate diet (diet therapy)	Standard carbohydrate diet	Biochemical and hormonal parameters, change in weight and adiposity

	600. Coleman 2012 [1057]	The United States of America	Intervention goals were to 1) eliminate unhealthy foods and beverages on campus, 2) develop nutrition services as the main source on campus for healthful eating (HE), and 3) promote school staff modeling of HE.	No intervention	Height and weight, BMI Z scores and percentile, BMI values.
	601. De Bock 2012 [1058, 1059]	Germany	Activities involving cooking and eating meals together with children, teachers and parents once weekly for 2 hours	Wait-list control	parent-completed questionnaire assessed fruit and vegetable intakes, water and sugared drinks consumption
	602. De Ruyter 2012 [1060, 1061]	Netherlands	Once daily can of a specialized drink: Participating children received a box at school each week labelled with their name and containing 8 cans, 1 for each day of the week plus 1 extra to be used as a spare in case a can was misplaced.	Sugar group - usual beverage consumption - no intervention	Body weight, height, skinfold thickness (of the biceps, triceps, and subscapular and suprailiac regions), waist circumference, fat mass
	603. Ebbeling 2006 [1062, 1063]	Not specified	Provision of non-caloric beverages and motivational counselling. The target number of individual beverage servings (i.e., 360 mL or 12 fl oz per referent serving) delivered to each home was based on household size	Normal beverage consumption	Height, weight, diet and physical activity recall, adherence to instructions, beverage delivery logistics, overall enjoyment of participation
	604. Ebbeling 2012 [1064]	The United States of America	Home delivery of non-caloric beverages (once in 2 weeks), motivational telephone calls (once in a month), check-in visits	No intervention	Height, weight, body fat, dietary intake, physical activity
	605. Feng 2016 [1065]	The United States of America	School-Based Activities: The multi-component Transformacion Para Salud program: A bilingual school-based curriculum emphasizing knowledge and skills for healthy eating, the Bienestar Health Program, was adapted.	No intervention	Sugar-sweetened beverages (SSBs) intake, BMI Percentile, body Fat %, waist circumference
	606. Ford 2010 [1066]	United Kingdom	A computerised device (Mandometer), dietetic consultations (four in 12 months), advise on improving eating habits and physical activity	Stabdard care	Weight, height, waist circumference, change in percentage body fat/body fat SDS, fasting glucose and insulin concentrations, lipid profile, high sensitivity C reactive protein (HsCRP), blood pressure

	607. French 2012 [1067]	The United States of America	Ounce of prevention (OP): infant centered program. Maternal-focused intervention (MOMS): maternal centered program	Usual care	Maternal height and weight, maternal nutritional intake, maternal eating behavior patterns, maternal depression and affect, child weight and length, maternal food security/hunger, maternal health numeracy, maternal meal planning, child nutritional intake
	608. Gatto 2012 [1068-1072]	The United States of America	Intervention classes and sessions with a 45-minute interactive cooking and nutrition education lesson, interactive gardening lesson	Control participants did not receive any nutrition, gardening, or cooking information between pre and post-testing	dietary fiber and vegetable intake, decreased diastolic blood pressure, BMI, waist circumference, total body fat
	609. Gross 2017 [1073, 1074]	The United States of America	Individual nutrition counseling and nutrition and parenting support groups coordinated with prenatal and pediatric visits	Standard prenatal and paediatric primary care	Infant activity, risk factors for reduced infant activity, family characteristics
	610. Haerens 2007 [1075, 1076]	Belgium	The intervention consisted of an adaptation of the adult computer-tailored dietary intake intervention. Students were exposed once in class to a 50-min theory-based computer-tailored dietary fat intake intervention, which was provided as a self-explanatory CD-ROM	No intervention	Dietary fat intake, use of computer program
	611. Helle 2019 [1077, 1078]	Norway	The intervention consisted of video clips focusing on feeding-related aspects like appropriate food-types and textures, how taste-preferences evolve and responsive feeding practices; and monthly cooking-films and recipes, demonstrating how to make homemade baby- and family food from easily available ingredients.	Routine care	child eating behaviours, dietary intake, mealtime routines and maternal feeding practices and feeding styles
	612. Hoffman 2011 [1079]	The United States of America	The program was based in social learning theory and included school-wide (daily loud speaker announcements), classroom (instructional DVD), lunchroom (daily stickers contingent on a bite of fruit or vegetable), and family (take-home activity books) components to promote F&V	No intervention	Weighed plate waste, F&V preferences, F&V knowledge, BMI, child acceptability of intervention components

			consumption with an emphasis on F&V in the school lunch		
	613. Horton 2013 [1080]	The United States of America	The intervention included the use of a culturally specific telenovela (based on documented effectiveness in past Latino-focused interventions) and accompanying family manual, and was designed to include as many family members as possible. The home visits were designed to include as many family members as possible to foster family support for healthy eating and to maximize sustainability of family behavior change	Delayed treatment- Control families received the DVD series and family manual after completing the final assessment protocol	Fast food consumption, vegetable consumption, monthly variety of fruits and monthly variety of vegetables
	614. Inostroza 2014 [1081]	Chile	Formula milk:<br>Formula (EXPL) with lower protein (1.65 g/100 kcal) and calorie content (62.8 kcal/100 mL) and probiotics.<br>Formula (CTRL) with higher protein (2.70 g/100 kcal) and calorie content (65.6 kcal/100 mL) and no probiotics / breast feeding.	Breast-fed infants	Height, weight, head circumference, BMI, weight for age z-scores, body composition, dietary intake, serum BUN, IGF-1, ghrelin, leptin, insulin, C-peptide
	615. Jamelske 2008 [1082]	The United States of America	Free fruit or vegetable snack every day	No intervention	Participants' preferences regarding fruits and vegetables and frequency of their intake, knowledge about fruits and vegetables, dietary recall (24h, over 3 days)
	616. Kelishadi 2009 [1083]	Iran	Education sessions on nutrition and physical activity (monthly) along with a dairy rich or energy restricted diet recommendation	Education session on nutrition and physical activity (monthly)	Weight, height, percentage of body fat, dietary intake, daily energy intake
	617. Kirk 2012 [1084]	The United States of America	Dietary counselling (LC - low carbohydrate or RGL - reduced glycemic load) and behavioral strategies along with daily vitamin/mineral supplements	Portion controlled diet along with daily vitamin/mineral supplements (standard of care)	Weight, height, waist circumference, BMI, BMI z-score, percent body fat, fasting insulin, glucose, total cholesterol, triglycerides, low-density lipoprotein cholesterol, high-density lipoprotein cholesterol, blood pressure, dietary intake

	618. Kong 2014 [1085]	China	Low GI group (consisting of 45-50% carbohydrate, 30-35% fat and 15-20% protein): The intervention was based on a strategy of increasing energy expenditure and reducing caloric intake using lifestyle behavioral change to achieve long-lasting impact. through dietary sessions (low GI - glycemic index)	Conventional Chinese diet (consisting of 55-60% carbohydrate, 25-30% fat and 10-15% protein)	Daily nutritional intake and food consumption, plasma glucose (hexokinase method), TC (enzymatic method), TG (enzymatic method without glycerol blanking), HDL-C
	619. Lappe 2017 [1086]	United States of America	The dairy group, consumed low-fat (skim, 1%, or 2%) milk or low-fat yogurt servings providing >1200 mg Ca/d, or 2)	Usual diet of <600 mg Ca/d.	Fat mass: BMC, lean mass, waist and hip circumferences, pubertal status, dietary intake, physical activity
	620. Lappe 2004 [1087]	USA	Calcium rich diet supplying at least 1,500 mg of calcium per day	Usual diet	Dietary Intake, Height, weight, and pubertal status
	621. Lent 2014 [1088]	The United States of America	There were three main intervention components:<br>1. the intervention included classroom-based nutrition education lessons on identifying healthy snacks<br>2. a branded social marketing campaign communicated messaging regarding healthy eating and well-being<br>3. corner store-level initiatives included store owner trainings, adding healthier items, and signage identifying healthy items	No intervention	Nutrition information, BMI, BMI z score, and BMI percentile
	622. Machuca 2016 [1089]	The United States of America	Group care model aims to strengthen the foundations of early childhood development, specifically optimal nutrition, responsive parenting, supportive family relationships, and maternal mental health.	Traditional one-on-one care (standard care)	overweight/obesity at age 2 years
	623. Maes 2011 [1090]	Europe	The Food-O-Meter consists of:<br>- A validated FFQ for measuring the dietary intake of adolescents;<br>- A food composition database; and<br>- A decision tree for generating	Generic standard advice in text format	Adolescents' acceptability of the advice, intervention effects at the nutrient level

			individualised advice for enhancing the dietary intakes of fibre, vitamin C, Ca, Fe and fat and for beverages.		
	624. Mendes 2016 [1091]	Brazil	Group A received a fixed diet plan	Group B underwent a calorie counting diet, in which each patient has received a table list with equivalent points for food and drinks, and was instructed to record all daily food or drink intake and calculate the total score of points consumed (1 point = 3.6 calories). They were allowed to eat any food but were limited to the recommended amount of points (or calories equivalents)	Dietary intake, Body composition, Total energy intake (TEI) and macronutrient percentages
	625. Mihas 2010 [1092]	Greece	School-based health education program covering the thematic areas of 'Nutrition–dietary habits' and 'Physical activity and health consisting of workbooks, education sessions, learning activities, posters and displays, meeting with parents	No intervention	Height, weight, BMI, dietary assessment, hours of watching television, screen time
	626. Muckelbauer 2009 [1093-1096]	Germany	The fountains provided cooled, filtered, water. Each child received a plastic water bottle (500 mL), and teachers were encouraged to organize filling of the water bottles each morning for all children in the corresponding classes.<br><br>The educational intervention consisted of classroom lessons dealing with the water needs of the body and the water circuit in nature.	No intervention	Height, weight, water flow, BMI and beverage consumption
	627. Papadaki 2010 [1097, 1098]	Netherlands, Denmark, United Kingdom, Greece, Germany, Spain, Bulgaria, and Czech Republic	low-calorie diet period were randomly assigned to 1 of 5 ad libitum diets:<br><br>low protein (LP)/ low glycemic index (LGI); low protein (LP)/ high glycemic index (HGI);	No intervention	Changes in anthropometric measurements,<br>BMI z score, Body composition, changes in the proportion of overweight and obese children, changes in waist-to-hip circumference ratio.

			high protein (HP)/ low glycemic index (LGI); high protein (HP)/ high glycemic index (HGI)		
	628. Paul 2011 [1099]	The United States of America	Three intervention groups:<br><br>1. ("Soothe/Sleep") instructed parents on discriminating between hunger and other sources of infant distress. Soothing strategies were taught to minimize feeding for non-hunger-related fussiness and to prolong sleep duration, particularly at night.<br><br>2.("Introduction of Solids") taught parents about hunger and satiety cues, the timing for the introduction of solid foods, and how to overcome infants' initial rejection of healthy foods through repeated exposure.<br><br>3. Both the interventions	No intervention	weight-for-length percentile at age 1 year, infant behavioural outcomes, conditional weight gain score, total daily sleep, timing and content of infant feed
	629. Pinket 2016 (ToyBox Study) [1100-1120]	Belgium, Bulgaria, Germany, Greece, Poland and Spain	Intervention to increase water intake and physical activity, decrease sedentary behaviour, and change snacking behaviour	Normal routine	Water intake and beverage consumption, differences in water intake and beverage consumption
	630. Rappaport 2013 [1121]	The United States of America	The School Nutrition Policy Initiative (SNPI) included the following components: (i) school self-assessment; (ii) teacher nutrition education training; (iii) student nutrition education by the trained teachers; (iv) school nutrition policy changes; (v) social marketing and (vi) parent and community outreach.	No intervention	Height, weight, BMI z Score
	631. Rausch Herscovici 2013 [1122]	Argentina	The program focused on increasing the children's knowledge of healthy nutrition and exercise through four workshops; educating the parents/caregivers; and offering healthy options at the school snack bar.	No intervention	Children's intake of healthy and unhealthy foods and BMI

	632. Rosado 2008 [1123]	The United States of America	ready-to-eat cereals (RTEC), 1 serving / 2 servings / 1 serving with nutrition education	No intervention	Anthropometry, body composition, physical activity, blood lipids
	633. Rosario 2013 [1124-1126]	Portugal	The intervention program was based on the Health Promotion Model and the social cognitive theory - nutrition and healthy eating implemented the intervention in the classroom	No intervention	Weight, height, BMI, dietary intake, physical activity
	634. Salazar Vazquez 2016 [1127]	Chile	Recommendations for reducing eating rate without changing diet or meal size according to the educational programme.	No intervention	Weight height, BMI, body surface area (BSA), waist circumference (cm), hip circumference (cm), waist to hip ratio, BMI percentile as normal (BMI<85%), overweight (85%≤BMI<95%), or obese (BMI ≥ 95%**)**
	635. Schroeder 2015 [1128]	The United States of America	Sessions focused on intervention content and delivery. Educational materials on feeding, diet and physical activity	No intervention	Weight, height, child Feeding Practices, dietary Assessment
	636. Schwartz 2016 [1129]	The United States of America	Installation of water jets in schools.	No intervention	BMI, overweight: (≥85th BMI percentile), obese: ≥95th BMI percentile; milk purchases
	637. Sicheri 2008 [1130, 1131]	Brazil	a healthy lifestyle education programme was implemented using simple messages encouraging water consumption instead of sugar-sweetened carbonated beverages	general sessions on health issues and printed general advices regarding healthy diets	Beverage intake measurement, weight variation (BMI, weight, height), overweight and obesity
	638. Struempler 2014 [1132]	The United States of America	Nutrition topics were sequentially taught: trying new foods, food groups, balanced meals, food nutrients, healthy snacks, and extending FV message to others.	No intervention	Weekly fruits and vegetables consumed through School Lunch Program
	639. Truby 2016 [1133-1135]	Australia	Diet counselling along with two diet patterns: the structured modified carbohydrate (SMC) or a structured low fat (SLF)	Wait-list control	Height, weight, waist circumferences, blood pressure, BMI z-score, liver function, plasma lipid profiles, fasting glucose and insulin, adipokines and

					inflammatory markers, diet, activity
	640. Van Horn 2005 (DISC Study) [1136, 1137]	United States of America	Sessions were held to educate the children about food high in saturated fat and their lower fat replacement as well as how to follow the diet advised.	Reading material only	Lipids, lipoproteins, and apolipoprotein measurements, fasting blood glucose, thyroxine, renal and hepatic function tests, height, weight, BMI, tricep/subscapular/suprailiac skinfold measurement, systolic/diastolic blood pressure, dietary intake
	641. Williams 2007 [1138]	Not specified	Diet (1500 kcal/day) which included only one of the two snacks from a healthy snack list (n=19)	Diet (1500 kcal/day) which included both the snacks from a healthy snack list	Physical activity, height, weight, and three skinfold measurements, blood pressure, heart rate, and fasting blood lipids (total cholesterol, high-density lipoprotein cholesterol (HDLC), low-density lipoprotein cholesterol (LDLC), and triglycerides) and glucose
	642. Wong 2017 [1139]	The United States of America	Sessions on nutritional education (6 per month) along with advice on water consumption (8 cups per day)	Sessions on nutritional education (standard care)	Weight, height, waist/hip circumference, participant adherence and satisfaction, diet, physical activity, urine specific gravity
**Diet and Behavioral therapy**	643. Beaulieu 2012 [1140]	Canada	(i) in classrooms (distribution of tools, recipes and pamphlets, audio messages by teachers and school principal, cooking sessions); (ii) at lunch time (improvisation play theatre); (iii) during free time periods (electronic messages, school website, quiz); and (iv) to parents (electronic messages, conference, distribution of tool	general and information technology courses	Psychological and behavioural outcomes regarding diet
	644. Daniels 2012 [1141-1144]	Australia	The intervention was a comprehensive skills-based program that used a cognitive behavioural approach and focused on the feeding and parenting practices that	Usual community child health services	Raw z-scores, change in raw z-scores

			mediate children's early feeding experiences.		
	645. Ermetici 2016 [1145, 1146]	Italy	The "EAT" Project (the Italian acronym for Educazione Alimentare Teenagers—Teenagers Nutritional Education)	Not specified	BMI z score, WHtR, Behavioral habits, Physical activity, Daily walking
	646. Golan 1998 [1147-1150]WAVES	Israel	Parents as sole agent of change (Experimental intervention)	Children responsible for change (Conventional intervention)	Activity level, Eating related to hunger, Eating style, Food intake
	647. Kitzman-Ulrich 2009 [1151]	The United States of America	Intervention 1: multifamily therapy plus psycho-education<br>Intervention 2: psycho-education only	Wait-list control	Height, weight, BMI, dietary intake, family measures
	648. Koo 2018 [1152]	Malaysia	GReat-Child Trial	Not specified	Body weight and height, Percentage of body fat, Waist circumference (WC), Physical activity
	649. The JenMe Program [1153-1155]	Australia	Diet and behavioral therapy	Waiting list for the intervention	No outcomes of interest reported
	650. Moschonis 2019 [1156]	Greece	Computerized decision-support tool (DST)-diet therapy	Generic dietary recommendations	dietary intake, perinatal data, physical activity, BMI, BMI z-score, waist circumference
	651. Mirza 2013 [1157]	USA	Low–glycemic load diet (LGD) along with sessions	Low-fat diet (LFD) along with sessions	BMI z score, changes in insulin resistance and metabolic risk markers
	652. Polonsky 2019 [1158]	USA	Dietary Intervention- breakfast in classroom with breakfast-specific nutrition education	breakfast offered in cafeteria	incidence of overweight and obesity, prevalence of overweight and obesity, BMI z scores, prevalence of obesity
	653. Sharma 2016 [1159]	Houston, USA	Bright Bites	School health program	anthropometric variables, diet intake
	654. Siegel 2011 [1160]	NA	"Healthy Eating Plan" (HEP) with dietitian<br>"Healthy Eating Plan" (HEP) with dietitian and portion control	"Healthy Eating Plan" (HEP) without dietitian	Change in BMI

Figure S1: Impact of obesity prevention interventions on prevalence of overweight

Study or Subgroup	log[Risk Ratio]	SE	Experimental Total	Control Total	Weight	Risk Ratio IV, Random, 95% CI
**1.4.1 Diet only**						
Coleman 2012	0.19	0.1586	208	216	3.0%	1.21 [0.89, 1.65]
Rosário 2013	-0.3168	0.4041	143	151	1.9%	0.73 [0.33, 1.61]
**Subtotal (95% CI)**			**351**	**367**	**4.9%**	**1.08 [0.71, 1.63]**
Heterogeneity: Tau² = 0.03; Chi² = 1.36, df = 1 (P = 0.24); I² = 27%						
Test for overall effect: Z = 0.34 (P = 0.73)						
**1.4.2 Exercise only**						
Farias 2015	-2.2399	0.4598	195	191	1.7%	0.11 [0.04, 0.26]
Hollis 2016	-0.1969	0.1207	49	176	3.2%	0.82 [0.65, 1.04]
Klakk 2013	-0.4588	0.2307	351	281	2.7%	0.63 [0.40, 0.99]
Liu 2007 (1)	-0.0109	0.2755	178	218	2.5%	0.99 [0.58, 1.70]
Liu 2007 (2)	-0.2485	0.2207	150	207	2.8%	0.78 [0.51, 1.20]
Liu 2008 (3)	0.3652	0.314	150	207	2.3%	1.44 [0.78, 2.67]
Liu 2008 (4)	-0.0109	0.2755	178	218	2.5%	0.99 [0.58, 1.70]
Martínez Vizcaíno 2008 (5)	-0.1823	0.3606	37	57	2.1%	0.83 [0.41, 1.69]
Martínez Vizcaíno 2008 (6)	-0.0379	0.3434	36	50	2.2%	0.96 [0.49, 1.89]
Pate 2005	0.0312	0.0705	827	712	3.3%	1.03 [0.90, 1.18]
Sacchetti 2013	0.0187	0.3401	212	216	2.2%	1.02 [0.52, 1.98]
**Subtotal (95% CI)**			**2363**	**2533**	**27.4%**	**0.83 [0.66, 1.04]**
Heterogeneity: Tau² = 0.08; Chi² = 31.09, df = 10 (P = 0.0006); I² = 68%						
Test for overall effect: Z = 1.59 (P = 0.11)						
**1.4.3 Behavioural therapy only**						
Alkon 2014	0.166	0.3196	99	110	2.3%	1.18 [0.63, 2.21]
CHOPPS Study	-0.174353	0.264479	73	71	2.5%	0.84 [0.50, 1.41]
De Villiers 2016	-0.0933	0.5127	391	406	1.5%	0.91 [0.33, 2.49]
De Vries 2015	-0.2123	0.4982	61	37	1.5%	0.81 [0.30, 2.15]
Doring 2016	-0.7	0.0781	36	56	3.3%	0.50 [0.43, 0.58]
Greve 2015	-0.0795	0.1558	377	409	3.1%	0.92 [0.68, 1.25]
Harvey Barino 2003	0	0.751146	20	20	0.9%	1.00 [0.23, 4.36]
Healthy School Start Study II	0.24686	0.245737	96	113	2.6%	1.28 [0.79, 2.07]
HomeStyles study	-0.446287	0.592457	29	28	1.3%	0.64 [0.20, 2.04]
INSIGHT trial	-0.5705	0.3212	116	116	2.3%	0.57 [0.30, 1.06]
Spiegel 2006	-0.1558	0.1399	534	479	3.1%	0.86 [0.65, 1.13]
Warren 2003	-1.0986	1.1339	42	42	0.5%	0.33 [0.04, 3.08]
**Subtotal (95% CI)**			**1874**	**1887**	**24.9%**	**0.80 [0.62, 1.05]**
Heterogeneity: Tau² = 0.11; Chi² = 34.23, df = 11 (P = 0.0003); I² = 68%						
Test for overall effect: Z = 1.63 (P = 0.10)						
**1.4.4 Diet and exercise**						
Aceves-Martins 2017	-0.4804	0.3185	108	167	2.3%	0.62 [0.33, 1.15]
Bayer 2009	1.5267	0.1285	355	932	3.2%	4.60 [3.58, 5.92]
Centis 2012	1.4643	0.1966	98	100	2.9%	4.32 [2.94, 6.36]
Fotu 2011	-0.0873	0.0518	1083	1396	3.4%	0.92 [0.83, 1.01]
Iaia 2017	-0.3196	0.2663	199	133	2.5%	0.73 [0.43, 1.22]
Li 2014	-0.297	0.0922	388	533	3.3%	0.74 [0.62, 0.89]
Llargués 2011	-0.0764	0.1693	225	201	3.0%	0.93 [0.66, 1.29]
Sanigorski 2008	0.103	0.1564	833	974	3.1%	1.11 [0.82, 1.51]
van Nassau 2014	-0.0794	0.1055	1383	705	3.2%	0.92 [0.75, 1.14]
**Subtotal (95% CI)**			**4672**	**5141**	**26.8%**	**1.23 [0.81, 1.86]**
Heterogeneity: Tau² = 0.37; Chi² = 211.76, df = 8 (P < 0.00001); I² = 96%						
Test for overall effect: Z = 0.97 (P = 0.33)						
**1.4.5 Exercise and behavioural therapy**						
Eliakim 2007	-0.1466	0.4161	44	38	1.8%	0.86 [0.38, 1.95]
Melnyk 2013	0.1725	1.41	154	183	0.3%	1.19 [0.07, 18.84]
Speroni 2007	1.328	0.3827	80	105	2.0%	3.77 [1.78, 7.99]
Xu 2017	-0.242	0.2468	251	245	2.6%	0.79 [0.48, 1.27]
**Subtotal (95% CI)**			**529**	**571**	**6.8%**	**1.33 [0.55, 3.21]**
Heterogeneity: Tau² = 0.54; Chi² = 12.51, df = 3 (P = 0.006); I² = 76%						
Test for overall effect: Z = 0.63 (P = 0.53)						
**1.4.7 Diet, exercise and behavioural therapy**						
Greening 2011	0.231112	0.181138	204	246	2.9%	1.26 [0.88, 1.80]
Sharma 2018	-0.1215	0.1554	483	365	3.1%	0.89 [0.65, 1.20]
Taveras 2017	0.2043	0.11	360	361	3.2%	1.23 [0.99, 1.52]
**Subtotal (95% CI)**			**1047**	**972**	**9.2%**	**1.12 [0.90, 1.38]**
Heterogeneity: Tau² = 0.01; Chi² = 3.38, df = 2 (P = 0.18); I² = 41%						
Test for overall effect: Z = 1.02 (P = 0.31)						
**Total (95% CI)**			**10836**	**11471**	**100.0%**	**0.97 [0.83, 1.15]**
Heterogeneity: Tau² = 0.20; Chi² = 353.29, df = 40 (P < 0.00001); I² = 89%						
Test for overall effect: Z = 0.33 (P = 0.74)						
Test for subgroup differences: Chi² = 7.40, df = 5 (P = 0.19), I² = 32.4%						

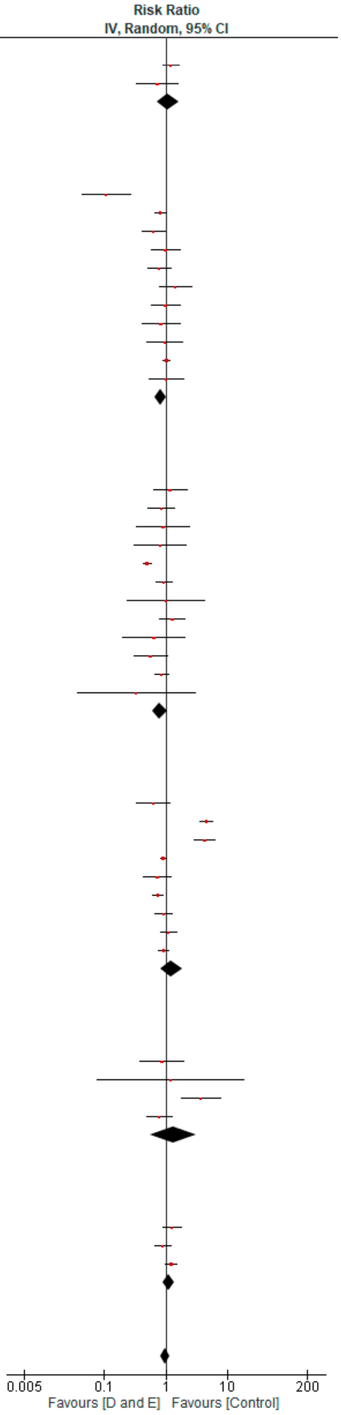

Footnotes
(1) Girls
(2) Boys
(3) Boys
(4) Girls
(5) Girls
(6) Boys

Figure S2: Impact of obesity prevention interventions on prevalence of obesity

Study or Subgroup	log[Risk Ratio]	SE	Weight	Risk Ratio IV, Random, 95% CI
**1.5.1 Diet only**				
Coleman 2012	0.19	0.1586	2.9%	1.21 [0.89, 1.65]
Rosário 2013	0.0409	0.1847	2.7%	1.04 [0.73, 1.50]
**Subtotal (95% CI)**			**5.6%**	**1.14 [0.90, 1.44]**
Heterogeneity: Tau² = 0.00; Chi² = 0.38, df = 1 (P = 0.54); I² = 0%				
Test for overall effect: Z = 1.05 (P = 0.29)				
**1.5.2 Exercise only**				
Farias 2015	-2.2399	0.4598	1.5%	0.11 [0.04, 0.26]
Hollis 2016	0.1582	0.233	2.5%	1.17 [0.74, 1.85]
Klakk 2013	-0.4588	0.2307	2.5%	0.63 [0.40, 0.99]
Liu 2007	0.3652	0.314	2.1%	1.44 [0.78, 2.67]
Liu 2007 (1)	-0.1978	0.2469	2.4%	0.82 [0.51, 1.33]
Liu 2008 (2)	-0.2485	0.2207	2.6%	0.78 [0.51, 1.20]
Liu 2008 (3)	-0.1978	0.2469	2.4%	0.82 [0.51, 1.33]
Martínez Vizcaíno 2008 (4)	-0.1823	0.3606	1.9%	0.83 [0.41, 1.69]
Martínez Vizcaíno 2008 (5)	-0.0379	0.3434	2.0%	0.96 [0.49, 1.89]
Sacchetti 2013	-0.0554	0.1666	2.8%	0.95 [0.68, 1.31]
**Subtotal (95% CI)**			**22.8%**	**0.79 [0.60, 1.05]**
Heterogeneity: Tau² = 0.13; Chi² = 27.49, df = 9 (P = 0.001); I² = 67%				
Test for overall effect: Z = 1.61 (P = 0.11)				
**1.5.3 Behavioural therapy only**				
Alkon 2014	0.1054	0.2843	2.3%	1.11 [0.64, 1.94]
Barkin 2018	0.0378	0.1169	3.0%	1.04 [0.83, 1.31]
De Villiers 2016	-0.3081	0.3703	1.9%	0.73 [0.36, 1.52]
Doring 2016	-0.7	0.0781	3.1%	0.50 [0.43, 0.58]
French 2018	-0.0938	0.137	2.9%	0.91 [0.70, 1.19]
Greve 2015	-0.1417	0.3807	1.8%	0.87 [0.41, 1.83]
Harvey Barino 2003	-1.771957	1.056942	0.5%	0.17 [0.02, 1.35]
Healthy School Start Study II	0.24686	0.395437	1.8%	1.28 [0.59, 2.78]
INSIGHT trial	-1.0986	0.6536	1.0%	0.33 [0.09, 1.20]
Spiegel 2006	-0.1285	0.1259	3.0%	0.88 [0.69, 1.13]
Warren 2003	1.0986	1.6187	0.2%	3.00 [0.13, 71.61]
**Subtotal (95% CI)**			**21.5%**	**0.81 [0.61, 1.08]**
Heterogeneity: Tau² = 0.13; Chi² = 46.62, df = 10 (P < 0.00001); I² = 79%				
Test for overall effect: Z = 1.43 (P = 0.15)				
**1.5.4 Exercise and behavioural therapy**				
Kain 2009 (6)	-0.3852	0.1914	2.7%	0.68 [0.47, 0.99]
Kain 2009 (7)	-0.5488	0.142	2.9%	0.58 [0.44, 0.76]
Speroni 2007	1.3705	0.4104	1.7%	3.94 [1.76, 8.80]
WAVES study	0.0697	0.1319	3.0%	1.07 [0.83, 1.39]
Xu 2017	0.0086	0.2398	2.5%	1.01 [0.63, 1.61]
**Subtotal (95% CI)**			**12.8%**	**1.00 [0.65, 1.54]**
Heterogeneity: Tau² = 0.20; Chi² = 26.32, df = 4 (P < 0.0001); I² = 85%				
Test for overall effect: Z = 0.02 (P = 0.99)				
**1.5.6 Diet and exercise**				
Aceves-Martins 2017	-0.6627	0.6551	1.0%	0.52 [0.14, 1.86]
Ariza 2019	0.2265	0.1119	3.0%	1.25 [1.01, 1.56]
Bayer 2009	2.3133	0.2431	2.5%	10.11 [6.28, 16.28]
Centis 2012	2.7823	0.3962	1.8%	16.16 [7.43, 35.12]
de Niet 2012	0.1049	0.2057	2.6%	1.11 [0.74, 1.66]
Fotu 2011	0.0608	0.0825	3.1%	1.06 [0.90, 1.25]
Iaia 2017	-1.0961	0.6982	0.9%	0.33 [0.09, 1.31]
Li 2014	-0.297	0.0922	3.1%	0.74 [0.62, 0.89]
Llargués 2011	0.238	0.3407	2.0%	1.27 [0.65, 2.47]
Sanigorski 2008	0.056	0.0914	3.1%	1.06 [0.88, 1.27]
van Nassau 2014	0.5048	0.2821	2.3%	1.66 [0.95, 2.88]
Waters 2018	0.1005	0.1002	3.1%	1.11 [0.91, 1.35]
**Subtotal (95% CI)**			**28.5%**	**1.50 [1.07, 2.09]**
Heterogeneity: Tau² = 0.27; Chi² = 155.38, df = 11 (P < 0.00001); I² = 93%				
Test for overall effect: Z = 2.38 (P = 0.02)				
**1.5.7 Diet, exercise and behavioural therapy**				
Greening 2011	-0.174353	0.13188	3.0%	0.84 [0.65, 1.09]
Sharma 2018	-0.0098	0.16	2.9%	0.99 [0.72, 1.35]
Taveras 2017	0.2831	0.108	3.1%	1.33 [1.07, 1.64]
**Subtotal (95% CI)**			**8.9%**	**1.04 [0.78, 1.40]**
Heterogeneity: Tau² = 0.05; Chi² = 7.57, df = 2 (P = 0.02); I² = 74%				
Test for overall effect: Z = 0.29 (P = 0.77)				
**Total (95% CI)**			**100.0%**	**1.02 [0.88, 1.19]**
Heterogeneity: Tau² = 0.18; Chi² = 321.29, df = 42 (P < 0.00001); I² = 87%				
Test for overall effect: Z = 0.26 (P = 0.79)				
Test for subgroup differences: Chi² = 11.45, df = 5 (P = 0.04), I² = 56.3%				

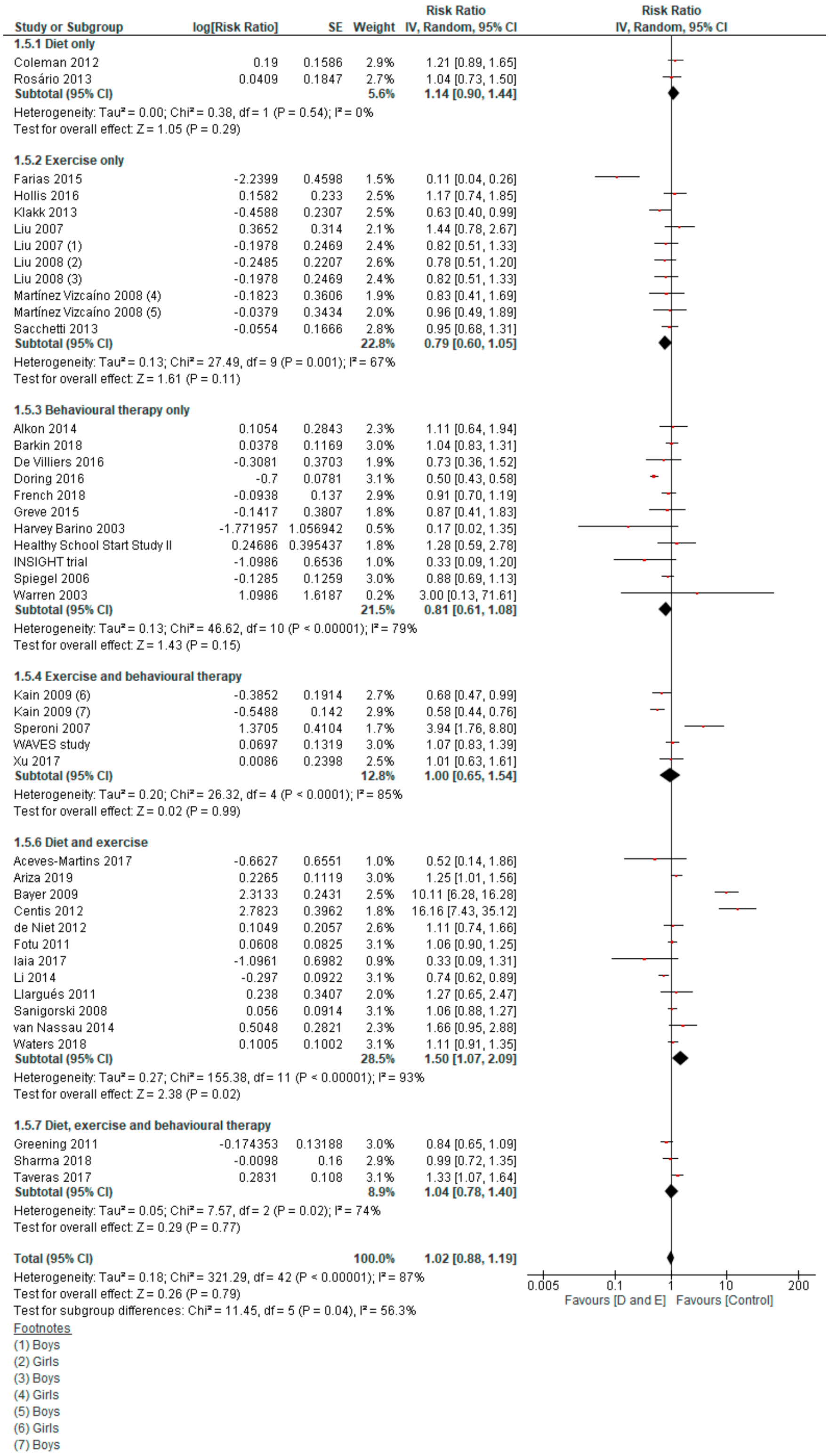

Footnotes
(1) Boys
(2) Girls
(3) Boys
(4) Girls
(5) Boys
(6) Girls
(7) Boys

Figure S3: Impact of obesity prevention interventions on percentage body fat change

Study or Subgroup	Mean Difference	SE	Experimental Total	Control Total	Weight	Mean Difference IV, Random, 95% CI
**1.6.1 Diet only**						
Feng 2016	0.06	1.1278	179	135	2.3%	0.06 [-2.15, 2.27]
Gatto 2012	-0.8	2.4572	34	70	0.7%	-0.80 [-5.62, 4.02]
Lappe 2004	-0.7	1.1092	27	32	2.3%	-0.70 [-2.87, 1.47]
Vandongen 1995 (1)	0.1	1.2795	15	20	1.9%	0.10 [-2.41, 2.61]
Vandongen 1995 (2)	2.4	1.0795	17	17	2.4%	2.40 [0.28, 4.52]
**Subtotal (95% CI)**			**272**	**274**	**9.7%**	**0.43 [-0.78, 1.64]**
Heterogeneity: Tau² = 0.32; Chi² = 4.79, df = 4 (P = 0.31); I² = 17%						
Test for overall effect: Z = 0.70 (P = 0.49)						
**1.6.2 Exercise only**						
Adamo 2017	1.5	1.3901	44	26	1.7%	1.50 [-1.22, 4.22]
Aggeloussi 2012	-6	2.3849	13	11	0.7%	-6.00 [-10.67, -1.33]
Lee 2010	-4.47	1.7607	11	7	1.2%	-4.47 [-7.92, -1.02]
Martínez Vizcaíno 2008 (3)	-0.6	1.4743	36	50	1.6%	-0.60 [-3.49, 2.29]
Martínez Vizcaíno 2008 (4)	-0.7	1.2644	37	57	2.0%	-0.70 [-3.18, 1.78]
Martínez-Vizcaíno 2014 (5)	-0.4	1.0431	62	66	2.5%	-0.40 [-2.44, 1.64]
Martínez-Vizcaíno 2014 (6)	-0.1	1.2099	56	74	2.1%	-0.10 [-2.47, 2.27]
MOPO study	2.2	4.1	250	246	0.3%	2.20 [-5.84, 10.24]
Scheffler 2007 (7)	0.92	0.9835	27	30	2.7%	0.92 [-1.01, 2.85]
Scheffler 2007 (8)	0.42	1.1275	38	32	2.3%	0.42 [-1.79, 2.63]
Silva 2015	3.2	2.8144	17	19	0.5%	3.20 [-2.32, 8.72]
Smith 2016	-2.2	0.9114	139	154	2.9%	-2.20 [-3.99, -0.41]
Vandongen 1995 (9)	0.8	1.186	20	20	2.1%	0.80 [-1.52, 3.12]
Vandongen 1995 (10)	2.4	1.0795	19	17	2.4%	2.40 [0.28, 4.52]
Velez 2010	-1.6	3.5295	13	15	0.4%	-1.60 [-8.52, 5.32]
**Subtotal (95% CI)**			**782**	**824**	**25.4%**	**-0.22 [-1.24, 0.80]**
Heterogeneity: Tau² = 1.85; Chi² = 28.42, df = 14 (P = 0.01); I² = 51%						
Test for overall effect: Z = 0.42 (P = 0.68)						
**1.6.3 Behavioural therapy only**						
Black 2010	1.56	1.65403	91	93	1.3%	1.56 [-1.68, 4.80]
Cretan Health and Nutrition Education programme	0.1	0.028283	331	303	6.0%	0.10 [0.04, 0.16]
De Vries 2015	-1.1	0.8442	61	37	3.1%	-1.10 [-2.75, 0.55]
Guelph Family Health Study	0.14	1.866878	16	12	1.1%	0.14 [-3.52, 3.80]
**Subtotal (95% CI)**			**499**	**445**	**11.6%**	**0.10 [0.04, 0.15]**
Heterogeneity: Tau² = 0.00; Chi² = 2.80, df = 3 (P = 0.42); I² = 0%						
Test for overall effect: Z = 3.51 (P = 0.0005)						
**1.6.4 Exercise and behavioural therapy**						
CHIRPY DRAGON	-0.99	0.4437	340	308	4.8%	-0.99 [-1.86, -0.12]
Eliakim 2007	-0.7	1.2719	44	38	2.0%	-0.70 [-3.19, 1.79]
Hrafnkelsson 2014	-0.9	1.760962	30	23	1.2%	-0.90 [-4.35, 2.55]
WAVES study	0.92	0.3787	556	641	5.1%	0.92 [0.18, 1.66]
Xu 2017	-0.6	0.6196	251	245	4.0%	-0.60 [-1.81, 0.61]
**Subtotal (95% CI)**			**1221**	**1255**	**17.1%**	**-0.29 [-1.33, 0.75]**
Heterogeneity: Tau² = 0.80; Chi² = 12.33, df = 4 (P = 0.02); I² = 68%						
Test for overall effect: Z = 0.55 (P = 0.58)						
**1.6.5 Diet and exercise**						
Davis 2009	-2	6.8898	15	7	0.1%	-2.00 [-15.50, 11.50]
De Henauw 2015	-0.444	0.3644	10392	4768	5.2%	-0.44 [-1.16, 0.27]
Dewar 2013	-0.83	1.2504	113	121	2.0%	-0.83 [-3.28, 1.62]
Donnelly 1996	1.5	1.7548	64	44	1.2%	1.50 [-1.94, 4.94]
Elder 2014	-1.3	0.9045	237	252	2.9%	-1.30 [-3.07, 0.47]
Fotu 2011	-1.1	0.585	815	897	4.2%	-1.10 [-2.25, 0.05]
Johnston 2010	-1.94	0.6987	46	25	3.7%	-1.94 [-3.31, -0.57]
Puder 2011	-0.9	0.5188	333	292	4.5%	-0.90 [-1.92, 0.12]
Story 2012	1.81	2.2286	267	187	0.8%	1.81 [-2.56, 6.18]
Yang 2017	-1.1	0.2525	418	350	5.6%	-1.10 [-1.59, -0.61]
**Subtotal (95% CI)**			**12700**	**6943**	**30.2%**	**-0.95 [-1.28, -0.61]**
Heterogeneity: Tau² = 0.00; Chi² = 8.03, df = 9 (P = 0.53); I² = 0%						
Test for overall effect: Z = 5.54 (P < 0.00001)						
**1.6.6 Diet, exercise and behavioral therapy**						
Rosenbaum 2007	-4.3	0.6636	49	24	3.9%	-4.30 [-5.60, -3.00]
Weber 2017 (11)	2.7	1.9809	34	61	1.0%	2.70 [-1.18, 6.58]
Weber 2017 (12)	-2.6	1.7976	36	64	1.2%	-2.60 [-6.12, 0.92]
**Subtotal (95% CI)**			**119**	**149**	**6.0%**	**-1.71 [-5.66, 2.25]**
Heterogeneity: Tau² = 9.90; Chi² = 11.50, df = 2 (P = 0.003); I² = 83%						
Test for overall effect: Z = 0.85 (P = 0.40)						
**Total (95% CI)**			**15593**	**9890**	**100.0%**	**-0.50 [-0.92, -0.07]**
Heterogeneity: Tau² = 0.77; Chi² = 141.16, df = 41 (P < 0.00001); I² = 71%						
Test for overall effect: Z = 2.30 (P = 0.02)						
Test for subgroup differences: Chi² = 38.33, df = 5 (P < 0.00001), I² = 87.0%						

Mean Difference IV, Random, 95% CI

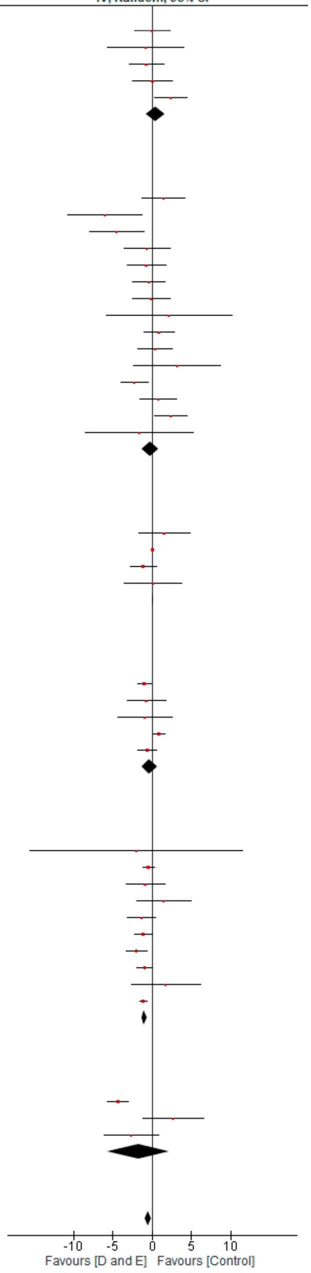

Footnotes
(1) Boys
(2) Girls
(3) Boys
(4) Girls
(5) Girls
(6) Boys
(7) Boys
(8) Girls
(9) Boys
(10) Girls
(11) Females
(12) Males

Figure S4: Impact of obesity prevention interventions on skinfold thickness (Tricep)

Study or Subgroup	Mean Difference	SE	Experimental Total	Control Total	Weight	Mean Difference IV, Random, 95% CI
**1.7.1 Diet only**						
de Ruyter 2012	2.3	0.75	322	319	3.7%	2.30 [0.83, 3.77]
Schroeder 2015	0.62	1.01	112	110	2.9%	0.62 [-1.36, 2.60]
Vandongen 1995 (1)	0.6	1.1233	17	17	2.6%	0.60 [-1.60, 2.80]
Vandongen 1995 (2)	0.3	1.1148	15	20	2.6%	0.30 [-1.88, 2.48]
**Subtotal (95% CI)**			**466**	**466**	**11.8%**	**1.20 [0.19, 2.21]**
Heterogeneity: Tau² = 0.13; Chi² = 3.41, df = 3 (P = 0.33); I² = 12%						
Test for overall effect: Z = 2.32 (P = 0.02)						
**1.7.2 Exercise only**						
Lee 2010	-2.74	1.9704	11	7	1.2%	-2.74 [-6.60, 1.12]
Martínez Vizcaíno 2008 (3)	-0.5	1.5849	36	50	1.7%	-0.50 [-3.61, 2.61]
Martínez Vizcaíno 2008 (4)	0.1	1.4801	37	57	1.9%	0.10 [-2.80, 3.00]
Robinson 1999	-0.99	2.3522	95	103	0.9%	-0.99 [-5.60, 3.62]
Scheffler 2007 (5)	-0.16	0.6746	38	32	3.9%	-0.16 [-1.48, 1.16]
Scheffler 2007 (6)	-0.59	0.5671	27	30	4.3%	-0.59 [-1.70, 0.52]
Vandongen 1995 (7)	0.8	1.0595	20	20	2.8%	0.80 [-1.28, 2.88]
Vandongen 1995 (8)	2	1.0285	19	17	2.8%	2.00 [-0.02, 4.02]
**Subtotal (95% CI)**			**283**	**316**	**19.5%**	**-0.04 [-0.78, 0.69]**
Heterogeneity: Tau² = 0.10; Chi² = 7.66, df = 7 (P = 0.36); I² = 9%						
Test for overall effect: Z = 0.12 (P = 0.91)						
**1.7.3 Behavioural therapy only**						
da Silva 2019	0.41	0.606085	286	315	4.1%	0.41 [-0.78, 1.60]
De Vries 2015	-0.7	0.4015	61	37	4.8%	-0.70 [-1.49, 0.09]
Dennison 2004	-0.41	1.5867	81	66	1.7%	-0.41 [-3.52, 2.70]
French 2018	0	0.491	235	258	4.5%	0.00 [-0.96, 0.96]
KOPS Study 2007	-0.6	3.1787	345	1419	0.6%	-0.60 [-6.83, 5.63]
Memphis Girls health Enrichment Multi-site (GEMS)	-0.1	1.2897	116	127	2.2%	-0.10 [-2.63, 2.43]
Thakur 2016	-3.64	0.7223	94	122	3.7%	-3.64 [-5.06, -2.22]
**Subtotal (95% CI)**			**1218**	**2344**	**21.6%**	**-0.77 [-1.88, 0.35]**
Heterogeneity: Tau² = 1.36; Chi² = 22.17, df = 6 (P = 0.001); I² = 73%						
Test for overall effect: Z = 1.34 (P = 0.18)						
**1.7.4 Diet and exercise**						
Centis 2012	-1.8	0.7399	98	100	3.7%	-1.80 [-3.25, -0.35]
Kain 2004 (9)	-1.4	0.28234	899	407	5.1%	-1.40 [-1.95, -0.85]
Kain 2004 (10)	-1.4	0.3025	860	264	5.0%	-1.40 [-1.99, -0.81]
Li 2014	-2.47	0.49	388	532	4.5%	-2.47 [-3.43, -1.51]
Mo-suwan 1998	-0.1	0.7338	147	145	3.7%	-0.10 [-1.54, 1.34]
Singhal 2010	-0.14	1.12	99	102	2.6%	-0.14 [-2.34, 2.06]
Stettler 2015	-9.4	5.4633	97	11	0.2%	-9.40 [-20.11, 1.31]
Story 2012	0.41	0.1004	267	187	5.4%	0.41 [0.21, 0.61]
van Nassau 2014	1.1	2.3307	1383	705	1.0%	1.10 [-3.47, 5.67]
**Subtotal (95% CI)**			**4238**	**2453**	**31.2%**	**-1.00 [-2.02, 0.01]**
Heterogeneity: Tau² = 1.63; Chi² = 96.94, df = 8 (P < 0.00001); I² = 92%						
Test for overall effect: Z = 1.94 (P = 0.05)						
**1.7.5 Exercise and behavioural therapy**						
Amini 2016	-1.12	0.7071	130	129	3.8%	-1.12 [-2.51, 0.27]
Kain 2009 (11)	-1.4	0.4778	717	225	4.5%	-1.40 [-2.34, -0.46]
Kain 2009 (12)	-1.4	0.4338	749	348	4.7%	-1.40 [-2.25, -0.55]
Magnusson 2012	-2.1	4.6546	29	25	0.3%	-2.10 [-11.22, 7.02]
Simons 2015	5.3	3.475695	131	126	0.5%	5.30 [-1.51, 12.11]
Singh 2007/2009 (13)	-1.8	3.453	89	77	0.5%	-1.80 [-8.57, 4.97]
Singh 2007/2009 (14)	-4.5	4.1226	102	67	0.3%	-4.50 [-12.58, 3.58]
**Subtotal (95% CI)**			**1947**	**997**	**14.6%**	**-1.33 [-1.89, -0.76]**
Heterogeneity: Tau² = 0.00; Chi² = 4.41, df = 6 (P = 0.62); I² = 0%						
Test for overall effect: Z = 4.59 (P < 0.00001)						
**1.7.6 Diet, exercise and behavioural therapy**						
Gerards 2015	0.45	1.9	35	32	1.3%	0.45 [-3.27, 4.17]
**Subtotal (95% CI)**			**35**	**32**	**1.3%**	**0.45 [-3.27, 4.17]**
Heterogeneity: Not applicable						
Test for overall effect: Z = 0.24 (P = 0.81)						
**Total (95% CI)**			**8187**	**6608**	**100.0%**	**-0.53 [-1.02, -0.04]**
Heterogeneity: Tau² = 1.15; Chi² = 165.21, df = 35 (P < 0.00001); I² = 79%						
Test for overall effect: Z = 2.10 (P = 0.04)						
Test for subgroup differences: Chi² = 21.67, df = 5 (P = 0.0006), I² = 76.9%						

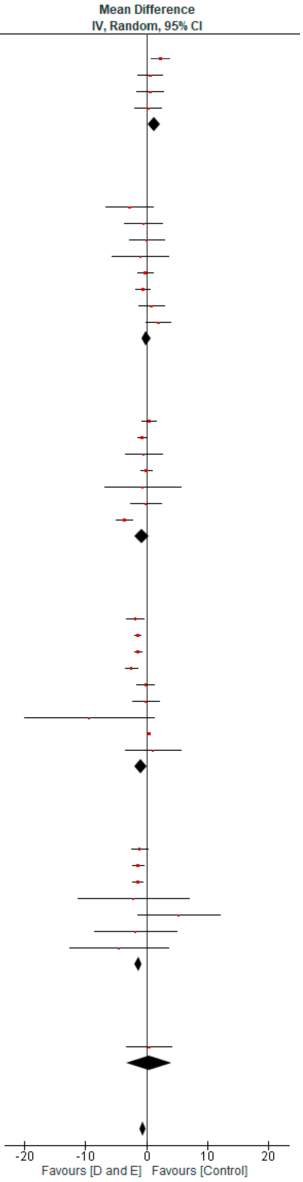

Footnotes
(1) Girls
(2) Boys
(3) Boys
(4) Girls
(5) Girls
(6) Boys
(7) Boys
(8) Girls
(9) BOYS
(10) Girls
(11) Girls
(12) Boys
(13) Boys
(14) Girls

Figure S5: Impact of obesity prevention interventions on waist circumference

Study or Subgroup	Mean Difference	SE	Experimental Total	Control Total	Weight	Mean Difference IV, Random, 95% CI
**1.8.1 Diet only**						
de Ruyter 2012	0.66	0.24	322	319	2.3%	0.66 [0.19, 1.13]
Feng 2016	-1.26	1.4041	176	134	1.8%	-1.26 [-4.01, 1.49]
Gatto 2012	-2.4	2.8636	34	70	1.0%	-2.40 [-8.01, 3.21]
Papadaki 2010	70.9	1.1	96	21	2.0%	70.90 [68.74, 73.06]
**Subtotal (95% CI)**			**628**	**544**	**7.0%**	**17.00 [-19.99, 53.98]**
Heterogeneity: Tau^2 = 1421.66; Chi^2 = 3909.55, df = 3 (P < 0.00001); I^2 = 100%						
Test for overall effect: Z = 0.90 (P = 0.37)						
**1.8.2 Exercise only**						
Martínez-Vizcaíno 2014 (1)	-1.2	1.684	56	74	1.6%	-1.20 [-4.50, 2.10]
Martínez-Vizcaíno 2014	-1.1	1.5469	62	66	1.7%	-1.10 [-4.13, 1.93]
Minnesota GEMS	1.4	0.8	26	27	2.1%	1.40 [-0.17, 2.97]
Nogueira 2014	3.1	1.655	71	67	1.6%	3.10 [-0.14, 6.34]
Robinson 1999	-1.16	3.7301	95	103	0.7%	-1.16 [-8.47, 6.15]
Silva 2015	0.3	2.7347	17	19	1.1%	0.30 [-5.06, 5.66]
Smith 2016	0	1.3255	125	141	1.8%	0.00 [-2.60, 2.60]
Thivel 2011 (2)	2.43	3.3632	60	41	0.8%	2.43 [-4.16, 9.02]
**Subtotal (95% CI)**			**512**	**538**	**11.4%**	**0.73 [-0.31, 1.77]**
Heterogeneity: Tau^2 = 0.00; Chi^2 = 6.31, df = 7 (P = 0.50); I^2 = 0%						
Test for overall effect: Z = 1.38 (P = 0.17)						
**1.8.3 Behavioural therapy only**						
Being Active Eating Well initiative (3)	-0.38	0.8645	351	681	2.1%	-0.38 [-2.07, 1.31]
Being Active Eating Well initiative (4)	1.15	0.7471	1028	904	2.1%	1.15 [-0.31, 2.61]
CHOPPS Study	-0.16	0.1914	73	71	2.3%	-0.16 [-0.54, 0.22]
Cretan Health and Nutrition Education programme	-2.2	0.047705	331	303	2.3%	-2.20 [-2.29, -2.11]
da Silva 2019	0.67	0.787048	286	314	2.1%	0.67 [-0.87, 2.21]
De Vries 2015	-0.4	0.5604	61	37	2.2%	-0.40 [-1.50, 0.70]
Doring 2016	-0.5	1.1792	36	56	1.9%	-0.50 [-2.81, 1.81]
French 2018	0.3	0.7551	235	258	2.1%	0.30 [-1.18, 1.78]
Gong 2014	-1.6	1.113386	160	166	1.9%	-1.60 [-3.78, 0.58]
KOPS Study 2007	0.4	3.2361	345	1419	0.9%	0.40 [-5.94, 6.74]
Memphis Girls health Enrichment Multi-site (GEMS)	-0.1	0.8546	116	127	2.1%	-0.10 [-1.77, 1.57]
Thakur 2016	-5.69	1.5058	94	122	1.7%	-5.69 [-8.64, -2.74]
Wadolowska 2019	0.36	0.0978	319	145	2.3%	0.36 [0.17, 0.55]
**Subtotal (95% CI)**			**3435**	**4603**	**26.3%**	**-0.54 [-1.57, 0.49]**
Heterogeneity: Tau^2 = 2.78; Chi^2 = 662.91, df = 12 (P < 0.00001); I^2 = 98%						
Test for overall effect: Z = 1.03 (P = 0.30)						
**1.8.4 Diet and exercise**						
Bonis 2014	0.3	0.9716	110	99	2.0%	0.30 [-1.60, 2.20]
Centis 2012	-3.2	1.2366	98	100	1.9%	-3.20 [-5.62, -0.78]
Elder 2014	-1.19	0.5646	271	269	2.2%	-1.19 [-2.30, -0.08]
Grydeland 2013	0.2	0.21	465	859	2.3%	0.20 [-0.21, 0.61]
Li 2014	-4.23	0.79	388	533	2.1%	-4.23 [-5.78, -2.68]
Nguyen 2012	-0.5	1.9761	73	78	1.4%	-0.50 [-4.37, 3.37]
Novotny 2015	-0.4	1.9093	41	44	1.5%	-0.40 [-4.14, 3.34]
Sanigorski 2008	3	0.5257	833	974	2.2%	3.00 [1.97, 4.03]
Santos 2014 (5)	-1.5	0.61	158	156	2.2%	-1.50 [-2.70, -0.30]
Santos 2014 (6)	-1.5	0.73	182	151	2.2%	-1.50 [-2.93, -0.07]
Singhal 2010	-1.3	0.57	99	102	2.2%	-1.30 [-2.42, -0.18]
Small 2014	0.57	0.94	33	27	2.0%	0.57 [-1.27, 2.41]
Waters 2018	-0.23	1.1156	1326	1437	1.9%	-0.23 [-2.42, 1.96]
Zask 2012	-0.6	0.0411	114	99	2.3%	-0.60 [-0.68, -0.52]
**Subtotal (95% CI)**			**4191**	**4928**	**28.6%**	**-0.67 [-1.34, 0.00]**
Heterogeneity: Tau^2 = 1.00; Chi^2 = 95.25, df = 13 (P < 0.00001); I^2 = 86%						
Test for overall effect: Z = 1.95 (P = 0.05)						
**1.8.5 Diet and behavioural therapy**						
Daniels 2012	0.9	1.6102	132	137	1.6%	0.90 [-2.26, 4.06]
**Subtotal (95% CI)**			**132**	**137**	**1.6%**	**0.90 [-2.26, 4.06]**
Heterogeneity: Not applicable						
Test for overall effect: Z = 0.56 (P = 0.58)						
**1.8.6 Exercise and behavioural therapy**						
Amini 2016	-0.69	0.9042	130	129	2.1%	-0.69 [-2.46, 1.08]
CHIRPY DRAGON	-0.4	0.4068	575	558	2.3%	-0.40 [-1.20, 0.40]
Kain 2009 (7)	0.4	0.6954	717	225	2.2%	0.40 [-0.96, 1.76]
Kain 2009 (8)	-0.5	0.5977	749	348	2.2%	-0.50 [-1.67, 0.67]
Magnusson 2012	0.3	1.667	29	25	1.6%	0.30 [-2.97, 3.57]
Siegrist 2011	-0.4	0.883354	226	158	2.1%	-0.40 [-2.13, 1.33]
Simons 2015	0.26	0.126642	131	126	2.3%	0.26 [0.01, 0.51]
Singh 2007/2009 (9)	-0.9	1.2252	89	77	1.9%	-0.90 [-3.30, 1.50]
Singh 2007/2009 (10)	-2.3	1.2274	102	67	1.9%	-2.30 [-4.71, 0.11]
Xu 2017	-0.1	0.8982	251	245	2.1%	-0.10 [-1.86, 1.66]
**Subtotal (95% CI)**			**2999**	**1958**	**20.6%**	**-0.01 [-0.34, 0.31]**
Heterogeneity: Tau^2 = 0.03; Chi^2 = 9.89, df = 9 (P = 0.36); I^2 = 9%						
Test for overall effect: Z = 0.07 (P = 0.94)						
**1.8.7 Diet, exercise and behavioural therapy**						
Baranowski 2003	2.4	0.3451	17	14	2.3%	2.40 [1.72, 3.08]
Gerards 2015	0.44	0.79	35	32	2.1%	0.44 [-1.11, 1.99]
**Subtotal (95% CI)**			**52**	**46**	**4.4%**	**1.55 [-0.36, 3.45]**
Heterogeneity: Tau^2 = 1.55; Chi^2 = 5.17, df = 1 (P = 0.02); I^2 = 81%						
Test for overall effect: Z = 1.59 (P = 0.11)						
**Total (95% CI)**			**11949**	**12754**	**100.0%**	**1.04 [0.30, 1.78]**
Heterogeneity: Tau^2 = 6.12; Chi^2 = 5621.04, df = 51 (P < 0.00001); I^2 = 99%						
Test for overall effect: Z = 2.74 (P = 0.006)						
Test for subgroup differences: Chi^2 = 10.24, df = 6 (P = 0.12), I^2 = 41.4%						

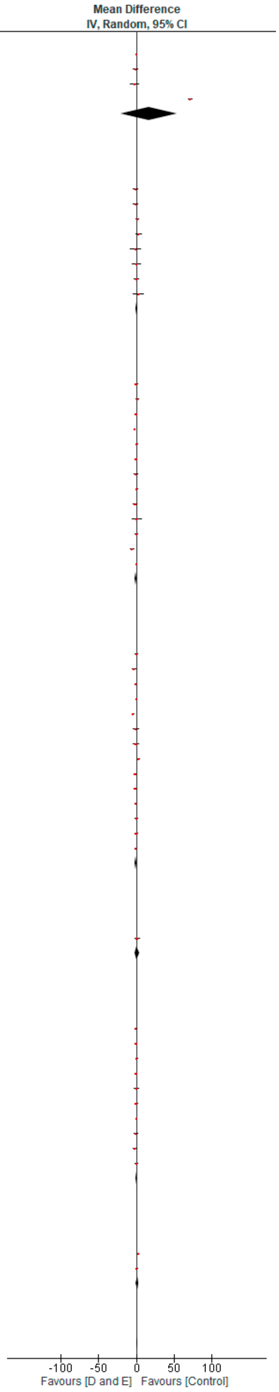

Footnotes
(1) Boys
(2) O
(3) Secondary School (in community 3 and 4)
(4) Primary School (in community 1 and 2)
(5) All younger students (6- to 8-year-old)
(6) All older students (9 to 12 years old)
(7) Girls
(8) Boys
(9) Boys
(10) Girls

Figure S6: Impact of obesity prevention interventions on health-related quality of life

Study or Subgroup	Mean Difference	SE	Weight	Mean Difference IV, Random, 95% CI
**1.9.1 Diet and exercise**				
Waters 2018	1.2	1.6722	100.0%	1.20 [-2.08, 4.48]
**Subtotal (95% CI)**			**100.0%**	**1.20 [-2.08, 4.48]**
Heterogeneity: Not applicable				
Test for overall effect: Z = 0.72 (P = 0.47)				
**Total (95% CI)**			**100.0%**	**1.20 [-2.08, 4.48]**
Heterogeneity: Not applicable				
Test for overall effect: Z = 0.72 (P = 0.47)				
Test for subgroup differences: Not applicable				

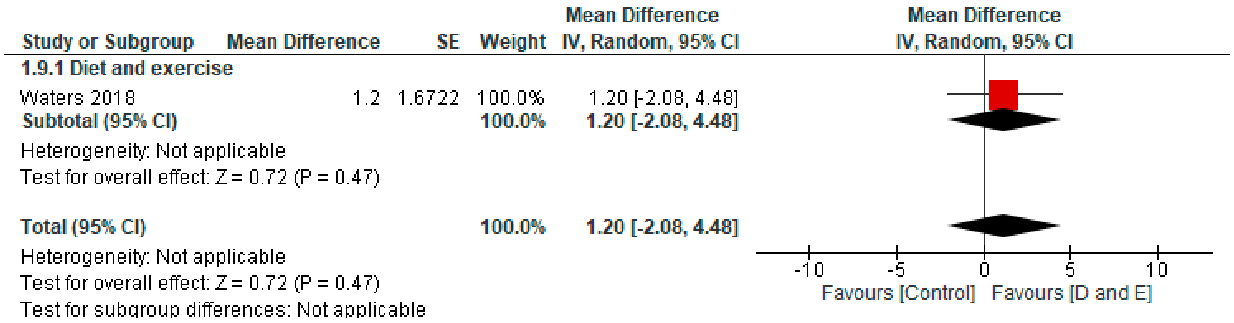

Figure S7: Impact of obesity prevention interventions on physical activity intensity

Study or Subgroup	Std. Mean Difference	SE	Experimental Total	Control Total	Weight	Std. Mean Difference IV, Random, 95% CI
**1.10.1 Exercise only**						
Vandongen 1995 (1)	-1.2	0.2963	19	17	2.5%	-1.20 [-1.78, -0.62]
Graves 2010	-0.8	1.0185	22	20	2.4%	-0.80 [-2.80, 1.20]
Vandongen 1995 (2)	-0.4	0.1453	20	20	2.5%	-0.40 [-0.68, -0.12]
Carlin 2018	-0.343672	0.214285	41	48	2.5%	-0.34 [-0.76, 0.08]
Jago 2019	-0.047731	0.182903	54	67	2.5%	-0.05 [-0.41, 0.31]
Benjamin 2015 (3)	1.3	0.535	34	18	2.5%	1.30 [0.25, 2.35]
Minnesota GEMS	2.9	13.7	26	27	0.2%	2.90 [-23.95, 29.75]
Aggeloussi 2012	9.9	0.8221	13	11	2.4%	9.90 [8.29, 11.51]
**Subtotal (95% CI)**			**229**	**228**	**17.5%**	**0.98 [-0.12, 2.09]**
Heterogeneity: Tau² = 1.98; Chi² = 173.62, df = 7 (P < 0.00001); I² = 96%						
Test for overall effect: Z = 1.74 (P = 0.08)						
**1.10.2 Behavioural therapy only**						
Early 2019	-11	19.3326	15	20	0.1%	-11.00 [-48.89, 26.89]
Meng 2018	-2.9	3.4943	64	33	1.5%	-2.90 [-9.75, 3.95]
French 2018	-1	1.1674	192	217	2.3%	-1.00 [-3.29, 1.29]
Being Active Eating Well initiative (4)	-0.1	0.2828	351	681	2.5%	-0.10 [-0.65, 0.45]
Being Active Eating Well initiative (5)	-0.1	0.2236	1028	904	2.5%	-0.10 [-0.54, 0.34]
Black 2010	-0.023995	0.147456	0	0	2.5%	-0.02 [-0.31, 0.27]
Keys Trial	-0.02	0.3166	149	142	2.5%	-0.02 [-0.64, 0.60]
De Vries 2015	0	0.117	61	37	2.5%	0.00 [-0.23, 0.23]
Guelph Family Health Study	0.102963	0.38217	16	12	2.5%	0.10 [-0.65, 0.85]
van Grieken 2017	0.12	0.1217	221	217	2.5%	0.12 [-0.12, 0.36]
Foster 2008	0.3	0.3566	184	140	2.5%	0.30 [-0.40, 1.00]
Gong 2014	0.472422	0.112341	160	166	2.5%	0.47 [0.25, 0.69]
Wadolowska 2019	2.5	1.4284	319	145	2.2%	2.50 [-0.30, 5.30]
Memphis Girls health Enrichment Multi-site (GEMS)	7.5	8.928	116	127	0.4%	7.50 [-10.00, 25.00]
**Subtotal (95% CI)**			**2876**	**2841**	**29.1%**	**0.11 [-0.05, 0.27]**
Heterogeneity: Tau² = 0.02; Chi² = 19.24, df = 13 (P = 0.12); I² = 32%						
Test for overall effect: Z = 1.32 (P = 0.19)						
**1.10.3 Diet and exercise**						
Sherwood 2015	-38.6	13.9219	26	29	0.2%	-38.60 [-65.89, -11.31]
Campbell 2013	-8.5	4.6475	241	239	1.1%	-8.50 [-17.61, 0.61]
Grydeland 2013	-4	0.4049	215	485	2.5%	-4.00 [-4.79, -3.21]
Santos 2014	-2.27	716.7	182	151	0.0%	-2.27 [-1406.98, 1402.44]
Nyström 2017	-0.5	2.76	143	138	1.7%	-0.50 [-5.91, 4.91]
Dewar 2013	-0.23	1.91	31	57	2.1%	-0.23 [-3.97, 3.51]
Verbestel 2014	-0.16	0.2661	61	35	2.5%	-0.16 [-0.68, 0.36]
Bonis 2014	0	1.4604	110	99	2.2%	0.00 [-2.86, 2.86]
De Coen 2012 (6)	0.51	0.1541	1032	557	2.5%	0.51 [0.21, 0.81]
Iaia 2017	1.7	0.5927	199	266	2.5%	1.70 [0.54, 2.86]
Fitzgibbon 2005	7.4	0.2902	96	94	2.5%	7.40 [6.83, 7.97]
Moir 2016	13	0.2452	59	76	2.5%	13.00 [12.52, 13.48]
Marcus 2009	18	33.2463	48	47	0.0%	18.00 [-47.16, 83.16]
De Coen 2012 (7)	19.01	0.1948	1032	557	2.5%	19.01 [18.63, 19.39]
Novotny 2015	19.7	28.7	41	44	0.1%	19.70 [-36.55, 75.95]
Li 2014	20.7	4.2	334	483	1.2%	20.70 [12.47, 28.93]
**Subtotal (95% CI)**			**3850**	**3357**	**26.1%**	**3.25 [-1.89, 8.38]**
Heterogeneity: Tau² = 81.61; Chi² = 7780.43, df = 15 (P < 0.00001); I² = 100%						
Test for overall effect: Z = 1.24 (P = 0.22)						
**1.10.4 Exercise and behavioural therapy**						
Eliakim 2007	-21	7.5878	44	38	0.6%	-21.00 [-35.87, -6.13]
Amini 2016	-0.16	44.039	130	129	0.0%	-0.16 [-86.47, 86.15]
Amaro 2006	-0.1	0.1442	153	88	2.5%	-0.10 [-0.38, 0.18]
JuvenTUM 3	0.104867	0.183996	64	55	2.5%	0.10 [-0.26, 0.47]
Siegrist 2011	0.296921	0.094229	615	139	2.5%	0.30 [0.11, 0.48]
Madsen 2013	0.7	1.22	58	52	2.3%	0.70 [-1.69, 3.09]
Annesi 2017 (YF4L)	0.85	0.743	86	55	2.4%	0.85 [-0.61, 2.31]
CHIRPY DRAGON	1.33	1.8912	478	556	2.1%	1.33 [-2.38, 5.04]
WAVES study	2.27	21.7335	334	386	0.1%	2.27 [-40.33, 44.87]
Saraf 2014	2.9	1.4795	643	672	2.2%	2.90 [0.00, 5.80]
Fetter 2018	11.1	14.1585	19	17	0.2%	11.10 [-16.65, 38.85]
Angelopoulos 2009	12.1	2.0046	321	325	2.0%	12.10 [8.17, 16.03]
Annesi 2016	32.38	14.3697	72	42	0.2%	32.38 [4.22, 60.54]
Viggiano 2018	64.45	13.0665	206	147	0.2%	64.45 [38.84, 90.06]
**Subtotal (95% CI)**			**3223**	**2701**	**19.9%**	**0.84 [0.09, 1.59]**
Heterogeneity: Tau² = 0.63; Chi² = 83.12, df = 13 (P < 0.00001); I² = 84%						
Test for overall effect: Z = 2.19 (P = 0.03)						
**1.10.5 Diet only**						
Vandongen 1995 (8)	-0.8	0.297	17	17	2.5%	-0.80 [-1.38, -0.22]
Vandongen 1995 (9)	-0.1	0.2121	15	20	2.5%	-0.10 [-0.52, 0.32]
**Subtotal (95% CI)**			**32**	**37**	**5.0%**	**-0.42 [-1.10, 0.26]**
Heterogeneity: Tau² = 0.18; Chi² = 3.68, df = 1 (P = 0.06); I² = 73%						
Test for overall effect: Z = 1.20 (P = 0.23)						
**1.10.6 Diet, exercise and behavioural therapy**						
Dzewaltowski 2010	0.99	3.9114	64	63	1.3%	0.99 [-6.68, 8.66]
Gortmaker 2012	10.5	4.3	114	0	1.2%	10.50 [2.07, 18.93]
**Subtotal (95% CI)**			**178**	**63**	**2.5%**	**5.58 [-3.74, 14.89]**
Heterogeneity: Tau² = 28.33; Chi² = 2.68, df = 1 (P = 0.10); I² = 63%						
Test for overall effect: Z = 1.17 (P = 0.24)						
**Total (95% CI)**			**10388**	**9227**	**100.0%**	**1.87 [0.59, 3.15]**
Heterogeneity: Tau² = 16.95; Chi² = 12520.28, df = 55 (P < 0.00001); I² = 100%						
Test for overall effect: Z = 2.87 (P = 0.004)						
Test for subgroup differences: Chi² = 10.98, df = 5 (P = 0.05), I² = 54.5%						

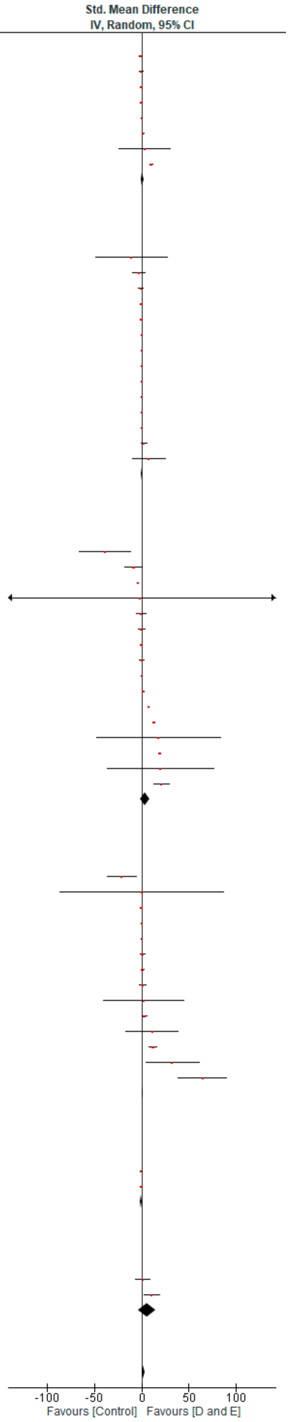

Footnotes
(1) Girls
(2) Boys
(3) E only
(4) Secondary School (in community 3 and 4)
(5) Primary School (in community 1 and 2)
(6) Participation in after school sports activities (h/week)
(7) Participants in sports club (hours/ week)
(8) Girls
(9) Boys

Figure S8: Impact of obesity prevention interventions on total caloric consumption

Study or Subgroup	Mean Difference	SE	Weight	Mean Difference IV, Random, 95% CI
**1.11.1 Exercise only**				
Fisher 2019	-159.9	66.1735	4.5%	-159.90 [-289.60, -30.20]
Lee 2010	71.8	72.8304	3.9%	71.80 [-70.94, 214.54]
Minnesota GEMS	-124	98.1	2.5%	-124.00 [-316.27, 68.27]
**Subtotal (95% CI)**			**10.9%**	**-68.73 [-220.74, 83.28]**
Heterogeneity: Tau² = 11874.77; Chi² = 5.94, df = 2 (P = 0.05); I² = 66%				
Test for overall effect: Z = 0.89 (P = 0.38)				
**1.11.2 Behavioural therapy only**				
Black 2010	-239.8	224.701723	0.5%	-239.80 [-680.21, 200.61]
De Moraes 2017	-116	90.7735	2.8%	-116.00 [-293.91, 61.91]
French 2018	-118	34.8574	9.3%	-118.00 [-186.32, -49.68]
Gong 2014	-33.2	49.638538	6.6%	-33.20 [-130.49, 64.09]
Memphis Girls health Enrichment Multi-site (GEMS)	-78	55.43	5.7%	-78.00 [-186.64, 30.64]
OSNAP Study	20.8	0.7736	15.4%	20.80 [19.28, 22.32]
**Subtotal (95% CI)**			**40.3%**	**-60.05 [-136.40, 16.30]**
Heterogeneity: Tau² = 5551.07; Chi² = 23.81, df = 5 (P = 0.0002); I² = 79%				
Test for overall effect: Z = 1.54 (P = 0.12)				
**1.11.3 Diet and exercise**				
De Coen 2012	80	9.8318	14.6%	80.00 [60.73, 99.27]
Donnelly 1996	-27	126.0313	1.6%	-27.00 [-274.02, 220.02]
Fitzgibbon 2005	80	5.6632	15.2%	80.00 [68.90, 91.10]
Harvey Barino 2003	-19.9	15.144768	13.7%	-19.90 [-49.58, 9.78]
Sherwood 2015	-111	110.3212	2.0%	-111.00 [-327.23, 105.23]
**Subtotal (95% CI)**			**47.1%**	**39.57 [-7.40, 86.53]**
Heterogeneity: Tau² = 1744.31; Chi² = 42.67, df = 4 (P < 0.00001); I² = 91%				
Test for overall effect: Z = 1.65 (P = 0.10)				
**1.11.4 Diet, exercise and behavioural therapy**				
Khan 2014	-59	121.8532	1.7%	-59.00 [-297.83, 179.83]
**Subtotal (95% CI)**			**1.7%**	**-59.00 [-297.83, 179.83]**
Heterogeneity: Not applicable				
Test for overall effect: Z = 0.48 (P = 0.63)				
**Total (95% CI)**			**100.0%**	**-8.88 [-41.73, 23.97]**
Heterogeneity: Tau² = 1821.04; Chi² = 186.33, df = 14 (P < 0.00001); I² = 92%				
Test for overall effect: Z = 0.53 (P = 0.60)				
Test for subgroup differences: Chi² = 6.05, df = 3 (P = 0.11), I² = 50.4%				

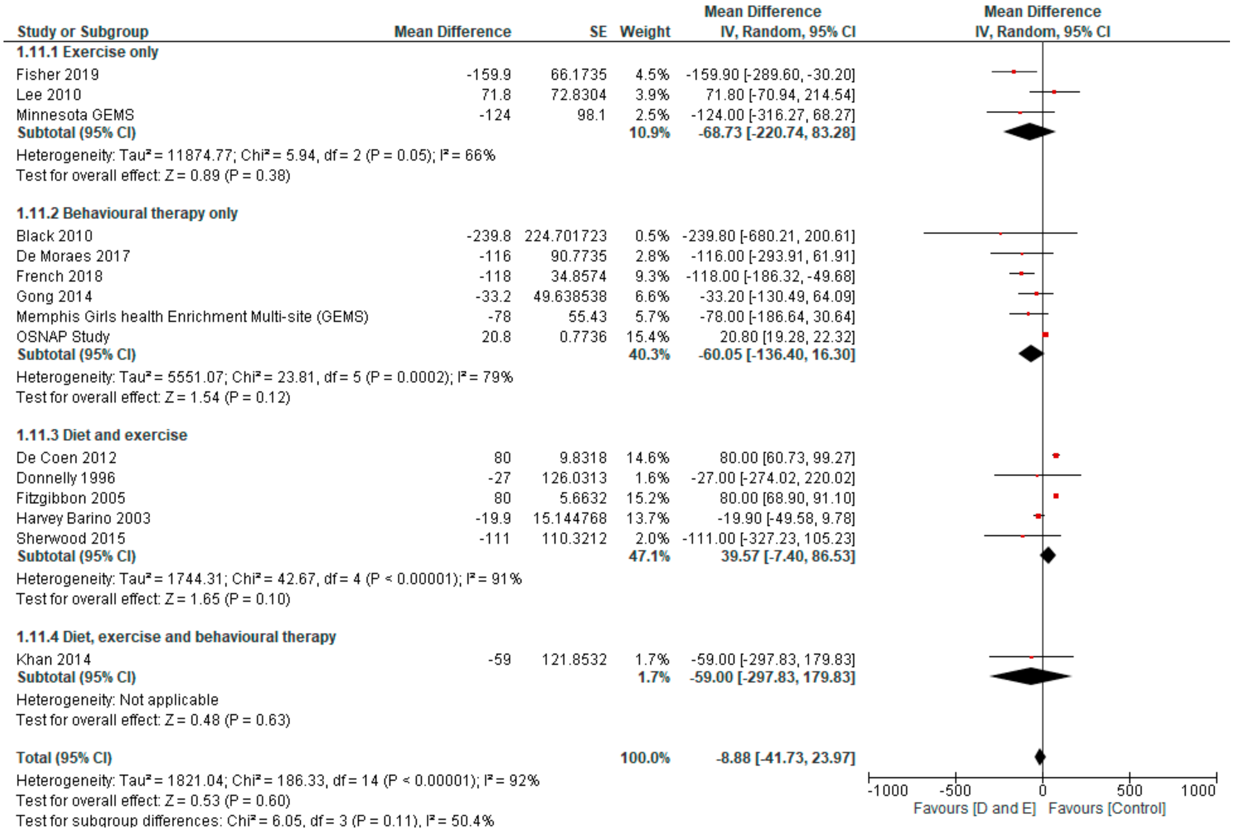

Figure S9: Impact of obesity management interventions on prevalence of overweight

Study or Subgroup	log[Risk Ratio]	SE	Experimental Total	Control Total	Weight	Risk Ratio IV, Random, 95% CI
**2.4.1 Diet and exercise**						
Kobel 2014	0.4104	0.2596	318	207	1.3%	1.51 [0.91, 2.51]
**Subtotal (95% CI)**			**318**	**207**	**1.3%**	**1.51 [0.91, 2.51]**
Heterogeneity: Not applicable						
Test for overall effect: Z = 1.58 (P = 0.11)						
**2.4.2 Diet, exercise and behavioural therapy**						
Braet 1997	-0.2566	0.2667	19	49	1.3%	0.77 [0.46, 1.30]
HEALTHY Study	0.0134	0.0323	2307	2296	85.5%	1.01 [0.95, 1.08]
**Subtotal (95% CI)**			**2326**	**2345**	**86.7%**	**1.01 [0.94, 1.08]**
Heterogeneity: Tau² = 0.00; Chi² = 1.01, df = 1 (P = 0.31); I² = 1%						
Test for overall effect: Z = 0.22 (P = 0.83)						
**2.4.3 Behavioural therapy only**						
Davoli 2013	0.0289	0.0952	187	185	9.8%	1.03 [0.85, 1.24]
Waling 2012	0.091	0.2061	37	35	2.1%	1.10 [0.73, 1.64]
**Subtotal (95% CI)**			**224**	**220**	**11.9%**	**1.04 [0.88, 1.23]**
Heterogeneity: Tau² = 0.00; Chi² = 0.07, df = 1 (P = 0.78); I² = 0%						
Test for overall effect: Z = 0.46 (P = 0.64)						
**Total (95% CI)**			**2868**	**2772**	**100.0%**	**1.02 [0.96, 1.08]**
Heterogeneity: Tau² = 0.00; Chi² = 3.50, df = 4 (P = 0.48); I² = 0%						
Test for overall effect: Z = 0.62 (P = 0.54)						
Test for subgroup differences: Chi² = 2.42, df = 2 (P = 0.30), I² = 17.2%						

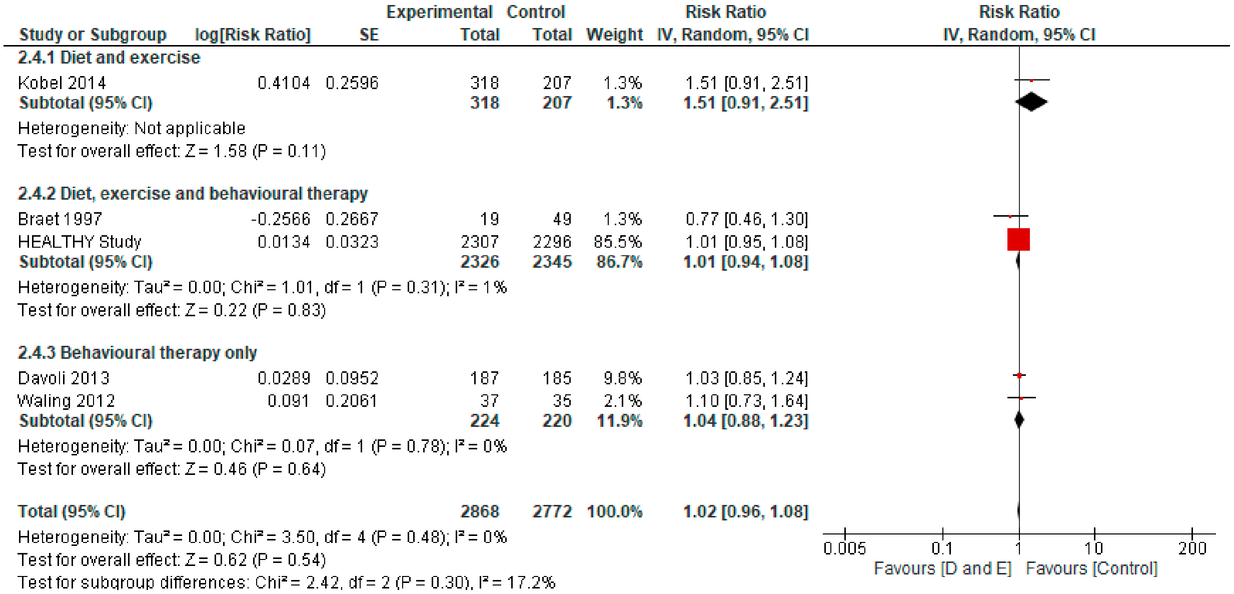

Figure S10: Impact of obesity management interventions on prevalence of obesity

Study or Subgroup	log[Risk Ratio]	SE	Experimental Total	Control Total	Weight	Risk Ratio IV, Random, 95% CI
**2.5.1 Diet and exercise**						
Kobel 2014	0.4104	0.2596	318	207	6.5%	1.51 [0.91, 2.51]
Lee 2014	-0.1975	0.2779	43	40	5.8%	0.82 [0.48, 1.42]
**Subtotal (95% CI)**			**361**	**247**	**12.3%**	**1.12 [0.62, 2.03]**
Heterogeneity: Tau² = 0.11; Chi² = 2.56, df = 1 (P = 0.11); I² = 61%						
Test for overall effect: Z = 0.38 (P = 0.71)						
**2.5.2 Behavioural therapy only**						
Davoli 2013	-0.2689	0.2034	187	185	9.6%	0.76 [0.51, 1.14]
Waling 2012	-0.0556	0.527	37	35	1.8%	0.95 [0.34, 2.66]
Yackobovitch-Gavan 2017	-0.1294	0.1517	61	49	14.5%	0.88 [0.65, 1.18]
**Subtotal (95% CI)**			**285**	**269**	**25.9%**	**0.84 [0.67, 1.06]**
Heterogeneity: Tau² = 0.00; Chi² = 0.35, df = 2 (P = 0.84); I² = 0%						
Test for overall effect: Z = 1.46 (P = 0.14)						
**2.5.3 Diet, exercise and behavioural therapy**						
HEALTHY Study	-0.0778	0.0503	2307	2296	33.2%	0.93 [0.84, 1.02]
Nemet 2013	0.1382	0.0717	147	67	28.5%	1.15 [1.00, 1.32]
**Subtotal (95% CI)**			**2454**	**2363**	**61.7%**	**1.02 [0.83, 1.27]**
Heterogeneity: Tau² = 0.02; Chi² = 6.08, df = 1 (P = 0.01); I² = 84%						
Test for overall effect: Z = 0.22 (P = 0.82)						
**Total (95% CI)**			**3100**	**2879**	**100.0%**	**0.98 [0.85, 1.13]**
Heterogeneity: Tau² = 0.01; Chi² = 11.37, df = 6 (P = 0.08); I² = 47%						
Test for overall effect: Z = 0.23 (P = 0.82)						
Test for subgroup differences: Chi² = 1.84, df = 2 (P = 0.40), I² = 0%						

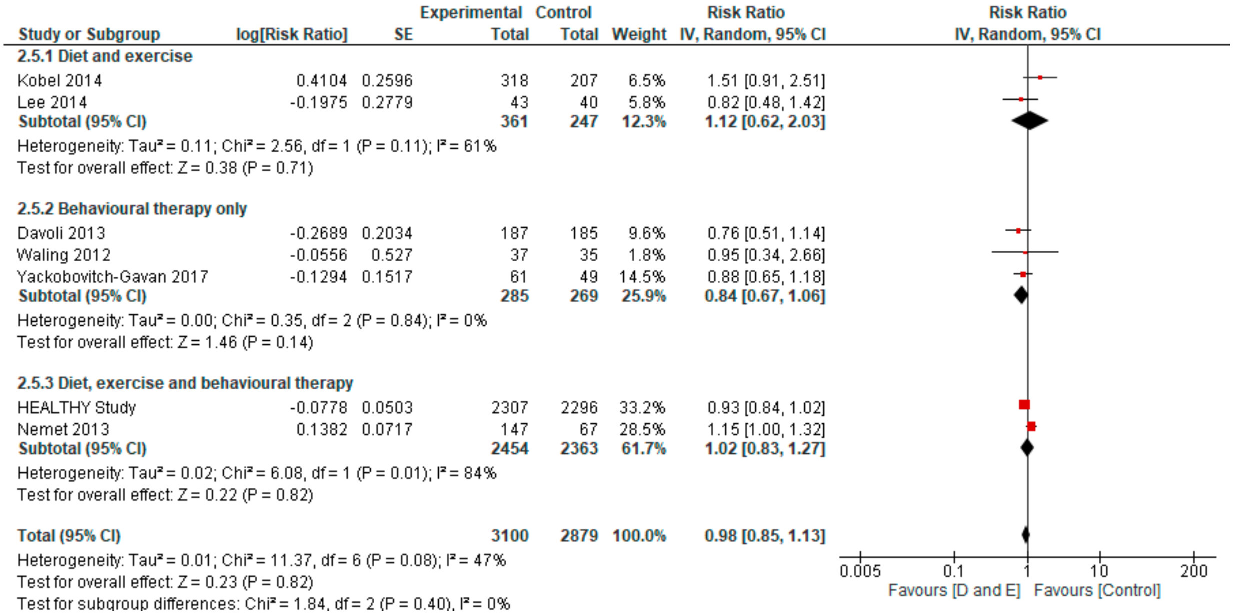

Figure S11: Impact of obesity management interventions on percentage body fat change

Study or Subgroup	Mean Difference	SE	Experimental Total	Control Total	Weight	Mean Difference IV, Random, 95% CI
**2.6.1 Diet only**						
Albala 2008	-0.42	0.0508	47	46	3.6%	-0.42 [-0.52, -0.32]
Casazza 2012	-0.2	0.320156	12	14	3.3%	-0.20 [-0.83, 0.43]
Ford 2009	-2.7	1.6478	43	46	1.0%	-2.70 [-5.93, 0.53]
Kong 2014	2.6	2.0875	34	27	0.7%	2.60 [-1.49, 6.69]
**Subtotal (95% CI)**			**136**	**133**	**8.5%**	**-0.36 [-0.81, 0.08]**
Heterogeneity: Tau² = 0.07; Chi² = 4.47, df = 3 (P = 0.22); I² = 33%						
Test for overall effect: Z = 1.60 (P = 0.11)						
**2.6.2 Exercise only**						
Ackel-D'Elia 2014 (1)	-6.27	0.8339	24	12	2.1%	-6.27 [-7.90, -4.64]
Ackel-D'Elia 2014 (2)	-0.15	0.9194	24	12	2.0%	-0.15 [-1.95, 1.65]
Aggeloussi 2012	-6.2	4.0256	9	9	0.2%	-6.20 [-14.09, 1.69]
Chen 2016	3.43	0.9929	25	25	1.8%	3.43 [1.48, 5.38]
Coimbra 2017	-0.9	2.911813	44	29	0.4%	-0.90 [-6.61, 4.81]
Cvetkovic 2018	1.55	2.922535	11	14	0.4%	1.55 [-4.18, 7.28]
Damaso 2014	1.2	1.530506	61	55	1.1%	1.20 [-1.80, 4.20]
Davis 2012	-1.42	0.387	73	78	3.1%	-1.42 [-2.18, -0.66]
Delgado-Floody 2018 (3)	-1.87	1.5106	49	18	1.1%	-1.87 [-4.83, 1.09]
Delgado-Floody 2018 (4)	-0.66	1.6592	27	8	1.0%	-0.66 [-3.91, 2.59]
Delgado-Floody 2018 (5)	-0.63	2.2464	43	11	0.6%	-0.63 [-5.03, 3.77]
Delgado-Floody 2018 (6)	-3.07	1.8484	32	9	0.8%	-3.07 [-6.69, 0.55]
Dennis 2013	-2.3	0.5678	36	42	2.7%	-2.30 [-3.41, -1.19]
Faith 2001	5.3	2.1535	6	4	0.6%	5.30 [1.08, 9.52]
Fiorilli 2017	-2.28	1.8055	14	12	0.9%	-2.28 [-5.82, 1.26]
Jones 2015 (7)	-6.68	3.3173	10	10	0.3%	-6.68 [-13.18, -0.18]
Jones 2015 (8)	-1.52	2.4696	9	8	0.5%	-1.52 [-6.36, 3.32]
Monteiro 2015	-4.86	1.3219	14	8	1.3%	-4.86 [-7.45, -2.27]
Morgan 2012	-1.8	0.7912	50	50	2.2%	-1.80 [-3.35, -0.25]
Nobre 2017	-3.1	2.6929	40	19	0.4%	-3.10 [-8.38, 2.18]
Nowicka 2009	-0.6	1.6951	38	38	0.9%	-0.60 [-3.92, 2.72]
Schaeffer 2014	-4	1.7114	10	8	0.9%	-4.00 [-7.35, -0.65]
Schranz 2014	3.6	2.049	30	26	0.7%	3.60 [-0.42, 7.62]
Seabra 2016	-5.4	2.1898	29	30	0.6%	-5.40 [-9.69, -1.11]
Seo 2019	-0.83	1.131	26	44	1.6%	-0.83 [-3.05, 1.39]
Shaibi 2006	2.1	0.761	11	11	2.3%	2.10 [0.61, 3.59]
Son 2017	-3.45	0.6481	20	20	2.5%	-3.45 [-4.72, -2.18]
Tkacz 2008	-0.3	0.904	139	69	2.0%	-0.30 [-2.07, 1.47]
Vasconcellos 2015	-2.8	2.5144	10	10	0.5%	-2.80 [-7.73, 2.13]
Zehsaz 2016	-1.1	2.4623	16	16	0.5%	-1.10 [-5.93, 3.73]
**Subtotal (95% CI)**			**930**	**705**	**36.2%**	**-1.36 [-2.32, -0.39]**
Heterogeneity: Tau² = 4.49; Chi² = 133.52, df = 29 (P < 0.00001); I² = 78%						
Test for overall effect: Z = 2.76 (P = 0.006)						
**2.6.3 Behavioural therapy only**						
Ahmad 2018	0.257	1.1026	67	67	1.6%	0.26 [-1.90, 2.42]
HELP trial	-0.21	0.691	87	87	2.4%	-0.21 [-1.56, 1.14]
Hystad 2013	0	1.426	36	44	1.2%	0.00 [-2.79, 2.79]
Kalavainen 2007	0.6	1.0405	35	35	1.7%	0.60 [-1.44, 2.64]
Kokkvoll 2014	-1.11	1.079	38	31	1.7%	-1.11 [-3.22, 1.00]
Nawi 2015	0.09	6	47	50	0.1%	0.09 [-11.67, 11.85]
Taylor 2015	-0.7	0.8588	89	92	2.1%	-0.70 [-2.38, 0.98]
**Subtotal (95% CI)**			**399**	**406**	**10.9%**	**-0.24 [-1.00, 0.52]**
Heterogeneity: Tau² = 0.00; Chi² = 1.83, df = 6 (P = 0.94); I² = 0%						
Test for overall effect: Z = 0.61 (P = 0.54)						
**2.6.4 Diet and exercise**						
Bernsten 2010	1.6	1.7997	32	16	0.9%	1.60 [-1.93, 5.13]
Bharath 2018	-3.4	3.041381	20	20	0.4%	-3.40 [-9.36, 2.56]
Coppins 2011	-0.8	1.6708	35	30	1.0%	-0.80 [-4.07, 2.47]
da Silva 2013	-2.3	1.4859	108	130	1.1%	-2.30 [-5.21, 0.61]
Diaz 2010	-0.3	1.7904	21	22	0.9%	-0.30 [-3.81, 3.21]
Lee 2014	-1.82	7.1439	43	40	0.1%	-1.82 [-15.82, 12.18]
Lopes 2016	-2.5	1.9301	17	16	0.8%	-2.50 [-6.28, 1.28]
Nemet 2005	6.1	3.53	20	20	0.3%	6.10 [-0.82, 13.02]
Salazar 2014	10.3	1.2404	35	85	1.4%	10.30 [7.87, 12.73]
Shalitin 2009 (9)	-2.1	0.2889	28	55	3.3%	-2.10 [-2.67, -1.53]
Shalitin 2009 (10)	-1	0.3399	27	52	3.2%	-1.00 [-1.67, -0.33]
Yin 2005	-1	0.9225	603	584	2.0%	-1.00 [-2.81, 0.81]
**Subtotal (95% CI)**			**989**	**1070**	**15.2%**	**0.23 [-1.50, 1.96]**
Heterogeneity: Tau² = 6.11; Chi² = 104.58, df = 11 (P < 0.00001); I² = 89%						
Test for overall effect: Z = 0.26 (P = 0.79)						
**2.6.5 Exercise and behavioural therapy**						
Bryant 2011	1.2	0.923258	35	35	2.0%	1.20 [-0.61, 3.01]
Chae 2010	1.2	0.394702	19	19	3.1%	1.20 [0.43, 1.97]
Dias 2018	0.5	2.3326	18	16	0.6%	0.50 [-4.07, 5.07]
Herget 2016	2	0.688	14	14	2.5%	2.00 [0.65, 3.35]
Nemet 2013	-0.8	2.248995	22	18	0.6%	-0.80 [-5.21, 3.61]
Odense overweight intervention study 2015	-2.9	1.6781	47	39	0.9%	-2.90 [-6.19, 0.39]
Shaw 2008	-8.99	2.319	13	10	0.6%	-8.99 [-13.54, -4.44]
Vajda 2007	-1.79	0.2472	21	28	3.4%	-1.79 [-2.27, -1.31]
**Subtotal (95% CI)**			**189**	**179**	**13.6%**	**-0.68 [-2.43, 1.08]**
Heterogeneity: Tau² = 4.57; Chi² = 77.01, df = 7 (P < 0.00001); I² = 91%						
Test for overall effect: Z = 0.76 (P = 0.45)						
**2.6.6 Diet and behavioural therapy**						
Koo 2018	-3.8	1.2021	63	63	1.5%	-3.80 [-6.16, -1.44]
**Subtotal (95% CI)**			**63**	**63**	**1.5%**	**-3.80 [-6.16, -1.44]**
Heterogeneity: Not applicable						
Test for overall effect: Z = 3.16 (P = 0.002)						
**2.6.7 Diet, exercise and behavioural therapy**						
Caballero 2003	0.3	0.0532	457	1247	3.6%	0.30 [0.20, 0.40]
Chen 2011	0.21	0.6315	27	27	2.6%	0.21 [-1.03, 1.45]
Jensen 2015 (11)	-1.6	1.9839	32	11	0.7%	-1.60 [-5.49, 2.29]
Jensen 2015 (12)	-0.6	1.9741	31	11	0.7%	-0.60 [-4.47, 3.27]
Lopera 2016	-6.8	1.8113	57	33	0.8%	-6.80 [-10.35, -3.25]
Reinehr 2003	-1	0.7084	34	32	2.4%	-1.00 [-2.39, 0.39]
Robertson 2017	1.87	1.45	45	43	1.2%	1.87 [-0.97, 4.71]
Savoye 2007	-6	0.96	75	44	1.9%	-6.00 [-7.88, -4.12]
Wesnigk 2016	19.77	4.0444	8	8	0.2%	19.77 [11.84, 27.70]
**Subtotal (95% CI)**			**766**	**1456**	**14.2%**	**-0.72 [-2.56, 1.12]**
Heterogeneity: Tau² = 5.76; Chi² = 86.98, df = 8 (P < 0.00001); I² = 91%						
Test for overall effect: Z = 0.77 (P = 0.44)						
**Total (95% CI)**			**3472**	**4012**	**100.0%**	**-0.78 [-1.16, -0.41]**
Heterogeneity: Tau² = 1.01; Chi² = 590.70, df = 70 (P < 0.00001); I² = 88%						
Test for overall effect: Z = 4.10 (P < 0.0001)						
Test for subgroup differences: Chi² = 12.15, df = 6 (P = 0.06), I² = 50.6%						

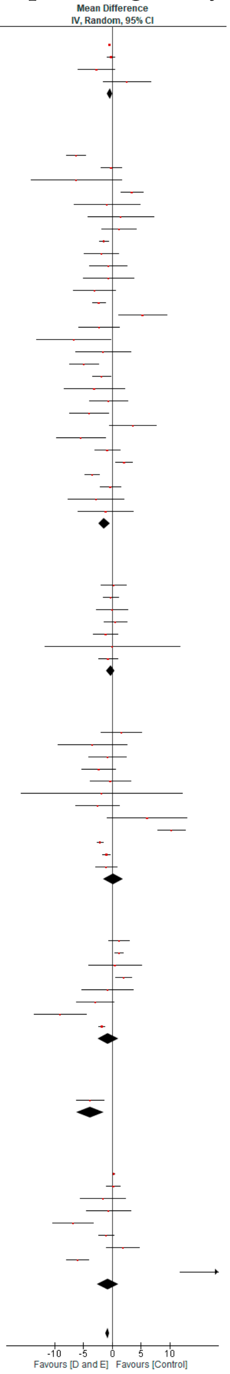

Footnotes

(1) Aerobic + resistance training
(2) Aerobic training group
(3) EG2: Obese girls
(4) EG1: Overweight boys
(5) EG2: Obese boys
(6) EG1: Overweight girls
(7) Boys
(8) Girls
(9) D and E vs Diet only
(10) D and E vs Exercise only
(11) Reduced CHO diet
(12) Low fat group

Figure S12: Impact of obesity management interventions on skinfold thickness (Tricep)

Study or Subgroup	Mean Difference	SE	Experimental Total	Control Total	Weight	Mean Difference IV, Random, 95% CI
**2.7.1 Behavioural therapy only**						
Kokkvoll 2014	-0.4	0.6632	38	31	17.3%	-0.40 [-1.70, 0.90]
**Subtotal (95% CI)**			**38**	**31**	**17.3%**	**-0.40 [-1.70, 0.90]**
Heterogeneity: Not applicable						
Test for overall effect: Z = 0.60 (P = 0.55)						
**2.7.2 Exercise only**						
Krombholz 2012	0.4	0.2836	211	217	18.7%	0.40 [-0.16, 0.96]
Nobre 2017	-1.3	1.4766	40	19	12.9%	-1.30 [-4.19, 1.59]
Schranz 2014	1.3	23.2	30	26	0.2%	1.30 [-44.17, 46.77]
**Subtotal (95% CI)**			**281**	**262**	**31.7%**	**0.34 [-0.21, 0.89]**
Heterogeneity: Tau² = 0.00; Chi² = 1.28, df = 2 (P = 0.53); I² = 0%						
Test for overall effect: Z = 1.22 (P = 0.22)						
**2.7.3 Exercise and behavioural therapy**						
Nayak 2016	-6.9	0.567209	90	104	17.7%	-6.90 [-8.01, -5.79]
**Subtotal (95% CI)**			**90**	**104**	**17.7%**	**-6.90 [-8.01, -5.79]**
Heterogeneity: Not applicable						
Test for overall effect: Z = 12.16 (P < 0.00001)						
**2.7.4 Diet, exercise and behavioural therapy**						
Family partners for health study	-2.5	0.0398	304	290	19.0%	-2.50 [-2.58, -2.42]
Reinehr 2003	-5	1.2515	34	32	14.2%	-5.00 [-7.45, -2.55]
**Subtotal (95% CI)**			**338**	**322**	**33.2%**	**-3.44 [-5.81, -1.07]**
Heterogeneity: Tau² = 2.34; Chi² = 3.99, df = 1 (P = 0.05); I² = 75%						
Test for overall effect: Z = 2.84 (P = 0.005)						
**Total (95% CI)**			**747**	**719**	**100.0%**	**-2.57 [-4.41, -0.73]**
Heterogeneity: Tau² = 4.66; Chi² = 178.39, df = 6 (P < 0.00001); I² = 97%						
Test for overall effect: Z = 2.73 (P = 0.006)						
Test for subgroup differences: Chi² = 136.11, df = 3 (P < 0.00001), I² = 97.8%						

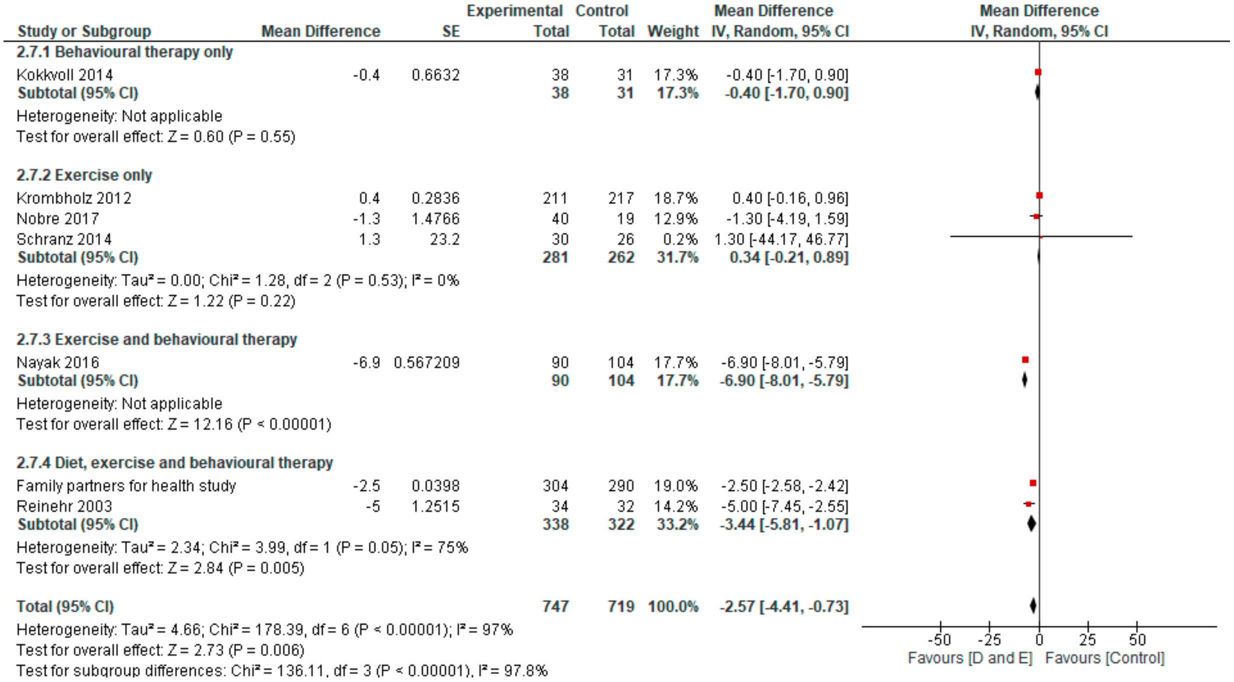

Figure S13: Impact of obesity management interventions on waist circumference

Study or Subgroup	Mean Difference	SE	Experimental Total	Control Total	Weight	Mean Difference IV, Random, 95% CI
**2.8.1 Diet only**						
Berkowitz 2011 (1)	-2	0.1716	65	37	4.6%	-2.00 [-2.34, -1.66]
Kong 2014	0.7	2.4441	52	27	0.7%	0.70 [-4.09, 5.49]
Mendes 2016	2.1	3.2931	25	19	0.4%	2.10 [-4.35, 8.55]
**Subtotal (95% CI)**			**142**	**83**	**5.8%**	**-1.22 [-3.29, 0.86]**
Heterogeneity: Tau² = 1.45; Chi² = 2.75, df = 2 (P = 0.25); I² = 27%						
Test for overall effect: Z = 1.15 (P = 0.25)						
**2.8.2 Exercise only**						
Christison 2016	-0.3	2.919717	59	21	0.5%	-0.30 [-6.02, 5.42]
Coimbra 2017	-7.9	5.832259	44	29	0.1%	-7.90 [-19.33, 3.53]
Delgado-Floody 2018 (2)	-1.08	2.3551	27	8	0.7%	-1.08 [-5.70, 3.54]
Delgado-Floody 2018 (3)	-3.82	2.0766	32	9	0.9%	-3.82 [-7.89, 0.25]
Delgado-Floody 2018 (4)	-1.87	3.4031	43	11	0.4%	-1.87 [-8.54, 4.80]
Delgado-Floody 2018 (5)	-0.96	2.2201	49	18	0.8%	-0.96 [-5.31, 3.39]
Dennis 2013	-2.9	0.8754	36	42	2.7%	-2.90 [-4.62, -1.18]
Fiorilli 2017	-1.76	3.8544	14	12	0.3%	-1.76 [-9.31, 5.79]
Jones 2015 (6)	-7.12	4.8484	10	10	0.2%	-7.12 [-16.62, 2.38]
Jones 2015 (7)	-0.28	3.686	9	8	0.3%	-0.28 [-7.50, 6.94]
Monteiro 2015	-10	3.2797	14	16	0.4%	-10.00 [-16.43, -3.57]
Seabra 2016	-6.1	2.8557	29	30	0.5%	-6.10 [-11.70, -0.50]
Seo 2019	2.65	2.8354	26	44	0.5%	2.65 [-2.91, 8.21]
Sigal 2014	-2.1	1.8376	75	76	1.1%	-2.10 [-5.70, 1.50]
Son 2017	-1.8	0.2729	20	20	4.4%	-1.80 [-2.33, -1.27]
Zehsaz 2016	-1.9	1.9561	16	16	1.0%	-1.90 [-5.73, 1.93]
**Subtotal (95% CI)**			**503**	**370**	**15.2%**	**-2.14 [-2.90, -1.38]**
Heterogeneity: Tau² = 0.19; Chi² = 16.11, df = 15 (P = 0.37); I² = 7%						
Test for overall effect: Z = 5.51 (P < 0.00001)						
**2.8.3 Behavioural therapy only**						
Ahmad 2018	2.716	1.7523	67	67	1.2%	2.72 [-0.72, 6.15]
Bagherniya 2017	1.6	1.3496	73	81	1.7%	1.60 [-1.05, 4.25]
Ball 2011 (8)	-8.3	6.451	10	5	0.1%	-8.30 [-20.94, 4.34]
Ball 2011 (9)	-8.6	7.2002	9	6	0.1%	-8.60 [-22.71, 5.51]
Berkowitz 2013	-0.54	0.1585	81	88	4.7%	-0.54 [-0.85, -0.23]
Boodai 2014	1.4	1.449301	31	32	1.6%	1.40 [-1.44, 4.24]
Croker 2012	-0.9	0.8314	33	30	2.9%	-0.90 [-2.53, 0.73]
HELP trial	-0.72	1.306	87	87	1.8%	-0.72 [-3.28, 1.84]
Kalavainen 2007	0.6	1.7102	35	35	1.2%	0.60 [-2.75, 3.95]
Kokkvoll 2014	-0.12	1.1479	38	31	2.1%	-0.12 [-2.37, 2.13]
Nawi 2015	-5.57	2.308264	47	50	0.8%	-5.57 [-10.09, -1.05]
Taylor 2015	-1.8	1.5753	89	92	1.4%	-1.80 [-4.89, 1.29]
Vasconcellos 2015	-0.06	0.0894	10	10	4.7%	-0.06 [-0.24, 0.12]
Walpole 2013	0.75	3.0681	20	18	0.5%	0.75 [-5.26, 6.76]
**Subtotal (95% CI)**			**630**	**632**	**24.7%**	**-0.27 [-0.75, 0.21]**
Heterogeneity: Tau² = 0.15; Chi² = 23.30, df = 13 (P = 0.04); I² = 44%						
Test for overall effect: Z = 1.10 (P = 0.27)						
**2.8.4 Diet and exercise**						
Bharath 2018	-4.7	1.118034	20	20	2.2%	-4.70 [-6.89, -2.51]
Coppins 2011	-2.2	2.8905	35	30	0.5%	-2.20 [-7.87, 3.47]
Graf 2006	1.5	2.857586	0	0	0.5%	1.50 [-4.10, 7.10]
Lee 2012 (10)	-3.1	0.35	16	13	4.3%	-3.10 [-3.79, -2.41]
Lee 2012 (11)	-4.3	0.35	16	13	4.3%	-4.30 [-4.99, -3.61]
Serra-Paya 2015	0.18	1.22	54	59	1.9%	0.18 [-2.21, 2.57]
Tarro 2014	0.81	0.76	421	198	3.1%	0.81 [-0.68, 2.30]
Toulabi 2012	-4.17	1.3529	76	76	1.7%	-4.17 [-6.82, -1.52]
Yin 2005	0.1	1.0743	603	584	2.2%	0.10 [-2.01, 2.21]
**Subtotal (95% CI)**			**1241**	**993**	**20.7%**	**-2.03 [-3.50, -0.56]**
Heterogeneity: Tau² = 3.53; Chi² = 59.66, df = 8 (P < 0.00001); I² = 87%						
Test for overall effect: Z = 2.70 (P = 0.007)						
**2.8.5 Exercise and behavioral therapy**						
Bryant 2011	-0.05	0.101143	35	35	4.7%	-0.05 [-0.25, 0.15]
Farah 2013	-5.1	1.9288	9	10	1.0%	-5.10 [-8.88, -1.32]
Garcia 2019	-2.3	2.068346	0	0	0.9%	-2.30 [-6.35, 1.75]
Herget 2016	1.8	0.936	14	14	2.6%	1.80 [-0.03, 3.63]
Md Yusop 2018	-1.3	2.3248	20	20	0.8%	-1.30 [-5.86, 3.26]
Odense overweight intervention study 2015	0	1.8886	48	38	1.1%	0.00 [-3.70, 3.70]
Sepulveda 2020	4	2.2087	25	24	0.8%	4.00 [-0.33, 8.33]
**Subtotal (95% CI)**			**151**	**141**	**11.9%**	**-0.16 [-1.72, 1.39]**
Heterogeneity: Tau² = 2.10; Chi² = 15.59, df = 6 (P = 0.02); I² = 62%						
Test for overall effect: Z = 0.20 (P = 0.84)						
**2.8.6 Diet and behavioural therapy**						
Koo 2018	-4.4	1.3811	63	63	1.7%	-4.40 [-7.11, -1.69]
Moschonis 2019	1.2	3.114482	35	30	0.5%	1.20 [-4.90, 7.30]
**Subtotal (95% CI)**			**98**	**93**	**2.1%**	**-2.30 [-7.61, 3.02]**
Heterogeneity: Tau² = 9.88; Chi² = 2.70, df = 1 (P = 0.10); I² = 63%						
Test for overall effect: Z = 0.85 (P = 0.40)						
**2.8.7 Diet, exercise and behavioural therapy**						
Bartelink 2014	-6.13	1.7219	67	29	1.2%	-6.13 [-9.50, -2.76]
Burrows 2012	-0.8	0.51	60	72	3.8%	-0.80 [-1.80, 0.20]
Family partners for health study (12)	-1.5	0.0398	304	209	4.8%	-1.50 [-1.58, -1.42]
Guo 2015	-1.33	2.3867	23	13	0.7%	-1.33 [-6.01, 3.35]
HEALTHY Study	-0.4	0.730032	824	820	3.1%	-0.40 [-1.83, 1.03]
Jensen 2015 (13)	-8.5	4.9955	31	11	0.2%	-8.50 [-18.29, 1.29]
Jensen 2015 (14)	-8.1	5.246	32	11	0.2%	-8.10 [-18.38, 2.18]
Kalarchian 2009	-3.41	1.36	97	95	1.7%	-3.41 [-6.08, -0.74]
Lopera 2016	1.6	1.8453	57	66	1.1%	1.60 [-2.02, 5.22]
Reinehr 2003	-4	1.3565	34	32	1.7%	-4.00 [-6.66, -1.34]
Robertson 2017	3.89	2.42	45	43	0.7%	3.89 [-0.85, 8.63]
Shelton 2007	2.46	3.3715	28	15	0.4%	2.46 [-4.15, 9.07]
**Subtotal (95% CI)**			**1602**	**1416**	**19.7%**	**-1.54 [-2.56, -0.52]**
Heterogeneity: Tau² = 1.17; Chi² = 29.46, df = 11 (P = 0.002); I² = 63%						
Test for overall effect: Z = 2.97 (P = 0.003)						
**Total (95% CI)**			**4367**	**3728**	**100.0%**	**-1.33 [-1.76, -0.90]**
Heterogeneity: Tau² = 1.03; Chi² = 624.25, df = 62 (P < 0.00001); I² = 90%						
Test for overall effect: Z = 6.01 (P < 0.00001)						
Test for subgroup differences: Chi² = 21.83, df = 6 (P = 0.001), I² = 72.5%						

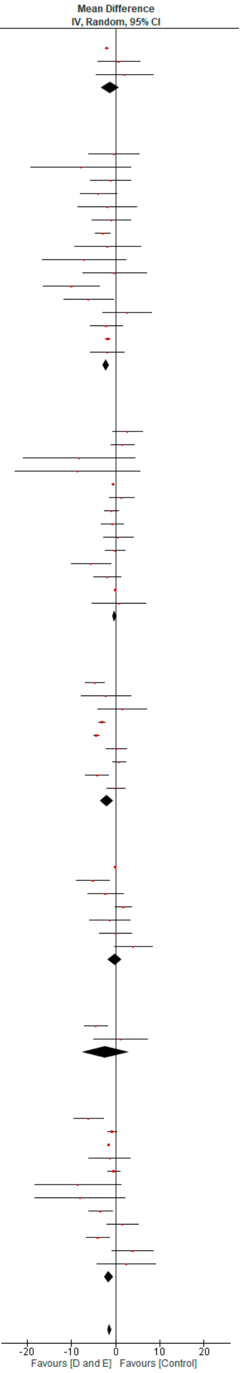

Footnotes
(1) W
(2) EG1: Overweight boys
(3) EG1: Overweight girls
(4) EG2: Obese boys
(5) EG2: Obese girls
(6) Boys
(7) Girls
(8) Youth Lifestyle Program
(9) Healthy Initiative Program
(10) Aerobic
(11) Resistance
(12) Waist
(13) Low fat group
(14) Reduced CHO diet

Figure S14: Impact of obesity management interventions on health-related quality of life

Study or Subgroup	Mean Difference	SE	Weight	Mean Difference IV, Random, 95% CI
**2.9.1 Behavioural therapy only**				
Chen 2019	6.86	5.525796	0.5%	6.86 [-3.97, 17.69]
HELP trial	0.14	1.87	4.2%	0.14 [-3.53, 3.81]
Tyler 2016	-3.58	6.14	0.4%	-3.58 [-15.61, 8.45]
**Subtotal (95% CI)**			**5.1%**	**0.49 [-2.84, 3.83]**
Heterogeneity: Tau² = 0.00; Chi² = 1.80, df = 2 (P = 0.41); I² = 0%				
Test for overall effect: Z = 0.29 (P = 0.77)				
**2.9.2 Diet and exercise**				
Alberga 2015	1.2	31.44	0.0%	1.20 [-60.42, 62.82]
**Subtotal (95% CI)**			**0.0%**	**1.20 [-60.42, 62.82]**
Heterogeneity: Not applicable				
Test for overall effect: Z = 0.04 (P = 0.97)				
**2.9.3 Exercise and behavioral therapy**				
Garcia 2019	5	14.672651	0.1%	5.00 [-23.76, 33.76]
**Subtotal (95% CI)**			**0.1%**	**5.00 [-23.76, 33.76]**
Heterogeneity: Not applicable				
Test for overall effect: Z = 0.34 (P = 0.73)				
**2.9.4 Diet and behavioural therapy**				
JenMe Program	-0.1	0.469295	66.7%	-0.10 [-1.02, 0.82]
**Subtotal (95% CI)**			**66.7%**	**-0.10 [-1.02, 0.82]**
Heterogeneity: Not applicable				
Test for overall effect: Z = 0.21 (P = 0.83)				
**2.9.5 Diet, exercise and behavioural therapy**				
Hofsteenge 2013	4.5	3.03	1.6%	4.50 [-1.44, 10.44]
Lopera 2016	1.6	2.3726	2.6%	1.60 [-3.05, 6.25]
Taveras 2017	0.3	0.8491	20.4%	0.30 [-1.36, 1.96]
**Subtotal (95% CI)**			**24.6%**	**0.71 [-0.80, 2.23]**
Heterogeneity: Tau² = 0.00; Chi² = 1.94, df = 2 (P = 0.38); I² = 0%				
Test for overall effect: Z = 0.92 (P = 0.36)				
**2.9.6 Exercise only**				
Weintraub 2008	-1.17	2.0152	3.6%	-1.17 [-5.12, 2.78]
**Subtotal (95% CI)**			**3.6%**	**-1.17 [-5.12, 2.78]**
Heterogeneity: Not applicable				
Test for overall effect: Z = 0.58 (P = 0.56)				
**Total (95% CI)**			**100.0%**	**0.09 [-0.66, 0.85]**
Heterogeneity: Tau² = 0.00; Chi² = 5.11, df = 9 (P = 0.82); I² = 0%				
Test for overall effect: Z = 0.25 (P = 0.81)				
Test for subgroup differences: Chi² = 1.37, df = 5 (P = 0.93), I² = 0%				

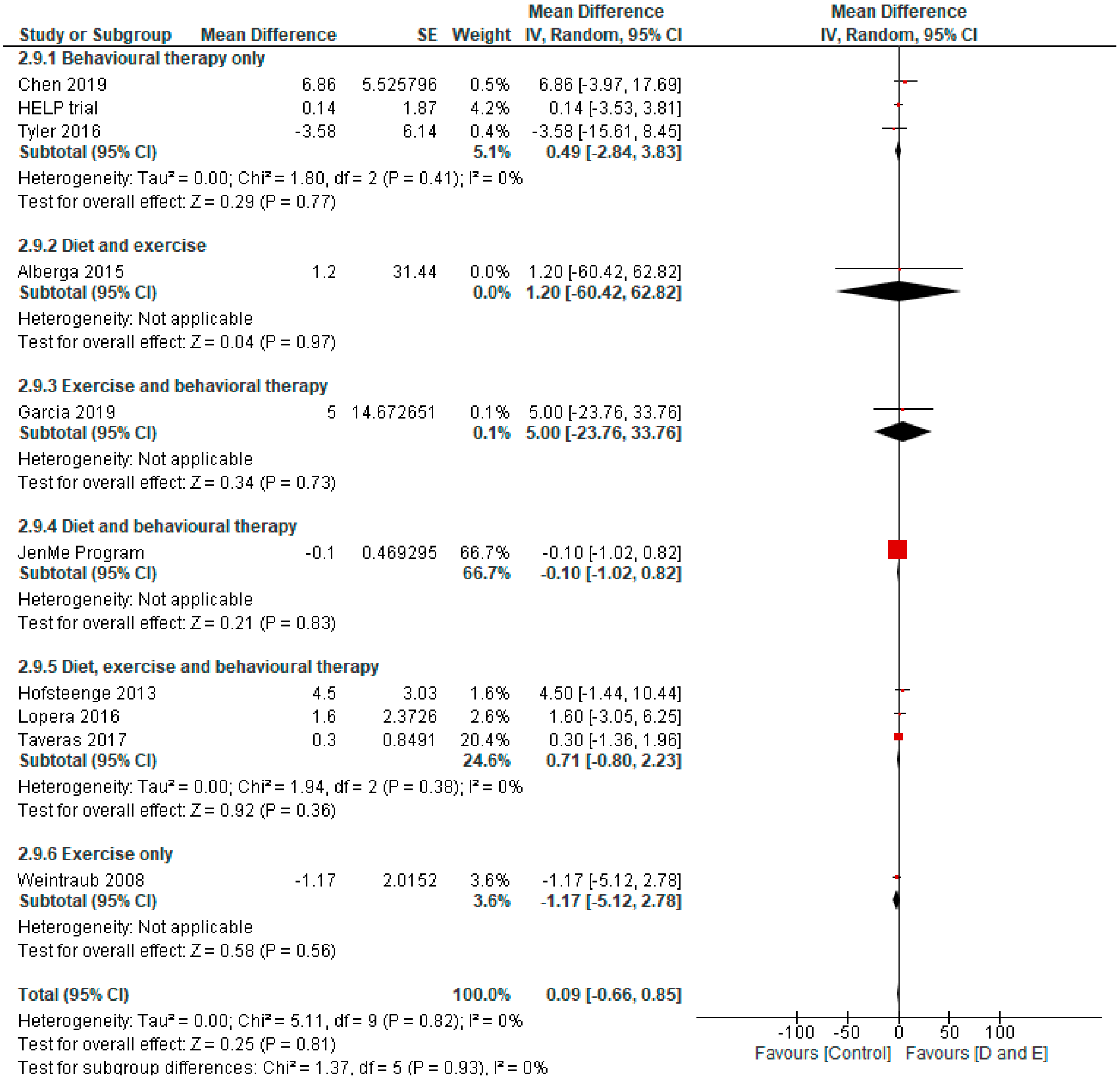

Figure S15: Impact of obesity management interventions on physical activity intensity

Study or Subgroup	Std. Mean Difference	SE	Weight	Std. Mean Difference IV, Random, 95% CI
**2.10.1 Exercise only**				
Aggeloussi 2012	14.2	0.7902	7.8%	14.20 [12.65, 15.75]
Seabra 2016	4.8	4.6298	2.4%	4.80 [-4.27, 13.87]
Weintraub 2008	1.32	1.2643	7.1%	1.32 [-1.16, 3.80]
**Subtotal (95% CI)**			**17.3%**	**6.95 [-3.37, 17.27]**
Heterogeneity: Tau² = 76.24; Chi² = 76.16, df = 2 (P < 0.00001); I² = 97%				
Test for overall effect: Z = 1.32 (P = 0.19)				
**2.10.2 Behavioural therapy only**				
Bagherniya 2017 (1)	31.9	4.1283	2.9%	31.90 [23.81, 39.99]
Davis 2013	0.264554	0.264466	8.3%	0.26 [-0.25, 0.78]
Eren 2017	25	3.2571	3.8%	25.00 [18.62, 31.38]
FRESH study	-0.071404	0.385308	8.2%	-0.07 [-0.83, 0.68]
Kulik 2015	-8.1	5.1918	2.1%	-8.10 [-18.28, 2.08]
Tucker 2019	-0.2	14.3617	0.3%	-0.20 [-28.35, 27.95]
**Subtotal (95% CI)**			**25.5%**	**7.36 [3.60, 11.12]**
Heterogeneity: Tau² = 12.12; Chi² = 119.63, df = 5 (P < 0.00001); I² = 96%				
Test for overall effect: Z = 3.84 (P = 0.0001)				
**2.10.3 Diet and exercise**				
Bernsten 2010 (2)	-13	10.917	0.6%	-13.00 [-34.40, 8.40]
Gillis 2007	-2.8	4.4118	2.6%	-2.80 [-11.45, 5.85]
Kobel 2014	22	2.4551	5.0%	22.00 [17.19, 26.81]
Nemet 2005	27.5	3.0628	4.0%	27.50 [21.50, 33.50]
Serra-Paya 2015	6.88	1.18	7.2%	6.88 [4.57, 9.19]
Wright 2014	1.54	1.92	5.9%	1.54 [-2.22, 5.30]
**Subtotal (95% CI)**			**25.3%**	**8.94 [0.06, 17.83]**
Heterogeneity: Tau² = 105.44; Chi² = 94.03, df = 5 (P < 0.00001); I² = 95%				
Test for overall effect: Z = 1.97 (P = 0.05)				
**2.10.4 Exercise and behavioural therapy**				
Backlund 2011	-100	1,024.1247	0.0%	-100.00 [-2107.25, 1907.25]
Cliff 2011	0.082499	0.270152	8.3%	0.08 [-0.45, 0.61]
Dias 2018	0	3.5757	3.4%	0.00 [-7.01, 7.01]
Nemet 2013	0.691704	0.328092	8.2%	0.69 [0.05, 1.33]
Silva 2018	9.8	8.4414	0.9%	9.80 [-6.74, 26.34]
**Subtotal (95% CI)**			**20.8%**	**0.33 [-0.07, 0.74]**
Heterogeneity: Tau² = 0.00; Chi² = 3.33, df = 4 (P = 0.50); I² = 0%				
Test for overall effect: Z = 1.60 (P = 0.11)				
**2.10.5 Diet and behavioural therapy**				
Golan 1998	0.93	0.1509	8.3%	0.93 [0.63, 1.23]
**Subtotal (95% CI)**			**8.3%**	**0.93 [0.63, 1.23]**
Heterogeneity: Not applicable				
Test for overall effect: Z = 6.16 (P < 0.00001)				
**2.10.6 Diet, exercise and behavioral therapy**				
Dzewaltowski 2010 (3)	4.81	4.2391	2.8%	4.81 [-3.50, 13.12]
**Subtotal (95% CI)**			**2.8%**	**4.81 [-3.50, 13.12]**
Heterogeneity: Not applicable				
Test for overall effect: Z = 1.13 (P = 0.26)				
**Total (95% CI)**			**100.0%**	**6.03 [4.35, 7.71]**
Heterogeneity: Tau² = 8.87; Chi² = 598.58, df = 21 (P < 0.00001); I² = 96%				
Test for overall effect: Z = 7.01 (P < 0.00001)				
Test for subgroup differences: Chi² = 22.87, df = 5 (P = 0.0004), I² = 78.1%				

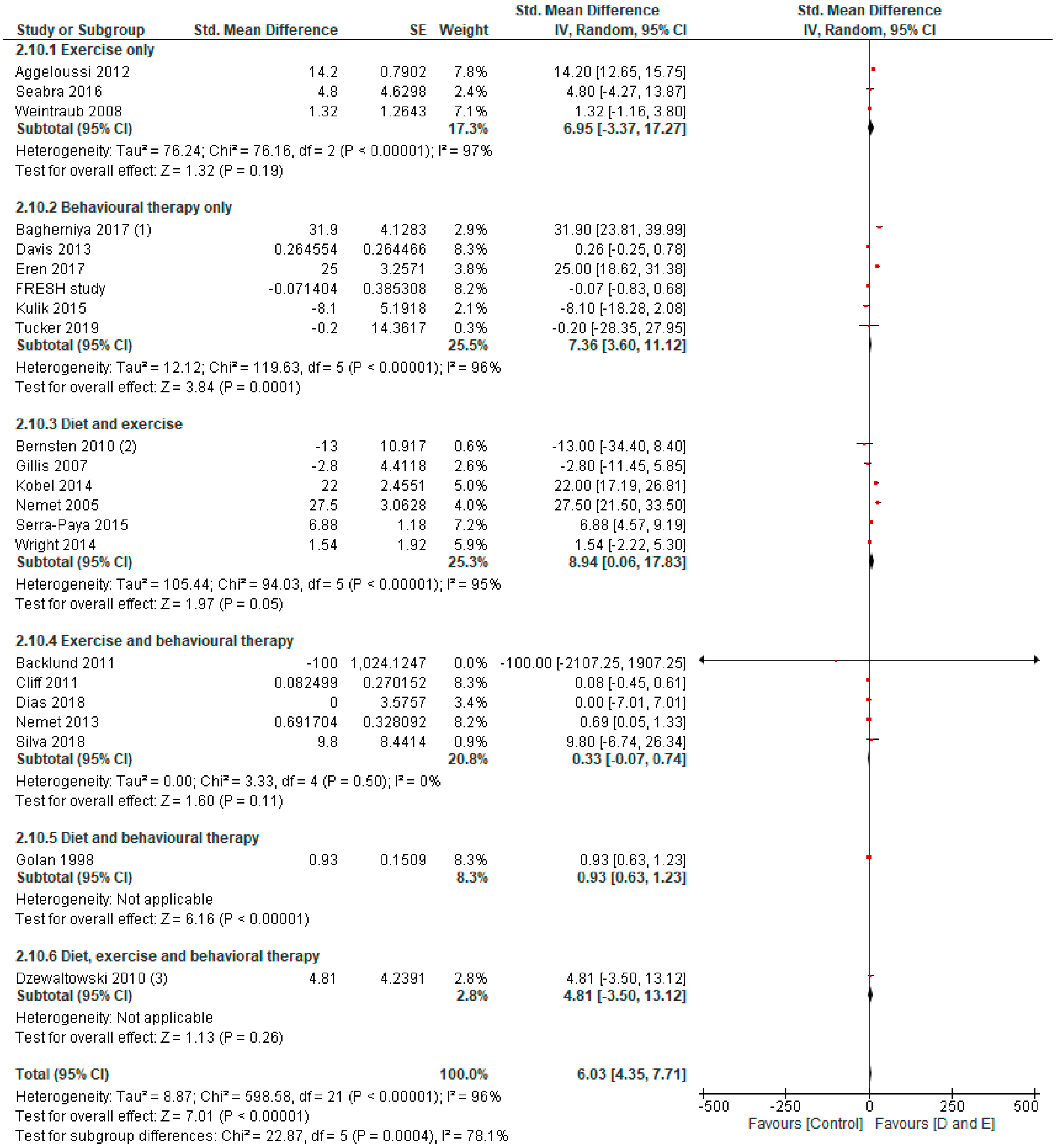

Footnotes
(1) T (BT)
(2) T (Diet and exercise)
(3) MVPA

Figure S16: Impact of obesity management interventions on total caloric consumption

Study or Subgroup	Mean Difference	SE	Experimental Total	Control Total	Weight	Mean Difference IV, Random, 95% CI
**2.11.1 Exercise only**						
Maud 2019	363.9	147.2182	22	21	2.0%	363.90 [75.36, 652.44]
Seabra 2016	-34	30.2783	29	30	12.1%	-34.00 [-93.34, 25.34]
Seo 2019	-57.1	112.3264	26	44	3.2%	-57.10 [-277.26, 163.06]
**Subtotal (95% CI)**			**77**	**95**	**17.3%**	**54.68 [-146.59, 255.94]**
Heterogeneity: Tau^2 = 22181.33; Chi^2 = 7.12, df = 2 (P = 0.03); I^2 = 72%						
Test for overall effect: Z = 0.53 (P = 0.59)						
**2.11.2 Diet only**						
Kong 2014	-416.6	156.7356	34	27	1.8%	-416.60 [-723.80, -109.40]
Mendes 2016	-15.8	121.9596	25	19	2.8%	-15.80 [-254.84, 223.24]
**Subtotal (95% CI)**			**59**	**46**	**4.6%**	**-204.11 [-596.17, 187.95]**
Heterogeneity: Tau^2 = 60600.22; Chi^2 = 4.07, df = 1 (P = 0.04); I^2 = 75%						
Test for overall effect: Z = 1.02 (P = 0.31)						
**2.11.3 Behavioural therapy only**						
Bagherniya 2017 (1)	-143.2	60.4503	73	81	7.3%	-143.20 [-261.68, -24.72]
Baranowski 2003 (2)	-231	53.287	17	14	8.3%	-231.00 [-335.44, -126.56]
Barkin 2018 (3)	-96	32.4566	278	272	11.7%	-96.00 [-159.61, -32.39]
Davis 2013	-78.58	126.415514	31	27	2.6%	-78.58 [-326.35, 169.19]
FRESH study	-17.23	135.126434	28	24	2.3%	-17.23 [-282.07, 247.61]
Hystad 2013	-7	309.3854	32	42	0.5%	-7.00 [-613.38, 599.38]
**Subtotal (95% CI)**			**459**	**460**	**32.8%**	**-131.58 [-188.16, -75.01]**
Heterogeneity: Tau^2 = 711.64; Chi^2 = 5.75, df = 5 (P = 0.33); I^2 = 13%						
Test for overall effect: Z = 4.56 (P < 0.00001)						
**2.11.4 Diet and exercise**						
Cohen 2016 (4)	-303	202.9449	10	7	1.1%	-303.00 [-700.76, 94.76]
Janicke 2008 (5)	60	256.766	24	21	0.7%	60.00 [-443.25, 563.25]
Nemet 2005	1,828	774.9	20	20	0.1%	1828.00 [309.22, 3346.78]
O'Connor 2013	-21.7	117.9911	18	16	2.9%	-21.70 [-252.96, 209.56]
**Subtotal (95% CI)**			**72**	**64**	**4.9%**	**11.86 [-360.23, 383.95]**
Heterogeneity: Tau^2 = 77769.75; Chi^2 = 7.66, df = 3 (P = 0.05); I^2 = 61%						
Test for overall effect: Z = 0.06 (P = 0.95)						
**2.11.5 Diet and behavioural therapy**						
Albala 2008 (6)	-100.7	6.6374	44	46	15.4%	-100.70 [-113.71, -87.69]
Garipağaoğlu 2008	-99	74.914	39	37	5.7%	-99.00 [-245.83, 47.83]
Mirza 2013	2	31.108	27	24	12.0%	2.00 [-58.97, 62.97]
Moschonis 2019	-138.3	110.86591	35	30	3.2%	-138.30 [-355.59, 78.99]
**Subtotal (95% CI)**			**145**	**137**	**36.3%**	**-69.75 [-141.31, 1.82]**
Heterogeneity: Tau^2 = 3064.48; Chi^2 = 10.57, df = 3 (P = 0.01); I^2 = 72%						
Test for overall effect: Z = 1.91 (P = 0.06)						
**2.11.6 Exercise and behavioral therapy**						
Md Yusop 2018	-2.2	96.279	20	20	4.0%	-2.20 [-190.90, 186.50]
**Subtotal (95% CI)**			**20**	**20**	**4.0%**	**-2.20 [-190.90, 186.50]**
Heterogeneity: Not applicable						
Test for overall effect: Z = 0.02 (P = 0.98)						
**Total (95% CI)**			**832**	**822**	**100.0%**	**-77.56 [-121.60, -33.52]**
Heterogeneity: Tau^2 = 3244.68; Chi^2 = 45.60, df = 19 (P = 0.0006); I^2 = 58%						
Test for overall effect: Z = 3.45 (P = 0.0006)						
Test for subgroup differences: Chi^2 = 5.75, df = 5 (P = 0.33), I^2 = 13.1%						

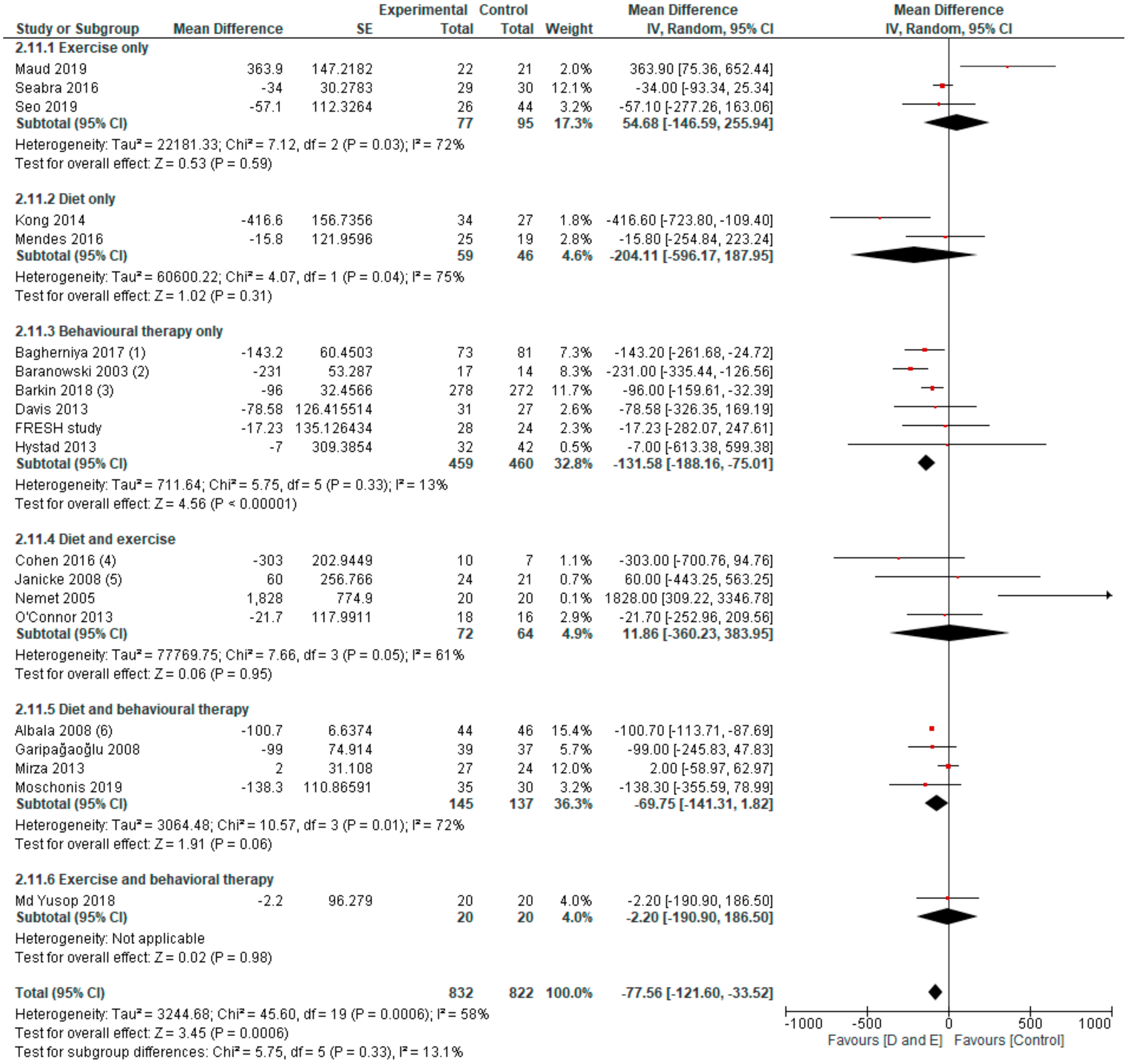

Footnotes
(1) T (CT)
(2) P
(3) P
(4) Standard Intrervention: 2 servings milk
(5) Family Based Condition
(6) T

References:

1. Alexander, A.G., et al., *A prospective multifactorial intervention on subpopulations of predominately hispanic children at high risk for obesity.* Obesity, 2014. **22**(1): p. 249-253.
2. Arlinghaus, K.R., D.P. O'Connor, and C.A. Johnston, *Frequency of school-based intervention needed to improve weight outcomes of Mexican-American adolescents with overweight or obesity: a randomized controlled trial.* Pediatric Obesity, 2019. **14**(12).
3. Bartelink, N.H., et al., *Long-term effects of the RealFit intervention on body composition, aerobic fitness, and behavior.* Childhood obesity, 2014. **10**(5): p. 383-391.
4. Bean, M.K., et al., *Motivational interviewing targeting diet and physical activity improves adherence to paediatric obesity treatment: results from the MI Values randomized controlled trial.* Pediatr Obes, 2015. **10**(2): p. 118-25.
5. Bean, M.K., et al., *A values-based Motivational Interviewing (MI) intervention for pediatric obesity: study design and methods for MI Values.* Contemporary Clinical Trials, 2011. **32**(5): p. 667-74.
6. Bean, M.K., et al., *Six-month dietary changes in ethnically diverse, obese adolescents participating in a multidisciplinary weight management program.* Clinical Pediatrics, 2011. **50**(5): p. 408-16.
7. Bocca, G., et al., *Results of a multidisciplinary treatment program in 3-year-old to 5-year-old overweight or obese children: a randomized controlled clinical trial.* Archives of Pediatrics & Adolescent Medicine, 2012. **166**(12): p. 1109-1115.
8. Bocca, G., et al., *Effect of obesity intervention programs on adipokines, insulin resistance, lipid profile, and low-grade inflammation in 3- to 5-y-old children.* Pediatric Research, 2014. **75**(2): p. 352-357.
9. Bocca, G., et al., *Three-year follow-up of 3-year-old to 5-year-old children after participation in a multidisciplinary or a usual-care obesity treatment program.* Clinical Nutrition. **33**(6): p. 1095-100.
10. Bocca, G., et al., *A multidisciplinary intervention programme has positive effects on quality of life in overweight and obese preschool children.* Acta Paediatrica. **103**(9): p. 962-7.
11. Bocca, G., et al., *Effect of a multidisciplinary treatment program on eating behavior in overweight and obese preschool children.* Journal of Pediatric Endocrinology & Metabolism. **31**(5): p. 507-513.
12. Boutelle, K.N., et al., *An intervention based on Schachter's externality theory for overweight children: the regulation of cues pilot.* Journal of pediatric psychology, 2014. **39**(4): p. 405-417.
13. Boutelle, K.N., et al., *A pilot study evaluating a one-session attention modification training to decrease overeating in obese children.* Appetite, 2014. **76**: p. 180-185.
14. Braet, C., M.v. Winckel, and K.v. Leeuwen, *Follow-up results of different treatment programs for obese children.* Acta Paediatrica, 1997. **86**(4): p. 397-402.
15. Burrows, T., et al., *Dietary outcomes of the healthy dads healthy kids randomised controlled trial.* Journal of pediatric gastroenterology and nutrition, 2012. **55**(4): p. 408-411.
16. Collins, C., et al., *The association between father-child dietary intakes: Results from the Healthy Dads, Healthy Kids community randomised controlled trial.* Obesity Research and Clinical Practice, 2012. **6**: p. 80.
17. Griffin, T., et al., *Healthy Dads, Healthy Kids UK, a weight management programme for fathers: Feasibility RCT.* BMJ Open, 2019. **9**(12).
18. Lloyd, A.B., et al., *Impact of the 'Healthy Dads, Healthy Kids' lifestyle programme on the activity- and diet-related parenting practices of fathers and mothers.* Pediatric Obesity, 2014. **9**(6): p. e149-e155.
19. Lloyd, A.B., et al., *Paternal Lifestyle-Related Parenting Practices Mediate Changes in Children's Dietary and Physical Activity Behaviors: findings From the Healthy Dads, Healthy Kids Community Randomized Controlled Trial.* Journal of physical activity & health, 2015. **12**(9): p. 1327-1335.

20. Lloyd, A.B., et al., *Investigating the measurement and operationalisation of obesity-related parenting variables of overweight fathers in the Healthy Dads, Healthy Kids community program.* Obesity Research and Clinical Practice, 2011. **5**: p. S72.
21. Morgan, P.J., et al., *Twelve-month outcomes of a father-child lifestyle intervention delivered by trained local facilitators in underserved communities: the Healthy Dads Healthy Kids dissemination trial.* Translational behavioral medicine, 2019. **9**(3): p. 560-569.
22. Morgan, P.J., et al., *The 'Healthy Dads, Healthy Kids' community randomized controlled trial: a community-based healthy lifestyle program for fathers and their children.* Preventive Medicine. **61**: p. 90-9.
23. Morgan, P.J., et al., *The 'Healthy Dads, Healthy Kids' randomized controlled trial: efficacy of a healthy lifestyle program for overweight fathers and their children.* International Journal of Obesity, 2011. **35**(3): p. 436-447.
24. Williams, A., et al., *Dietary outcomes of overweight fathers and their children in the Healthy Dads, Healthy Kids community randomised controlled trial.* Journal of Human Nutrition & Dietetics. **31**(4): p. 523-532.
25. *Lubans DR, Morgan PJ, Collins CE, Okely AD, Burrows T, Callister R. Mediators of weight loss in the'Healthy Dads, Healthy Kids' pilot study for overweight fathers. International Journal of Behavioral Nutrition and Physical Activity. 2012 Dec 1;9(1):45.*
26. Chen, J.-L., et al., *Efficacy of a child-centred and family-based program in promoting healthy weight and healthy behaviors in Chinese American children: a randomized controlled study.* Journal of Public Health, 2010. **32**(2): p. 219-229.
27. Chen, J.-L., et al., *The efficacy of the web-based childhood obesity prevention program in Chinese American adolescents (Web ABC study).* Journal of Adolescent Health, 2011. **49**(2): p. 148-154.
28. Chen, J.-L., et al., *Web-based active balance childhood study for Chinese-American adolescents.* Communicating Nursing Research, 2011. **44**: p. 263-263.
29. Johnson, S.L., et al., *A longitudinal intervention to improve young children's liking and consumption of new foods: findings from the Colorado LEAP study.* International Journal of Behavioral Nutrition & Physical Activity. **16**(1): p. 49.
30. Bellows, L.L., et al., *The Colorado LEAP study: rationale and design of a study to assess the short term longitudinal effectiveness of a preschool nutrition and physical activity program.* BMC Public Health, 2013. **13**(1): p. 1146-1146.
31. DeBar, L.L., et al., *A primary care–based, multicomponent lifestyle intervention for overweight adolescent females.* Pediatrics, 2012. **129**(3): p. e611-e620.
32. Dubuy, V., et al., *Evaluation of a real world intervention using professional football players to promote a healthy diet and physical activity in children and adolescents from a lower socio-economic background: a controlled pretest-posttest design.* BMC Public Health, 2014. **14**(1): p. 457.
33. Dunker, K.L.L. and A.M. Claudino, *Preventing weight-related problems among adolescent girls: A cluster randomized trial comparing the Brazilian 'New Moves' program versus observation.* Obesity Research and Clinical Practice, 2018. **12**(1): p. 102-115.
34. Dzewaltowski, D.A., et al., *HOP'N after-school project: An obesity prevention randomized controlled trial.* The International Journal of Behavioral Nutrition and Physical Activity Vol 7 2010, ArtID 90, 2010. **7**.
35. Elinder, L.S., et al., *A participatory and capacity-building approach to healthy eating and physical activity–SCIP-school: a 2-year controlled trial.* International Journal of Behavioral Nutrition and Physical Activity, 2012. **9**(1): p. 145.
36. Elinder, L.S., et al., *Longitudinal changes in health behaviours and body weight among Swedish school children - associations with age, gender and parental education - the SCIP school cohort.* BMC Public Health, 2014. **14**(1): p. 640-640.

37. Ellis, D.A., et al., *The effects of multisystemic therapy on family support for weight loss among obese African-American adolescents: findings from a randomized controlled trial.* Journal of Developmental & Behavioral Pediatrics, 2010. **31**(6): p. 461-468.
38. Naar-King, S., et al., *A Randomized Pilot Study of Multisystemic Therapy Targeting Obesity in African-American Adolescents.* Journal of Adolescent Health, 2009. **45**(4): p. 417-419.
39. Naar-King, S., et al., *Sequential Multiple Assignment Randomized Trial (SMART) to Construct Weight Loss Interventions for African American Adolescents.* Journal of Clinical Child & Adolescent Psychology. **45**(4): p. 428-41.
40. Berry, D.C., et al., *Rationale, design, methodology and sample characteristics for the family partners for health study: a cluster randomized controlled study.* BMC Public Health. **12**: p. 250.
41. Berry, D.C., et al., *Benefits for African American and white low-income 7-10-year-old children and their parents taught together in a community-based weight management program in the rural southeastern United States.* BMC Public Health. **18**(1): p. 1107.
42. Berry, D.C., et al., *The family partners for health study: A cluster randomized controlled trial for child and parent weight management.* Nutrition and Diabetes, 2014. **4**(JANUARY).
43. Figueroa-Colon, R., et al., *Feasibility of a clinic-based hypocaloric dietary intervention implemented in a school setting for obese children.* Obesity Research, 1996. **4**(5): p. 419-429.
44. Foster, G.D., et al., *A school-based intervention for diabetes risk reduction.* New England Journal of Medicine, 2010. **363**(5): p. 443-453.
45. Gillis, B., et al., *Rationale, design and methods of the HEALTHY study nutrition intervention component.* International Journal of Obesity, 2009. **33**(SUPPL. 4): p. S29-S36.
46. Healthy Study, G., et al., *HEALTHY study rationale, design and methods: moderating risk of type 2 diabetes in multi-ethnic middle school students.* International journal of obesity, 2009. **33 Suppl 4**: p. S4-20.
47. McMurray, R.G., et al., *Rationale, design and methods of the HEALTHY study physical education intervention component.* International Journal of Obesity, 2009. **33**(SUPPL. 4): p. S37-S43.
48. Siega-Riz, A.M., et al., *The effects of the HEALTHY study intervention on middle school student dietary intakes.* International Journal of Behavioral Nutrition & Physical Activity, 2011. **8**: p. 8p-8p.
49. Venditti, E.M., et al., *Rationale, design and methods of the HEALTHY study behavior intervention component.* International Journal of Obesity, 2009. **33**(SUPPL. 4): p. S44-S51.
50. Willi, S.M., et al., *Cardiovascular risk factors in multi-ethnic middle school students: The HEALTHY primary prevention trial.* Pediatric Obesity, 2012. **7**(3): p. 230-239.
51. Fulkerson, J.A., et al., *Healthy Home Offerings via the Mealtime Environment (HOME): feasibility, acceptability, and outcomes of a pilot study.* Obesity, 2010. **18**(S1): p. S69-S74.
52. Flattum, C., et al., *HOME Plus: Program design and implementation of a family-focused, community-based intervention to promote the frequency and healthfulness of family meals, reduce children's sedentary behavior, and prevent obesity.* International Journal of Behavioral Nutrition & Physical Activity. **12**: p. 53.
53. Fulkerson, J.A., et al., *Promoting healthful family meals to prevent obesity: HOME Plus, a randomized controlled trial.* International Journal of Behavioral Nutrition and Physical Activity, 2015. **12**(1).
54. Fulkerson, J.A., et al., *Family Home Food Environment and Nutrition-Related Parent and Child Personal and Behavioral Outcomes of the Healthy Home Offerings via the Mealtime Environment (HOME) Plus Program: a Randomized Controlled Trial.* Journal of the academy of nutrition and dietetics, 2018. **118**(2): p. 240-251.
55. Fulkerson, J.A., et al., *The Healthy Home Offerings via the Mealtime Environment (HOME) Plus study: design and methods.* Contemporary Clinical Trials. **38**(1): p. 59-68.

56. Myers, M.L., et al., *Case study: behavior changes in the family-focused obesity prevention HOME Plus program.* Public health nursing (boston, mass.), 2018. **35**(4): p. 299-306.
57. Gabriele, J.M., et al., *Development of an internet-based obesity prevention program for children.* Journal of Diabetes Science & Technology, 2010. **4**(3): p. 723-32.
58. Hawkins, K.R., et al., *Efficacy of a school-based obesity prevention intervention at reducing added sugar and sodium in children's school lunches: the LA Health randomized controlled trial.* International Journal of Obesity, 2018. **42**(11): p. 1845-1852.
59. Gallagher, K.S., et al., *Treating rural pediatric obesity through telemedicine: baseline data from a randomized controlled trial.* Journal of Pediatric Psychology, 2011. **36**(6): p. 687-695.
60. Gatti, C., et al., *Obesity and Metabolic Parameters in Adolescents: A School-Based Intervention Program in French Polynesia.* Journal of Adolescent Health, 2015. **56**(2): p. 174-180.
61. Gee, L. and W.S. Agras, *A randomized pilot study of a brief outpatient problem-solving intervention to promote healthy eating and activity habits in adolescents.* Clinical Pediatrics, 2014. **53**(3): p. 293-296.
62. Gerards, S.M., et al., *The effectiveness of lifestyle triple P in the Netherlands: a randomized controlled trial.* PLoS One, 2015. **10**(4).
63. Gerards, S.M., et al., *Lifestyle Triple P: a parenting intervention for childhood obesity.* BMC Public Health. **12**: p. 267.
64. Germann, J.N., et al., *Long-term evaluation of multi-disciplinary treatment of morbid obesity in low-income minority adolescents: La Rabida Children's Hospital's FitMatters program.* Journal of Adolescent Health, 2006. **39**(4): p. 553-561.
65. Goldfield, G., et al., *Cost-effectiveness of group and mixed family-based treatment for childhood obesity.* International Journal of Obesity, 2001. **25**(12): p. 1843-1849.
66. Golley, R.K., et al., *Twelve-month effectiveness of a parent-led, family-focused weight-management program for prepubertal children: a randomized, controlled trial.* Pediatrics, 2007. **119**(3): p. 517-525.
67. Golley, R.K., A.M. Magarey, and L.A. Daniels, *Children's food and activity patterns following a six-month child weight management program.* International Journal of Pediatric Obesity, 2011. **6**(5-6): p. 409-414.
68. Janicke, D.M., et al., *The Extension Family Lifestyle Intervention Project (E-FLIP for Kids): Design and methods.* Contemporary Clinical Trials, 2011. **32**(1): p. 50-58.
69. *Janicke, D. M., Lim, C. S., Perri, M. G., Mathews, A. E., Bobroff, L. B., Gurka, M. J., ... & Silverstein, J. H. (2019). Featured Article: Behavior Interventions Addressing Obesity in Rural Settings: The E-FLIP for Kids Trial. Journal of pediatric psychology, 44(8), 889-901.* .
70. *Graef, D. M., Janicke, D. M., & McCrae, C. S. (2014). Sleep patterns of a primarily obese sample of treatment-seeking children. Journal of Clinical Sleep Medicine, 10(10), 1111-1117.*
71. *Labyak, C. A., Janicke, D. M., Lim, C. S., Colee, J., & Mathews, A. E. (2013). Anthropometrics to identify overweight children at most risk for the development of cardiometabolic disease. ICAN: Infant, Child, & Adolescent Nutrition, 5(6), 341-346.*
72. Guo, H., et al., *Intervention of childhood and adolescents obesity in Shantou city.* Obesity Research & Clinical Practice, 2015. **9**(4): p. 357-364.
73. Haines, J., et al., *Healthy habits, happy homes: randomized trial to improve household routines for obesity prevention among preschool-aged children.* JAMA pediatrics, 2013. **167**(11): p. 1072-1079.
74. Taveras, E.M., et al., *Healthy Habits, Happy Homes: Methods and baseline data of a randomized controlled trial to improve household routines for obesity prevention.* Preventive medicine, 2012. **55**(5): p. 418-426.

75. Händel, M.N., et al., *Effects of the Healthy Start randomized intervention trial on physical activity among normal weight preschool children predisposed to overweight and obesity.* PloS one, 2017. **12**(10).
76. Olsen, N.J., et al., *Primary prevention of fat and weight gain among obesity susceptible normal weight preschool children. Results from the "Healthy Start" randomized controlled intervention.* Obesity facts, 2018. **11**: p. 192-193.
77. Olsen, N.J., et al., *Primary prevention of fat and weight gain among obesity susceptible healthy weight preschool children. Main results from the "Healthy Start" randomized controlled intervention.* Obesity facts, 2019. **12**: p. 35-36.
78. Olsen, N.J., et al., *Primary prevention of excessive fat gain among 2-6 year olds. Results from the "healthy start" randomized intervention.* Annals of nutrition & metabolism, 2017. **71**: p. 735-736.
79. Olsen, N.J., et al., *The Healthy Start project: a randomized, controlled intervention to prevent overweight among normal weight, preschool children at high risk of future overweight.* BMC Public Health, 2012. **12**(1): p. 590-590.
80. Hofsteenge, G.H., et al., *Effect of the Go4it multidisciplinary group treatment for obese adolescents on health related quality of life: a randomised controlled trial.* BMC public health, 2013. **13**(1): p. 939.
81. Hofsteenge, G.H., et al., *Go4it; study design of a randomised controlled trial and economic evaluation of a multidisciplinary group intervention for obese adolescents for prevention of diabetes mellitus type 2.* BMC Public Health, 2008. **8**: p. 410.
82. Weijs, P.J., et al., *Long-term effect of the Go4it group treatment for obese adolescents: A randomised controlled trial.* Clinical Nutrition, Supplement, 2012. **7**(1): p. 130.
83. Hughes, A.R., et al., *Randomized, controlled trial of a best-practice individualized behavioral program for treatment of childhood overweight: Scottish Childhood Overweight Treatment Trial (SCOTT).* Pediatrics, 2008. **121**(3): p. e539-e546.
84. Israel, A.C., et al., *An evaluation of enhanced self-regulation training in the treatment of childhood obesity.* Journal of pediatric psychology, 1994. **19**(6): p. 737-749.
85. Jelalian, E., et al., *Predictors of attrition and weight loss in an adolescent weight control program.* Obesity, 2008. **16**(6): p. 1318-1323.
86. *Jelalian E, Lloyd-Richardson EE, Mehlenbeck RS, Hart CN, Flynn-O'Brien K, Kaplan J, Neill M, Wing RR. Behavioral weight control treatment with supervised exercise or peer-enhanced adventure for overweight adolescents. The Journal of pediatrics. 2010 Dec 1;157(6):923-8.*
87. Jelalian, E., et al., *'Adventure therapy' combined with cognitive-behavioral treatment for overweight adolescents.* International Journal of Obesity, 2006. **30**(1): p. 31-9.
88. Jelalian, E., et al., *Two-year follow up of a behavioral adolescent weight control intervention.* Obesity, 2010. **18**: p. S104.
89. Lloyd-Richardson, E.E., et al., *Two-year follow-up of an adolescent behavioral weight control intervention.* Pediatrics. **130**(2): p. e281-8.
90. Jensen, D.E., et al., *Fasting gut hormone levels change with modest weight loss in obese adolescents.* Pediatric obesity, 2015. **10**(5): p. 380-387.
91. Johnson, W.G., et al., *Dietary and exercise interventions for juvenile obesity: long-term effect of behavioral and public health models.* Obesity Research, 1997. **5**(3): p. 257-261.
92. Kalarchian, M.A., et al., *Family-based treatment of severe pediatric obesity: randomized, controlled trial.* Pediatrics, 2009. **124**(4): p. 1060-1068.
93. Khan, N.A., et al., *Impact of the fitkids physical activity intervention on adiposity in prepubertal children.* Pediatrics, 2014. **133**(4): p. e875-e883.

94. Knowlden, A.P., et al., *Impact evaluation of enabling mothers to prevent pediatric obesity through web-based education and reciprocal determinism (EMPOWER) randomized control trial.* Health Education & Behavior, 2015. **42**(2): p. 171-184.
95. *Knowlden A, Sharma M. One-year efficacy testing of enabling mothers to prevent pediatric obesity through web-based education and reciprocal determinism (EMPOWER) randomized control trial. Health Education & Behavior. 2016 Feb;43(1):94-106.*
96. Knowlden, A.P. and E. Conrad, *Two-Year Outcomes of the Enabling Mothers to Prevent Pediatric Obesity Through Web-Based Education and Reciprocal Determinism (EMPOWER) Randomized Control Trial.* Health Education & Behavior. **45**(2): p. 262-276.
97. Lopera, C.A., et al., *Effect of water-versus land-based exercise training as a component of a multidisciplinary intervention program for overweight and obese adolescents.* Physiology & behavior, 2016. **165**: p. 365-373.
98. Maatoug, J., et al., *School-Based Intervention as a Component of a Comprehensive Community Program for Overweight and Obesity Prevention, Sousse, Tunisia, 2009-2014.* Preventing chronic disease, 2015. **12**: p. E160-E160.
99. Maatoug, J., et al., *Challenges and results of a school-based intervention to manage excess weight among school children in Tunisia 2012-2014.* International Journal of Adolescent Medicine & Health, 2017. **29**(2): p. 01.
100. MacDonell, K., et al., *A pilot study of motivational interviewing targeting weight-related behaviors in overweight or obese African American adolescents.* Journal of Adolescent Health, 2012. **50**(2): p. 201-203.
101. Magarey, A.M., et al., *A parent-led family-focused treatment program for overweight children aged 5 to 9 years: the PEACH RCT.* Pediatrics, 2011. **127**(2): p. 214-222.
102. Croyden, D.L., et al., *A narrative account of implementation lessons learnt from the dissemination of an up-scaled state-wide child obesity management program in Australia: PEACH™ (Parenting, Eating and Activity for Child Health) Queensland.* BMC Public Health, 2018. **18**: p. 1-1.
103. Williams, S.L., et al., *Parent engagement and attendance in PEACHTM QLD - an up-scaled parent-led childhood obesity program.* BMC Public Health. **17**(1): p. 559.
104. Mauriello, L.M., et al., *Results of a multi-media multiple behavior obesity prevention program for adolescents.* Preventive medicine, 2010. **51**(6): p. 451-456.
105. Moens, E. and C. Braet, *Training parents of overweight children in parenting skills: a 12-month evaluation.* Behavioural and Cognitive Psychotherapy, 2012. **40**(1): p. 1-18.
106. Moore, S.M., et al., *Two family interventions to reduce BMI in low-income urban youth: a randomized trial.* Pediatrics, 2019. **143**(6): p. e20182185.
107. Moore, S.M., et al., *IMPACT: a multi-level family and school intervention targeting obesity in urban youth.* Contemporary Clinical Trials. **36**(2): p. 574-86.
108. Nemet, D., et al., *A combined nutritional-behavioral-physical activity intervention for the treatment of childhood obesity--a 7-year summary.* Journal of Pediatric Endocrinology & Metabolism. **27**(5-6): p. 445-51.
109. Nowicka, P., et al., *Family Weight School treatment: 1-year results in obese adolescents.* International Journal of Pediatric Obesity, 2008. **3**(3): p. 141-147.
110. Nyberg, G., et al., *Effectiveness of a universal parental support programme to promote healthy dietary habits and physical activity and to prevent overweight and obesity in 6-year-old children: the healthy school start study, a cluster-randomised controlled trial.* Plos one, 2015. **10**(2).
111. Nyberg, G., et al., *A healthy school start - parental support to promote healthy dietary habits and physical activity in children: design and evaluation of a cluster-randomised intervention.* BMC Public Health, 2011. **11**(1): p. 185-185.

112. Patsopoulou, A., et al., *Evaluating the efficacy of the feeding exercise randomized trial in overweight and obese adolescents.* Childhood Obesity, 2017. **13**(2): p. 128-137.
113. Po'e, E.K., et al., *Growing Right Onto Wellness (GROW): A family-centered, community-based obesity prevention randomized controlled trial for preschool child–parent pairs.* Contemporary clinical trials, 2013. **36**(2): p. 436-449.
114. Phaitrakoon, J., et al., *Effects of an Obesity Control Program for Thai Elementary School Children: A Quasi-Experimental Study.* Pacific Rim International Journal of Nursing Research, 2014. **18**(4): p. 290-304.
115. Reinehr, T., et al., *Long-term follow-up of overweight children: after training, after a single consultation session, and without treatment.* Journal of pediatric gastroenterology and nutrition, 2003. **37**(1): p. 72-74.
116. *Reinehr T, Enriori PJ, Harz K, Cowley MA, Roth CL. Pancreatic polypeptide in obese children before and after weight loss. International journal of obesity. 2006 Oct;30(10):1476-81.*
117. *Reinehr T, De Sousa G, Roth CL. Obestatin and ghrelin levels in obese children and adolescents before and after reduction of overweight. Clinical endocrinology. 2008 Feb;68(2):304-10.*
118. *Fritsch P, Kleber M, Schlagenhauf A, Laschnik B, Fritsch M, Muntean W, Mangge H, Reinehr T. Normalization of haemostatic alterations in overweight children with weight loss due to lifestyle intervention. Atherosclerosis. 2011 May 1;216(1):170-3.*
119. *Reinehr T, Roth CL, Alexy U, Kersting M, Kiess W, Andler W. Ghrelin levels before and after reduction of overweight due to a low-fat high-carbohydrate diet in obese children and adolescents. International journal of obesity. 2005 Apr;29(4):362-8.*
120. Kolip, P., et al., *Evaluation of the "Obeldicks Light Training" Programme for Overweight Children and Adolescents.* Gesundheitswesen (bundesverband der arzte des offentlichen gesundheitsdienstes (germany)), 2015. **77 Suppl 1**: p. S56-7.
121. Reinehr, T., et al., *7-Year follow-up of a lifestyle intervention in overweight children: Comparison to an untreated control group.* Clinical Nutrition. **37**(5): p. 1558-1562.
122. Reinehr, T., et al., *Development and evaluation of the lifestyle intervention "obeldicks light" for overweight children and adolescents.* Journal of Public Health, 2011. **19**(4): p. 377-384.
123. Reinehr, T., et al., *An effective lifestyle intervention in overweight children: Findings from a randomized controlled trial on " Obeldicks light".* Clinical Nutrition, 2010. **29**(3): p. 331-336.
124. Schaefer, A., et al., *An effective lifestyle intervention in overweight children: One-year follow-up after the randomized controlled trial on " Obeldicks light".* Clinical Nutrition, 2011. **30**(5): p. 629-633.
125. Resnicow, K., et al., *Results of Go Girls: A weight control program for overweight African-American Adolescent females.* Obesity Research, 2005. **13**(10): p. 1739-1748.
126. Resnicow, K., et al., *Motivational interviewing and dietary counseling for obesity in primary care: an RCT.* Pediatrics, 2015. **135**(4): p. 649-657.
127. Robertson, W., et al., *Randomised controlled trial and economic evaluation of the 'Families for Health'programme to reduce obesity in children.* Archives of disease in childhood, 2017. **102**(5): p. 416-426.
128. Robertson, W., et al., *Randomised controlled trial evaluating the effectiveness and cost-effectiveness of 'Families for Health', a family-based childhood obesity treatment intervention delivered in a community setting for ages 6 to 11 years.* Health technology assessment (winchester, england), 2017. **21**(1): p. 1-180.
129. Robertson, W., et al., *Pilot of "Families for Health": community-based family intervention for obesity.* Archives of Disease in Childhood, 2008. **93**(11): p. 921-6.

130. Robertson, W., et al., *Evaluation of the effectiveness and cost-effectiveness of Families for Health V2 for the treatment of childhood obesity: study protocol for a randomized controlled trial.* Trials [Electronic Resource]. **14**: p. 81.
131. Rosenbaum, M., et al., *School-based intervention acutely improves insulin sensitivity and decreases inflammatory markers and body fatness in junior high school students.* Journal of Clinical Endocrinology & Metabolism, 2007. **92**(2): p. 504-8.
132. Sarvestani, R.S., et al., *Effect of dietary behaviour modification on anthropometric indices and eating behaviour in obese adolescent girls.* Journal of Advanced Nursing, 2009. **65**(8): p. 1670-1675.
133. Savoye, M., et al., *Effects of a weight management program on body composition and metabolic parameters in overweight children: a randomized controlled trial.* Jama, 2007. **297**(24): p. 2697-2704.
134. Savoye, M., et al., *Anthropometric and psychosocial changes in obese adolescents enrolled in a Weight Management Program.* Journal of the American Dietetic Association, 2005. **105**(3): p. 364-70.
135. Savoye, M., et al., *Reversal of early abnormalities in glucose metabolism in obese youth: results of an intensive lifestyle randomized controlled trial.* Diabetes Care. **37**(2): p. 317-24.
136. Savoye, M., et al., *Long-term Results of an Obesity Program in an Ethnically Diverse Pediatric Population.* Pediatrics, 2011. **127**(3): p. 402-410.
137. Sharma, S.V., et al., *Impact of the Coordinated Approach to Child Health Early Childhood Program for Obesity Prevention among Preschool Children: The Texas Childhood Obesity Research Demonstration Study.* Childhood Obesity, 2019. **15**(1): p. 1-13.
138. Shelton, D., et al., *Randomised controlled trial: A parent-based group education programme for overweight children.* Journal of paediatrics and child health, 2007. **43**(12): p. 799-805.
139. Stark, L.J., et al., *Clinic and home-based behavioral intervention for obesity in preschoolers: a randomized trial.* The Journal of pediatrics, 2018. **192**: p. 115-121. e1.
140. Stark, L.J., et al., *A pilot randomized controlled trial of a behavioral family-based intervention with and without home visits to decrease obesity in preschoolers.* Journal of Pediatric Psychology. **39**(9): p. 1001-12.
141. Stark, L.J., et al., *Learning about Activity and Understanding Nutrition for Child Health (LAUNCH): rationale, design, and implementation of a randomized clinical trial of a family-based pediatric weight management program for preschoolers.* Contemporary clinical trials, 2017. **52**: p. 10-19.
142. Stark, L.J., et al., *Maintenance Following a Randomized Trial of a Clinic and Home-based Behavioral Intervention of Obesity in Preschoolers.* Journal of pediatrics, 2019.
143. Stark, L.J., et al., *A pilot randomized controlled trial of a clinic and home-based behavioral intervention to decrease obesity in preschoolers.* Obesity, 2011. **19**(1): p. 134-41.
144. Van Allen, J., et al., *Changes in parent motivation predicts changes in body mass index z-score (zBMI) and dietary intake among preschoolers enrolled in a family-based obesity intervention.* Journal of Pediatric Psychology. **39**(9): p. 1028-37.
145. Steinberg, N., et al., *Effects of a Program for Improving Biomechanical Characteristics During Walking and Running in Children Who Are Obese.* Pediatric Physical Therapy. **29**(4): p. 330-340.
146. Story, M., et al., *An after-school obesity prevention program for African-American girls: the Minnesota GEMS pilot study.* Ethnicity & Disease, 2003. **13**(1 Suppl 1): p. S54-64.
147. Story, M., et al., *Recruitment of African-American pre-adolescent girls into an obesity prevention trial: the GEMS pilot studies.* Ethnicity & Disease, 2003. **13**(1 Suppl 1): p. S78-87.
148. Todd, M.K., et al., *Effect of a family-based intervention on electronic media use and body composition among boys aged 8—11 years: a pilot study.* Journal of Child Health Care, 2008. **12**(4): p. 344-358.

149. Vandongen, R., et al., *A controlled evaluation of a fitness and nutrition intervention program on cardiovascular health in 10- to 12-year-old children.* Preventive Medicine. **24**(1): p. 9-22.
150. Vos, R.C., et al., *The effect of family-based multidisciplinary cognitive behavioral treatment on health-related quality of life in childhood obesity.* Quality of Life Research, 2012. **21**(9): p. 1587-1594.
151. Vos, R.C., et al., *The effect of family-based multidisciplinary cognitive behavioral treatment in children with obesity: study protocol for a randomized controlled trial.* Trials [Electronic Resource], 2011. **12**: p. 110.
152. Wafa, S.W., et al., *Randomized controlled trial of a good practice approach to treatment of childhood obesity in Malaysia: Malaysian Childhood Obesity Treatment Trial (MASCOT).* International journal of pediatric obesity, 2011. **6**(sup3): p. e62-69.
153. W. W, S., et al., *The Malaysian Childhood Obesity Treatment Trial (MASCOT).* Malaysian Journal of Nutrition, 2011. **17**(2): p. 229-236.
154. Weber, K.S., et al., *Positive Effects of Promoting Physical Activity and Balanced Diets in a Primary School Setting with a High Proportion of Migrant School Children.* Experimental and Clinical Endocrinology and Diabetes, 2017. **125**(8): p. 554-562.
155. Wesnigk, J., et al., *Impact of Lifestyle Intervention on HDL-Induced eNOS Activation and Cholesterol Efflux Capacity in Obese Adolescent.* Cardiology research and practice, 2016. **2016**.
156. West, F., et al., *Randomised clinical trial of a family-based lifestyle intervention for childhood obesity involving parents as the exclusive agents of change.* Behaviour research and therapy, 2010. **48**(12): p. 1170-1179.
157. Wilfley, D.E., et al., *Efficacy of maintenance treatment approaches for childhood overweight: a randomized controlled trial.* Jama, 2007. **298**(14): p. 1661-1673.
158. *Goldschmidt AB, Sinton MM, Aspen VP, Tibbs TL, Stein RI, Saelens BE, Frankel F, Epstein LH, Wilfley DE. Psychosocial and familial impairment among overweight youth with social problems. International Journal of Pediatric Obesity. 2010 Oct 1;5(5):428-35.*
159. Goldschmidt, A.B., et al., *Predictors of child weight loss and maintenance among family-based treatment completers.* Journal of Consulting & Clinical Psychology. **82**(6): p. 1140-1150.
160. Wilfley, D.E., et al., *Dose, content, and mediators of family-based treatment for childhood obesity a multisite randomized clinical trial.* JAMA Pediatrics, 2017. **171**(12): p. 1151-1159.
161. Williams, N.A., et al., *Risk factors for poor attendance in a family-based pediatric obesity intervention program for young children.* Journal of Developmental & Behavioral Pediatrics, 2010. **31**(9): p. 705-712.
162. Williamson, D.A., et al., *Wise Mind project: a school-based environmental approach for preventing weight gain in children.* Obesity, 2007. **15**(4): p. 906-917.
163. Wright, J.A., et al., *Randomized trial of a family-based, automated, conversational obesity treatment program for underserved populations.* Obesity, 2013. **21**(9): p. E369-E378.
164. Østbye, T., et al., *Parent-focused change to prevent obesity in preschoolers: results from the KAN-DO study.* Preventive medicine, 2012. **55**(3): p. 188-195.
165. Ostbye, T., et al., *Kids and adults now! Defeat Obesity (KAN-DO): rationale, design and baseline characteristics.* Contemporary Clinical Trials, 2011. **32**(3): p. 461-9.
166. Amaro, S., et al., *Kaledo, a new educational board-game, gives nutritional rudiments and encourages healthy eating in children: a pilot cluster randomized trial.* Eur J Pediatr, 2006. **165**(9): p. 630-5.
167. Amini, M., et al., *A school-based intervention to reduce excess weight in overweight and obese primary school children.* Annals of nutrition and metabolism. Conference: 12th european nutrition conference, FENS 2015. Berlin germany. Conference start: 20151020. Conference end: 20151023. Conference publication: (var.pagings), 2015. **67**: p. 409.

168. Anderson, Y.C., et al., *The effect of a multi-disciplinary obesity intervention compared to usual practice in those ready to make lifestyle changes: Design and rationale of Whanau Pakari.* BMC Obesity, 2015. **2**(1).
169. Anderson, Y.C., et al., *Economic evaluation of a multi-disciplinary community-based intervention programme for New Zealand children and adolescents with obesity.* Obesity Research & Clinical Practice, 2018. **12**(3): p. 293-298.
170. Annesi, J.J., A.E. Smith, and G.A. Tennant, *Reducing high BMI in African American preschoolers: effects of a behavior-based physical activity intervention on caloric expenditure.* Southern medical journal, 2013. **106**(8): p. 456-459.
171. Annesi, J.J., A.E. Smith, and G.A. Tennant, *Effects of a cognitive-behaviorally based physical activity treatment for 4- and 5-year-old children attending US preschools.* International Journal of Behavioral Medicine. **20**(4): p. 562-6.
172. Annesi, J.J., et al., *Effects of an after-school care-administered physical activity and nutrition protocol on body mass index, fitness levels, and targeted psychological factors in 5- to 8-year-olds.* Translational behavioral medicine, 2015. **6**(3): p. 347-57.
173. Annesi, J.J., et al., *Effects of the Youth Fit 4 Life physical activity/nutrition protocol on body mass index, fitness and targeted social cognitive theory variables in 9- to 12-year-olds during after-school care.* Journal of Paediatrics & Child Health, 2017. **53**(4): p. 365-373.
174. Bäcklund, C., G. Sundelin, and C. Larsson, *Effects of a 2-year lifestyle intervention on physical activity in overweight and obese children.* Advances in Physiotherapy, 2011. **13**(3): p. 97-109.
175. Bäcklund, C., G. Sundelin, and C. Larsson, *Effect of a 1-year lifestyle intervention on physical activity in overweight and obese children.* Advances in Physiotherapy, 2011. **13**(3): p. 87-96.
176. Aparecida Alves Bianchini, J., et al., *Obese adolescents who gained/maintained or lost weight had similar body composition and cardiometabolic risk factors following a multidisciplinary intervention.* Journal of Exercise Science & Fitness, 2014. **12**(1): p. 38-45.
177. Butte, N.F., et al., *Efficacy of a Community- Versus Primary Care–Centered Program for Childhood Obesity: TX CORD RCT.* Obesity, 2017. **25**(9): p. 1584-1593.
178. Bryant, M., et al., *Results of a feasibility randomised controlled trial (RCT) for WATCH IT: a programme for obese children and adolescents.* Clinical Trials, 2011. **8**(6): p. 755-764.
179. Chae, H.W., et al., *Effects of a structured exercise program on insulin resistance, inflammatory markers and physical fitness in obese Korean children.* Journal of Pediatric Endocrinology & Metabolism. **23**(10): p. 1065-72.
180. Cliff, D.P., et al., *Movement Skills and Physical Activity in Obese Children: Randomized Controlled Trial.* Medicine & Science in Sports & Exercise, 2011. **43**(1): p. 90-100.
181. Cong, Z., et al., *Sedentary behaviors among Hispanic children: influences of parental support in a school intervention program.* American Journal of Health Promotion. **26**(5): p. 270-80.
182. Ardic, A., et al., *The effectiveness of the COPE healthy lifestyles TEEN program in overweight and obese adolescents: randomized controlled study.* Obesity facts, 2019. **12**: p. 113-114.
183. Ardic, A. and S. Erdogan, *The effectiveness of the COPE healthy lifestyles TEEN program: a school-based intervention in middle school adolescents with 12-month follow-up.* Journal of Advanced Nursing. **73**(6): p. 1377-1389.
184. Melnyk, B.M., et al., *The COPE Healthy Lifestyles TEEN program: feasibility, preliminary efficacy, & lessons learned from an after school group intervention with overweight adolescents.* Journal of Pediatric Health Care, 2007. **21**(5): p. 315-22.
185. *Davis JN, Gyllenhammer LE, Vanni AA, Meija M, Tung A, Schroeder ET, Spruijt-Metz D, Goran MI. Startup circuit training program reduces metabolic risk in Latino adolescents. Medicine and science in sports and exercise. 2011 Nov;43(11):2195.*

186. de Heer, H.D., et al., *Effectiveness and Spillover of an After-School Health Promotion Program for Hispanic Elementary School Children.* American Journal of Public Health, 2011. **101**(10): p. 1907-1913.
187. Del Rio, N.G., et al., *Effects of a Gamified Educational Program in the Nutrition of Children with Obesity.* Journal of Medical Systems. **43**(7): p. 198.
188. Dias, K.A., et al., *Effect of High-Intensity Interval Training on Fitness, Fat Mass and Cardiometabolic Biomarkers in Children with Obesity: a Randomised Controlled Trial.* Sports medicine (auckland, N.Z.), 2018. **48**(3): p. 733-746.
189. Dias, K.A., et al., *Effects of exercise intensity and nutrition advice on myocardial function in obese children and adolescents: a multicentre randomised controlled trial study protocol.* BMJ open, 2016. **6**(4) (no pagination).
190. Eliakim, A., et al., *The effects of nutritional-physical activity school-based intervention on fatness and fitness in preschool children.* Journal of Pediatric Endocrinology and Metabolism, 2007. **20**(6): p. 711-718.
191. Myers, E.F., et al., *Energy balance for kids with play: design and implementation of a multi-component school-based obesity prevention program.* Childhood Obesity. **10**(3): p. 251-9.
192. *Madsen, K., Linchey, J., Gerstein, D., Ross, M., Myers, E., Brown, K., & Crawford, P. (2015). Energy balance 4 kids with play: results from a two-year cluster-randomized trial. Childhood Obesity, 11(4), 375-383.*
193. Farah, B.Q., et al., *Does exercise intensity affect blood pressure and heart rate in obese adolescents? A 6-month multidisciplinary randomized intervention study.* Pediatric Obesity, 2014. **9**(2): p. 111-120.
194. Farpour-Lambert, N.J., et al., *Effectiveness of individual and group programmes to treat obesity and reduce cardiovascular disease risk factors in pre-pubertal children.* Clinical Obesity, 2019. **9**(6).
195. Fetter, D.S., et al., *Effect of the Shaping Healthy Choices Program, a Multicomponent, School-Based Nutrition Intervention, on Physical Activity Intensity.* Journal of the American College of Nutrition. **37**(6): p. 472-478.
196. Gutin, B., et al., *Preliminary findings of the effect of a 3-year after-school physical activity intervention on fitness and body fat: the Medical College of Georgia Fitkid Project.* International Journal of Pediatric Obesity, 2008. **3**: p. 3-9.
197. Yin, Z., et al., *An environmental approach to obesity prevention in children: Medical College of Georgia FitKid Project year 1 results.* Obesity Research, 2005. **13**(12): p. 2153-61.
198. Yin, Z., et al., *An after-school physical activity program for obesity prevention in children: the Medical College of Georgia FitKid Project.* 2005. **1**(1): p. 67-89.
199. Yin, Z., et al., *The impact of a 3-year after-school obesity prevention program in elementary school children.* Childhood Obesity. **8**(1): p. 60-70.
200. Freira, S., et al., *Quality-of-life outcomes of a weight management program for adolescents based on motivational interviewing.* Patient Education & Counseling. **102**(4): p. 718-725.
201. Freitas, C.R.M., et al., *Effects of a psychological intervention on the quality of life of obese adolescents under a multidisciplinary treatment.* Jornal de pediatria. (no pagination), 2016, 2016. **Date of Publication: January 27**.
202. Carlone Baldino Garcia, N., et al., *Multidisciplinary obesity treatment program improved health-related quality of life and positively correlated with anthropometric and body composition but not with cardiorespiratory fitness parameters in adolescents.* Quality of Life Research. **28**(7): p. 1803-1812.
203. Glabska, D., et al., *The National After-School Athletics Program Participation as a Tool to Reduce the Risk of Obesity in Adolescents after One Year of Intervention: A Nationwide Study.* International Journal of Environmental Research & Public Health [Electronic Resource], 2019. **16**(3): p. 31.

204. Gortmaker, S.L., et al., *Effect of an After-School Intervention on Increases in Children's Physical Activity.* Medicine & Science in Sports & Exercise, 2012. **44**(3): p. 450-457.
205. Graf, C., et al., *School-based prevention: effects on obesity and physical performance after 4 years.* Journal of Sports Sciences, 2008. **26**(10): p. 987-994.
206. Greening, L., et al., *Efficacy of a school-based childhood obesity intervention program in a rural southern community: TEAM Mississippi Project.* Obesity (19307381), 2011. **19**(6): p. 1213-1219.
207. Grillich, L., et al., *Effectiveness evaluation of a health promotion programme in primary schools: a cluster randomised controlled trial.* BMC Public Health. **16**: p. 679.
208. Akgul Gundogdu, N., E.U. Sevig, and N. Guler, *The effect of the solution-focused approach on nutrition-exercise attitudes and behaviours of overweight and obese adolescents: Randomised controlled trial.* Journal of Clinical Nursing. **27**(7-8): p. e1660-e1672.
209. *Heino, M. T., Knittle, K., Fried, E., Sund, R., Haukkala, A., Borodulin, K., ... & Hankonen, N. (2019). Visualisation and network analysis of physical activity and its determinants: Demonstrating opportunities in analysing baseline associations in the Let's Move It trial. Health Psychology and Behavioral Medicine, 7(1), 269-289.*
210. Hankonen, N., et al., *'Let's Move It' - a school-based multilevel intervention to increase physical activity and reduce sedentary behaviour among older adolescents in vocational secondary schools: a study protocol for a cluster-randomised trial.* BMC public health, 2016. **16**: p. 451.
211. *Hankonen, N., Heino, M. T. J., Araujo-Soares, V., Absetz, P., Sniehotta, F. F., & Haukkala, A. (2016). Using theory and evidence to increase physical activity: Let's Move It school-based multi-level intervention. European Health Psychologist, 18(S), 575.*
212. Heo, M., et al., *Effective nationwide school-based participatory extramural program on adolescent body mass index, health knowledge and behaviors.* BMC Pediatrics, 2018. **18**(1).
213. Herget, S., et al., *High-intensity interval training for overweight adolescents: program acceptance of a media supported intervention and changes in body composition.* International journal of environmental research and public health, 2016. **13**(11) (no pagination).
214. Hoffman, J., et al., *An Integrated Clinic-Community Partnership for Child Obesity Treatment: a Randomized Pilot Trial.* Pediatrics, 2018. **141**(1).
215. Hrafnkelsson, H., et al., *Result of school-based intervention on cardiovascular risk factors.* Scandinavian Journal of Primary Health Care, 2014. **32**(4): p. 149-155.
216. Ickovics, J.R., et al., *Implementing School-Based Policies to Prevent Obesity: cluster Randomized Trial.* American journal of preventive medicine, 2019. **56**(1): p. e1-e11.
217. Kain, J., et al., *Two-year controlled effectiveness trial of a school-based intervention to prevent obesity in Chilean children.* Public Health Nutrition. **12**(9): p. 1451-61.
218. Kalantari, N., et al., *Indicator for success of obesity reduction programs in adolescents: Body composition or body mass index? evaluating a school-based health promotion project after 12 weeks of intervention.* International Journal of Preventive Medicine, 2017. **8**: p. 128-132.
219. Karczewski, S.A., J.S. Carter, and D.D. DeCator, *The Role of Ethnicity in School-Based Obesity Intervention for School-Aged Children: A Pilot Evaluation.* Journal of School Health, 2016. **86**(11): p. 778-786.
220. Li, B., et al., *The CHIRPY DRAGON intervention in preventing obesity in Chinese primaryschool-aged children: A cluster-randomised controlled trial.* PLoS Medicine, 2019. **16**(11).
221. Li, B., et al., *Cluster-randomised controlled trial to assess the effectiveness and cost-effectiveness of an obesity prevention programme for Chinese primary school-aged children: the CHIRPY DRAGON study protocol.* BMJ Open. **7**(11): p. e018415.
222. Madsen, K., et al., *School-community partnerships: a cluster-randomized trial of an after-school soccer program.* JAMA Pediatrics, 2013. **167**(4): p. 321-326.

223. Magnusson, K.T., et al., *Limited effects of a 2-year school-based physical activity intervention on body composition and cardiorespiratory fitness in 7-year-old children.* Health Education Research. **27**(3): p. 484-94.
224. Melnyk, B.M., et al., *Promoting healthy lifestyles in high school adolescents: a randomized controlled trial.* American Journal of Preventive Medicine. **45**(4): p. 407-15.
225. Kriemler, S., et al., *Effect of school based physical activity programme (KISS) on fitness and adiposity in primary schoolchildren: cluster randomised controlled trial.* BMJ, 2010. **340**: p. c785.
226. Meyer, U., et al., *Long-term effect of a school-based physical activity program (KISS) on fitness and adiposity in children: a cluster-randomized controlled trial.* PLoS ONE [Electronic Resource]. **9**(2): p. e87929.
227. Zahner, L., et al., *A school-based physical activity program to improve health and fitness in children aged 6-13 years ("Kinder-Sportstudie KISS"): study design of a randomized controlled trial [ISRCTN15360785].* BMC Public Health, 2006. **6**: p. 147.
228. Nayak, B.S. and V.H. Bhat, *School based multicomponent intervention for obese children in Udupi district, South India - A randomized controlled trial.* Journal of clinical and diagnostic research, 2016. **10**(12): p. SC24-SC28.
229. Nemet, D., et al., *Health promotion intervention in low socioeconomic kindergarten children.* Journal of Pediatrics, 2011. **158**(5): p. 796-801.e1.
230. Nemet, D., et al., *Long term effects of a health promotion intervention in low socioeconomic Arab-Israeli kindergartens.* BMC Pediatrics, 2013. **13**(1).
231. Nemet, D., et al., *Effects of a combined intervention for treating severely obese prepubertal children.* Journal of Pediatric Endocrinology & Metabolism. **26**(1-2): p. 91-6.
232. Huang, T., et al., *Effects of an obesity intervention program on cognitive function in children: A randomized controlled trial.* Obesity. **23**(10): p. 2101-8.
233. Larsen, K.T., et al., *Effectiveness of a one-year multi-component day-camp intervention for overweight children: study protocol of the Odense overweight intervention study (OOIS).* BMC Public Health, 2014. **14**: p. 313.
234. Larsen, K.T., et al., *Cost-effectiveness of a day-camp weight-loss intervention programme for children: Results based on a randomised controlled trial with one-year follow-up.* Scandinavian Journal of Public Health. **45**(6): p. 666-674.
235. Larsen, K.T., et al., *A Multi-Component Day-Camp Weight-Loss Program Is Effective in Reducing BMI in Children after One Year: A Randomized Controlled Trial.* PLoS ONE [Electronic Resource]. **11**(6): p. e0157182.
236. Pablos, A., et al., *Effectiveness of a school-based program focusing on diet and health habits taught through physical exercise.* Applied Physiology, Nutrition, & Metabolism = Physiologie Appliquee, Nutrition et Metabolisme. **43**(4): p. 331-337.
237. Pbert, L., et al., *A School-Based Program for Overweight and Obese Adolescents: A Randomized Controlled Trial.* Journal of School Health. **86**(10): p. 699-708.
238. Plavsic, L., et al., *Effects of high-intensity interval training and nutrition advice on cardiometabolic markers and aerobic fitness in adolescent girls with obesity.* Physiologie appliquee, nutrition et metabolisme [Applied physiology, nutrition, and metabolism], 2019.
239. Reilly, J.J., et al., *Physical activity to prevent obesity in young children: cluster randomised controlled trial.* BMJ, 2006. **333**(7577): p. 1041.
240. Saraf, D.S., et al., *Effectiveness of a school based intervention for prevention of non-communicable diseases in middle school children of rural North India: a randomized controlled trial.* Indian J Pediatr, 2015. **82**(4): p. 354-62.

241. Sepúlveda, A.R., et al., *Feasibility, acceptability, and effectiveness of a multidisciplinary intervention in childhood obesity from primary care: Nutrition, physical activity, emotional regulation, and family.* European Eating Disorders Review, 2019.
242. Sgambato, M.R., et al., *Effectiveness of school-home intervention for adolescent obesity prevention: Parallel school-randomized study.* British journal of nutrition, 2019.
243. Shaw, M., et al., *Effect of a successful intensive lifestyle program on insulin sensitivity and glucose tolerance in obese youth.* Diabetes Care, 2009. **32**(1): p. 45-47.
244. Daley, A.J., et al., *Exercise therapy as a treatment for psychopathologic conditions in obese and morbidly obese adolescents: a randomized, controlled trial.* Pediatrics, 2006. **118**(5): p. 2126-2134.
245. Daley, A.J., et al., *Protocol for: Sheffield Obesity Trial (SHOT): a randomised controlled trial of exercise therapy and mental health outcomes in obese adolescents [ISRCNT83888112].* BMC Public Health, 2005. **5**: p. 113.
246. Siegel, R.M., et al., *Obese children in a community YMCA "Fun 2B Fit" program have a reduction in BMI Z-scores.* Clinical Pediatrics. **53**(7): p. 698-700.
247. Siegrist, M., et al., *Effects of a physical education program on physical activity, fitness, and health in children: the JuvenTUM project.* Scandinavian Journal of Medicine & Science in Sports. **23**(3): p. 323-30.
248. Silva, D.R., et al., *Impact of a classroom standing desk intervention on daily objectively measured sedentary behavior and physical activity in youth.* Journal of Science & Medicine in Sport, 2018. **21**(9): p. 919-924.
249. *Simons M, Brug J, Chinapaw MJM, de Boer M, Seidell J, de Vet E (2015) Replacing Non-Active Video Gaming by Active Video Gaming to Prevent Excessive Weight Gain in Adolescents. PLoS ONE 10(7): e0126023. doi:10.1371/journal.pone.0126023.*
250. Simons, M., et al., *Active video games as a tool to prevent excessive weight gain in adolescents: rationale, design and methods of a randomized controlled trial.* BMC Public Health, 2014. **14**: p. 275.
251. Singh, A.S., et al., *Dutch obesity intervention in teenagers: effectiveness of a school-based program on body composition and behavior.* Archives of Pediatrics & Adolescent Medicine, 2009. **163**(4): p. 309-317.
252. Singh, A.S., et al., *Short-term effects of school-based weight gain prevention among adolescents.* Archives of Pediatrics & Adolescent Medicine, 2007. **161**(6): p. 565-571.
253. Slusser, W.M., et al., *Improving Overweight among At-risk Minority Youth: Results of a Pilot Intervention in After-school Programs.* Journal of Health Care for the Poor & Underserved, 2013. **24**(2, Supp): p. 12-24.
254. Sousa, P., et al., *Preventing adolescent obesity through an interdisciplinary game-based mhealth system (TeenPower).* Obesity Facts, 2019. **12**: p. 110.
255. *Sousa, P., Frontini, R., Carvalho, M., & Alves, R. (2018). Teenpower: An interdisciplinary game-based mHealth system to empower adolescents towards obesity prevention. In Proceedings of Academics World International Conference.*
256. *Sousa, P., Duarte, E., Ferreira, R., Esperança, A., Frontini, R., Santos-Rocha, R., ... & Marques, N. (2019). An mH ealth intervention programme to promote healthy behaviours and prevent adolescent obesity (TeenPower): A study protocol. Journal of advanced nursing, 75(3), 683-691. (Protocol).*
257. Spencer, R.A., et al., *Peer mentoring is associated with positive change in physical activity and aerobic fitness of grades 4, 5, and 6 students in the heart healthy kids program.* Health Promotion Practice. **15**(6): p. 803-11.
258. Speroni, K.G., C. Earley, and M. Atherton, *Evaluating the effectiveness of the KIDS LIVING FIT program: a comparative study.* Journal of School Nursing, 2007. **23**(6): p. 329-336.

259. Vajda, I., et al., *Effects of 3 hours a week of physical activity on body fat and cardio-respiratory parameters in obese boys.* Acta Physiologica Hungarica, 2007. **94**(3): p. 191-8.
260. Leeuwen, J.v., et al., *The effect of a multidisciplinary intervention program for overweight and obese children on cardiorespiratory fitness and blood pressure.* Family Practice, 2019. **36**(2): p. 147-153.
261. Lehtinen-Jacks, S., et al., *Healthier diets for families with toddlers: A cluster randomized pilot trial in Finnish child health clinics.* Obesity Facts, 2018. **11**: p. 54-55.
262. Viggiano, A., et al., *Kaledo, a board game for nutrition education of children and adolescents at school: cluster randomized controlled trial of healthy lifestyle promotion.* European journal of pediatrics, 2015. **174**(2): p. 217-228.
263. Viggiano, E., et al., *Healthy lifestyle promotion in primary schools through the board game Kaledo: a pilot cluster randomized trial.* European journal of pediatrics, 2018. **177**(9): p. 1371-1375.
264. Wang, Z., et al., *Childhood obesity prevention through a community-based cluster randomized controlled physical activity intervention among schools in china: the health legacy project of the 2nd world summer youth olympic Games (YOG-Obesity study).* International journal of obesity, 2018. **42**(4): p. 625-633.
265. Xu, F., et al., *Policy-oriented, school-based physical activity intervention to prevent childhood obesity in China (the health legacy project of the second Summer Youth Olympic Games): A cluster randomised trial.* The Lancet Diabetes and Endocrinology, 2016. **4**(SPEC. ISSUE 3): p. S9.
266. Adab, P., et al., *The West Midlands ActiVe lifestyle and healthy Eating in School children (WAVES) study: a cluster randomised controlled trial testing the clinical effectiveness and cost-effectiveness of a multifaceted obesity prevention intervention programme targeted at children aged 6-7 years.* Health Technology Assessment (Winchester, England). **22**(8): p. 1-608.
267. Adab, P., et al., *Effectiveness of a childhood obesity prevention programme delivered through schools, targeting 6 and 7 year olds: Cluster randomised controlled trial (WAVES study).* BMJ (Online), 2018. **360**.
268. Adab, P., et al., *A cluster-randomised controlled trial to assess the effectiveness and cost-effectiveness of a childhood obesity prevention programme delivered through schools, targeting 6-7 year old children: the WAVES study protocol.* BMC public health, 2015. **15**: p. 488.
269. Griffin, T.L., et al., *Process evaluation results of a cluster randomised controlled childhood obesity prevention trial: the WAVES study.* BMC Public Health, 2017. **17**: p. 1-13.
270. Griffin, T.L., et al., *Process evaluation design in a cluster randomised controlled childhood obesity prevention trial: The WAVES study.* International Journal of Behavioral Nutrition and Physical Activity, 2014. **11**(1).
271. *Canaway A, Frew E, Lancashire E, Pallan M, Hemming K, Adab P, WAVES trial investigators. Economic evaluation of a childhood obesity prevention programme for children: Results from the WAVES cluster randomised controlled trial conducted in schools. PloS one. 2019;14(7).*
272. Widhalm, K., O. Helk, and O. Pachinger, *The Viennese EDDY Study as a Role Model for Obesity: Prevention by Means of Nutritional and Lifestyle Interventions.* Obesity Facts. **11**(3): p. 247-256.
273. Haiquan, X., et al., *Comprehensive school-based intervention to control overweight and obesity in China: a cluster randomized controlled trial.* Asia Pacific Journal of Clinical Nutrition, 2017. **26**(6): p. 1139-1151.
274. Md Yusop, N.B., et al., *The effectiveness of a stage-based lifestyle modification intervention for obese children.* BMC Public Health. **18**(1): p. 299.
275. Ackel-D'Elia, C., et al., *Effects of different physical exercises on leptin concentration in obese adolescents.* International Journal of Sports Medicine. **35**(2): p. 164-71.
276. Adamo, K.B., et al., *Effects of a preschool intervention on physical activity and body composition.* The Journal of pediatrics, 2017. **188**: p. 42-49. e2.

277. Adamo, K.B., et al., *Activity Begins in Childhood (ABC) - inspiring healthy active behaviour in preschoolers: Study protocol for a cluster randomized controlled trial.* Trials, 2014. **15**(1).
278. Aggeloussi, S., et al., *Adipocytokine Levels in Children: Effects of Fatness and Training.* Pediatric Exercise Science, 2012. **24**(3): p. 461-471.
279. Alberga, A.S., et al., *The effects of resistance exercise training on body composition and strength in obese prepubertal children.* Phys Sportsmed, 2013. **41**(3): p. 103-9.
280. Almas, A., M. Islam, and T.H. Jafar, *School-based physical activity programme in preadolescent girls (9-11 years): a feasibility trial in Karachi, Pakistan.* Arch Dis Child, 2013. **98**(7): p. 515-9.
281. Andrade, S., et al., *Two years of school-based intervention program could improve the physical fitness among Ecuadorian adolescents at health risk: subgroups analysis from a cluster-randomized trial.* BMC pediatrics, 2016. **16**(1): p. 51.
282. Balagopal, P., et al., *Reversal of obesity-related hypoadiponectinemia by lifestyle intervention: a controlled, randomized study in obese adolescents.* The Journal of Clinical Endocrinology & Metabolism, 2005. **90**(11): p. 6192-6197.
283. Balagopal, P., et al., *Effect of lifestyle changes on whole-body protein turnover in obese adolescents.* International Journal of Obesity, 2003. **27**(10): p. 1250-1257.
284. Balagopal, P., et al., *Lifestyle-only intervention attenuates the inflammatory state associated with obesity: a randomized controlled study in adolescents.* Journal of Pediatrics, 2005. **146**(3): p. 342-8.
285. Balagopal, P., et al., *Response of fractional synthesis rate (FSR) of fibrinogen, concentration of D-dimer and fibrinolytic balance to physical activity-based intervention in obese children.* Journal of Thrombosis & Haemostasis, 2008. **6**(8): p. 1296-303.
286. Balagopal, P.B., et al., *Changes in circulating satiety hormones in obese children: a randomized controlled physical activity-based intervention study.* Obesity, 2010. **18**(9): p. 1747-53.
287. Barbosa Filho, V.C., et al., *"For whom was it effective?" Moderators of the effect of a school-based intervention on potential physical activity determinants among Brazilian students.* Preventive Medicine, 2017. **97**: p. 80-85.
288. Barbosa Filho, V.C., et al., *Effect of a Multicomponent Intervention on Lifestyle Factors among Brazilian Adolescents from Low Human Development Index Areas: A Cluster-Randomized Controlled Trial.* Int J Environ Res Public Health, 2019. **16**(2).
289. Barbosa Filho, V.C., et al., *Rationale and methods of a cluster-randomized controlled trial to promote active and healthy lifestyles among Brazilian students: the "Fortaleça sua Saúde" program.* BMC Public Health, 2015. **15**(1): p. 1-15.
290. Barkin, S.L., et al., *Changing overweight Latino preadolescent body mass index: the effect of the parent-child dyad.* Clinical pediatrics, 2011. **50**(1): p. 29-36.
291. Bastian, K.A., et al., *Does School-Based Health Promotion Affect Physical Activity on Weekends? And, Does It Reach Those Students Most in Need of Health Promotion?* PLoS ONE [Electronic Resource]. **10**(10): p. e0137987.
292. Benjamin Neelon, S.E., et al., *A community-based intervention increases physical activity and reduces obesity in school-age children in North Carolina.* Childhood Obesity, 2015. **11**(3): p. 297-303.
293. Berge, J.M., et al., *Play it forward! A community-based participatory research approach to childhood obesity prevention.* Families, Systems & Health: The Journal of Collaborative Family HealthCare, 2016. **34**(1): p. 15-30.
294. Bibiloni, M.D.M., et al., *Reversion of overweight and obesity in Vilafranca del Penedès child population: aCTIVA'T Program (2012).* Gaceta sanitaria, 2019. **33**(2): p. 197-202.
295. Bohlin, A., et al., *Childhood obesity treatment: telephone coaching is as good as usual care in maintaining weight loss–a randomized controlled trial.* Clinical obesity, 2017. **7**(4): p. 199-205.

296. Borjesson, U., et al., *Providing Mothers with a Pedometer and Subsequent Effect on the Physical Activity of Their Children: a Randomized Controlled Trial of Children with Obesity.* Childhood obesity (Print), 2019.
297. Camhi, S.M., J. Phillips, and D.R. Young, *The influence of body mass index on long-term fitness from physical education in adolescent girls.* Journal of school health, 2011. **81**(7): p. 409-416.
298. *Young DR, Phillips JA, Yu T, Haythornthwaite JA. Effects of a life skills intervention for increasing physical activity in adolescent girls. Arch Pediatr Adolesc Med. 2006; 160(12):1255–61. [PubMed: 17146023]*
299. Carlin, A., et al., *Effects of a peer-led Walking In ScHools intervention (the WISH study) on physical activity levels of adolescent girls: A cluster randomised pilot study.* Trials, 2018. **19**(1).
300. Chang, C., et al., *Effect of supervised exercise intervention on metabolic risk factors and physical fitness in Chinese obese children in early puberty.* Obesity Reviews, 2008. **9 Suppl 1**: p. 135-41.
301. Chen, S.R., et al., *Effects of a physical activity intervention on autonomic and executive functions in obese young adolescents: A randomized controlled trial.* Health Psychology. **35**(10): p. 1120-5.
302. Chen, H. and H. Sun, *The Effects of Active Videogame Feedback and Practicing Experience on Children's Physical Activity Intensity and Enjoyment.* Games Health J, 2017. **6**(4): p. 200-204.
303. Christison, A.L., et al., *Exergaming for Health: A Randomized Study of Community-Based Exergaming Curriculum in Pediatric Weight Management.* Games for Health Journal. **5**(6): p. 413-421.
304. Coimbra, S., et al., *Physical exercise intervention at school improved hepcidin, inflammation, and iron metabolism in overweight and obese children and adolescents.* Pediatric Research, 2017: p. N.PAG-N.PAG.
305. Cvetkovic, N., et al., *Exercise training in overweight and obese children: Recreational football and high-intensity interval training provide similar benefits to physical fitness.* Scandinavian Journal of Medicine & Science in Sports. **28 Suppl 1**: p. 18-32.
306. Damaso, A.R., et al., *Aerobic plus resistance training was more effective in improving the visceral adiposity, metabolic profile and inflammatory markers than aerobic training in obese adolescents.* Journal of Sports Sciences. **32**(15): p. 1435-45.
307. Davis, C.L., et al., *Aerobic exercise and snoring in overweight children: a randomized controlled trial.* Obesity, 2006. **14**(11): p. 1985-91.
308. Davis, C.L., et al., *Exercise Improves Executive Function and Achievement and Alters Brain Activation in Overweight Children: A Randomized, Controlled Trial.* Health Psychology, 2011. **30**(1): p. 91-98.
309. Davis, C.L., et al., *Exercise dose and diabetes risk in overweight and obese children: a randomized controlled trial.* JAMA, 2012. **308**(11): p. 1103-12.
310. Deforche, B., et al., *Post-treatment phone contact: a weight maintenance strategy in obese youngsters.* International journal of obesity, 2005. **29**(5): p. 543-546.
311. de Greeff, J.W., et al., *Effect of physically active academic lessons on body mass index and physical fitness in primary school children.* Journal of School Health, 2016. **86**(5): p. 346-352.
312. Delgado-Floody, P., et al., *Effects of 28 weeks of high-intensity interval training during physical education classes on cardiometabolic risk factors in Chilean schoolchildren: a pilot trial.* European Journal of Pediatrics. **177**(7): p. 1019-1027.
313. Dennis, B.A., et al., *Oxidative stress and cardiovascular risk in overweight children in an exercise intervention program.* Childhood Obesity. **9**(1): p. 15-21.
314. Donnelly, J.E., et al., *Physical Activity Across the Curriculum (PAAC): a randomized controlled trial to promote physical activity and diminish overweight and obesity in elementary school children.* Preventive medicine, 2009. **49**(4): p. 336-341.
315. DuBose, K.D., et al., *Physical activity across the curriculum (PAAC): Rationale and design.* Contemporary Clinical Trials, 2008. **29**(1): p. 83-93.

316. Eichner, J.E., O.A. Folorunso, and W.E. Moore, *A Physical Activity Intervention and Changes in Body Mass Index at a Middle School With a Large American Indian Population, Oklahoma, 2004-2009.* Preventing chronic disease, 2016. **13**: p. E163-E163.
317. Eiholzer, U., et al., *High-intensity training increases spontaneous physical activity in children: a randomized controlled study.* Journal of pediatrics, 2010. **156**(2): p. 242-6.
318. Epstein, L.H., et al., *A randomized trial of the effects of reducing television viewing and computer use on body mass index in young children.* Archives of pediatrics & adolescent medicine, 2008. **162**(3): p. 239-245.
319. Erfle, S.E. and A. Gamble, *Effects of daily physical education on physical fitness and weight status in middle school adolescents.* Journal of School Health, 2015. **85**(1): p. 27-35.
320. Escobar-Chaves, S.L., et al., *The Fun Families Study: intervention to reduce children's TV viewing.* Obesity, 2010. **18**(S1): p. S99-S101.
321. Faith, M.S., et al., *Effects of contingent television on physical activity and television viewing in obese children.* Pediatrics, 2001. **107**(5): p. 1043-1048.
322. Farias, E.d.S., et al., *Effects of programmed physical activity on body composition in post-pubertal schoolchildren.* Jornal de pediatria, 2015. **91**(2): p. 122-129.
323. Farmer, V., et al., *The effect of increasing risk and challenge in the school playground on physical activity and weight in children: a cluster randomised controlled trial (PLAY).* International Journal of Obesity, 2017. **41**(5): p. 793-800.
324. Fiorilli, G., et al., *Different consecutive training protocols to design an intervention program for overweight youth: A controlled study.* Diabetes, Metabolic Syndrome and Obesity: Targets and Therapy, 2017. **10**: p. 37-45.
325. Goldfield, G.S., et al., *Effects of open-loop feedback on physical activity and television viewing in overweight and obese children: a randomized, controlled trial.* Pediatrics, 2006. **118**(1): p. e157-e166.
326. Goldfield, G.S., et al., *Open-loop feedback to increase physical activity in obese children.* International Journal of Obesity & Related Metabolic Disorders: Journal of the International Association for the Study of Obesity. **24**(7): p. 888-92.
327. Goldfield, G.S., et al., *Gender differences in response to a physical activity intervention in overweight and obese children.* Journal of Physical Activity & Health, 2008. **5**(4): p. 592-606.
328. Graves, L.E., et al., *The effect of active video gaming on children's physical activity, behavior preferences and body composition.* Pediatr Exerc Sci, 2010. **22**(4): p. 535-46.
329. Gutin, B., et al., *Effect of physical training and its cessation on percent fat and bone density of children with obesity.* Obesity Research, 1999. **7**(2): p. 208-14.
330. Hagströmer, M., et al., *Participation in organized weekly physical exercise in obese adolescents reduced daily physical activity.* Acta paediatrica, 2009. **98**(2): p. 352-354.
331. Ham, O.K., et al., *Transtheoretical Model Based Exercise Counseling Combined with Music Skipping Rope Exercise on Childhood Obesity.* Asian Nursing Research, 2016. **10**(2): p. 116-122.
332. Hayes, R.M., et al., *Effects of a Brief Physical Activity Program on Young Students' Physical Fitness.* Clinical Pediatrics. **54**(12): p. 1145-52.
333. Herrick, H., et al., *Use of SPARK to Promote After-School Physical Activity.* Journal of School Health, 2012. **82**(10): p. 457-461.
334. Hollis, J.L., et al., *Effects of a 'school-based'physical activity intervention on adiposity in adolescents from economically disadvantaged communities: secondary outcomes of the 'Physical Activity 4 Everyone'RCT.* International Journal of Obesity, 2016. **40**(10): p. 1486-1493.
335. Hollis, J.L., et al., *A socio-ecologically framed, school-based physical activity intervention has beneficial effects on obesity outcomes in adolescents from low ses communities: The PA4E1 RCT.* Obesity Facts, 2015. **8**: p. 110.

336. Sutherland, R., et al., *A cluster randomised trial of an intervention to increase the implementation of physical activity practices in secondary schools: study protocol for scaling up the Physical Activity 4 Everyone (PA4E1) program.* BMC public health, 2019. **19**(1): p. 883.
337. Sutherland, R., et al., *A randomized controlled trial to assess the potential efficacy, feasibility and acceptability of an m-health intervention targeting parents of school aged children to improve the nutritional quality of foods packed in the lunchbox 'SWAP IT'.* International Journal of Behavioral Nutrition and Physical Activity, 2019. **16**(1).
338. Sutherland, R., et al., *Cost effectiveness of a multi-component school-based physical activity intervention targeting adolescents: the 'Physical Activity 4 Everyone' cluster randomized trial.* International journal of behavioral nutrition and physical activity, 2016. **13**: p. 94.
339. Hull, P.C., et al., *Childhood obesity prevention cluster randomized trial for Hispanic families: outcomes of the healthy families study.* Pediatric Obesity. **13**(11): p. 686-696.
340. Ildiko, V., et al., *Activity-related changes of body fat and motor performance in obese seven-year-old boys.* Journal of Physiological Anthropology. **26**(3): p. 333-7.
341. Ingul, C.B., et al., *Effect of High Intensity Interval Training on Cardiac Function in Children with Obesity: a Randomised Controlled Trial.* Progress in cardiovascular diseases, 2018. **61**(2): p. 214-221.
342. Jago, R., et al., *Action 3: 30R: results of a Cluster Randomised Feasibility Study of a Revised Teaching Assistant-Led Extracurricular Physical Activity Intervention for 8 to 10 Year Olds.* International journal of environmental research and public health, 2019. **16**(1).
343. Jones, R.A., et al., *Acceptability and Potential Efficacy of Single-Sex After-School Activity Programs for Overweight and At-Risk Children: The Wollongong SPORT RCT.* Pediatric Exercise Science, 2015. **27**(4): p. 535-545.
344. Klakk, H., et al., *Effect of four additional physical education lessons on body composition in children aged 8–13 years–a prospective study during two school years.* BMC pediatrics, 2013. **13**(1): p. 170.
345. Krafft, C.E., et al., *An eight month randomized controlled exercise intervention alters resting state synchrony in overweight children.* Neuroscience. **256**: p. 445-55.
346. Krafft, C.E., et al., *An 8-month randomized controlled exercise trial alters brain activation during cognitive tasks in overweight children.* Obesity, 2014. **22**(1): p. 232-242.
347. Krombholz, H., *The impact of a 20-month physical activity intervention in child care centers on motor performance and weight in overweight and healthy-weight preschool children.* Perceptual and Motor Skills, 2012. **115**(3): p. 919-932.
348. Lazzer, S., et al., *Fat oxidation rate during and after a low- or high-intensity exercise in severely obese Caucasian adolescents.* European journal of applied physiology, 2009. **108**(2): p. 383-91.
349. Lee, K.J., et al., *Aerobic exercise training-induced decrease in plasma visfatin and insulin resistance in obese female adolescents.* International Journal of Sport Nutrition & Exercise Metabolism. **20**(4): p. 275-81.
350. Liu, A.L., et al., *Report on childhood obesity in China (6) evaluation of a classroom-based physical activity promotion program.* Biomedical & Environmental Sciences, 2007. **20**(1): p. 19-23.
351. Liu, A., et al., *Evaluation of a classroom-based physical activity promoting programme.* Obesity Reviews, 2008. **9**: p. 130-134.
352. Maddison, R., et al., *Active video games: the mediating effect of aerobic fitness on body composition.* International Journal of Behavioral Nutrition and Physical Activity, 2012. **9**(1): p. 54.
353. Maddison, R., et al., *Feasibility, design and conduct of a pragmatic randomized controlled trial to reduce overweight and obesity in children: The electronic games to aid motivation to exercise (eGAME) study.* BMC Public Health, 2009. **9**: p. 146.
354. Maddison, R., et al., *Effects of active video games on body composition: a randomized controlled trial.* Am J Clin Nutr, 2011. **94**(1): p. 156-63.

355. Manley, D., et al., *Self-efficacy, physical activity, and aerobic fitness in middle school children: Examination of a pedometer intervention program.* Journal of pediatric nursing, 2014. **29**(3): p. 228-237.
356. Vizcaíno, V.M., et al., *Assessment of an after-school physical activity program to prevent obesity among 9-to 10-year-old children: a cluster randomized trial.* International journal of obesity, 2008. **32**(1): p. 12-22.
357. Salcedo Aguilar, F., et al., *Impact of an after-school physical activity program on obesity in children.* Journal of pediatrics, 2010. **157**(1): p. 36-42.e3.
358. Martínez-Vizcaíno, V., et al., *Gender differences on effectiveness of a school-based physical activity intervention for reducing cardiometabolic risk: A cluster randomized trial.* International Journal of Behavioral Nutrition and Physical Activity, 2014. **11**(1).
359. Miguet, M., et al., *Effect of HIIT versus MICT on body composition and energy intake in dietary restrained and unrestrained adolescents with obesity.* Physiologie appliquee, nutrition et metabolisme [Applied physiology, nutrition, and metabolism], 2019.
360. Miguet, M., et al., *Energy intake, appetite and food reward responses to High Intensity Interval Exercise might depend on the degree of obesity in adolescents.* Obesity facts, 2018. **11**: p. 33-.
361. Møller, N.C., et al., *Do extra compulsory physical education lessons mean more physically active children - findings from the childhood health, activity, and motor performance school study Denmark (The CHAMPS-study DK).* International Journal of Behavioral Nutrition & Physical Activity, 2014. **11**: p. 199-221.
362. Monteiro, P.A., et al., *Concurrent and aerobic exercise training promote similar benefits in body composition and metabolic profiles in obese adolescents.* Lipids in health and disease, 2015. **14**(1): p. 153.
363. Ahola, R., et al., *Gamified physical activation of young men--a Multidisciplinary Population-Based Randomized Controlled Trial (MOPO study).* BMC Public Health. **13**: p. 32.
364. *Leinonen AM, Pyky R, Ahola R, Kangas M, Siirtola P, Luoto T, Enwald H, Ikäheimo TM, Röning J, Keinänen-Kiukaanniemi S, Mäntysaari M, Korpelainen R, Jämsä T. Feasibility of Gamified Mobile Service Aimed at Physical Activation in Young Men: Population-Based Randomized Controlled Study (MOPO). JMIR Mhealth Uhealth. 2017 Oct 10;5(10):e146. doi: 10.2196/mhealth.6675.*
365. *Jauho AM, Pyky R, Ahola R, Kangas M, Virtanen P, Korpelainen R, Jämsä T. Effect of wrist-worn activity monitor feedback on physical activity behavior: A randomized controlled trial in Finnish young men. Prev Med Rep. 2015 Jul 22;2:628-34. doi: 10.1016/j.pmedr.2015.07.005. eCollection 2015. .*
366. Morgan, P., K. Saunders, and D. Lubans, *Improving physical self-perception in adolescent boys from disadvantaged schools: psychological outcomes from the P hysical A ctivity L eaders randomized controlled trial.* Pediatric obesity, 2012. **7**(3): p. e27-e32.
367. Muller, I., et al., *Effect of a Multidimensional Physical Activity Intervention on Body Mass Index, Skinfolds and Fitness in South African Children: Results from a Cluster-Randomised Controlled Trial.*
368. Nascimento, H., et al., *Impact of a School-Based Intervention Protocol - ACORDA Project - On Adipokines in An Overweight and Obese Pediatric Population.* Pediatric exercise science, 2016. **28**(3): p. 407-416.
369. Neumark-Sztainer, D., et al., *New Moves: a school-based obesity prevention program for adolescent girls.* Preventive medicine, 2003. **37**(1): p. 41-51.
370. Bauer, K.W., et al., *Relationships between the family environment and school-based obesity prevention efforts: can school programs help adolescents who are most in need?* Health Education Research, 2011. **26**(4): p. 675-88.
371. Neumark-Sztainer, D., et al., *Dietary approaches to healthy weight management for adolescents: the New Moves model.* Adolescent Medicine, 2008. **19**(3): p. 421-30, viii.

372. Nobre, G.G., et al., *Twelve weeks of Plyometric training improves motor performance of 7-to 9-year-old boys who were overweight/obese: a randomized controlled intervention.* The Journal of Strength & Conditioning Research, 2017. **31**(8): p. 2091-2099.
373. Nogueira, R.C., B.K. Weeks, and B.R. Beck, *An in-school exercise intervention to enhance bone and reduce fat in girls: the CAPO Kids trial.* Bone. **68**: p. 92-9.
374. Nowicka, P., et al., *Sports camp with six months of support from a local sports club as a treatment for childhood obesity.* Scandinavian journal of public health, 2009. **37**(8): p. 793-800.
375. Olvera, N., et al., *BOUNCE: a community-based mother–daughter healthy lifestyle intervention for low-income Latino families.* Obesity, 2010. **18**(S1): p. S102-S104.
376. Olvera, N.N., et al., *A healthy lifestyle program for Latino daughters and mothers: the BOUNCE overview and process evaluation.* American Journal of Health Education, 2008. **39**(5): p. 283-295.
377. Oreskovic, N.M., et al., *A multimodal counseling-based adolescent physical activity intervention.* Journal of Adolescent Health, 2016. **59**(3): p. 332-337.
378. Oreskovic, N.M., et al., *Design and implementation of a physical activity intervention to enhance children's use of the built environment (the CUBE study).* Contemporary Clinical Trials. **40**: p. 172-9.
379. Pate, R.R., et al., *Promotion of physical activity among high-school girls: a randomized controlled trial.* American Journal of Public Health, 2005. **95**(9): p. 1582-7.
380. Prado, W.L., et al., *Effect of a 12-Week Low vs. High Intensity Aerobic Exercise Training on Appetite-Regulating Hormones in Obese Adolescents: A Randomized Exercise Intervention Study.* Pediatric Exercise Science. **27**(4): p. 510-7.
381. Racil, G., et al., *Plyometric exercise combined with high-intensity interval training improves metabolic abnormalities in young obese females more so than interval training alone.* Applied Physiology, Nutrition & Metabolism, 2016. **41**(1): p. 103-109.
382. Riiser, K., et al., *The outcomes of a 12-week Internet intervention aimed at improving fitness and health-related quality of life in overweight adolescents: the Young & Active controlled trial.* PLoS One, 2014. **9**(12).
383. Robbins, L.B., et al., *Girls on the move program to increase physical activity participation.* Nursing research, 2006. **55**(3): p. 206-216.
384. Robbins, L.B., et al., *Examining reach, dose, and fidelity of the "Girls on the Move" after-school physical activity club: A process evaluation.* BMC public health, 2016. **16**(1): p. 671.
385. Robinson, T.N., *Reducing children's television viewing to prevent obesity: a randomized controlled trial.* Jama, 1999. **282**(16): p. 1561-1567.
386. Robinson, T.N., et al., *Effects of reducing television viewing on children's requests for toys: a randomized controlled trial.* J Dev Behav Pediatr, 2001. **22**(3): p. 179-84.
387. Robinson, T.N., et al., *A randomized controlled trial of culturally tailored dance and reducing screen time to prevent weight gain in low-income African American girls: Stanford GEMS.* Archives of pediatrics & adolescent medicine, 2010. **164**(11): p. 995-1004.
388. Robinson, T.N., et al., *Dance and reducing television viewing to prevent weight gain in African-American girls: the Stanford GEMS pilot study.* Ethn Dis, 2003. **13**(1 Suppl 1): p. S65-77.
389. Robinson, T.N., et al., *Stanford GEMS phase 2 obesity prevention trial for low-income African-American girls: Design and sample baseline characteristics.* Contemporary Clinical Trials, 2008. **29**(1): p. 56-69.
390. Rolland-Cachera, M.F., et al., *Massive obesity in adolescents: dietary interventions and behaviours associated with weight regain at 2 y follow-up.* International Journal of Obesity & Related Metabolic Disorders: Journal of the International Association for the Study of Obesity, 2004. **28**(4): p. 514-9.
391. Roth, K., et al., *Effects of a Physical Activity Intervention in Preschool Children.* Medicine & Science in Sports & Exercise, 2015. **47**(12): p. 2542-2551.

392. Roth, K., et al., *Prevention through Activity in Kindergarten Trial (PAKT): a cluster randomised controlled trial to assess the effects of an activity intervention in preschool children.* BMC Public Health, 2010. **10**(1): p. 410-419.
393. Sacchetti, R., et al., *Effects of a 2-year school-based intervention of enhanced physical education in the primary school.* Journal of school health, 2013. **83**(9): p. 639-646.
394. Salmon, J., et al., *Outcomes of a group-randomized trial to prevent excess weight gain, reduce screen behaviours and promote physical activity in 10-year-old children: switch-play.* International journal of obesity, 2008. **32**(4): p. 601-612.
395. Maddison, R., et al., *Screen-Time Weight-loss Intervention Targeting Children at Home (SWITCH): a randomized controlled trial.* International Journal of Behavioral Nutrition & Physical Activity. **11**: p. 111.
396. Salmon, J., et al., *Reducing sedentary behaviour and increasing physical activity among 10-year-old children: overview and process evaluation of the 'Switch-Play' intervention.* 2005. **1**(1): p. 7-17.
397. Schaeffer, D.J., et al., *An 8-month exercise intervention alters frontotemporal white matter integrity in overweight children.* Psychophysiology. **51**(8): p. 728-33.
398. Scheffler, C., K. Ketelhut, and I. Mohasseb, *Does physical education modify the body composition?--results of a longitudinal study of pre-school children.* Anthropologischer Anzeiger, 2007. **65**(2): p. 193-201.
399. Schranz, N., et al., *Can resistance training change the strength, body composition and self-concept of overweight and obese adolescent males? A randomised controlled trial.* Br J Sports Med, 2014. **48**(20): p. 1482-1488.
400. Schwanke, N.L., et al., *Differences in body posture, strength and flexibility in schoolchildren with overweight and obesity: A quasi-experimental study.* Manual Therapy. **22**: p. 138-44.
401. Seabra, A., et al., *Effects of 6-month soccer and traditional physical activity programmes on body composition, cardiometabolic risk factors, inflammatory, oxidative stress markers and cardiorespiratory fitness in obese boys.* Journal of Sports Sciences, 2016. **34**(19): p. 1822-1829.
402. Seo, Y.G., et al., *The Effect of a Multidisciplinary Lifestyle Intervention on Obesity Status, Body Composition, Physical Fitness, and Cardiometabolic Risk Markers in Children and Adolescents with Obesity.* Nutrients, 2019. **11**(1).
403. Shaibi, G.Q., et al., *Effects of resistance training on insulin sensitivity in overweight Latino adolescent males.* Medicine & Science in Sports & Exercise, 2006. **38**(7): p. 1208-15.
404. Siegrist, M., et al., *A cluster randomised school-based lifestyle intervention programme for the prevention of childhood obesity and related early cardiovascular disease (JuvenTUM 3).* BMC Public Health, 2011. **11**(1): p. 258-258.
405. Siegrist, M., et al., *Effects of a cluster-randomized school-based prevention program on physical activity and microvascular function (JuvenTUM 3).* Atherosclerosis, 2018. **278**: p. 73-81.
406. Sigal, R.J., et al., *Effects of aerobic training, resistance training, or both on percentage body fat and cardiometabolic risk markers in obese adolescents: the healthy eating aerobic and resistance training in youth randomized clinical trial.* JAMA Pediatrics. **168**(11): p. 1006-14.
407. Sigmund, E., W. El Ansari, and D. Sigmundová, *Does school-based physical activity decrease overweight and obesity in children aged 6-9 years? A two-year non-randomized longitudinal intervention study in the Czech Republic.* BMC Public Health, 2012. **12**(1): p. 570-570.
408. Silva, H.J., et al., *Improvements on Cardiovascular Diseases Risk Factors in Obese Adolescents: A Randomized Exercise Intervention Study.* Journal of Physical Activity & Health. **12**(4): p. 553-60.
409. Simon, C., et al., *A socio-ecological approach promoting physical activity and limiting sedentary behavior in adolescence showed weight benefits maintained 2.5 years after intervention cessation.* International journal of obesity, 2014. **38**(7): p. 936-943.

410. Simon, C., et al., *Successful overweight prevention in adolescents by increasing physical activity: a 4-year randomized controlled intervention.* International Journal of Obesity, 2008. **32**(10): p. 1489-98.
411. Simon, C., et al., *Intervention centred on adolescents' physical activity and sedentary behaviour (ICAPS): Concept and 6-month results.* International Journal of Obesity, 2004. **28**(SUPPL. 3): p. S96-S103.
412. Smith, J.J., et al., *Mediating effects of resistance training skill competency on health-related fitness and physical activity: The ATLAS cluster randomised controlled trial.* Journal of sports sciences, 2016. **34**(8): p. 772-779.
413. Lubans, D., et al., *Improving muscular fitness enhances psychological well-being in low-income adolescent boys: Findings from the ATLAS cluster RCT.* Journal of Science and Medicine in Sport, 2015. **19**: p. e75.
414. Lubans, D.R., et al., *Assessing the sustained impact of a school-based obesity prevention program for adolescent boys: the ATLAS cluster randomized controlled trial.* International Journal of Behavioral Nutrition & Physical Activity. **13**: p. 92.
415. Smith, J.D., et al., *An individually tailored family-centered intervention for pediatric obesity in primary care: study protocol of a randomized type II hybrid effectiveness-implementation trial (Raising Healthy Children study).* Implementation science, 2018. **13**(1): p. 11.
416. Smith, J.J., et al., *Rationale and study protocol for the 'active teen leaders avoiding screen-time' (ATLAS) group randomized controlled trial: an obesity prevention intervention for adolescent boys from schools in low-income communities.* Contemp Clin Trials, 2014. **37**(1): p. 106-19.
417. Smith, J.J., et al., *Smart-phone obesity prevention trial for adolescent boys in low-income communities: The ATLAS RCT.* Pediatrics, 2014. **134**(3): p. e723-e731.
418. Son, W.M., et al., *Combined exercise training reduces blood pressure, arterial stiffness, and insulin resistance in obese prehypertensive adolescent girls.* FASEB journal, 2017. **31**(1).
419. Karki, S., et al., *Combined exercise training reduces blood pressure, arterial stiffness, and insulin resistance in obese prehypertensive adolescent girls.* 2017.
420. Spruijt-Metz, D., et al., *Reducing sedentary behavior in minority girls via a theory-based, tailored classroom media intervention.* International Journal of Pediatric Obesity, 2008. **3**(4): p. 240-248.
421. Staiano, A.E., A.A. Abraham, and S.L. Calvert, *Adolescent exergame play for weight loss and psychosocial improvement: a controlled physical activity intervention.* Obesity, 2013. **21**(3): p. 598-601.
422. Staiano, A., et al., *A randomized controlled trial of dance exergaming for exercise training in overweight and obese adolescent girls.* Pediatric Obesity, 2017. **12**(2): p. 120-128.
423. Staiano, A.E., A.A. Abraham, and S.L. Calvert, *Motivating effects of cooperative exergame play for overweight and obese adolescents.* Journal of Diabetes Science & Technology. **6**(4): p. 812-9.
424. Staiano, A.E., et al., *Home-based exergaming among children with overweight and obesity: a randomized clinical trial.* Pediatric Obesity. **13**(11): p. 724-733.
425. Staiano, A.E., et al., *Twelve weeks of dance exergaming in overweight and obese adolescent girls: transfer effects on physical activity, screen time, and self-efficacy.* Journal of sport and health science, 2017. **6**(1): p. 4-10.
426. Tkacz, J., et al., *Aerobic exercise program reduces anger expression among overweight children.* Pediatric Exercise Science. **20**(4): p. 390-401.
427. Thivel, D., et al., *Effect of a 6-month school-based physical activity program on body composition and physical fitness in lean and obese schoolchildren.* European journal of pediatrics, 2011. **170**(11): p. 1435-1443.

428. Tsang, T.W., et al., *A randomized controlled trial of Kung Fu training for metabolic health in overweight/obese adolescents: The "martial fitness" study.* Journal of Pediatric Endocrinology and Metabolism, 2009. **22**(7): p. 595-607.
429. van Stralen, M.M., et al., *Mediators of the effect of the JUMP-in intervention on physical activity and sedentary behavior in Dutch primary schoolchildren from disadvantaged neighborhoods.* International journal of behavioral nutrition and physical activity, 2012. **9**(1): p. 131.
430. Jurg, M.E., et al., *A controlled trial of a school-based environmental intervention to improve physical activity in Dutch children: JUMP-in, kids in motion.* Health Promotion International, 2006. **21**(4): p. 320-30.
431. Vasconcellos, F., et al., *Health markers in obese adolescents improved by a 12-week recreational soccer program: a randomised controlled trial.* Journal of Sports Sciences. **34**(6): p. 564-75.
432. Velez, A., D.L. Golem, and S.M. Arent, *The impact of a 12-week resistance training program on strength, body composition, and self-concept of Hispanic adolescents.* The Journal of Strength & Conditioning Research, 2010. **24**(4): p. 1065-1073.
433. Villa-González, E., et al., *Effects of a school-based intervention on active commuting to school and health-related fitness.* BMC public health, 2017. **17**(1): p. 20.
434. Walther, C., et al., *Effect of increased exercise in school children on physical fitness and endothelial progenitor cells: a prospective randomized trial.* Circulation, 2009. **120**(22): p. 2251-9.
435. Weintraub, D.L., et al., *Team sports for overweight children: The Stanford Sports to Prevent Obesity Randomized Trial (SPORT).* Archives of Pediatrics and Adolescent Medicine, 2008. **162**(3): p. 232-237.
436. Whooten, R.C., et al., *Effects of Before-School Physical Activity on Obesity Prevention and Wellness.* American Journal of Preventive Medicine. **54**(4): p. 510-518.
437. Wong, P.C., et al., *Effects of a 12-week exercise training programme on aerobic fitness, body composition, blood lipids and C-reactive protein in adolescents with obesity.* Annals of the Academy of Medicine, Singapore, 2008. **37**(4): p. 286-93.
438. Wright, C.M., et al., *The FLEX study school-based physical activity programs - measurement and evaluation of implementation.* BMC public health, 2019. **19**(1): p. 73.
439. Wright, C.M., et al., *Study protocol: the Fueling Learning through Exercise (FLEX) study - a randomized controlled trial of the impact of school-based physical activity programs on children's physical activity, cognitive function, and academic achievement.* BMC Public Health. **16**(1): p. 1078.
440. Yıldırım, M., et al., *What helps children to move more at school recess and lunchtime? Mid-intervention results from Transform-Us! cluster-randomised controlled trial.* Br J Sports Med, 2014. **48**(3): p. 271-277.
441. Zehsaz, F., N. Farhangi, and M. Ghahramani, *Exercise training lowers serum chemerin concentration in obese children.* Science and sports. (no pagination), 2016, 2016. **Date of Publication: July 22**.
442. Zehsaz, F., N. Farhangi, and M. Ghahramani, *The response of circulating omentin-1 concentration to 16-week exercise training in male children with obesity.* Physician & Sportsmedicine. **44**(4): p. 355-361.
443. Abraham, A.A., et al., *Lifestyle intervention using an internet-based curriculum with cell phone reminders for obese Chinese teens: A randomized controlled study.* PLoS ONE, 2015. **10**(5).
444. Ahmad, N., et al., *Family-based intervention using face-to-face sessions and social media to improve Malay primary school children's adiposity: a randomized controlled field trial of the Malaysian REDUCE programme.* Nutrition Journal, 2018. **17**(1): p. N.PAG-N.PAG.
445. Alkon, A., et al., *Nutrition and physical activity randomized control trial in child care centers improves knowledge, policies, and children's body mass index.* BMC Public Health, 2014. **14**(1): p. 215-215.

446. Arlinghaus, K.R., et al., *Companeros: High School Students Mentor Middle School Students to Address Obesity Among Hispanic Adolescents.* Preventing Chronic Disease. **14**: p. E92.
447. Angelopoulos, P.D., et al., *Changes in BMI and blood pressure after a school based intervention: The CHILDREN study.* European Journal of Public Health, 2009. **19**(3): p. 319-325.
448. Armstrong, S., et al., *Texting Motivational Interviewing: a Randomized Controlled Trial of Motivational Interviewing Text Messages Designed to Augment Childhood Obesity Treatment.* Childhood obesity (Print), 2017. **14**(1): p. 4-10.
449. Bacardí-Gascon, M., M.E. Pérez-Morales, and A. Jiménez-Cruz, *A six month randomized school intervention and an 18-month follow-up intervention to prevent childhood obesity in Mexican elementary schools.* Nutricion Hospitalaria, 2012. **27**(3): p. 755-762.
450. Bagherniya, M., et al., *School-Based Nutrition Education Intervention Using Social Cognitive Theory for Overweight and Obese Iranian Adolescent Girls: A Cluster Randomized Controlled Trial.* International Quarterly of Community Health Education, 2017. **38**(1): p. 37-45.
451. Bagherniya, M., et al., *Assessment of the efficacy of physical activity level and lifestyle behavior interventions applying social cognitive theory for overweight and obese girl adolescents.* Journal of research in health sciences, 2018. **18**(2).
452. Ball, G.D.C., et al., *One-on-one lifestyle coaching for managing adolescent obesity: Findings from a pilot, randomized controlled trial in a real-world, clinical setting.* Paediatrics and Child Health, 2011. **16**(6): p. 345-350.
453. Baranowski, T., et al., *The Fun, Food, and Fitness Project (FFFP): the Baylor GEMS pilot study.* Ethnicity & Disease, 2003. **13**(1 Suppl 1): p. S30-9.
454. Barkin, S.L., et al., *Effect of a behavioral intervention for underserved preschool-age children on change in body mass index: A randomized clinical trial.* JAMA - Journal of the American Medical Association, 2018. **320**(5): p. 450-460.
455. Berkowitz, R.I., et al., *Treatment of adolescent obesity comparing self-guided and group lifestyle modification programs: a potential model for primary care.* Journal of pediatric psychology, 2013. **38**(9): p. 978-86.
456. Black, M.M., et al., *Challenge! health promotion/obesity prevention mentorship model among urban, black adolescents.* Pediatrics, 2010. **126**(2): p. 280-288.
457. Bolton, K.A., et al., *The outcomes of health-promoting communities: being active eating well initiative-a community-based obesity prevention intervention in Victoria, Australia.* International Journal of Obesity. **41**(7): p. 1080-1090.
458. de Silva-Sanigorski, A.M., et al., *Scaling up community-based obesity prevention in Australia: background and evaluation design of the Health Promoting Communities: Being Active Eating Well initiative.* BMC Public Health, 2010. **10**(1): p. 65-65.
459. Boodai, S.A., J.H. McColl, and J.J. Reilly, *National Adolescent Treatment Trial for Obesity in Kuwait (NATTO): project design and results of a randomised controlled trial of a good practice approach to treatment of adolescent obesity in Kuwait.* Trials, 2014. **15**(1): p. 234-234.
460. Boutelle, K.N., et al., *Guided self-help for the treatment of pediatric obesity.* Pediatrics, 2013. **131**(5): p. e1435-42.
461. Broccoli, S., et al., *Motivational interviewing to treat overweight children and their parents in paediatrician's medical office: good results, but one year is not sufficient.* Epidemiologia e prevenzione, 2015. **39**(5-6): p. 393.
462. Broccoli, S., et al., *Motivational interviewing to treat overweight children: 24-month follow-up of a randomized controlled trial.* Pediatrics, 2016. **137**(1).
463. Byrd, B., et al., *The marketing plan and outcome indicators for recruiting and retaining parents in the HomeStyles randomized controlled trial.* Trials, 2017. **18**: p. 1-18.

464. Byrd-Bredbenner, C., et al., *Promoting healthy home environments and lifestyles in families with preschool children: HomeStyles, a randomized controlled trial.* Contemporary Clinical Trials. **64**: p. 139-151.
465. Martin-Biggers, J.T., et al., *HomeStyles: recruitment strategies for a childhood obesity prevention randomized controlled trial.* FASEB journal, 2016. **30**.
466. Casazza, K. and M. Ciccazzo, *The method of delivery of nutrition and physical activity information may play a role in eliciting behavior changes in adolescents.* Eating Behaviors, 2007. **8**(1): p. 73-82.
467. Cason, K.L. and B.N. Logan, *Education intervention improves 4th-grade schoolchildren's nutrition and physical activity knowledge and behaviors.* Topics in Clinical Nutrition, 2006. **21**(3): p. 234-240.
468. Chawla, N., et al., *Effectiveness Of A School-Based Multicomponent Intervention On Nutritional Status Among Primary School Children In Bangkok, Thailand.* Journal of Ayub Medical College, Abbottabad: JAMC. **29**(1): p. 13-20.
469. Chen, J.L., C.M. Guedes, and A.E. Lung, *Smartphone-Based Healthy Weight Management Intervention for Chinese American Adolescents: short-Term Efficacy and Factors Associated With Decreased Weight.* Journal of adolescent health, 2018. **(no pagination)**.
470. Brennan, L., *Does motivational interviewing improve retention or outcome in cognitive behaviour therapy for overweight and obese adolescents?* Obesity Research & Clinical Practice. **10**(4): p. 481-6.
471. Brennan, L., et al., *Motivational interviewing and cognitive behaviour therapy in the treatment of adolescent overweight and obesity: study design and methodology.* Contemporary Clinical Trials, 2008. **29**(3): p. 359-75.
472. Brennan, L., et al., *Physiological and behavioural outcomes of a randomised controlled trial of a cognitive behavioural lifestyle intervention for overweight and obese adolescents.* Obesity Research and Clinical Practice, 2013. **7**(1): p. e23-e41.
473. Brennan, L., et al., *Treatment acceptability and psychosocial outcomes of a randomised controlled trial of a cognitive behavioural lifestyle intervention for overweight and obese adolescents.* Behaviour Change, 2012. **29**(1): p. 36-62.
474. Tsiros, M.D., et al., *Cognitive behavioral therapy improves diet and body composition in overweight and obese adolescents.* American Journal of Clinical Nutrition, 2008. **87**(5): p. 1134-40.
475. James, J., P. Thomas, and D. Kerr, *Preventing childhood obesity: Two year follow-up results from the Christchurch obesity prevention programme in schools (CHOPPS).* British Medical Journal, 2007. **335**(7623): p. 762-764.
476. James, J., et al., *Preventing childhood obesity by reducing consumption of carbonated drinks: cluster randomised controlled trial.* Bmj, 2004. **328**(7450): p. 1237.
477. Cloutier, M.M., et al., *Outcomes of an early childhood obesity prevention program in a low-income community: a pilot, randomized trial.* Pediatric Obesity, 2018. **13**(11): p. 677-685.
478. Cloutier, M.M., et al., *The Early Childhood Obesity Prevention Program (ECHO): an ecologically-based intervention delivered by home visitors for newborns and their mothers.* BMC public health, 2015. **15**: p. 584.
479. Cox, R., et al., *The Healthy Eating, Active Living (HEAL) Study: Outcomes, Lessons Learnt and Future Recommendations.* Child Abuse Review, 2017. **26**(3): p. 196-214.
480. Crespo, N.C., et al., *A randomized controlled trial to prevent obesity among Latino paediatric patients.* Pediatric Obesity. **13**(11): p. 697-704.
481. Hatzis, C.M., C. Papandreou, and A.G. Kafatos, *School health education programs in Crete: Evaluation of behavioural and health indices a decade after initiation.* Preventive Medicine, 2010. **51**(3/4): p. 262-267.

482. Kafatos, A., Y. Manios, and J. Moschandreas, *Health and nutrition education in primary schools of Crete: follow-up changes in body mass index and overweight status.* European Journal of Clinical Nutrition, 2005. **59**(9): p. 1090-1092.
483. *Manios Y, Moschandreas J, Hatzis C, Kafatos A. Evaluation of a health and nutrition education program in primary school children of Crete over a three-year period. Preventive medicine. 1999 Feb 1;28(2):149-59".*
484. Croker, H., et al., *Family-based behavioural treatment of childhood obesity in a UK National Health Service setting: randomized controlled trial.* International Journal of Obesity. **36**(1): p. 16-26.
485. Da Silva, K.B.B., et al., *Evaluation of the computer-based intervention program stayingfit Brazil to promote healthy eating habits: the results from a school cluster-randomized controlled trial.* International journal of environmental research and public health, 2019. **16**(10).
486. Davis, J.N., et al., *Effects of a randomized maintenance intervention on adiposity and metabolic risk factors in overweight minority adolescents.* Pediatric Obesity. **7**(1): p. 16-27.
487. Davis, A.M., et al., *Treating rural pediatric obesity through telemedicine: outcomes from a small randomized controlled trial.* Journal of pediatric psychology, 2013. **38**(9): p. 932-43.
488. Davoli, A.M., et al., *Pediatrician-led motivational interviewing to treat overweight children: An RCT.* Pediatrics, 2013. **132**(5): p. e1236-e1246.
489. Day, M.E., et al., *Action schools! BC--Healthy Eating: effects of a whole-school model to modifying eating behaviours of elementary school children.* Canadian Journal of Public Health, 2008. **99**(4): p. 328-331.
490. De Lepeleere, S., et al., *The effect of an online video intervention 'Movie Models' on specific parenting practices and parental self-efficacy related to children's physical activity, screen-time and healthy diet: a quasi experimental study.* BMC Public Health. **17**(1): p. 366.
491. De Lepeleere, S., et al., *Using the Intervention Mapping Protocol to develop an online video intervention for parents to prevent childhood obesity: Movie Models.* Global Health Promotion, 2018. **25**(2): p. 56-66.
492. de Moraes, M.M., et al., *Discouraging soft drink consumption reduces blood glucose and cholesterol of Brazilian elementary students: Secondary analysis of a randomized controlled trial.* Preventive Medicine, 2017: p. 223-228.
493. Dennison, B.A., et al., *An Intervention to Reduce Television Viewing by Preschool Children.* Archives of Pediatrics and Adolescent Medicine, 2004. **158**(2): p. 170-176.
494. de Villiers, A., et al., *Primary School Children's Nutrition Knowledge, Self-Efficacy, and Behavior, after a Three-Year Healthy Lifestyle Intervention (HealthKick).* Ethnicity & disease, 2016. **26**(2): p. 171-80.
495. de Visser, R., et al., *Changes in School Health Program Improve Middle School Students' Behaviors.* American Journal of Health Behavior, 2016. **40**(5): p. 568-77.
496. De Vries, A.G.M., et al., *An activity stimulation programme during a child's first year reduces some indicators of adiposity at the age of two-and-a-half.* Acta Paediatrica, International Journal of Paediatrics, 2015. **104**(4): p. 414-421.
497. Döring, N., et al., *Motivational interviewing to prevent childhood obesity: a cluster RCT.* Pediatrics, 2016. **137**(5): p. e20153104.
498. Doring, N., et al., *Primary prevention of childhood obesity through counselling sessions at Swedish child health centres: design, methods and baseline sample characteristics of the PRIMROSE cluster-randomised trial.* BMC Public Health. **14**: p. 335.
499. Döring, N., et al., *Economic evaluation of primrose-a trial-based analysis of an early childhood intervention to prevent obesity.* Frontiers in Endocrinology, 2018. **9**(MAR).
500. Eno Persson, J., et al., *Prevention of Childhood Obesity in Child Health Services: Follow-Up of the PRIMROSE Trial.* Childhood Obesity. **14**(2): p. 99-105.

501. Rohde, J.F., et al., *Cross-sectional associations between maternal self-efficacy and dietary intake and physical activity in four-year-old children of first-time Swedish mothers.* Appetite. **125**: p. 131-138.
502. Dunn, L.L., et al., *An observational evaluation of move-to-improve, a classroom-based physical activity program, New York City schools, 2010.* Preventing Chronic Disease, 2012. **9**: p. E146-E146.
503. Early, G.J. and N.D. Cheffer, *Motivational Interviewing and Home Visits to Improve Health Behaviors and Reduce Childhood Obesity: A Pilot Study.* Hispanic Health Care International, 2019. **17**(3): p. 103-110.
504. Eldridge, G., et al., *Effects of parent-only childhood obesity prevention programs on BMIz and body image in rural preteens.* Body Image, 2016. **16**: p. 143-153.
505. Epstein, L.H., et al., *Effects of decreasing sedentary behaviors on activity choice in obese children.* Health psychology, 1995. **16**(2): p. 107-13.
506. Epstein, L.H., et al., *The effect of reinforcement or stimulus control to reduce sedentary behavior in the treatment of pediatric obesity.* Health Psychology, 2004. **23**(4): p. 371-380.
507. Epstein, L.H., et al., *Increasing healthy eating vs. reducing high energy-dense foods to treat pediatric obesity.* Obesity, 2008. **16**(2): p. 318-26.
508. Epstein, L.H., et al., *Cost-effectiveness of family-based group treatment for child and parental obesity.* Childhood Obesity, 2014. **10**(2): p. 114-21.
509. Danielsen, Y.S., et al., *Family-based behavioral treatment of obesity-the FABO-study.* Obesity facts, 2015. **8**: p. 234.
510. Skjakodegard, H.F., et al., *Study Protocol: A randomized controlled trial evaluating the effect of family-based behavioral treatment of childhood and adolescent obesity-The FABO-study.* BMC Public Health. **16**(1): p. 1106.
511. Danielsen, Y.S., et al., *Effect of a family-based cognitive behavioural intervention on body mass index, self-esteem and symptoms of depression in children with obesity (aged 7-13): A randomised waiting list controlled trial.* Obesity Research and Clinical Practice, 2013. **7**(2): p. e116-e128.
512. Eren Fidanci, B., N. Akbayrak, and F. Arslan, *Assessment of a Health Promotion Model on Obese Turkish Children.* Journal of Nursing Research. **25**(6): p. 436-446.
513. Fisher, J.O., et al., *Title: Efficacy of a food parenting intervention for mothers with low income to reduce preschooler's solid fat and added sugar intakes: A randomized controlled trial.* International Journal of Behavioral Nutrition and Physical Activity, 2019. **16**(1).
514. Flodmark, C.E., et al., *Prevention of progression to severe obesity in a group of obese schoolchildren treated with family therapy.* Pediatrics, 1993. **91**(5): p. 880-4.
515. Foster, G.D., et al., *A policy-based school intervention to prevent overweight and obesity.* Pediatrics, 2008. **121**(4): p. e794-802.
516. French, S.A., et al., *Multicomponent Obesity Prevention Intervention in Low-Income Preschoolers: primary and Subgroup Analyses of the NET-Works Randomized Clinical Trial, 2012-2017.* American journal of public health, 2018. **108**(12): p. 1695-1706.
517. Boutelle, K.N., et al., *Design of the FRESH study: A randomized controlled trial of a parent-only and parent-child family-based treatment for childhood obesity.* Contemporary Clinical Trials. **45**(Pt B): p. 364-370.
518. Boutelle, K.N., G. Cafri, and S.J. Crow, *Parent-only treatment for childhood obesity: A randomized controlled trial.* Obesity, 2011. **19**(3): p. 574-580.
519. Boutelle, K.N., et al., *Effect of Attendance of the Child on Body Weight, Energy Intake, and Physical Activity in Childhood Obesity Treatment: A Randomized Clinical Trial.* JAMA Pediatrics, 2017. **171**(7): p. 622-628.
520. Gholami, M., et al., *Mothers improve their daughters' vegetable intake: A randomized controlled trial.* Psychology, Health & Medicine, 2015. **20**(1): p. 1-7.

521. Gomez, S.F., et al., *Effect of a community-based childhood obesity intervention program on changes in anthropometric variables, incidence of obesity, and lifestyle choices in Spanish children aged 8 to 10 years.* European journal of pediatrics, 2018. **(no pagination)**.
522. Gomez, S.F., et al., *Study protocol: effects of the THAO-child health intervention program on the prevention of childhood obesity - the POIBC study.* BMC Pediatrics. **14**: p. 215.
523. Gong, L., et al., *Weight loss, inflammatory markers, and improvements of iron status in overweight and obese children.* Journal of Pediatrics, 2014. **164**(4): p. 795-800.e2.
524. Gourlan, M., P. Sarrazin, and D. Trouilloud, *Motivational interviewing as a way to promote physical activity in obese adolescents: A randomised-controlled trial using self-determination theory as an explanatory framework.* Psychology & Health, 2013. **28**(11): p. 1265-1286.
525. Graf, C., et al., *Who benefits from intervention in, as opposed to screening of, overweight and obese children?* Cardiology in the Young, 2006. **16**(5): p. 474-80.
526. Greve, J. and E. Heinesen, *Evaluating the impact of a school-based health intervention using a randomized field experiment.* Economics & Human Biology, 2015. **18**: p. 41-56.
527. Haines, J., et al., *Guelph Family Health Study: pilot study of a home-based obesity prevention intervention.* Canadian Journal of Public Health, 2018. **109**(4): p. 549-560.
528. Krystia, O., et al., *A randomized home-based childhood obesity prevention pilot intervention has favourable effects on parental body composition: Preliminary evidence from the Guelph Family Health Study.* BMC Obesity, 2019. **6**(1).
529. Mirotta, J.A., et al., *Guelph Family Health Study's Home-Based Obesity Prevention Intervention Increases Fibre and Fruit Intake in Preschool-Aged Children.* Revue canadienne de la pratique et de la recherche en dietetique [Canadian journal of dietetic practice and research], 2018. **79**(2): p. 86-90.
530. Haire-Joshu, D., et al., *The use of mentoring programs to improve energy balance behaviors in high-risk children.* Obesity, 2010. **18 Suppl 1**: p. S75-83.
531. Hakanen, M., et al., *Development of overweight in an atherosclerosis prevention trial starting in early childhood. The STRIP study.* International journal of obesity, 2006. **30**(4): p. 618-26.
532. Hakanen, M., et al., *Dietary and lifestyle counselling reduces the clustering of overweight-related cardiometabolic risk factors in adolescents.* Acta Paediatrica, 2010. **99**(6): p. 888-895.
533. Harvey-Berino, J. and J. Rourke, *Obesity prevention in preschool native-american children: a pilot study using home visiting.* Obesity Research, 2003. **11**(5): p. 606-11.
534. Baur, L., *Effectiveness of a home-based early intervention on children's BMI at age two years: Randomised controlled trial.* Obesity Facts, 2012. **5**: p. 34.
535. Wen, L.M., et al., *Early intervention of multiple home visits to prevent childhood obesity in a disadvantaged population: A home-based randomised controlled trial (Healthy Beginnings Trial).* BMC Public Health, 2007. **7**.
536. Wen, L.M., et al., *Sustainability of Effects of an Early Childhood Obesity Prevention Trial Over Time: a Further 3-Year Follow-up of the Healthy Beginnings Trial.* JAMA pediatrics, 2015. **169**(6): p. 543-551.
537. Christie, D., et al., *Effects of a motivational lifestyle intervention (the Healthy Eating and Lifestyle Programme (HELP)) on metabolic outcomes in obese adolescents: findings from a randomized controlled trial.* Pediatric diabetes, 2015. **16**: p. 45-.
538. Christie, D., et al., *RCT of a motivational lifestyle intervention (the healthy eating and lifestyle programme (help)) for obese young people.* Archives of disease in childhood., 2015. **100**: p. A2.
539. Christie, D., et al., *Assessing the efficacy of the Healthy Eating and Lifestyle Programme (HELP) compared with enhanced standard care of the obese adolescent in the community: study protocol for a randomized controlled trial.* Trials, 2011. **12**(1): p. 242-242.

540. Christie, D., et al., *35. Does a Motivational Lifestyle Intervention (the Healthy Eating and Lifestyle Programme (HELP)) Work for Obese Young People.* Journal of Adolescent Health, 2015. **56**: p. S19-S19.
541. Panca, M., et al., *Cost-effectiveness of a community-delivered multicomponent intervention compared with enhanced standard care of obese adolescents: cost-utility analysis alongside a randomised controlled trial (the HELP trial).* BMJ open, 2018. **8**(2): p. e018640.
542. *Christie D, Hudson LD, Kinra S, et al. . A community-based motivational personalised lifestyle intervention to reduce BMI in obese adolescents: results from the Healthy Eating and Lifestyle Programme (HELP) randomised controlled trial. Arch Dis Child 2017;102:695–701. 10.1136/archdischild-2016-311586.*
543. Hidayanty, H., et al., *A social cognitive theory-based programme for eating patterns and sedentary activity among overweight adolescents in Makassar, South Sulawesi : a cluster randomised controlled trial.* Asia Pacific Journal of Clinical Nutrition, 2016. **25**: p. S83-S92.
544. Horodynski, M.A. and M. Stommel, *Nutrition education aimed at toddlers: an intervention study.* Pediatric Nursing, 2005. **31**(5): p. 364-388.
545. Hu, Y., et al., *Effectiveness of a Kindergarten-Based Intervention for Preventing Childhood Obesity.* Pediatrics, 2017. **140**(6).
546. Huang, J.S., et al., *Body image and self-esteem among adolescents undergoing an intervention targeting dietary and physical activity behaviors.* Journal of Adolescent Health, 2007. **40**(3): p. 245-51.
547. Hystad, H.T., et al., *A randomised study on the effectiveness of therapist-led v. self-help parental intervention for treating childhood obesity.* British Journal of Nutrition. **110**(6): p. 1143-50.
548. Campbell, K.J., et al., *The extended Infant Feeding, Activity and Nutrition Trial (InFANT Extend) Program: a cluster-randomized controlled trial of an early intervention to prevent childhood obesity.* BMC Public Health. **16**: p. 166.
549. Walsh, A.D., et al., *Paternal self-efficacy for promoting children's obesity protective diets and associations with children's dietary intakes.* International Journal of Behavioral Nutrition & Physical Activity. **16**(1): p. 53.
550. Adams, E.L., et al., *INSIGHT responsive parenting intervention reduces infant's screen time and television exposure.* International Journal of Behavioral Nutrition & Physical Activity. **15**(1): p. 24.
551. Hohman, E.E., et al., *INSIGHT responsive parenting intervention is associated with healthier patterns of dietary exposures in infants.* Obesity, 2017. **25**(1): p. 185-191.
552. Hohman, E.E., et al., *INSIGHT study parenting intervention to prevent childhood obesity improves patterns of dietary exposures in infants.* FASEB journal, 2016. **30**.
553. Paul, I.M., et al., *Effect of a responsive parenting educational intervention on childhood weight outcomes at 3 years of age: The INSIGHT randomized clinical trial.* JAMA - Journal of the American Medical Association, 2018. **320**(5): p. 461-468.
554. Savage, J.S., et al., *Effect of the INSIGHT responsive parenting intervention on rapid infant weight gain and overweight status at Age 1 year: A randomized clinical trial.* JAMA Pediatrics, 2016. **170**(8): p. 742-749.
555. Jansen, E., S. Mulkens, and A. Jansen, *Tackling childhood overweight: treating parents exclusively is effective.* International journal of obesity, 2011. **35**(4): p. 501-509.
556. Jansen, W., et al., *A school-based intervention to reduce overweight and inactivity in children aged 6-12 years: study design of a randomized controlled trial.* BMC Public Health, 2008. **8**: p. 257.
557. Jancey, J.M., et al., *Dietary outcomes of a community based intervention for mothers of young children: A randomised controlled trial.* International Journal of Behavioral Nutrition & Physical Activity, 2014. **11**: p. 182-198.

558. Miller, A.L., et al., *Enhancing self-regulation as a strategy for obesity prevention in Head Start preschoolers: the growing healthy study.* BMC Public Health, 2012. **12**(1): p. 1040-1040.
559. *Jansen, E. C., Miller, A. L., Lumeng, J. C., Kaciroti, N., Herb, H. E. B., Horodynski, M. A., ... & Peterson, K. E. (2017). Externalizing behavior is prospectively associated with intake of added sugar and sodium among low socioeconomic status preschoolers in a sex-specific manner. International Journal of Behavioral Nutrition and Physical Activity, 14(1), 135.*
560. *Johansson, E., Hagströmer, M., Svensson, V., Ek, A., Forssén, M., Nero, H., & Marcus, C. (2015). Objectively measured physical activity in two-year-old children–levels, patterns and correlates. International Journal of Behavioral Nutrition and Physical Activity, 12(1), p3.*
561. Sobko, T., et al., *A randomised controlled trial for overweight and obese parents to prevent childhood obesity--Early STOPP (STockholm Obesity Prevention Program).* BMC Public Health, 2011. **11**(1): p. 336-336.
562. Jones, M., et al., *Randomized, controlled trial of an Internet-facilitated intervention for reducing binge eating and overweight in adolescents.* Pediatrics, 2008. **121**(3): p. 453-462.
563. Kaufman-Shriqui, V., et al., *Effect of a School-Based Intervention on Nutritional Knowledge and Habits of Low-Socioeconomic School Children in Israel: A Cluster-Randomized Controlled Trial.* Nutrients. **8**(4): p. 234.
564. Kalavainen, M., et al., *Cost-effectiveness of routine and group programs for treatment of obese children.* Pediatrics International, 2009. **51**(5): p. 606-611.
565. Kalavainen, M., M. Korppi, and O. Nuutinen, *Long-term efficacy of group-based treatment for childhood obesity compared with routinely given individual counselling.* International Journal of Obesity, 2011. **35**(4): p. 530-533.
566. Kalavainen, M., et al., *Impact of childhood obesity treatment on body composition and metabolic profile.* World Journal of Pediatrics, 2012. **8**(1): p. 31-37.
567. Kalavainen, M.P., M.O. Korppi, and O.M. Nuutinen, *Clinical efficacy of group-based treatment for childhood obesity compared with routinely given individual counseling.* International Journal of Obesity, 2007. **31**(10): p. 1500-8.
568. Kebaili, R., et al., *School-based intervention to promote healthy nutrition in Sousse, Tunisia.* International Journal of Adolescent Medicine & Health, 2014. **26**(2): p. 253-258.
569. Østbye, T., et al., *The keys to healthy family child care homes (KEYS) intervention study: Design, rationale and baseline characteristics.* Obesity Reviews, 2014. **15**: p. 238.
570. Ostbye, T., et al., *The keys to healthy family child care homes intervention: study design and rationale.* Contemporary Clinical Trials. **40**: p. 81-9.
571. Kharofa, R.Y., et al., *Randomized controlled trial of a Wellness Action Plan to promote healthy diet and activity in pediatric primary care.* Preventive medicine reports, 2015. **2**: p. 899-905.
572. Klesges, R.C., et al., *Memphis Girls health Enrichment Multi-site Studies (GEMS): Phase 2: design and baseline.* Contemporary Clinical Trials, 2008. **29**(1): p. 42-55.
573. Beech, B.M., et al., *Child- and parent-targeted interventions: The Memphis GEMS pilot study.* Ethnicity and Disease, 2003. **13**(1 SUPPL. 1): p. S1-40.
574. Klesges, R.C., et al., *The Memphis Girls' health Enrichment Multi-site Studies (GEMS): An evaluation of the efficacy of a 2-year obesity prevention program in African American girls.* Archives of Pediatrics and Adolescent Medicine, 2010. **164**(11): p. 1007-1014.
575. Kumanyika, S.K., et al., *Phase 1 of the Girls health Enrichment Multi-site Studies (GEMS): conclusion.* Ethnicity & Disease, 2003. **13**(1 Suppl 1): p. S88-91.
576. Obarzanek, E. and C.A. Pratt, *Girls health Enrichment Multi-site Studies (GEMS): new approaches to obesity prevention among young African-American girls.* Ethnicity & Disease, 2003. **13**(1 Suppl 1): p. S1-5.

577. Kokkvoll, A., et al., *Health in overweight children: 2-year follow-up of Finnmark Activity School--a randomised trial.* Archives of Disease in Childhood. **100**(5): p. 441-8.
578. Danielzik, S., S. Pust, and M.J. Müller, *School-based interventions to prevent overweight and obesity in prepubertal children: Process and 4-years outcome evaluation of the Kiel Obesity Prevention Study (KOPS).* Acta Paediatrica, International Journal of Paediatrics, 2007. **96**(SUPPL. 454): p. 19-25.
579. Plachta-Danielzik, S., et al., *Eight-year follow-up of school-based intervention on childhood overweight--the Kiel Obesity Prevention Study.* Obesity Facts. **4**(1): p. 35-43.
580. Plachta-Danielzik, S., et al., *Four-year follow-up of school-based intervention on overweight children: the KOPS study.* Obesity, 2007. **15**(12): p. 3159-69.
581. Kubik, M.Y., et al., *Providing obesity prevention counseling to children during a primary care clinic visit: results from a pilot study.* Journal of the American Dietetic Association, 2008. **108**(11): p. 1902-6.
582. Kulendran, M., et al., *The use of commitment techniques to support weight loss maintenance in obese adolescents.* Psychology & health, 2016. **31**(11): p. 1332-1341.
583. Kulik, N., et al., *Brief report: a randomized controlled trial examining peer support and behavioral weight loss treatment.* Journal of adolescence, 2015. **44**: p. 117-123.
584. Lee, R.M., et al., *Impact of the Out-of-School Nutrition and Physical Activity (OSNAP) Group Randomized Controlled Trial on Children's Food, Beverage, and Calorie Consumption among Snacks Served.* Journal of the Academy of Nutrition & Dietetics. **118**(8): p. 1425-1437.
585. Lee, R.M., et al., *Impact of implementation factors on children's water consumption in the Out-of-School Nutrition and Physical Activity group-randomized trial.* New Directions for Youth Development. **2014**(143): p. 79-101.
586. Creanor, S., et al., *Detailed statistical analysis plan for a cluster randomised controlled trial of the Healthy Lifestyles Programme (HeLP), a novel school-based intervention to prevent obesity in school children.* Trials, 2016. **17**: p. 599-599.
587. Lloyd, J., et al., *Effectiveness of the Healthy Lifestyles Programme (HeLP) to prevent obesity in UK primary-school children: a cluster randomised controlled trial.* The lancet. Child & adolescent health, 2018. **2**(1): p. 35-45.
588. Lloyd, J., et al., *Trial baseline characteristics of a cluster randomised controlled trial of a school-located obesity prevention programme; the Healthy Lifestyles Programme (HeLP) trial.* BMC public health, 2017. **17**(1): p. 291.
589. Lloyd, J., et al., *Intervention fidelity in the definitive cluster randomised controlled trial of the Healthy Lifestyles Programme (HeLP) trial: Findings from the process evaluation.* International Journal of Behavioral Nutrition and Physical Activity, 2017. **14**(1).
590. Lloyd, J.J., et al., *Evidence, theory and context--using intervention mapping to develop a school-based intervention to prevent obesity in children.* International Journal of Behavioral Nutrition & Physical Activity, 2011. **8**: p. 73.
591. Lloyd, J.J., K.M. Wyatt, and S. Creanor, *Behavioural and weight status outcomes from an exploratory trial of the Healthy Lifestyles Programme (HeLP): A novel school-based obesity prevention programme.* BMJ Open, 2012. **2**(3).
592. Wyatt, K. and J. Lloyd, *Development of a novel, school located, obesity prevention programme, the Healthy Lifestyles Programme (HeLP).* Education & Health, 2013. **31**(2): p. 89-95.
593. Wyatt, K.M., et al., *The Healthy Lifestyles Programme (HeLP), a novel school-based intervention to prevent obesity in school children: study protocol for a randomised controlled trial.* Trials [Electronic Resource]. **14**: p. 95.
594. Lochrie, A.S., et al., *Effects of a family based intervention study for overweight children: 6 month medical outcome data.* Diabetes, 2010.

595. Loeb, K.L., et al., *Adapting family-based treatment for paediatric obesity: a randomized controlled pilot trial.* European eating disorders review, 2019. **27**(5): p. 521-530.
596. Looney, S.M. and H.A. Raynor, *Examining the effect of three low-intensity pediatric obesity interventions: A pilot randomized controlled trial.* Clinical Pediatrics, 2014. **53**(14): p. 1367-1374.
597. Love-Osborne, K., et al., *School-based health center-based treatment for obese adolescents: feasibility and body mass index effects.* Childhood Obesity. **10**(5): p. 424-31.
598. Luca, P., et al., *Adolescents with severe obesity: outcomes of participation in an intensive obesity management programme.* Pediatric Obesity. **10**(4): p. 275-82.
599. Lumeng, J.C., et al., *Improving self-regulation for obesity prevention in head start: A randomized controlled trial.* Pediatrics, 2017. **139**(5).
600. Mameli, C., et al., *Combined use of a wristband and a smartphone to reduce body weight in obese children: randomized controlled trial.* Pediatric Obesity. **13**(2): p. 81-87.
601. Markert, J., et al., *Telephone-based adiposity prevention for families with overweight children (T.A.F.F.-Study): One year outcome of a randomized, controlled trial.* International Journal of Environmental Research and Public Health, 2014. **11**(10): p. 10327-10344.
602. Markert, J., et al., *Prevention of childhood obesity: Recruiting strategies via local paediatricians and study protocol for a telephone-based counselling programme.* Obesity Research and Clinical Practice, 2013. **7**(6): p. e476-e486.
603. Mårild, S., et al., *A controlled study of lifestyle treatment in primary care for children with obesity.* Pediatric Obesity, 2013. **8**(3): p. 207-217.
604. Martínez-Andrade, G.O., et al., *Feasibility and impact of Creciendo Sanos, a clinic-based pilot intervention to prevent obesity among preschool children in Mexico City.* BMC Pediatrics, 2014. **14**(1): p. 77-77.
605. McEachan, R.R.C., et al., *The HAPPY (Healthy and Active Parenting Programmme for early Years) feasibility randomised control trial: acceptability and feasibility of an intervention to reduce infant obesity.* BMC Public Health, 2016. **16**(1): p. 1-15.
606. McGarvey, E., et al., *Feasibility and benefits of a parent-focused preschool child obesity intervention.* American Journal of Public Health, 2004. **94**(9): p. 1490-1495.
607. Meng, Y., et al., *Promoting Healthy Diet, Physical Activity, and Life-Skills in High School Athletes: Results from the WAVE Ripples for Change Childhood Obesity Prevention Two-Year Intervention.* Nutrients, 2018. **10**(7): p. 23.
608. Moore, J.B., et al., *Childhood obesity study: a pilot study of the effect of the nutrition education program Color My Pyramid.* Journal of School Nursing, 2009. **25**(3): p. 230-9.
609. Mora, T., E. Llargués, and A. Recasens, *Does health education affect BMI? Evidence from a school-based randomised-control trial.* Economics and Human Biology, 2015. **17**: p. 190-201.
610. Morandi, A., et al., *Prevention of obesity in toddlers: The PROBIT trial.* Obesity Facts, 2018. **11**: p. 58-59.
611. Fornari, E., et al., *The Prevention of Obesity in Toddlers (PROBIT) trial.* Journal of Pediatric Gastroenterology and Nutrition, 2018. **66**: p. 902.
612. Morshed, A.B., et al., *The Impact of a Healthy Weight Intervention Embedded in a Home-Visiting Program on Children's Weight and Mothers' Feeding Practices.* Journal of Nutrition Education & Behavior, 2019. **51**(2): p. 237-244.
613. Munsch, S., et al., *Randomized controlled comparison of two cognitive behavioral therapies for obese children: mother versus mother-child cognitive behavioral therapy.* Psychotherapy & Psychosomatics, 2008. **77**(4): p. 235-46.
614. Muth, N.D., et al., *Making an IMPACT: effect of a school-based pilot intervention.* North Carolina Medical Journal, 2008. **69**(6): p. 432-40.

615. Nardo Junior, N., et al., *Building a response criterion for pediatric multidisciplinary obesity intervention success based on combined benefits.* European Journal of Pediatrics. **177**(6): p. 1-12.
616. Nawi, A.M. and F.I.C. Jamaludin, *Effect of internet-based intervention on obesity among adolescents in Kuala Lumpur: a school-based cluster randomised trial.* Malaysian journal of medical sciences, 2015. **22**(4): p. 47-56.
617. de la Torre, A., et al., *Niños Sanos, Familia Sana: Mexican immigrant study protocol for a multifaceted CBPR intervention to combat childhood obesity in two rural California towns.* BMC Public Health, 2013. **13**(1): p. 1033-1033.
618. Sadeghi, B., et al., *A three-year multifaceted intervention to prevent obesity in children of Mexican-heritage.* BMC Public Health. **19**(1): p. 582.
619. Norman, G., et al., *Outcomes of a 1-year randomized controlled trial to evaluate a behavioral 'stepped-down' weight loss intervention for adolescent patients with obesity.* Pediatric Obesity. **11**(1): p. 18-25.
620. Norman, Å., et al., *Parental support in promoting children's health behaviours and preventing overweight and obesity - A long-term follow-up of the cluster-randomised healthy school start study II trial.* BMC Pediatrics, 2019. **19**(1).
621. Nyberg, G., et al., *Effectiveness of a universal parental support programme to promote health behaviours and prevent overweight and obesity in 6-year-old children in disadvantaged areas, the Healthy School Start Study II, a cluster-randomised controlled trial.* International Journal of Behavioral Nutrition & Physical Activity. **13**: p. 4.
622. Ochoa-Aviles, A., et al., *A school-based intervention improved dietary intake outcomes and reduced waist circumference in adolescents: a cluster randomized controlled trial.* Nutrition Journal. **16**(1): p. 79.
623. Ochoa, A., et al., *Effect of the school-based health promotion intervention activital on dietary intake and waist circumference: a cluster randomized controlled trial.* Annals of nutrition & metabolism, 2017. **71**: p. 1272-1273.
624. Omorou, A.Y., et al., *Adolescents' Physical Activity and Sedentary Behavior: A Pathway in Reducing Overweight and Obesity. The PRALIMAP 2-Year Cluster Randomized Controlled Trial.* Journal of physical activity & health. **12**(5): p. 628-35.
625. Poeta, M., et al., *Waist Circumference and Healthy Lifestyle Preferences/Knowledge Monitoring in a Preschool Obesity Prevention Program.* Nutrients, 2019. **11**(9): p. 2139.
626. Rerksuppaphol, L. and S. Rerksuppaphol, *Internet based obesity prevention program for Thai school children-a randomized control trial.* Journal of Clinical and Diagnostic Research, 2017. **11**(3): p. SC07-SC11.
627. Resnicow, K., et al., *Study design and baseline description of the BMI2 trial: reducing paediatric obesity in primary care practices.* Pediatric Obesity, 2012. **7**(1): p. 3-15.
628. *Resnicow K, Harris D, Wasserman R, Schwartz RP, Perez-Rosas V, Mihalcea R, Snetselaar L. Advances in motivational interviewing for pediatric obesity: results of the brief motivational interviewing to reduce body mass index trial and future directions. Pediatric Clinics. 2016 Jun 1;63(3):539-62.*
629. Reyes-Morales, H., et al., *Effect of an intervention based on child-care centers to reduce risk behaviors for obesity in preschool children.* Boletin medico del hospital infantil de mexico, 2016. **73**(2): p. 75-83.
630. Rohde, J.F., et al., *Effects of the Healthy Start randomized intervention on dietary intake among obesity-prone normal-weight children.* Public Health Nutrition. **20**(16): p. 2988-2997.
631. Saelens, B.E., P. Lozano, and K. Scholz, *A randomized clinical trial comparing delivery of behavioral pediatric obesity treatment using standard and enhanced motivational approaches.* Journal of Pediatric Psychology. **38**(9): p. 954-64.

632. Santos, I.S., et al., *Promotion of weight gain in early childhood does not increase metabolic risk in adolescents: A 15-year follow-up of a cluster-randomized controlled trial.* Journal of Nutrition, 2015. **145**(12): p. 2749-2755.
633. Satoh, A., et al., *Dietary guidance for obese children and their families using a model nutritional balance chart.* Japan Journal of Nursing Science, 2007. **4**(2): p. 95-102.
634. Sen, M., et al., *Examination of a board game approach to children's involvement in family-based weight management vs. traditional family-based behavioral counseling in primary care.* European Journal of Pediatrics. **177**(8): p. 1231-1238.
635. Schwartz, R.P., et al., *Office-based motivational interviewing to prevent childhood obesity: A feasibility study.* Archives of Pediatrics and Adolescent Medicine, 2007. **161**(5): p. 495-501.
636. Sharifi, M., et al., *Cost-effectiveness of a clinical childhood obesity intervention.* Pediatrics, 2017. **140**(5).
637. Taveras, E.M., et al., *Comparative effectiveness of childhood obesity interventions in pediatric primary care: a cluster-randomized clinical trial.* JAMA pediatrics, 2015. **169**(6): p. 535-542.
638. *Sousa, P., Fonseca, H., Gaspar, P., & Gaspar, F. (2015). Controlled trial of an Internet-based intervention for overweight teens (Next. Step): effectiveness analysis. European journal of pediatrics, 174(9), 1143-1157.*
639. Sousa, P., et al., *Internet-based intervention programme for obese adolescents and their families (Next.Step): research protocol of a controlled trial.* Journal of Advanced Nursing (John Wiley & Sons, Inc.), 2014. **70**(4): p. 904-914.
640. Spiegel, S.A. and D. Foulk, *Reducing overweight through a multidisciplinary school-based intervention.* Obesity, 2006. **14**(1): p. 88-96.
641. Small, L., et al., *A Pilot Intervention Designed to Address Behavioral Factors That Place Overweight/Obese Young Children at Risk for Later-Life Obesity.* Western Journal of Nursing Research, 2017. **39**(8): p. 1192-1212.
642. Stock, S., et al., *Healthy Buddies: a novel, peer-led health promotion program for the prevention of obesity and eating disorders in children in elementary school.* Pediatrics, 2007. **120**(4): p. e1059-68.
643. Stookey, J.D., et al., *Healthy apple program to support child care centers to alter nutrition and physical activity practices and improve child weight: a cluster randomized trial.* BMC Public Health. **17**(1): p. 965.
644. *Tanofsky-Kraff M, Shomaker LB, Wilfley DE, Young JF, Sbrocco T, Stephens M, Ranzenhofer LM, Elliott C, Brady S, Radin RM, Vannucci A. Targeted prevention of excess weight gain and eating disorders in high-risk adolescent girls: a randomized controlled trial. The American journal of clinical nutrition. 2014 Oct 1;100(4):1010-8.*
645. Burke, N.L., et al., *Impact of age and race on outcomes of a program to prevent excess weight gain and disordered eating in adolescent girls.* Nutrients, 2017. **9**(9) (no pagination).
646. Tanofsky-Kraff, M., et al., *Excess weight gain prevention in adolescents: three-year outcome following a randomized controlled trial.* Journal of consulting and clinical psychology, 2017. **85**(3): p. 218-227.
647. Tanofsky-Kraff, M., et al., *A pilot study of interpersonal psychotherapy for preventing excess weight gain in adolescent girls at-risk for obesity.* International Journal of Eating Disorders, 2010. **43**(8): p. 701-6.
648. Taveras, E.M., et al., *Comparative effectiveness of clinical-community childhood obesity interventions a randomized clinical trial.* JAMA Pediatrics, 2017. **171**(8).
649. Taveras, E.M., et al., *Connect for Health: design of a clinical-community childhood obesity intervention testing best practices of positive outliers.* Contemporary clinical trials, 2015. **45**(Pt B): p. 287-295.

650. Thompson, D., et al., *Creating action plans in a serious video game increases and maintains child fruit-vegetable intake: a randomized controlled trial.* Int J Behav Nutr Phys Act, 2015. **12**: p. 39.
651. Thakur, J.S., et al., *Impact of 20 Week Lifestyle Intervention Package on Anthropometric Biochemical and Behavioral Characteristics of Schoolchildren in North India.* Journal of tropical pediatrics, 2016. **62**(5): p. 368-376.
652. Taylor, R.W., et al., *A Tailored Family-Based Obesity Intervention: a Randomized Trial.* Pediatrics, 2015. **136**(2): p. 281-289.
653. Adams, A., et al., *Healthy children, strong families: Results of a randomized trial of obesity prevention for preschool American Indian children and their families.* Obesity, 2011. **19**: p. S110.
654. Tomayko, E.J., et al., *Healthy Children, Strong Families 2: A randomized controlled trial of a healthy lifestyle intervention for American Indian families designed using community-based approaches.* Clinical Trials. **14**(2): p. 152-161.
655. *Tomayko, E. J., Prince, R. J., Cronin, K. A., Kim, K., Parker, T., & Adams, A. K. (2019). The Healthy Children, Strong Families 2 (HCSF2) Randomized Controlled Trial Improved Healthy Behaviors in American Indian Families with Young Children. Current Developments in Nutrition, 3(Supplement_2), 53-62.*
656. Tucker, J.M., et al., *Evaluation of a Primary Care Weight Management Program in Children Aged 2-5 years: Changes in Feeding Practices, Health Behaviors, and Body Mass Index.*
657. Tyler, D.O. and S.D. Horner, *A primary care intervention to improve weight in obese children: A feasibility study.* Journal of the American Association of Nurse Practitioners, 2016. **28**(2): p. 98-106.
658. van Grieken, A., et al., *Personalized Web-Based Advice in Combination With Well-Child Visits to Prevent Overweight in Young Children: Cluster Randomized Controlled Trial.* Journal of Medical Internet Research, 2017. **19**(7): p. 1-15.
659. Raat, H., et al., *Primary prevention of overweight in preschool children, the BeeBOFT study (breastfeeding, breakfast daily, outside playing, few sweet drinks, less TV viewing): design of a cluster randomized controlled trial.* BMC Public Health. **13**: p. 974.
660. Wadolowska, L., et al., *Changes in Sedentary and Active Lifestyle, Diet Quality and Body Composition Nine Months after an Education Program in Polish Students Aged 11⁻12 Years: report from the ABC of Healthy Eating Study.* Nutrients, 2019. **11**(2).
661. Wald, E.R., et al., *Treating childhood obesity in primary care.* Clinical Pediatrics, 2011. **50**(11): p. 1010-7.
662. Wald, E.R., et al., *An Interactive Web-Based Intervention to Achieve Healthy Weight in Young Children.* Clinical pediatrics, 2018. **57**(5): p. 547-557.
663. Waling, M. and C. Larsson, *Improved dietary intake among overweight and obese children followed from 8 to 12 years of age in a randomised controlled trial.* Journal of Nutritional Science, 2012. **1**.
664. Walpole, B., et al., *Motivational interviewing to enhance self-efficacy and promote weight loss in overweight and obese adolescents: a randomized controlled trial.* Journal of Pediatric Psychology. **38**(9): p. 944-53.
665. Warren, J.M., et al., *Evaluation of a pilot school programme aimed at the prevention of obesity in children.* Health Promotion International, 2003. **18**(4): p. 287-296.
666. Wylie-Rosett, J., et al., *Embedding weight management into safety-net pediatric primary care: randomized controlled trial.* International journal of behavioral nutrition and physical activity, 2018. **15**(1): p. 12.
667. Yackobovitch-Gavan, M., et al., *Intervention for childhood obesity based on parents only or parents and child compared with follow-up alone.* Pediatric Obesity. **13**(11): p. 647-655.
668. Zimmerman, F.J., et al., *The value of social-cognitive theory to reducing preschool TV viewing: A pilot randomized trial.* Preventive Medicine, 2012. **54**(3/4): p. 212-218.

669. Aceves-Martins, M., et al., *A School-Based, Peer-Led, Social Marketing Intervention To Engage Spanish Adolescents in a Healthy Lifestyle ("We Are Cool"-Som la Pera Study): A Parallel-Cluster Randomized Controlled Study.* Childhood Obesity. **13**(4): p. 300-313.
670. Alberga, A.S., et al., *Effects of aerobic and resistance training on abdominal fat, apolipoproteins and high-sensitivity C-reactive protein in adolescents with obesity: the HEARTY randomized clinical trial.* Int J Obes (Lond), 2015. **39**(10): p. 1494-500.
671. Alberga, A.S., et al., *Healthy Eating, Aerobic and Resistance Training in Youth (HEARTY): study rationale, design and methods.* Contemporary Clinical Trials. **33**(4): p. 839-47.
672. Alberga, A.S., et al., *Effects of aerobic training, resistance training, or both on cardiorespiratory and musculoskeletal fitness in adolescents with obesity: the HEARTY trial.* Physiologie appliquee, nutrition ET metabolisme [applied physiology, nutrition, and metabolism], 2016. **41**(3): p. 255-265.
673. Alberga, A.S., et al., *Understanding low adherence to an exercise program for adolescents with obesity: the HEARTY trial.* Obesity Science and Practice, 2019. **5**(5): p. 437-448.
674. Cameron, J.D., et al., *The mediating role of energy intake on the relationship between screen time behaviour and body mass index in adolescents with obesity: The HEARTY study.* Appetite. **107**: p. 437-444.
675. Cameron, J.D., et al., *Body composition and energy intake - skeletal muscle mass is the strongest predictor of food intake in obese adolescents: The HEARTY trial.* Applied Physiology, Nutrition, & Metabolism = Physiologie Appliquee, Nutrition et Metabolisme. **41**(6): p. 611-7.
676. Goldfield, G.S., et al., *Effects of aerobic training, resistance training or both on health-related quality of life in adolescents with obesity: the HEARTY trial.* Canadian journal of diabetes., 2015. **39**: p. S18.
677. Goldfield, G.S., et al., *Effects of aerobic training, resistance training, or both on psychological health in adolescents with obesity: the HEARTY randomized controlled trial.* Journal of consulting and clinical psychology, 2015. **83**(6): p. 1123-1135.
678. Goldfield, G.S., et al., *Effects of aerobic or resistance training or both on health-related quality of life in youth with obesity: the HEARTY Trial.* Applied Physiology, Nutrition, & Metabolism = Physiologie Appliquee, Nutrition et Metabolisme. **42**(4): p. 361-370.
679. Goldfield, G.S., et al., *Effects of aerobic training, resistance training, or both on brain-derived neurotrophic factor in adolescents with obesity: the hearty randomized controlled trial.* Physiology & behavior, 2018. **191**: p. 138-145.
680. Walsh, J.J., et al., *Changes in the Brain-Derived Neurotrophic Factor Are Associated with Improvements in Diabetes Risk Factors after Exercise Training in Adolescents with Obesity: the HEARTY Randomized Controlled Trial.* Neural plasticity, 2018. **2018**: p. 7169583.
681. Alberga, A.S., et al., *Does exercise training affect resting metabolic rate in adolescents with obesity?* Physiologie appliquee, nutrition et metabolisme [Applied physiology, nutrition, and metabolism], 2017. **42**(1): p. 15-22.
682. Aperman-Itzhak, T., et al., *School-Based Intervention to Promote a Healthy Lifestyle and Obesity Prevention Among Fifth- and Sixth-Grade Children.* American Journal of Health Education, 2018. **49**(5): p. 289-295.
683. Boudreau, A.D.A., et al., *Latino families, primary care, and childhood obesity: a randomized controlled trial.* American journal of preventive medicine, 2013. **44**(3): p. S247-S257.
684. Ariza, C., et al., *The Incidence of Obesity, Assessed as Adiposity, Is Reduced After 1 Year in Primary Schoolchildren by the POIBA Intervention.* Journal of nutrition, 2019. **149**(2): p. 258-269.
685. Banks, J., et al., *Evaluating the transferability of a hospital-based childhood obesity clinic to primary care: a randomised controlled trial.* Br J Gen Pract, 2012. **62**(594): p. e6-e12.
686. Barkin, S.L., et al., *Culturally tailored, family-centered, behavioral obesity intervention for Latino-American preschool-aged children.* Pediatrics, 2012. **130**(3): p. 445-456.

687. Barnett, A.W., *Structured Physical Activity and Dietary Education Program for Obese Adolescents: An Evaluation of a Quality Improvement Project at a Rural Primary Care Clinic.* Structured Physical Activity & Dietary Education Program for Obese Adolescents: An Evaluation of a Quality Improvement Project at a Rural Primary Care Clinic, 2017: p. 1-1.
688. Bayer, O., et al., *Short-and mid-term effects of a setting based prevention program to reduce obesity risk factors in children: a cluster-randomized trial.* Clinical Nutrition, 2009. **28**(2): p. 122-128.
689. Berntsen, S., et al., *Obese children playing towards an active lifestyle.* International Journal of Pediatric Obesity, 2010. **5**(1): p. 64-71.
690. Bharath, L.P., et al., *Combined resistance and aerobic exercise training reduces insulin resistance and central adiposity in adolescent girls who are obese: randomized clinical trial.* European Journal of Applied Physiology. **118**(8): p. 1653-1660.
691. Bhave, S., et al., *Effectiveness of a 5-year school-based intervention programme to reduce adiposity and improve fitness and lifestyle in Indian children; the SYM-KEM study.* Archives of disease in childhood, 2016. **101**(1): p. 33-41.
692. Bonis, M., et al., *Improving physical activity in daycare interventions.* Childhood Obesity, 2014. **10**(4): p. 334-341.
693. Brandstetter, S., et al., *Overweight prevention implemented by primary school teachers: a randomised controlled trial.* Obesity facts, 2012. **5**(1): p. 1-11.
694. Kesztyüs, D., et al., *Economic evaluation of URMEL-ICE, a school-based overweight prevention programme comprising metabolism, exercise and lifestyle intervention in children.* The European Journal of Health Economics, 2013. **14**(2): p. 185-195.
695. Bruyndonckx, L., et al., *Diet, exercise, and endothelial function in obese adolescents.* Pediatrics, 2015. **135**(3): p. e653-e661.
696. Burguera, B., et al., *ACTYBOSS: activity, behavioral therapy in young subjects–after-school intervention pilot project on obesity prevention.* Obesity facts, 2011. **4**(5): p. 400-406.
697. Caballero, B., et al., *Pathways: a school-based, randomized controlled trial for the prevention of obesity in American Indian schoolchildren.* The American journal of clinical nutrition, 2003. **78**(5): p. 1030-1038.
698. Davis, S.M., et al., *Pathways curriculum and family interventions to promote healthful eating and physical activity in American Indian schoolchildren.* Preventive Medicine, 2003. **37**: p. S24-34.
699. Going, S., et al., *The effects of the Pathways Obesity Prevention Program on physical activity in American Indian children.* Preventive Medicine. **37**(6 Pt 2): p. S62-9.
700. Going, S.B., *Physical activity measurements: lessons learned from the Pathways study.* Journal of Public Health Management & Practice, 2010. **16**(5): p. 420-425.
701. Going, S.B., et al., *Physical activity assessment in American Indian schoolchildren in the Pathways study.* American Journal of Clinical Nutrition, 1999. **69**(4 Suppl): p. 788S-795S.
702. Harnack, L., et al., *Intervention-related bias in reporting of food intake by fifth-grade children participating in an obesity prevention study.* 2004. **1**(11): p. 1117-21.
703. Himes, J.H., et al., *Impact of the Pathways intervention on dietary intakes of American Indian schoolchildren.* Preventive Medicine. **37**(6 Pt 2): p. S55-61.
704. Stevens, J., et al., *The impact of the Pathways intervention on psychosocial variables related to diet and physical activity in American Indian schoolchildren.* Preventive Medicine. **37**(6 Pt 2): p. S70-9.
705. Stone, E.J., et al., *Design, implementation, and quality control in the Pathways American-Indian multicenter trial.* Preventive Medicine. **37**(6 Pt 2): p. S13-23.
706. Story, M., et al., *Changes in the nutrient content of school lunches: Results from the Pathways study.* Preventive Medicine, 2003. **37**(SUPPL. 1): p. S35-S45.

707. Campbell, K.J., et al., *A parent-focused intervention to reduce infant obesity risk behaviors: a randomized trial.* Pediatrics, 2013. **131**(4): p. 652-660.

708. Centis, E., et al., *A controlled, class-based multicomponent intervention to promote healthy lifestyle and to reduce the burden of childhood obesity.* Pediatric obesity, 2012. **7**(6): p. 436-445.

709. Cezard, G., et al., *Adiposity and response to an obesity prevention intervention in Pakistani and Bangladeshi primary school boys and girls: a secondary analysis using the BEACHeS feasibility study.* BMJ open, 2016. **6**(2).

710. Adab, P., et al., *Preventing childhood obesity, phase II feasibility study focusing on South Asians: BEACHeS.* BMJ Open, 2014. **4**(4).

711. Cohen, J.F., et al., *The CHANGE study: a healthy-lifestyles intervention to improve rural children's diet quality.* Journal of the Academy of Nutrition and Dietetics, 2014. **114**(1): p. 48-53.

712. Cohen, T.R., et al., *A family-centered lifestyle intervention for obese six-to eight-year-old children: Results from a one-year randomized controlled trial conducted in Montreal, Canada.* Canadian Journal of Public Health, 2016. **107**(4-5): p. e453-e460.

713. Cohen, T., et al., *Bone Health is Maintained, While Fat Mass is Reduced in Pre-pubertal Children with Obesity Participating in a 1-Year Family-Centered Lifestyle Intervention.* Calcified Tissue International, 2017. **101**(6): p. 612-622.

714. Cohen, T.R., et al., *A family-centered lifestyle intervention to improve body composition and bone mass in overweight and obese children 6 through 8 years: a randomized controlled trial study protocol.* BMC Public Health. **13**: p. 383.

715. Cohen, T.R., et al., *Changes in eating behavior and plasma leptin in children with obesity participating in a family-centered lifestyle intervention.* Appetite. **125**: p. 81-89.

716. Cohen, T.R., et al., *Changes in lean mass and bone parameters in obese children participating in a familycentered lifestyle intervention: Results from a 1-year RCT.* FASEB Journal, 2017. **31**(1).

717. Coppins, D., et al., *Effectiveness of a multi-disciplinary family-based programme for treating childhood obesity (The Family Project).* European journal of clinical nutrition, 2011. **65**(8): p. 903-909.

718. Crespo, N.C., et al., *Results of a multi-level intervention to prevent and control childhood obesity among Latino children: the Aventuras Para Niños Study.* Annals of Behavioral Medicine, 2012. **43**(1): p. 84-100.

719. Ayala, G.X., et al., *A restaurant-based intervention to promote sales of healthy children's menu items: the Kids' Choice Restaurant Program cluster randomized trial.* BMC Public Health. **16**: p. 250.

720. Da Silva, L., et al., *The effectiveness of a physical activity and nutrition education program in the prevention of overweight in schoolchildren in Criciúma, Brazil.* European journal of clinical nutrition, 2013. **67**(11): p. 1200-1204.

721. Davis, J.N., et al., *Aerobic and strength training reduces adiposity in overweight Latina adolescents.* Medicine and science in sports and exercise, 2009. **41**(7): p. 1494.

722. Davis, S.M., et al., *CHILE: Outcomes of a group randomized controlled trial of an intervention to prevent obesity in preschool Hispanic and American Indian children.* Preventive medicine, 2016. **89**: p. 162-168.

723. Cruz, T.H., et al., *Engagement, recruitment, and retention in a trans-community, randomized controlled trial for the prevention of obesity in rural American Indian and Hispanic children.* Journal of Primary Prevention. **35**(3): p. 135-49.

724. Cruz, T.H., et al., *Effects of an Obesity Prevention Intervention on Physical Activity Among Preschool Children.* Health Promotion Practice, 2016. **17**(5): p. 693-701.

725. Cruz, T.H., et al., *Effects of an Obesity Prevention Intervention on Physical Activity Among Preschool Children: The CHILE Study.* Health Promotion Practice. **17**(5): p. 693-701.

726. Davis, S.M., et al., *CHILE: an evidence-based preschool intervention for obesity prevention in Head Start.* Journal of School Health. **83**(3): p. 223-9.
727. Morshed, A.B., et al., *The Impact of the CHILE Intervention on the Food Served in Head Start Centers in Rural New Mexico.* Journal of school health, 2016. **86**(6): p. 414-423.
728. Davison, K.K., et al., *Evaluation overview for the Massachusetts childhood obesity research demonstration (MA-CORD) project.* Childhood Obesity, 2015. **11**(1): p. 23-36.
729. Blaine, R.E., et al., *Using School Staff Members to Implement a Childhood Obesity Prevention Intervention in Low-Income School Districts: the Massachusetts Childhood Obesity Research Demonstration (MA-CORD Project), 2012-2014.* Preventing chronic disease. **14**: p. E03.
730. Taveras, E.M., et al., *Design of the Massachusetts Childhood Obesity Research Demonstration (MA-CORD) Study.* Childhood Obesity, 2015. **11**(1): p. 11-22.
731. Taveras, E.M., et al., *Clinical effectiveness of the massachusetts childhood obesity research demonstration initiative among low-income children.* Obesity. **25**(7): p. 1159-1166.
732. Woo Baidal, J.A., et al., *Childhood obesity prevention in the women, infants, and children program: Outcomes of the MA-CORD study.* Obesity. **25**(7): p. 1167-1174.
733. De Coen, V., et al., *Effects of a 2-year healthy eating and physical activity intervention for 3–6-year-olds in communities of high and low socio-economic status: the POP (Prevention of Overweight among Pre-school and school children) project.* Public health nutrition, 2012. **15**(9): p. 1737-1745.
734. De Henauw, S., et al., *Effects of a community-oriented obesity prevention programme on indicators of body fatness in preschool and primary school children. Main results from the IDEFICS study.* Obesity reviews, 2015. **16**: p. 16-29.
735. Ahrens, W., et al., *Understanding and preventing childhood obesity and related disorders--IDEFICS: a European multilevel epidemiological approach.* Nutrition Metabolism & Cardiovascular Diseases, 2006. **16**(4): p. 302-8.
736. Arvidsson, L., et al., *Fat, sugar and water intakes among families from the IDEFICS intervention and control groups: First observations from I.Family.* Obesity Reviews, 2015. **16**: p. 127-137.
737. Bel-Serrat, S., et al., *Food consumption and cardiovascular risk factors in European children: the IDEFICS study.* Pediatric Obesity. **8**(3): p. 225-36.
738. de Bourdeaudhuij, I., et al., *Behavioural effects of a community-oriented setting-based intervention for prevention of childhood obesity in eight European countries. Main results from the IDEFICS study.* Obesity reviews, 2015. **16**: p. 30-40.
739. de Bourdeaudhuij, I., et al., *Implementation of the IDEFICS intervention across European countries: Perceptions of parents and relationship with BMI.* Obesity Reviews, 2015. **16**: p. 78-88.
740. De Henauw, S., et al., *The IDEFICS community-oriented intervention programme: a new model for childhood obesity prevention in Europe?* International Journal of Obesity, 2011. **35**: p. S16-23.
741. De Miguel-Etayo, P., et al., *Physical fitness reference standards in European children: the IDEFICS study.* International Journal of Obesity, 2014. **38**: p. S57-66.
742. De Miguel-Etayo, P., et al., *Physical fitness reference standards in European children: the IDEFICS study.* International journal of obesity. **38 Suppl 2**: p. S57-66.
743. Dello Russo, M., et al., *The Impact of Adding Sugars to Milk and Fruit on Adiposity and Diet Quality in Children: A Cross-Sectional and Longitudinal Analysis of the Identification and Prevention of Dietary- and Lifestyle-Induced Health Effects in Children and Infants (IDEFICS) Study.* Nutrients, 2018. **10**(10): p. 21.
744. Donatiello, E., et al., *Physical activity, adiposity and urbanization level in children: results for the Italian cohort of the IDEFICS study.* Public Health (Elsevier), 2013. **127**(8): p. 761-765.
745. Fernandez-Alvira, J.M., et al., *Parental education and frequency of food consumption in European children: the IDEFICS study.* Public Health Nutrition. **16**(3): p. 487-98.

746. Formisano, A., et al., *Family structure and childhood obesity: results of the IDEFICS Project.* Public Health Nutrition. **17**(10): p. 2307-15.
747. Hunsberger, M., et al., *Infant feeding practices and prevalence of obesity in eight European countries - the IDEFICS study.* Public Health Nutrition. **16**(2): p. 219-27.
748. Iguacel, I., et al., *Prospective associations between social vulnerabilities and children's weight status. Results from the IDEFICS study.* International journal of obesity. **42**(10): p. 1691-1703.
749. Kovács, E., et al., *Adherence to the obesity-related lifestyle intervention targets in the IDEFICS study.* International Journal of Obesity, 2014. **38**: p. S144-51.
750. Lissner, L., et al., *Differential outcome of the IDEFICS intervention in overweight versus non-overweight children: Did we achieve 'primary' or 'secondary' prevention?* Obesity Reviews, 2015. **16**: p. 119-126.
751. Michels, N., et al., *Effect of the IDEFICS multilevel obesity prevention on children's sleep duration.* Obesity Reviews, 2015. **16**: p. 68-77.
752. Nappo, A., et al., *High-sensitivity C-reactive protein is a predictive factor of adiposity in children: results of the identification and prevention of dietary- and lifestyle-induced health effects in children and infants (IDEFICS) study.* Journal of the American Heart Association. **2**(3): p. e000101.
753. Papoutsou, S., et al., *Timing of solid food introduction and association with later childhood overweight and obesity: The IDEFICS study.* Maternal & Child Nutrition, 2018. **14**(1): p. 01.
754. Pigeot, I., et al., *The IDEFICS intervention trial to prevent childhood obesity: Design and study methods.* Obesity Reviews, 2015. **16**: p. 4-15.
755. Pigeot, I., S. de Henauw, and T. Baranowski, *The IDEFICS (Identification and prevention of Dietary- and lifestyle-induced health EFfects In Children and infantS) trial outcomes and process evaluations.* Obesity Reviews. **16 Suppl 2**: p. 2-3.
756. Santaliestra-Pasias, A.M., et al., *Food and beverage intakes according to physical activity levels in European children: the IDEFICS (Identification and prevention of Dietary and lifestyle induced health EFfects In Children and infantS) study.* Public Health Nutrition. **21**(9): p. 1717-1725.
757. Santaliestra-Pasias, A.M., et al., *Clustering of lifestyle behaviours and relation to body composition in European children. The IDEFICS study.* European Journal of Clinical Nutrition. **69**(7): p. 811-6.
758. Suling, M., et al., *Design and results of the pretest of the IDEFICS study.* International Journal of Obesity, 2011. **35**: p. S30-44.
759. Svensson, Å., et al., *European children's sugar intake on weekdays versus weekends: The IDEFICS study.* European Journal of Clinical Nutrition, 2014. **68**(7): p. 822-828.
760. Tognon, G., et al., *Adherence to a Mediterranean-like dietary pattern in children from eight European countries. The IDEFICS study.* International Journal of Obesity, 2014. **38**: p. S108-14.
761. Tubic, B., et al., *Relation between bone mineral density, biological markers and anthropometric measures in 4-year-old children: a pilot study within the IDEFICS study.* International Journal of Obesity, 2011. **35**: p. S119-24.
762. Verbestel, V., et al., *Effectiveness of the IDEFICS intervention on objectively measured physical activity and sedentary time in European children.* Obesity Reviews, 2015. **16**: p. 57-67.
763. Verbestel, V., et al., *Using the intervention mapping protocol to develop a community-based intervention for the prevention of childhood obesity in a multi-centre European project: The IDEFICS intervention.* The International Journal of Behavioral Nutrition and Physical Activity Vol 8 2011, ArtID 82, 2011. **8**.
764. Zaqout, M., et al., *Influence of physical fitness on cardio-metabolic risk factors in European children. The IDEFICS study.* International Journal of Obesity. **40**(7): p. 1119-25.
765. de Niet, J., et al., *The effect of a short message service maintenance treatment on body mass index and psychological well-being in overweight and obese children: a randomized controlled trial.* Pediatric obesity, 2012. **7**(3): p. 205-219.

766. de Niet, J., et al., *Short message service reduces dropout in childhood obesity treatment: a randomized controlled trial.* Health Psychology. **31**(6): p. 797-805.
767. de Silva-Sanigorski, A.M., et al., *Reducing obesity in early childhood: results from Romp & Chomp, an Australian community-wide intervention program.* The American journal of clinical nutrition, 2010. **91**(4): p. 831-840.
768. de Silva-Sanigorski, A.M., et al., *Process and impact evaluation of the Romp & Chomp obesity prevention intervention in early childhood settings: lessons learned from implementation in preschools and long day care settings.* Childhood Obesity. **8**(3): p. 205-15.
769. Dewar, D.L., et al., *The nutrition and enjoyable activity for teen girls study: a cluster randomized controlled trial.* American journal of preventive medicine, 2013. **45**(3): p. 313-317.
770. Lubans, D.R., et al., *The Nutrition and Enjoyable Activity for Teen Girls (NEAT girls) randomized controlled trial for adolescent girls from disadvantaged secondary schools: rationale, study protocol, and baseline results.* BMC Public Health, 2010. **10**(1): p. 652.
771. Collins, C.E., et al., *12 month changes in dietary intake of adolescent girls attending schools in low-income communities following the NEAT Girls cluster randomized controlled trial.* Appetite, 2014. **73**: p. 147-155.
772. Dewar, D.L., et al., *Exploring changes in physical activity, sedentary behaviors and hypothesized mediators in the NEAT girls group randomized controlled trial.* Journal of Science & Medicine in Sport, 2014. **17**(1): p. 39-46.
773. Lubans, D., et al., *Two-year outcomes from the NEAT Girls obesity prevention cluster randomized controlled trial.* Journal of Science and Medicine in Sport, 2013. **16**: p. e34.
774. Lubans, D., et al., *Preventing obesity among adolescent girls in lowincome secondary schools: One-year outcomes of the NEAT Girls cluster randomized controlled trial.* Obesity Research and Clinical Practice, 2012. **6**: p. 39.
775. Lubans, D., et al., *Preventing obesity among adolescent girls: Outcomes of the nutrition and enjoyable activity for teen girls cluster randomized controlled trial.* Journal of Science and Medicine in Sport, 2012. **15**: p. S332.
776. Lubans, D.R., et al., *Two year outcomes and moderators of intervention effects from the NEAT Girls obesity prevention group randomised controlled trial.* Obesity Research and Clinical Practice, 2013. **7**: p. e36-e37.
777. Lubans, D.R., et al., *Preventing obesity among adolescent girls: One-year outcomes of the nutrition and enjoyable activity for teen girls (NEAT Girls) cluster randomized controlled trial.* Archives of Pediatrics and Adolescent Medicine, 2012. **166**(9): p. 821-827.
778. Díaz, R.G., et al., *Lifestyle intervention in primary care settings improves obesity parameters among Mexican youth.* Journal of the American Dietetic Association, 2010. **110**(2): p. 285-290.
779. Dietrich, S., et al., *Obesity intervention on the healthy lifestyle in childhood: results of the PRESTO (PrEvention STudy of Obesity) Study.* Italian Journal of Public Health, 2008. **5**(1).
780. Damon, S., S. Dietrich, and K. Widhalm, *PRESTO--Prevention Study of Obesity: a project to prevent obesity during childhood and adolescence.* Acta Paediatrica Supplement. **94**(448): p. 47-8.
781. Donnelly, J.E., et al., *Nutrition and physical activity program to attenuate obesity and promote physical and metabolic fitness in elementary school children.* Obesity Research, 1996. **4**(3): p. 229-243.
782. Duggins, M., et al., *Impact of family YMCA membership on childhood obesity: a randomized controlled effectiveness trial.* The Journal of the American Board of Family Medicine, 2010. **23**(3): p. 323-333.
783. Economos, C.D., et al., *A community intervention reduces BMI z-score in children: Shape Up Somerville first year results.* Obesity, 2007. **15**(5): p. 1325-1336.

784. Economos, C.D., et al., *Shape Up Somerville two-year results: A community-based environmental change intervention sustains weight reduction in children.* Preventive Medicine, 2013. **57**(4): p. 322-327.
785. Folta, S.C., et al., *Assessing the use of school public address systems to deliver nutrition messages to children: Shape up Somerville--audio adventures.* Journal of School Health, 2006. **76**(9): p. 459-484.
786. Folta, S.C., et al., *Changes in diet and physical activity resulting from the Shape Up Somerville community intervention.* BMC Pediatrics. **13**: p. 157.
787. Hatfield, D.P., et al., *The critical role of communications in a multilevel obesity-prevention intervention: Lessons learned for alcohol educators.* Patient Education & Counseling. **100 Suppl 1**: p. S3-S10.
788. Elder, J.P., et al., *Childhood obesity prevention and control in city recreation centres and family homes: the MOVE/me M uevo P roject.* Pediatric obesity, 2014. **9**(3): p. 218-231.
789. Eliakim, A., et al., *The effect of a combined intervention on body mass index and fitness in obese children and adolescents–a clinical experience.* European journal of pediatrics, 2002. **161**(8): p. 449-454.
790. Epstein, L.H., et al., *Effects of mastery criteria and contingent reinforcement for family-based child weight control.* Addictive Behaviors, 1994. **19**(2): p. 135-145.
791. Epstein, L.H., et al., *Decreasing sedentary behaviors in treating pediatric obesity.* Archives of pediatrics & adolescent medicine, 2000. **154**(3): p. 220-226.
792. Epstein, L.H., et al., *The challenge of identifying behavioral alternatives to food: clinic and field studies.* Annals of Behavioral Medicine, 2005. **30**(3): p. 201-209.
793. Epstein, L.H., et al., *Reducing variety enhances effectiveness of family-based treatment for pediatric obesity.* Eating behaviors, 2015. **17**: p. 140-143.
794. Epstein, L.H., et al., *Ten-year follow-up of behavioral, family-based treatment for obese children.* Jama, 1990. **264**(19): p. 2519-2523.
795. Fairclough, S.J., et al., *Promoting healthy weight in primary school children through physical activity and nutrition education: a pragmatic evaluation of the CHANGE! randomised intervention study.* BMC public health, 2013. **13**(1): p. 626.
796. Boddy, L.M., et al., *Using formative research to develop the healthy eating component of the CHANGE! school-based curriculum intervention.* BMC Public Health. **12**: p. 710.
797. Fitzgibbon, M.L., et al., *Two-year follow-up results for Hip-Hop to Health Jr.: a randomized controlled trial for overweight prevention in preschool minority children.* The Journal of pediatrics, 2005. **146**(5): p. 618-625.
798. Fitzgibbon, M.L., et al., *A community-based obesity prevention program for minority children: Rationale and study design for Hip-Hop to Health Jr.* Preventive Medicine, 2002. **34**(2): p. 289-297.
799. Fitzgibbon, M.L., et al., *Hip-Hop to Health Jr. for Latino preschool children.* Obesity, 2006. **14**(9): p. 1616-25.
800. Fitzgibbon, M.L., et al., *Hip-hop to health Jr. Obesity prevention effectiveness trial: Postintervention results.* Obesity, 2011. **19**(5): p. 994-1003.
801. Kong, A., et al., *Hip-Hop to Health Jr. Randomized Effectiveness Trial: 1-Year Follow-up Results.* American journal of preventive medicine, 2016. **50**(2): p. 136-144.
802. Stolley, M.R., et al., *Hip-Hop to Health Jr., an obesity prevention program for minority preschool children: baseline characteristics of participants.* Preventive Medicine. **36**(3): p. 320-9.
803. Fitzgibbon, M., et al., *Adaptation and Dissemination of an Evidence-Based Obesity Prevention Intervention.* Journal of Nutrition Education & Behavior, 2013. **45**(4 Supplement): p. S81-S81.
804. Fitzgibbon, M.L., et al., *Family-based hip-hop to health: outcome results.* Obesity (19307381), 2013. **21**(2): p. 274-283.

805. Flattum, C., et al., *Evaluation of an Individualized Counseling Approach as Part of a Multicomponent School-Based Program to Prevent Weight-Related Problems among Adolescent Girls.* Journal of the American Dietetic Association, 2011. **111**(8): p. 1218-1223.
806. Foster, B.A., et al., *A pilot study of parent mentors for early childhood obesity.* Journal of obesity, 2016. **2016**.
807. Foster, B.A., et al., *A randomized clinical trial of the effects of parent mentors on early childhood obesity: Study design and baseline data.* Contemporary Clinical Trials. **45**(Pt B): p. 164-169.
808. Fotu, K., et al., *Outcome results for the Ma'alahi Youth Project, a Tongan community-based obesity prevention programme for adolescents.* obesity reviews, 2011. **12**: p. 41-50.
809. Fotu, K.F., et al., *Process evaluation of a community-based adolescent obesity prevention project in Tonga.* BMC Public Health, 2011. **11**(1): p. 284-284.
810. Francis, M., S.S. Nichols, and N. Dalrymple, *The effects of a school-based intervention programme on dietary intakes and physical activity among primary-school children in Trinidad and Tobago.* Public health nutrition, 2010. **13**(5): p. 738-747.
811. Nichols, S.D.S., M.P. Francis, and N. Dalrymple, *Sustainability of a curriculum-based intervention on dietary behaviours and physical activity among primary school children in Trinidad and Tobago.* West Indian Medical Journal, 2014. **63**(1): p. 68-77.
812. French, S.A., et al., *Household obesity prevention: take action—a group-randomized trial.* Obesity, 2011. **19**(10): p. 2082-2088.
813. French, S.A., et al., *Physical changes in the home environment to reduce television viewing and sugar-sweetened beverage consumption among 5-to 12-year-old children: a randomized pilot study.* Pediatric obesity, 2016. **11**(5): p. e12-e15.
814. Gallotta, M.C., et al., *Impacts of coordinative training on normal weight and overweight/obese children's attentional performance.* Frontiers in Human Neuroscience, 2015. **9**(OCTOBER).
815. Gallotta, M.C., et al., *Effects of combined physical education and nutritional programs on schoolchildren's healthy habits.* PeerJ, 2016. **2016**(4).
816. Garcia-Hermoso, A., et al., *Effects of a long-term physical exercise program with and without diet on obese boys after six-month detraining.* World Journal of Pediatrics. **10**(1): p. 38-45.
817. Garipagaoglu, M., et al., *Family-based group treatment versus individual treatment in the management of childhood obesity: randomized, prospective clinical trial.* European Journal of Pediatrics, 2009. **168**(9): p. 1091-9.
818. Gentile, D.A., et al., *Evaluation of a multiple ecological level child obesity prevention program: Switch® what you Do, View, and Chew.* BMC medicine, 2009. **7**(1): p. 49.
819. Eisenmann, J.C., et al., *SWITCH: Rationale, design, and implementation of a community, school, and family-based intervention to modify behaviors related to childhood obesity.* BMC Public Health, 2008. **8**.
820. Gillis, D., M. Brauner, and E. Granot, *A community-based behavior modification intervention for childhood obesity.* Journal of Pediatric Endocrinology and Metabolism, 2007. **20**(2): p. 197-204.
821. Gortmaker, S.L., et al., *Reducing obesity via a school-based interdisciplinary intervention among youth: Planet Health.* Archives of pediatrics & adolescent medicine, 1999. **153**(4): p. 409-418.
822. Austin, S.B., et al., *The impact of a school-based obesity prevention trial on disordered weight-control behaviors in early adolescent girls.* Archives of Pediatrics and Adolescent Medicine, 2005. **159**(3): p. 225-230.
823. Austin, S.B., et al., *School-based overweight preventive intervention lowers incidence of disordered weight-control behaviors in early adolescent girls.* Archives of Pediatrics & Adolescent Medicine, 2007. **161**(9): p. 865-869.
824. Chavarro, J.E., et al., *Effects of a school-based obesity-prevention intervention on menarche (United States).* Cancer Causes and Control, 2005. **16**(10): p. 1245-1252.

825. Gray, H.L., I.R. Contento, and P.A. Koch, *Linking implementation process to intervention outcomes in a middle school obesity prevention curriculum,'Choice, Control and Change'.* Health education research, 2015. **30**(2): p. 248-261.
826. Grydeland, M., et al., *Intervention effects on physical activity: the HEIA study-a cluster randomized controlled trial.* International Journal of Behavioral Nutrition and Physical Activity, 2013. **10**(1): p. 17.
827. Bergh, I.H., et al., *Mid-way and post-intervention effects on potential determinants of physical activity and sedentary behavior, results of the HEIA study - a multi-component school-based randomized trial.* Int J Behav Nutr Phys Act, 2012. **9**: p. 63.
828. Bergh, I.H., et al., *Personal and social-environmental correlates of objectively measured physical activity in Norwegian pre-adolescent children.* Scandinavian Journal of Medicine & Science in Sports, 2011. **21**(6): p. e315-24.
829. Bergh, I.H., et al., *Post-intervention effects on screen behaviours and mediating effect of parental regulation: the HEalth In Adolescents study--a multi-component school-based randomized controlled trial.* BMC Public Health, 2014. **14**: p. 200.
830. Bjelland, M., et al., *Changes in adolescents' intake of sugar-sweetened beverages and sedentary behaviour: results at 8 month mid-way assessment of the HEIA study--a comprehensive, multi-component school-based randomized trial.* Int J Behav Nutr Phys Act, 2011. **8**: p. 63.
831. Grydeland, M., et al., *Correlates of weight status among Norwegian 11-year-olds: The HEIA study.* BMC Public Health, 2012. **12**(1): p. 1053-1053.
832. Grydeland, M., et al., *Effects of a 20-month cluster randomised controlled school-based intervention trial on BMI of school-aged boys and girls: the HEIA study.* British Journal of Sports Medicine, 2014. **48**(9): p. 768-773.
833. Lien, N., et al., *Design of a 20-month comprehensive, multicomponent school-based randomised trial to promote healthy weight development among 11-13 year olds: The HEalth In Adolescents study.* Scandinavian Journal of Public Health, 2010. **38**(5 Suppl): p. 38-51.
834. Habib-Mourad, C., et al., *Health-E-PALS: promoting healthy eating and physical activity in Lebanese school children–intervention development.* Education and health., 2014. **32**(1): p. 3-8.
835. Habib-Mourad, C., et al., *An intervention to promote Healthy Eating and Physical Activity in Lebanese School children: Health-E-PALS, a pilot cluster randomised controlled trial.* Obesity Facts, 2014. **7**: p. 158-159.
836. Habib-Mourad, C., et al., *Promoting healthy eating and physical activity among school children: findings from Health-E-PALS, the first pilot intervention from Lebanon.* BMC Public Health. **14**: p. 940.
837. Harder-Lauridsen, N.M., et al., *A randomized controlled trial on a multicomponent intervention for overweight school-aged children–Copenhagen, Denmark.* BMC pediatrics, 2014. **14**(1): p. 273.
838. Hasson, R.E., et al., *Randomized controlled trial to improve adiposity, inflammation, and insulin resistance in obese African-American and Latino youth.* Obesity, 2012. **20**(4): p. 811-818.
839. Hendy, H.M., K.E. Williams, and T.S. Camise, *Kid's Choice Program improves weight management behaviors and weight status in school children.* Appetite, 2011. **56**(2): p. 484-494.
840. Hollar, D., et al., *Healthier options for public schoolchildren program improves weight and blood pressure in 6-to 13-year-olds.* Journal of the American Dietetic Association, 2010. **110**(2): p. 261-267.
841. Hollar, D., C. Heitz, and W. Zhou, *More young children in an obesity prevention intervention in ms and la head start centers improve/maintain BMI percentile and waist circumference compared to nonparticipants.* Circulation, 2015. **131**.

842. Hollar, D., et al., *Effective multi-level, multi-sector, school-based obesity prevention programming improves weight, blood pressure, and academic performance, especially among low-income, minority children.* Journal of Health Care for the Poor & Underserved, 2010. **21**(2): p. 93-108.
843. Hollar, D., et al., *Effect of a two-year obesity prevention intervention on percentile changes in body mass index and academic performance in low-income elementary school children.* American Journal of Public Health, 2010. **100**(4): p. 646-53.
844. Iaia, M., et al., *An educational intervention to promote healthy lifestyles in preschool children: a cluster-RCT.* International Journal of Obesity, 2017. **41**(4): p. 582-590.
845. Janicke, D.M., et al., *Comparison of parent-only vs family-based interventions for overweight children in underserved rural settings: outcomes from project STORY.* Archives of pediatrics & adolescent medicine, 2008. **162**(12): p. 1119-1125.
846. Armstrong, B., C.S. Lim, and D.M. Janicke, *Park Density Impacts Weight Change in a Behavioral Intervention for Overweight Rural Youth.* Behavioral Medicine. **41**(3): p. 123-30.
847. Janicke, D.M., et al., *Sensible Treatment of Obesity in Rural Youth (STORY): Design and methods.* Contemporary Clinical Trials, 2008. **29**(2): p. 270-280.
848. Jiang, J., et al., *The effects of a 3-year obesity intervention in schoolchildren in Beijing.* Child: care, health and development, 2007. **33**(5): p. 641-646.
849. Jiang, J., et al., *A two year family based behaviour treatment for obese children.* Archives of disease in childhood, 2005. **90**(12): p. 1235-1238.
850. Johnston, C.A., et al., *Effects of a school-based weight maintenance program for Mexican-American children: results at 2 years.* Obesity, 2010. **18**(3): p. 542-547.
851. Johnston, C.A., et al., *Achieving long-term weight maintenance in mexican-american adolescents with a school-based intervention.* Journal of Adolescent Health, 2013. **53**(3): p. 335-341.
852. Johnston, C.A., et al., *Results of an intensive school-based weight loss program with overweight Mexican American children.* International Journal of Pediatric Obesity, 2007. **2**(3): p. 144-152.
853. Johnston, C.A., et al., *Weight loss in overweight Mexican American children: A randomized, controlled trial.* Pediatrics, 2007. **120**(6): p. e1450-e1457.
854. Kain, J., et al., *School-based obesity prevention in Chilean primary school children: methodology and evaluation of a controlled study.* International journal of obesity, 2004. **28**(4): p. 483-493.
855. Kain, J., et al., *School-based obesity prevention intervention in Chilean children: effective in controlling, but not reducing obesity.* Journal of obesity, 2014. **2014**.
856. Kipping, R.R., et al., *Effect of intervention aimed at increasing physical activity, reducing sedentary behaviour, and increasing fruit and vegetable consumption in children: active for Life Year 5 (AFLY5) school based cluster randomised controlled trial.* Bmj, 2014. **348**: p. g3256.
857. Anderson, E.L., et al., *Long-term effects of the Active for Life Year 5 (AFLY5) school-based cluster-randomised controlled trial.* BMJ Open, 2016. **6**(11).
858. Jago, R., et al., *Lessons learned from the AFLY5 RCT process evaluation: implications for the design of physical activity and nutrition interventions in schools.* BMC Public Health, 2015. **15**(1): p. 1-10.
859. Lawlor, D.A., et al., *The Active for Life Year 5 (AFLY5) school based cluster randomised controlled trial: study protocol for a randomized controlled trial.* Trials [Electronic Resource], 2011. **12**: p. 181.
860. Lawlor, D.A., et al., *The Active for Life Year 5 (AFLY5) school-based cluster randomised controlled trial protocol: detailed statistical analysis plan.* Trials, 2013. **14**(1): p. 1-21.
861. Kipping, R.R., R. Jago, and D.A. Lawlor, *Diet outcomes of a pilot school-based randomised controlled obesity prevention study with 9-10year olds in England.* Preventive Medicine, 2010. **51**(1): p. 56-62.
862. Kipping, R.R., et al., *Randomised controlled trial adapting US school obesity prevention to England.* Archives of Disease in Childhood, 2008. **93**(6): p. 469-473.

863. Kobel, S., et al., *Intervention effects of a school-based health promotion programme on obesity related behavioural outcomes.* Journal of obesity, 2014. **2014**.
864. Dreyhaupt, J., et al., *Evaluation of a health promotion program in children: Study protocol and design of the cluster-randomized Baden-Württemberg primary school study [DRKS-ID: DRKS00000494].* BMC Public Health, 2012. **12**(1): p. 157-157.
865. Kesztyüs, D., et al., *Costs and effects of a state-wide health promotion program in primary schools in Germany - the Baden-Württemberg Study: a cluster-randomized, controlled trial.* PloS one, 2017. **12**(2): p. e0172332.
866. Kobel, S., et al., *Effects of a Randomised Controlled School-Based Health Promotion Intervention on Obesity Related Behavioural Outcomes of Children with Migration Background.* Journal of Immigrant & Minority Health, 2017. **19**(2): p. 254-262.
867. Kobel, S., et al., *Intervention effects of a kindergarten-based health promotion programme on obesity related behavioural outcomes and BMI percentiles.* Preventive medicine reports, 2019. **15**.
868. Kobel, S., et al., *Design, Implementation, and Study Protocol of a Kindergarten-Based Health Promotion Intervention.* BioMed Research International. **2017**: p. 4347675.
869. Kocken, P.L., et al., *Effects of a theory-based education program to prevent overweightness in primary school children.* Nutrients, 2016. **8**(1): p. 12.
870. Kong, A.S., et al., *School-based health center intervention improves body mass index in overweight and obese adolescents.* J Obes, 2013. **2013**: p. 575016.
871. LaRowe, T.L., et al., *Active Early: one-year policy intervention to increase physical activity among early care and education programs in Wisconsin.* BMC public health, 2016. **16**(1): p. 607.
872. Larsen, M.N., et al., *Positive effects on bone mineralisation and muscular fitness after 10 months of intense school-based physical training for children aged 8-10 years: the FIT FIRST randomised controlled trial.* Br J Sports Med, 2018. **52**(4): p. 254-260.
873. Leach, R.A. and J.M. Yates, *Nutrition and youth soccer for childhood overweight: a pilot novel chiropractic health education intervention.* Journal of manipulative and physiological therapeutics, 2008. **31**(6): p. 434-441.
874. Lee, A., et al., *Childhood obesity management shifting from health care system to school system: intervention study of school-based weight management programme.* BMC public health, 2014. **14**(1): p. 1128.
875. Lee, S., et al., *Effects of aerobic versus resistance exercise without caloric restriction on abdominal fat, intrahepatic lipid, and insulin sensitivity in obese adolescent boys: a randomized, controlled trial.* Diabetes, 2012. **61**(11): p. 2787-2795.
876. Deldin, A., J.L. Kuk, and S. Lee, *Influence of Sex on the Changes in Regional Fat and Skeletal Muscle Mass in Response to Exercise Training in Adolescents with Obesity.* Childhood Obesity, 2019. **15**(3): p. 216-222.
877. Horner, K., et al., *Effect of Aerobic versus Resistance Exercise on Pulse Wave Velocity, Intima Media Thickness and Left Ventricular Mass in Obese Adolescents.* Pediatric Exercise Science. **27**(4): p. 494-502.
878. Wilms, E., et al., *The Impact of Pectin Supplementation on Intestinal Barrier Function in Healthy Young Adults and Healthy Elderly.* Nutrients, 2019. **11**.
879. Lee, S., et al., *Aerobic exercise but not resistance exercise reduces visceral adiposity, liver fat and insulin resistance in obese adolescent girls: A randomized controlled trial.* Diabetes, 2013. **62**: p. A56.
880. Lee, S. and J.L. Kuk, *Changes in fat and skeletal muscle with exercise training in obese adolescents: comparison of whole-body MRI and dual energy X-ray absorptiometry.* Obesity. **21**(10): p. 2063-71.

881. Leme, A.C.B., et al., *Preventing obesity among Brazilian adolescent girls: Six-month outcomes of the Healthy Habits, Healthy Girls–Brazil school-based randomized controlled trial.* Preventive medicine, 2016. **86**: p. 77-83.
882. Leme, A.C. and S.T. Philippi, *The "Healthy Habits, Healthy Girls" randomized controlled trial for girls: study design, protocol, and baseline results.* Cadernos de Saude Publica. **31**(7): p. 1381-94.
883. Leme, A.C.B., et al., *Sustained impact of the "Healthy Habits, Healthy Girls – Brazil" school-based randomized controlled trial for adolescents living in low-income communities.* Preventive Medicine Reports, 2018. **10**: p. 346-352.
884. Leme, A.C.B., et al., *"Healthy Habits, Healthy Girls—Brazil": an obesity prevention program with added focus on eating disorders.* Eating and weight disorders, 2018: p. 1-13.
885. Li, X.-H., et al., *Effectiveness of a school-based physical activity intervention on obesity in school children: a nonrandomized controlled trial.* BMC public health, 2014. **14**(1): p. 1282.
886. Lioret, S., et al., *A parent focused child obesity prevention intervention improves some mother obesity risk behaviors: the Melbourne inFANT program.* International Journal of Behavioral Nutrition and Physical Activity, 2012. **9**(1): p. 100.
887. Cameron, A.J., et al., *Variation in outcomes of the Melbourne Infant, Feeding, Activity and Nutrition Trial (InFANT) Program according to maternal education and age.* Preventive Medicine. **58**: p. 58-63.
888. Campbell, K., et al., *The Infant Feeding Activity and Nutrition Trial (INFANT) an early intervention to prevent childhood obesity: cluster-randomised controlled trial.* BMC Public Health, 2008. **8**(1): p. 103-103.
889. Lioret, S., et al., *Parent focused intervention impacts obesity risk behaviours in infants: Results of the melbourne infant program cluster-randomised controlled trial.* Obesity Facts, 2012. **5**: p. 33.
890. Spence, A.C., et al., *Mediators of improved child diet quality following a health promotion intervention: the Melbourne InFANT Program.* International Journal of Behavioral Nutrition & Physical Activity. **11**: p. 137.
891. Walsh, A.D., et al., *The effect of an early childhood obesity intervention on father's obesity risk behaviors: The Melbourne InFANT Program.* International Journal of Behavioral Nutrition and Physical Activity, 2014. **11**(1).
892. Spence, A.C., et al., *A health promotion intervention can affect diet quality in early childhood.* Journal of Nutrition. **143**(10): p. 1672-8.
893. Lisón, J.F., et al., *Exercise intervention in childhood obesity: a randomized controlled trial comparing hospital-versus home-based groups.* Academic pediatrics, 2012. **12**(4): p. 319-325.
894. Llargues, E., et al., *Assessment of a school-based intervention in eating habits and physical activity in school children: the AVall study.* J Epidemiol Community Health, 2011. **65**(10): p. 896-901.
895. Llargues, E., et al., *Medium-term evaluation of an educational intervention on dietary and physical exercise habits in schoolchildren: the Avall 2 study.* Endocrinologia y Nutricion. **59**(5): p. 288-95.
896. Llargues, E., et al., *Four-year outcomes of an educational intervention in healthy habits in schoolchildren: the Avall 3 Trial.* European Journal of Public Health. **27**(1): p. 42-47.
897. Lopes, W.A., et al., *Effects of 12 weeks of combined training without caloric restriction on inflammatory markers in overweight girls.* Journal of sports sciences, 2016. **34**(20): p. 1902-1912.
898. Lubans, D.R., P.J. Morgan, and R. Callister, *Potential moderators and mediators of intervention effects in an obesity prevention program for adolescent boys from disadvantaged schools.* Journal of science and medicine in sport, 2012. **15**(6): p. 519-525.
899. Lubans, D., et al., *Randomised controlled trial of the Physical Activity Leaders (PALs) program for low-active adolescent boys from disadvantaged secondary schools.* Journal of Science and Medicine in Sport, 2010. **13**: p. e12.

900. Lubans, D.R., et al., *Randomized controlled trial of the Physical Activity Leaders (PALs) program for adolescent boys from disadvantaged secondary schools.* Preventive Medicine, 2011. **52**(3-4): p. 239-246.
901. Lynch, B.A., et al., *Elementary School–Based Obesity Intervention Using an Educational Curriculum.* Journal of primary care & community health, 2016. **7**(4): p. 265-271.
902. Malakellis, M., et al., *School-based systems change for obesity prevention in adolescents: outcomes of the Australian Capital Territory 'It's Your Move!'.* Australian and New Zealand journal of public health, 2017. **41**(5): p. 490-496.
903. Manger, W.M., et al., *Obesity prevention in young schoolchildren: Results of a pilot study.* Journal of School Health, 2012. **82**(10): p. 462-468.
904. Marcus, C., et al., *A 4-year, cluster-randomized, controlled childhood obesity prevention study: STOPP.* International Journal of Obesity, 2009. **33**(4): p. 408-417.
905. Moir, C., et al., *Early intervention to encourage physical activity in infants and toddlers: a randomized controlled trial.* Medicine & Science in Sports & Exercise, 2016. **48**(12): p. 2446-2453.
906. Cameron, S.L., et al., *Excessive growth from 6 to 24 months of age: Results from the prevention of overweight in infancy (POI) randomised controlled trial.* Archives of Disease in Childhood, 2014. **99**: p. A109.
907. Fangupo, L.J., et al., *Impact of an early-life intervention on the nutrition behaviors of 2-y-old children: a randomized controlled trial.* American Journal of Clinical Nutrition, 2015. **102**(3): p. 704-712.
908. Taylor, B., et al., *The prevention of obesity in infancy by targeting sleep or food and activity: RCT outcomes at 5 years.* Obesity facts, 2017. **10**: p. 23-.
909. Taylor, B.J., et al., *Prevention of Overweight in Infancy (POI.nz) study: a randomised controlled trial of sleep, food and activity interventions for preventing overweight from birth.* BMC Public Health. **11**: p. 942.
910. Taylor, R., *Providing additional guidance and support to parents about sleep, diet and physical activity from birth to 2 years of age: The Prevention of Overweight in Infancy study.* Obesity Research and Clinical Practice, 2014. **8**: p. 102-103.
911. Taylor, R.W., et al., *Sleep, nutrition, and physical activity interventions to prevent obesity in infancy: Follow-up of the Prevention of Overweight in Infancy (POI) randomized controlled trial at ages 3.5 and 5 y.* American Journal of Clinical Nutrition, 2018. **108**(2): p. 228-236.
912. Taylor, R.W., et al., *Three-year follow-up of a randomised controlled trial to reduce excessive weight gain in the first two years of life: protocol for the POI follow-up study.* BMC public health, 2016. **16**(1): p. 771.
913. Mo-suwan, L., et al., *Effects of a controlled trial of a school-based exercise program on the obesity indexes of preschool children.* The American journal of clinical nutrition, 1998. **68**(5): p. 1006-1011.
914. Murphy, E.C., et al., *Effects of an exercise intervention using Dance Dance Revolution on endothelial function and other risk factors in overweight children.* International Journal of Pediatric Obesity, 2009. **4**(4): p. 205-214.
915. Maloney, A.E., et al., *A pilot of a video game (DDR) to promote physical activity and decrease sedentary screen time.* Obesity (Silver Spring), 2008. **16**(9): p. 2074-80.
916. Natale, R.A., et al., *Role modeling as an early childhood obesity prevention strategy: effect of parents and teachers on preschool children's healthy lifestyle habits.* Journal of Developmental & Behavioral Pediatrics, 2014. **35**(6): p. 378-387.
917. Natale, R.A., et al., *Effect of a child care center-based obesity prevention program on body mass index and nutrition practices among preschool-aged children.* Health promotion practice, 2014. **15**(5): p. 695-705.

918. Messiah, S.E., et al., *Healthy caregivers-healthy children (HC2) phase 2: Integrating culturally sensitive childhood obesity prevention strategies into childcare center policies.* Contemporary Clinical Trials. **53**: p. 60-67.
919. Natale, R., et al., *Healthy Caregivers-Healthy Children (HC2): A Childcare Center Based Obesity Prevention.* Journal of Nutrition Education & Behavior, 2013. **45**(4 Supplement): p. S86-7.
920. Natale, R., et al., *Design and methods for evaluating an early childhood obesity prevention program in the childcare center setting.* BMC Public Health, 2013. **13**(1): p. 78-78.
921. Natale, R.N., et al., *Healthy caregivers-healthy children: A primary prevention program with preschool children.* Cardiology (Switzerland), 2016. **134**: p. 71.
922. McCollister, K.E., et al., *Cost analysis of a childcare center-based intervention to prevent obesity in the preschool years.* Journal of comprehensive pediatrics, 2015. **6**(2).
923. Natale, R.A., et al., *Obesity Prevention Program in Childcare Centers: two-Year Follow-Up.* American journal of health promotion, 2017. **31**(6): p. 502-510.
924. Nemet, D., et al., *Short-and long-term beneficial effects of a combined dietary–behavioral–physical activity intervention for the treatment of childhood obesity.* Pediatrics, 2005. **115**(4): p. e443-e449.
925. Nemet, D., et al., *Effects of a multidisciplinary childhood obesity treatment intervention on adipocytokines, inflammatory and growth mediators.* Hormone research in paediatrics. **79**(6): p. 325-32.
926. Nerud, K. and H. Samra, *Make a move intervention to reduce childhood obesity.* The Journal of School Nursing, 2017. **33**(3): p. 205-213.
927. Nguyen, B., et al., *Twelve-month outcomes of the loozit randomized controlled trial: a community-based healthy lifestyle program for overweight and obese adolescents.* Archives of pediatrics & adolescent medicine, 2012. **166**(2): p. 170-177.
928. *Kornman KP, Shrewsbury VA, Chou AC, Nguyen B, Lee A, O'Connor J, Steinbeck KS, Hill AJ, Kohn MR, Shah S, Baur LA. Electronic therapeutic contact for adolescent weight management: The Loozit® study. Telemedicine and e-Health. 2010 Jul 1;16(6):678-85.*
929. Nguyen, B., et al., *A process evaluation of an adolescent weight management intervention: findings and recommendations.* Health Promotion International. **30**(2): p. 201-12.
930. Nguyen, B., et al., *Two-year outcomes of an adjunctive telephone coaching and electronic contact intervention for adolescent weight-loss maintenance: the Loozit randomized controlled trial.* International Journal of Obesity. **37**(3): p. 468-72.
931. Shrewsbury, V.A., et al., *Short-term outcomes of community-based adolescent weight management: The Loozit® Study.* BMC Pediatrics, 2011. **11**(1): p. 13-13.
932. Shrewsbury, V.A., et al., *A randomised controlled trial of a community-based healthy lifestyle program for overweight and obese adolescents: the Loozit study protocol.* BMC Public Health, 2009. **9**: p. 119.
933. Nollen, N.L., et al., *Mobile technology for obesity prevention: a randomized pilot study in racial-and ethnic-minority girls.* American journal of preventive medicine, 2014. **46**(4): p. 404-408.
934. Novotny, R., et al., *Pacific kids DASH for health (PacDASH) randomized, controlled trial with DASH eating plan plus physical activity improves fruit and vegetable intake and diastolic blood pressure in children.* Childhood Obesity, 2015. **11**(2): p. 177-186.
935. Nourian, M., R. Kelishadi, and A. Najimi, *Lifestyle interventions and weight control of adolescents with abdominal obesity: a randomized controlled trial based on health belief model.* Iranian red crescent medical journal, 2017. **19**(2).
936. Nyström, C.D., et al., *Mobile-based intervention intended to stop obesity in preschool-aged children: the MINISTOP randomized controlled trial.* The American journal of clinical nutrition, 2017. **105**(6): p. 1327-1335.

937. Cadenas-Sanchez, C., et al., *Parental body mass index and its association with body composition, physical fitness and lifestyle factors in their 4-year-old children: results from the MINISTOP trial.* European journal of clinical nutrition, 2017. **71**(10): p. 1200-1205.
938. Delisle, C., et al., *A web- and mobile phone-based intervention to prevent obesity in 4-year-olds (MINISTOP): a population-based randomized controlled trial.* BMC public health, 2015. **15**: p. 95.
939. Delisle Nyström, C., et al., *A 12-month follow-up of a mobile-based (mHealth) obesity prevention intervention in pre-school children: the MINISTOP randomized controlled trial.* BMC Public Health, 2018. **18**(1): p. N.PAG-N.PAG.
940. Henriksson, P., et al., *Associations of Fat Mass and Fat-Free Mass with Physical Fitness in 4-Year-Old Children: Results from the MINISTOP Trial.* Nutrients, 2016. **8**(8): p. 30.
941. Parekh, N., et al., *Associations of Parental Self-Efficacy With Diet, Physical Activity, Body Composition, and Cardiorespiratory Fitness in Swedish Preschoolers: Results From the MINISTOP Trial.* Health Education & Behavior. **45**(2): p. 238-246.
942. Obert, P., et al., *Impact of Diet and Exercise Training-Induced Weight Loss on Myocardial Mechanics in Severely Obese Adolescents.* Obesity, 2013. **21**(10): p. 2091-2098.
943. O'Connor, T., et al., *Feasibility of an obesity intervention for paediatric primary care targeting parenting and children: Helping HAND.* Child: care, health and development, 2013. **39**(1): p. 141-149.
944. Parra-Medina, D., et al., *Promoting weight maintenance among overweight and obese Hispanic children in a rural practice.* Childhood Obesity, 2015. **11**(4): p. 355-363.
945. Poeta, L.S., et al., *Interdisciplinary intervention in obese children and impact on health and quality of life.* Jornal de pediatria, 2013. **89**(5): p. 499-504.
946. Pope, M., *Preventing Weight Gain in Children Who Are School Age and African-American.* Pediatric Physical Therapy, 2016. **28**(2): p. 207-216.
947. Puder, J.J., et al., *Effect of multidimensional lifestyle intervention on fitness and adiposity in predominantly migrant preschool children (Ballabeina): cluster randomised controlled trial.* Bmj, 2011. **343**: p. d6195.
948. Bürgi, F., et al., *Effect of a lifestyle intervention on adiposity and fitness in socially disadvantaged subgroups of preschoolers: A cluster-randomized trial (Ballabeina).* Preventive Medicine, 2012. **54**(a5): p. 335-340.
949. Niederer, I., et al., *Effects of a lifestyle intervention on adiposity and fitness in overweight or low fit preschoolers (Ballabeina).* Obesity, 2013. **21**(3): p. E287-E293.
950. Niederer, I., et al., *Influence of a lifestyle intervention in preschool children on physiological and psychological parameters (Ballabeina): Study design of a cluster randomized controlled trial.* BMC Public Health, 2009. **9**.
951. Quattrin, T., et al., *Treatment outcomes of overweight children and parents in the medical home.* Pediatrics, 2014. **134**(2): p. 290-297.
952. Quattrin, T., et al., *Cost-effectiveness of family-based obesity treatment.* Pediatrics, 2017. **140**(3).
953. Quattrin, T., et al., *Treatment Outcomes of Overweight Children and Parents in the Medical Home.* Pediatrics, 2014. **134**(2): p. 290-297.
954. Rifas-Shiman, S.L., et al., *Two-year follow-up of a primary care-based intervention to prevent and manage childhood obesity: the High Five for Kids study.* Pediatric obesity, 2017. **12**(3): p. e24-e27.
955. Haire-Joshu, D., et al., *High 5 for Kids: the impact of a home visiting program on fruit and vegetable intake of parents and their preschool children.* Preventive medicine, 2008. **47**(1): p. 77-82.
956. Cespedes, E.M., et al., *Participant characteristics and intervention processes associated with reductions in television viewing in the High Five for Kids study.* Preventive Medicine, 2014. **62**: p. 64-70.

957. Taveras, E.M., et al., *Randomized controlled trial to improve primary care to prevent and manage childhood obesity the high five for kids study.* Archives of Pediatrics and Adolescent Medicine, 2011. **165**(8): p. 714-722.
958. Taveras, E.M., et al., *Correlates of participation in a pediatric primary care-based obesity prevention intervention.* Obesity, 2011. **19**(2): p. 449-452.
959. Rodearmel, S.J., et al., *A family-based approach to preventing excessive weight gain.* Obesity, 2006. **14**(8): p. 1392-1401.
960. Rodearmel, S.J., et al., *Small changes in dietary sugar and physical activity as an approach to preventing excessive weight gain: the America on the Move family study.* Pediatrics, 2007. **120**(4): p. e869-e879.
961. Rooney, B.L., et al., *Growing healthy families: family use of pedometers to increase physical activity and slow the rate of obesity.* WMJ-MADISON-, 2005. **104**(5): p. 54.
962. Rosenkranz, R.R., T.K. Behrens, and D.A. Dzewaltowski, *A group-randomized controlled trial for health promotion in Girl Scouts: healthier troops in a SNAP (Scouting Nutrition & Activity Program).* BMC public health, 2010. **10**(1): p. 81.
963. Guagliano, J.M. and R.R. Rosenkranz, *Physical activity promotion and obesity prevention in Girl Scouts: Scouting Nutrition and Activity Program+.* Pediatrics International, 2012. **54**(6): p. 810-815.
964. Rush, E., et al., *A school-based obesity control programme: Project Energize. Two-year outcomes.* British Journal of Nutrition, 2012. **107**(4): p. 581-587.
965. Graham, D., et al., *Increasing activity and improving nutrition through a schools-based programme: Project Energize. 1. Design, programme, randomisation and evaluation methodology.* Public Health Nutrition. **11**(10): p. 1076-84.
966. Rush, E. and S. Mc Lennan, *Project energize: Reducing childhood obesity; translation from a randomised controlled trial to an effective regional health service.* Annals of Nutrition and Metabolism, 2013. **63**: p. 308.
967. Rush, E., et al., *Project Energize: whole-region primary school nutrition and physical activity programme; evaluation of body size and fitness 5 years after the randomised controlled trial.* British Journal of Nutrition, 2014. **111**(2): p. 363-371.
968. Herbert, P.C., et al., *Effectiveness of the Energize Elementary School Program to Improve Diet and Exercise.* Journal of School Health, 2013. **83**(11): p. 780-786.
969. Saelens, B.E., et al., *Behavioral weight control for overweight adolescents initiated in primary care.* Obesity research, 2002. **10**(1): p. 22-32.
970. Saelens, B.E., et al., *Efficacy of increasing physical activity to reduce children's visceral fat: A pilot randomized controlled trial.* International Journal of Pediatric Obesity, 2011. **6**(2): p. 102-112.
971. Saelens, B.E., P. Lozano, and K. Scholz, *A randomized clinical trial comparing delivery of behavioral pediatric obesity treatment using standard and enhanced motivational approaches.* Journal of pediatric psychology, 2013. **38**(9): p. 954-964.
972. Safdie, M., et al., *Impact of a school-based intervention program on obesity risk factors in Mexican children.* salud pública de méxico, 2013. **55**: p. 374-387.
973. Safdie, M., et al., *Promoting healthful diet and physical activity in the Mexican school system for the prevention of obesity in children.* Salud Publica de Mexico. **55 Suppl 3**: p. 357-73.
974. Sahota, P., et al., *Evaluation of implementation and effect of primary school based intervention to reduce risk factors for obesity.* Bmj, 2001. **323**(7320): p. 1027.
975. Sahota, P., et al., *Randomised controlled trial of primary school based intervention to reduce risk factors for obesity.* BMJ. **323**(7320): p. 1029-32.
976. Salazar, G., et al., *Pilot nutrition and physical activity intervention for preschool children attending daycare centres (JUNJI); primary and secondary outcomes.* Nutricion hospitalaria, 2014. **29**(5): p. 1004-1012.

977. Sanigorski, A.M., et al., *Reducing unhealthy weight gain in children through community capacity-building: results of a quasi-experimental intervention program, Be Active Eat Well.* International journal of obesity, 2008. **32**(7): p. 1060-1067.
978. Johnson, B.A., et al., *Multilevel analysis of the Be Active Eat Well intervention: environmental and behavioural influences on reductions in child obesity risk.* International Journal of Obesity. **36**(7): p. 901-7.
979. Moodie, M.L., et al., *The cost-effectiveness of a successful community-based obesity prevention program: The be active eat well program.* Obesity (19307381), 2013. **21**(10): p. 2072-2080.
980. Santos, R.G., et al., *Effectiveness of peer-based healthy living lesson plans on anthropometric measures and physical activity in elementary school students: a cluster randomized trial.* JAMA pediatrics, 2014. **168**(4): p. 330-337.
981. Schwingshandl, J., et al., *Effect of an individualised training programme during weight reduction on body composition: a randomised trial.* Archives of disease in childhood, 1999. **81**(5): p. 426-428.
982. Serra-Paya, N., et al., *Effectiveness of a multi-component intervention for overweight and obese children (Nereu program): a randomized controlled trial.* PLoS One, 2015. **10**(12).
983. Serra-Paya, N., et al., *Evaluation of a family intervention programme for the treatment of overweight and obese children (Nereu Programme): a randomized clinical trial study protocol.* BMC Public Health. **13**: p. 1000.
984. Shalitin, S., et al., *Effects of a twelve-week randomized intervention of exercise and/or diet on weight loss and weight maintenance, and other metabolic parameters in obese preadolescent children.* Hormone Research, 2009. **72**(5): p. 287-301.
985. Levy, T.S., et al., *Effectiveness of a diet and physical activity promotion strategy on the prevention of obesity in Mexican school children.* BMC public health, 2012. **12**(1): p. 152.
986. Morales-Ruan Mdel, C., et al., *Effects of an intervention strategy for school children aimed at reducing overweight and obesity within the State of Mexico.* Salud Publica de Mexico. **56 Suppl 2**: p. s113-22.
987. Sherwood, N.E., et al., *Healthy Homes/Healthy Kids: a randomized trial of a pediatric primary care-based obesity prevention intervention for at-risk 5–10 year olds.* Contemporary clinical trials, 2013. **36**(1): p. 228-243.
988. Sherwood, N.E., et al., *Pediatric primary care–based obesity prevention for parents of preschool children: A pilot study.* Childhood Obesity, 2015. **11**(6): p. 674-682.
989. Sherwood, N.E., et al., *The Healthy Homes/Healthy Kids 5-10 Obesity Prevention Trial: 12 and 24-month outcomes.* Pediatric Obesity, 2019. **14**(8).
990. Shofan, Y., et al., *A school-based program of physical activity may prevent obesity.* European journal of clinical nutrition, 2011. **65**(6): p. 768-770.
991. Singhal, N., et al., *Effects of controlled school-based multi-component model of nutrition and lifestyle interventions on behavior modification, anthropometry and metabolic risk profile of urban Asian Indian adolescents in North India.* European journal of clinical nutrition, 2010. **64**(4): p. 364-373.
992. Skouteris, H., et al., *A parent-based intervention to promote healthy eating and active behaviours in pre-school children: evaluation of the MEND 2–4 randomized controlled trial.* Pediatric obesity, 2016. **11**(1): p. 4-10.
993. Slawson, D.L., et al., *College students as facilitators in reducing adolescent obesity disparity in Southern Appalachia: Team Up for Healthy Living.* Contemporary clinical trials, 2015. **43**: p. 39-52.
994. Small, L., et al., *The preliminary effects of a primary care–based randomized treatment trial with overweight and obese young children and their parents.* Journal of Pediatric Health Care, 2014. **28**(3): p. 198-207.

995. Sosa, E.T., et al., *¡ Miranos!(Look at Us! We Are Healthy!) Home-Based and Parent Peer–Led Childhood Obesity Prevention.* Health promotion practice, 2016. **17**(5): p. 675-681.
996. Sosa, E.T., et al., *¡Miranos! (Look at Us! We Are Healthy!).* Health Promotion Practice, 2016. **17**(5): p. 675-681.
997. Yin, Z., et al., *Study protocol for a cluster randomized controlled trial to test "¡Míranos! Look at Us, We Are Healthy!" - An early childhood obesity prevention program.* BMC Pediatrics, 2019. **19**(1).
998. Stettler, N., et al., *Prevention of excess weight gain in paediatric primary care: beverages only or multiple lifestyle factors. The S mart S tep S tudy, a cluster-randomized clinical trial.* Pediatric obesity, 2015. **10**(4): p. 267-274.
999. Story, M., et al., *Bright Start: description and main outcomes from a group-randomized obesity prevention trial in American Indian children.* Obesity, 2012. **20**(11): p. 2241-2249.
1000. Suarez-Balcazar, Y., et al., *A university–school collaboration to enhance healthy choices among children.* Journal of prevention & intervention in the community, 2014. **42**(2): p. 140-151.
1001. Tarro, L., et al., *Follow-up of a Healthy Lifestyle Education Program (the Educació en Alimentació Study): 2 Years After Cessation of Intervention.* Journal of Adolescent Health, 2014. **55**(6): p. 782-789.
1002. Giralt, M., et al., *A primary-school-based study to reduce prevalence of childhood obesity in Catalunya (Spain)--EDAL-Educacio en alimentacio: study protocol for a randomised controlled trial.* Trials [Electronic Resource], 2011. **12**: p. 54.
1003. Llaurado, E., et al., *EdAl-2 (Educacio en Alimentacio) programme: reproducibility of a cluster randomised, interventional, primary-school-based study to induce healthier lifestyle activities in children.* BMJ Open. **4**(11): p. e005496.
1004. Tarro, L., et al., *A primary-school-based study to reduce the prevalence of childhood obesity--the EdAl (Educacio en Alimentacio) study: a randomized controlled trial.* Trials [Electronic Resource]. **15**: p. 58.
1005. Taylor, R.W., et al., *Reducing weight gain in children through enhancing physical activity and nutrition: the APPLE project.* International Journal of Pediatric Obesity, 2006. **1**(3): p. 146-152.
1006. Fung, C., et al., *From "best practice" to "next practice": the effectiveness of school-based health promotion in improving healthy eating and physical activity and preventing childhood obesity.* International Journal of Behavioral Nutrition & Physical Activity. **9**: p. 27.
1007. McAuley, K.A., et al., *Economic evaluation of a community-based obesity prevention program in children: the APPLE project.* Obesity, 2010. **18**(1): p. 131-6.
1008. Taylor, R.W., et al., *Two-year follow-up of an obesity prevention initiative in children: the APPLE project.* American journal of clinical nutrition, 2008. **88**(5): p. 1371-1377.
1009. Taylor, R.W., et al., *APPLE Project: 2-y findings of a community-based obesity prevention program in primary school age children.* American Journal of Clinical Nutrition, 2007. **86**(3): p. 735-42.
1010. Taylor, R.W., et al., *Reducing weight gain in children through enhancing physical activity and nutrition: the APPLE project.* International Journal of Pediatric Obesity. **1**(3): p. 146-52.
1011. Taylor, R.W., et al., *Effect of a baby-led approach to complementary feeding on infant growth and overweight: a randomized clinical trial.* JAMA pediatrics, 2017. **171**(9): p. 838-846.
1012. Toulabi, T., et al., *The influence of a behavior modification interventional program on body mass index in obese adolescents.* Journal of the Formosan Medical Association, 2012. **111**(3): p. 153-159.
1013. Tucker, S., et al., *A School Based Community Partnership for Promoting Healthy Habits for Life.* Journal of Community Health, 2011. **36**(3): p. 414-422.
1014. Tucker, S.J., et al., *Reducing Pediatric Overweight: Nurse-Delivered Motivational Interviewing in Primary Care.* Journal of Pediatric Nursing, 2013. **28**(6): p. 536-547.
1015. van Grieken, A., et al., *Promotion of a healthy lifestyle among 5-year-old overweight children: health behavior outcomes of the'Be active, eat right'study.* BMC public health, 2014. **14**(1): p. 59.

1016. Veldhuis, L., et al., *'Be active, eat right', evaluation of an overweight prevention protocol among 5-year-old children: design of a cluster randomised controlled trial.* BMC Public Health, 2009. **9**(1): p. 177-177.
1017. van Nassau, F., et al., *The Dutch Obesity Intervention in Teenagers (DOiT) cluster controlled implementation trial: intervention effects and mediators and moderators of adiposity and energy balance-related behaviours.* International Journal of Behavioral Nutrition and Physical Activity, 2014. **11**(1): p. 158.
1018. Chin, A.P.M.J.M., et al., *Why did soft drink consumption decrease but screen time not? Mediating mechanisms in a school-based obesity prevention program.* International Journal of Behavioral Nutrition and Physical Activity, 2008. **5**.
1019. Vanhelst, J., et al., *Effects of a multidisciplinary rehabilitation program on pediatric obesity: the CEMHaVi program.* International Journal of Rehabilitation Research, 2011. **34**(2): p. 110-114.
1020. Verbestel, V., et al., *Prevention of overweight in children younger than 2 years old: a pilot cluster-randomized controlled trial.* Public Health Nutrition, 2014. **17**(6): p. 1384-1392.
1021. Veugelers, P.J. and A.L. Fitzgerald, *Effectiveness of school programs in preventing childhood obesity: a multilevel comparison.* American journal of public health, 2005. **95**(3): p. 432-435.
1022. Vilchis-Gil, J., et al., *Decreased body mass index in schoolchildren after yearlong information sessions with parents reinforced with web and mobile phone resources: community trial.* Journal of medical Internet research, 2016. **18**(6): p. e174.
1023. Vilchis-Gil, J., S. Flores-Huerta, and M. Klünder-Klünder, *Effect on the Metabolic Biomarkers in Schoolchildren After a Comprehensive Intervention Using Electronic Media and In-Person Sessions to Change Lifestyles: Community Trial.* Journal of Medical Internet Research, 2018. **20**(2): p. 10-10.
1024. Wake, M., et al., *Outcomes and costs of primary care surveillance and intervention for overweight or obese children: the LEAP 2 randomised controlled trial.* Bmj, 2009. **339**: p. b3308.
1025. Wake, M., et al., *Economic evaluation of a primary care trial to reduce weight gain in overweight/obese children: the LEAP trial.* Ambulatory Pediatrics, 2008. **8**(5): p. 336-41.
1026. Waling, M., et al., *A one-year intervention has modest effects on energy and macronutrient intakes of overweight and obese Swedish children.* The Journal of nutrition, 2010. **140**(10): p. 1793-1798.
1027. Wang, J.-j., et al., *Evaluation of a comprehensive intervention with a behavioural modification strategy for childhood obesity prevention: a nonrandomized cluster controlled trial.* BMC Public Health, 2015. **15**(1): p. 1206.
1028. Waters, E., et al., *Cluster randomised trial of a school-community child health promotion and obesity prevention intervention: findings from the evaluation of fun 'n healthy in Moreland!* BMC public health, 2018. **18**(1): p. 92.
1029. White, M.A., et al., *Mediators of weight loss in a family-based intervention presented over the internet.* Obesity research, 2004. **12**(7): p. 1050-1059.
1030. Williamson, D.A., et al., *Efficacy of an internet-based behavioral weight loss program for overweight adolescent African-American girls.* Eating & Weight Disorders: EWD. **10**(3): p. 193-203.
1031. Williamson, D.A., et al., *Two-year internet-based randomized controlled trial for weight loss in African-American girls.* Obesity, 2006. **14**(7): p. 1231-43.
1032. Wright, K. and Z. Suro, *Using Community–Academic Partnerships and a Comprehensive School-Based Program to Decrease Health Disparities in Activity in School-Aged Children.* Journal of Prevention & Intervention in the Community, 2014. **42**(2): p. 125-139.
1033. Xu, F., et al., *A school-based comprehensive lifestyle intervention among Chinese kids against Obesity (CLICK-Obesity) in Nanjing City, China: the baseline data.* Asia Pacific journal of clinical nutrition, 2014. **23**(1): p. 48.
1034. Xu, F., et al., *The effectiveness of a cluster-randomized controlled childhood obesity prevention study among Chinese primary school students: CLICK-obesity.* Obesity Reviews, 2014. **15**: p. 236.

1035. Xu, F., et al., *Effectiveness of a randomized controlled lifestyle intervention to prevent obesity among Chinese primary school students: Click-obesity study.* PLoS ONE, 2015. **10**(10).
1036. Xu, F., et al., *A school-based comprehensive lifestyle intervention among chinese kids against obesity (CLICK-Obesity): rationale, design and methodology of a randomized controlled trial in Nanjing city, China.* BMC Public Health, 2012. **12**(1): p. 316-316.
1037. Yang, Y., et al., *Effect of an obesity prevention program focused on motivating environments in childhood: a school-based prospective study.* International Journal of Obesity, 2017. **41**(7): p. 1027-1034.
1038. Zask, A., et al., *Tooty Fruity Vegie: an obesity prevention intervention evaluation in Australian preschools.* Health Promotion Journal of Australia, 2012. **23**(1): p. 10-15.
1039. Adams, J., A. Zask, and U. Dietrich, *Tooty Fruity Vegie in Preschools: an obesity prevention intervention in preschools targeting children's movement skills and eating behaviours.* Health Promotion Journal of Australia. **20**(2): p. 112-9.
1040. Zask, A., et al., *Three year follow-up of an early childhood intervention: is movement skill sustained?* International Journal of Behavioral Nutrition & Physical Activity. **9**: p. 127.
1041. Akdemir, M., L. Donmez, and H. Polat, *The effect of nutritional and physical activity interventions on nutritional status and obesity in primary school children: A cluster randomized controlled study.* Kuwait Medical Journal, 2017. **49**(2): p. 105-113.
1042. Alaimo, K., et al., *Effects of changes in lunch-time competitive foods, nutrition practices, and nutrition policies on low-income middle-school children's diets.* Childhood obesity, 2013. **9**(6): p. 509-523.
1043. Albala, C., et al., *Effects of replacing the habitual consumption of sugar-sweetened beverages with milk in Chilean children.* American Journal of Clinical Nutrition, 2008. **88**(3): p. 605-611.
1044. Andersen, L.B.B., et al., *The effects of water and dairy drinks on dietary patterns in overweight adolescents.* International Journal of Food Sciences & Nutrition, 2016. **67**(3): p. 314-324.
1045. Ask, A.S., et al., *Serving of free school lunch to secondary-school pupils–a pilot study with health implications.* Public health nutrition, 2010. **13**(2): p. 238-244.
1046. Bean, M.K., et al., *Dietary intake in a randomized-controlled pilot of NOURISH: A parent intervention for overweight children.* Preventive medicine, 2012. **55**(3): p. 224-227.
1047. Bean, M.K., et al., *Dietary intake in a randomized-controlled pilot of NOURISH: a parent intervention for overweight children.* Preventive Medicine. **55**(3): p. 224-7.
1048. Mazzeo, S.E., et al., *Parent skills training to enhance weight loss in overweight children: Evaluation of NOURISH.* Eating Behaviors, 2014. **15**(2): p. 225-229.
1049. Mazzeo, S.E., et al., *Nourishing Our Understanding of Role Modeling to Improve Support and Health (NOURISH): Design and methods.* Contemporary Clinical Trials, 2012. **33**(3): p. 515-522.
1050. Beets, M.W., et al., *Intervention leads to improvements in the nutrient profile of snacks served in afterschool programs: a group randomized controlled trial.* Translational behavioral medicine, 2016. **6**(3): p. 329-338.
1051. Beets, M.W., et al., *Economic evaluation of a group randomized controlled trial on healthy eating and physical activity in afterschool programs.* Preventive Medicine. **106**: p. 60-65.
1052. Beets, M.W., et al., *Making healthy eating and physical activity policy practice: the design and overview of a group randomized controlled trial in afterschool programs.* Contemporary Clinical Trials. **38**(2): p. 291-303.
1053. Beets, M.W., et al., *Physical activity outcomes in afterschool programs: A group randomized controlled trial.* Preventive Medicine. **90**: p. 207-15.
1054. Berkowitz, R.I., et al., *Meal replacements in the treatment of adolescent obesity: A randomized controlled trial.* Obesity, 2011. **19**(6): p. 1193-1199.

1055. Blum, J.E.W., et al., *Reduced availability of sugar-sweetened beverages and diet soda has a limited impact on beverage consumption patterns in Maine high school youth.* Journal of nutrition education and behavior, 2008. **40**(6): p. 341-347.
1056. Casazza, K., et al., *Reduced carbohydrate diet to improve metabolic outcomes and decrease adiposity in obese peripubertal African American girls.* Journal of Pediatric Gastroenterology and Nutrition, 2012. **54**(3): p. 336-342.
1057. Coleman, K.J., et al., *The healthy options for nutrition environments in schools (Healthy ONES) group randomized trial: using implementation models to change nutrition policy and environments in low income schools.* International Journal of Behavioral Nutrition and Physical Activity, 2012. **9**(1): p. 80.
1058. De Bock, F., et al., *Positive impact of a pre-school-based nutritional intervention on children's fruit and vegetable intake: results of a cluster-randomized trial.* Public Health Nutrition, 2012. **15**(3): p. 466-475.
1059. De Bock, F., et al., *A participatory parent-focused intervention promoting physical activity in preschools: design of a cluster-randomized trial.* BMC Public Health, 2010. **10**: p. 49.
1060. de Ruyter, J.C., et al., *A trial of sugar-free or sugar-sweetened beverages and body weight in children.* New England Journal of Medicine, 2012. **367**(15): p. 1397-1406.
1061. *de Ruyter JC, Olthof MR, Kuijper LD, Katan MB. Effect of sugar-sweetened beverages on body weight in children: design and baseline characteristics of the Doubleblind, Randomized INtervention study in Kids. Contemp Clin Trials 2012;33:247-57*
1062. Ebbeling, C.B., et al., *Effects of decreasing sugar-sweetened beverage consumption on body weight in adolescents: a randomized, controlled pilot study.* Pediatrics, 2006. **117**(3): p. 673-680.
1063. *Epstein LH, Gordy CC, Raynor HA, Beddome M, Kilanowski CK, Paluch R. Increasing fruit and vegetable intake and decreasing fat and sugar intake in families at risk for childhood obesity. Obesity research. 2001 Mar;9(3):171-8.*
1064. Ebbeling, C.B., et al., *A randomized trial of sugar-sweetened beverages and adolescent body weight.* N Engl J Med, 2012. **367**: p. 1407-1416.
1065. Feng, D., et al., *A multicomponent intervention helped reduce sugar-sweetened beverage intake in economically disadvantaged hispanic children.* American Journal of Health Promotion, 2016. **30**(8): p. 594-603.
1066. Ford, A.L., et al., *Treatment of childhood obesity by retraining eating behaviour: randomised controlled trial.* Bmj, 2010. **340**.
1067. French, G.M., et al., *An evaluation of mother-centered anticipatory guidance to reduce obesogenic infant feeding behaviors.* Pediatrics, 2012. **130**(3): p. e507-e517.
1068. Gatto, N.M., et al., *LA Sprouts: a garden-based nutrition intervention pilot program influences motivation and preferences for fruits and vegetables in Latino youth.* Journal of the Academy of Nutrition and Dietetics, 2012. **112**(6): p. 913-920.
1069. Davis, J.N., et al., *LA Sprouts: A Gardening, Nutrition, and Cooking Intervention for Latino Youth Improves Diet and Reduces Obesity.* Journal of the American Dietetic Association, 2011. **111**(8): p. 1224-1230.
1070. Gatto, N.M., et al., *LA sprouts randomized controlled nutrition and gardening program reduces obesity and metabolic risk in latino youth.* Obesity, 2015. **23**(6): p. 1244-1251.
1071. Gatto, N.M., et al., *LA sprouts randomized controlled nutrition, cooking and gardening programme reduces obesity and metabolic risk in Hispanic/Latino youth.* Pediatric obesity, 2017. **12**(1): p. 28-37.
1072. Martinez, L.C., et al., *Design and methodology of the LA Sprouts nutrition, cooking and gardening program for Latino youth: a randomized controlled intervention.* Contemporary clinical trials, 2015. **42**: p. 219-227.

1073. Gross, R.S., et al., *Randomized controlled trial of an early child obesity prevention intervention: Impacts on infant tummy time.* Obesity, 2017. **25**(5): p. 920-927.
1074. Gross, R.S., et al., *Randomized Controlled Trial of a Primary Care-Based Child Obesity Prevention Intervention on Infant Feeding Practices.* Journal of Pediatrics, 2016. **174**: p. 171-177.e2.
1075. Haerens, L., et al., *A computer-tailored dietary fat intake intervention for adolescents: results of a randomized controlled trial.* Annals of Behavioral Medicine, 2007. **34**(3): p. 253-262.
1076. Haerens, L., et al., *Evaluation of a 2-year physical activity and healthy eating intervention in middle school children.* Health Education Research, 2006. **21**(6): p. 911-921.
1077. Helle, C., et al., *Evaluation of an eHealth intervention aiming to promote healthy food habits from infancy-the Norwegian randomized controlled trial Early Food for Future Health.* International Journal of Behavioral Nutrition and Physical Activity, 2019. **16**(1): p. 1.
1078. Helle, C., et al., *Early food for future health: a randomized controlled trial evaluating the effect of an eHealth intervention aiming to promote healthy food habits from early childhood.* BMC Public Health. **17**(1): p. 729.
1079. Hoffman, J.A., et al., *Decaying behavioral effects in a randomized, multi-year fruit and vegetable intake intervention.* Preventive medicine, 2011. **52**(5): p. 370-375.
1080. Horton, L.A., et al., *Targeting children's dietary behaviors in a family intervention:'Entre familia: reflejos de salud'.* salud pública de méxico, 2013. **55**: p. 397-405.
1081. Inostroza, J., et al., *Low-protein formula slows weight gain in infants of overweight mothers.* Journal of pediatric gastroenterology and nutrition, 2014. **59**(1): p. 70.
1082. Jamelske, E., et al., *Preliminary findings from an evaluation of the USDA Fresh Fruit and Vegetable Program in Wisconsin schools.* Wisconsin Medical Journal (WMJ), 2008. **107**(5): p. 225.
1083. Kelishadi, R., et al., *Can a dairy-rich diet be effective in long-term weight control of young children?* Journal of the American College of Nutrition, 2009. **28**(5): p. 601-610.
1084. Kirk, S., et al., *Role of carbohydrate modification in weight management among obese children: a randomized clinical trial.* The Journal of pediatrics, 2012. **161**(2): p. 320-327. e1.
1085. Kong, A.P., et al., *A randomized controlled trial to investigate the impact of a low glycemic index (GI) diet on body mass index in obese adolescents.* BMC public health, 2014. **14**(1): p. 180.
1086. Lappe, J.M., et al., *The effect of increasing dairy calcium intake of adolescent girls on changes in body fat and weight.* The American journal of clinical nutrition, 2017. **105**(5): p. 1046-1053.
1087. Lappe, J.M., et al., *Girls on a high-calcium diet gain weight at the same rate as girls on a normal diet: a pilot study.* Journal of the American Dietetic Association, 2004. **104**(9): p. 1361-1367.
1088. Lent, M.R., et al., *A randomized controlled study of a healthy corner store initiative on the purchases of urban, low-income youth.* Obesity, 2014. **22**(12): p. 2494-2500.
1089. Machuca, H., et al., *Well baby group care: Evaluation of a promising intervention for primary obesity prevention in toddlers.* Childhood Obesity, 2016. **12**(3): p. 171-178.
1090. Maes, L., et al., *Pilot evaluation of the HELENA (Healthy Lifestyle in Europe by Nutrition in Adolescence) Food-O-Meter, a computer-tailored nutrition advice for adolescents: a study in six European cities.* Public health nutrition, 2011. **14**(7): p. 1292-1302.
1091. Mendes, M., et al., *Effects of two diet techniques and delivery mode on weight loss, metabolic profile and food intake of obese adolescents: a fixed diet plan and a calorie-counting diet.* European journal of clinical nutrition, 2016. **(no pagination)**.
1092. Mihas, C., et al., *Evaluation of a nutrition intervention in adolescents of an urban area in Greece: short-and long-term effects of the VYRONAS study.* Public health nutrition, 2010. **13**(5): p. 712-719.
1093. Muckelbauer, R., et al., *Promotion and provision of drinking water in schools for overweight prevention: randomized, controlled cluster trial.* Pediatrics, 2009. **123**(4): p. e661-e667.
1094. *Muckelbauer R, Libuda L, Clausen K, Reinehr T, Kersting M. A simple dietary intervention in the school setting decreased incidence of overweight in children. Obesity facts. 2009;2(5):282-5.*

1095. Muckelbauer, R., et al., *Changes in water and sugar-containing beverage consumption and body weight outcomes in children.* British Journal of Nutrition. **115**(11): p. 2057-66.
1096. Muckelbauer, R., et al., *Immigrational Background Affects the Effectiveness of a School-based Overweight Prevention Program Promoting Water Consumption.* Obesity (19307381), 2010. **18**(3): p. 528-534.
1097. Papadaki, A., et al., *The effect of protein and glycemic index on children's body composition: the DiOGenes randomized study.* Pediatrics, 2010. **126**(5): p. e1143-e1152.
1098. Larsen, T.M., et al., *The Diet, Obesity and Genes (Diogenes) Dietary Study in eight European countries - a comprehensive design for long-term intervention.* Obesity Reviews, 2010. **11**(1): p. 76-91.
1099. Paul, I.M., et al., *Preventing obesity during infancy: a pilot study.* Obesity, 2011. **19**(2): p. 353-361.
1100. Pinket, A.-S., et al., *Effect and process evaluation of a cluster randomized control trial on water intake and beverage consumption in preschoolers from six European countries: the ToyBox-study.* PloS one, 2016. **11**(4).
1101. Birnbaum, J., et al., *Effects of a kindergarten-based, family-involved intervention on motor performance ability in 3- to 6-year-old children: the ToyBox-study.* Journal of sports sciences, 2017. **35**(4): p. 377-384.
1102. Cheah, W.L., et al., *Implementation of an obesity prevention intervention to pre-school children in Borneo, Malaysia: The ToyBox Malaysia study.* Obesity Facts, 2019. **12**: p. 29-30.
1103. De Craemer, M., et al., *Effect and process evaluation of a kindergarten-based, family-involved cluster randomised controlled trial in six European countries on four- to six-year-old children's steps per day: the ToyBox-study.* International Journal of Behavioral Nutrition & Physical Activity, 2017. **14**: p. 1-16.
1104. De Miguel-Etayo, P., et al., *Recruitment rate in a kindergarten-based, family-involved intervention to prevent childhood obesity across europe by socio-economic status: The toybox study.* Annals of Nutrition and Metabolism, 2018. **73**: p. 25-26.
1105. Duvinage, K., et al., *Developing the intervention material to increase physical activity levels of European preschool children: the ToyBox-study.* Obesity Reviews. **15 Suppl 3**: p. 27-39.
1106. Gonzalez-Gil, E.M., et al., *Reliability of primary caregivers reports on lifestyle behaviours of European pre-school children: the ToyBox-study.* Obesity Reviews. **15 Suppl 3**: p. 61-6.
1107. Latomme, J., et al., *Effect and process evaluation of a kindergarten-based, family-involved intervention with a randomized cluster design on sedentary behaviour in 4- to 6-year old European preschool children: The ToyBoxstudy.* PLoS ONE, 2017. **12**(4).
1108. Malden, S., et al., *Translating the ToyBox obesity prevention intervention to the Scottish preschool setting: Adaptation processes and implementation challenges.* Obesity Facts, 2019. **12**: p. 30.
1109. Malden, S., et al., *Feasibility of the ToyBox-Scotland obesity prevention intervention in preschools: results of a cluster randomised controlled trial.* Obesity facts, 2019. **12**: p. 213-.
1110. Malden, S., et al., *Adaptation of the ToyBox pre-school obesity prevention programme for use in Scotland: Intervention adaptation processes and baseline results of a feasibility cluster randomised controlled trial.* Obesity Facts, 2018. **11**: p. 213.
1111. Malden, S., et al., *A feasibility cluster randomised controlled trial of a preschool obesity prevention intervention: ToyBox-Scotland.* Pilot and Feasibility Studies, 2019. **5**(1).
1112. Manios, Y., *The 'ToyBox-study' obesity prevention programme in early childhood: an introduction.* Obesity Reviews. **13 Suppl 1**: p. 1-2.
1113. Manios, Y., et al., *Designing and implementing a kindergarten-based, family-involved intervention to prevent obesity in early childhood: The ToyBox-study.* Obesity Reviews, 2014. **15**(SUPPL.3): p. 5-13.

1114. Miguel-Berges, M.L., et al., *Associations between food and beverage consumption and different types of sedentary behaviours in European preschoolers: the ToyBox-study.* European journal of nutrition, 2017. **56**(5): p. 1939-1951.
1115. Miguel-Berges, M.L., et al., *Combined longitudinal effect of physical activity and screen time on food and beverage consumption in European preschool children: The toybox-study.* Nutrients, 2019. **11**(5).
1116. Miguel-Berges, M.L., et al., *Clustering of energy balance-related behaviours and parental education in European preschool children: the ToyBox study.* British journal of nutrition, 2017. **118**(12): p. 1089-1096.
1117. Pinket, A.S., et al., *Diet quality in European pre-schoolers: evaluation based on diet quality indices and association with gender, socio-economic status and overweight, the ToyBox-study.* Public Health Nutrition. **19**(13): p. 2441-50.
1118. Pinket, A.S., et al., *Multibehavioural Interventions with a Focus on Specific Energy Balance-Related Behaviours Can Affect Diet Quality in Preschoolers from Six European Countries: The ToyBox-Study.* Nutrients, 2017. **9**(5): p. 10.
1119. Poh, B.K., et al., *Implementation and challenges of an obesity prevention intervention for preschool children in Kuala Lumpur metropolitan: The ToyBox study Malaysia.* Obesity facts, 2019. **12**: p. 29.
1120. Reeves, S., et al., *ToyBox Study Malaysia: Improving healthy energy balance and obesity-related behaviours among pre-schoolers in Malaysia.* Nutrition Bulletin, 2018. **43**(3): p. 290-295.
1121. Rappaport, E., C. Daskalakis, and J. Sendecki, *Using routinely collected growth data to assess a school-based obesity prevention strategy.* International journal of obesity, 2013. **37**(1): p. 79-85.
1122. Rausch Herscovici, C., I. Kovalskys, and M.J. De Gregorio, *Gender differences and a school-based obesity prevention program in Argentina: a randomized trial.* Revista Panamericana de Salud Pública, 2013. **34**: p. 75-82.
1123. Rosado, J.L., et al., *An increase of cereal intake as an approach to weight reduction in children is effective only when accompanied by nutrition education: a randomized controlled trial.* Nutrition journal, 2008. **7**(1): p. 28.
1124. Rosário, R., et al., *Impact of an intervention through teachers to prevent consumption of low nutrition, energy-dense foods and beverages: A randomized trial.* Preventive Medicine, 2013. **57**(1): p. 20-25.
1125. Rosário, R., et al., *The impact of an intervention taught by trained teachers on childhood fruit and vegetable intake: A randomized trial.* Journal of Obesity, 2012. **2012**.
1126. Rosário, R., et al., *The impact of an intervention taught by trained teachers on childhood overweight.* International Journal of Environmental Research and Public Health, 2012. **9**(4): p. 1355-1367.
1127. Salazar Vázquez, B., et al., *Control of overweight and obesity in childhood through education in meal time habits. the 'good manners for a healthy future'programme.* Pediatric obesity, 2016. **11**(6): p. 484-490.
1128. Schroeder, N., et al., *Early obesity prevention: A randomized trial of a practice-based intervention in 0–24-month infants.* Journal of obesity, 2015. **2015**.
1129. Schwartz, A.E., et al., *Effect of a school-based water intervention on child body mass index and obesity.* JAMA pediatrics, 2016. **170**(3): p. 220-226.
1130. Sichieri, R., et al., *School randomised trial on prevention of excessive weight gain by discouraging students from drinking sodas.* Public health nutrition, 2009. **12**(2): p. 197-202.
1131. Sichieri, R., et al., *Water and sugar-sweetened beverage consumption and changes in BMI among Brazilian fourth graders after 1-year follow-up.* Public Health Nutrition, 2013. **16**(1): p. 73-77.

1132. Struempler, B.J., et al., *Changes in fruit and vegetable consumption of third-grade students in body quest: food of the warrior, a 17-class childhood obesity prevention program.* Journal of nutrition education and behavior, 2014. **46**(4): p. 286-292.
1133. Truby, H., et al., *A randomized controlled trial of two different macronutrient profiles on weight, body composition and metabolic parameters in obese adolescents seeking weight loss.* PloS one, 2016. **11**(3).
1134. Truby, H., et al., *Adolescents seeking weight management: who is putting their hand up and what are they looking for?* Journal of Paediatrics & Child Health, 2011. **47**(1-2): p. 2-4.
1135. Truby, H., et al., *The Eat Smart Study: a randomised controlled trial of a reduced carbohydrate versus a low fat diet for weight loss in obese adolescents.* BMC Public Health, 2010. **10**(1): p. 464-464.
1136. Van Horn, L., et al., *Children's adaptations to a fat-reduced diet: the Dietary Intervention Study in Children (DISC).* Pediatrics, 2005. **115**(6): p. 1723-1733.
1137. Albertson, A.M., et al., *The relationship of ready-to-eat cereal consumption to nutrient intake, blood lipids, and body mass index of children as they age through adolescence.* Journal of the American Dietetic Association, 2009. **109**(9): p. 1557-1565.
1138. Williams, C.L., B.A. Strobino, and J. Brotanek, *Weight control among obese adolescents: a pilot study.* International journal of food sciences and nutrition, 2007. **58**(3): p. 217-230.
1139. Wong, J.M., et al., *Effects of advice to drink 8 cups of water per day in adolescents with overweight or obesity: a randomized clinical trial.* JAMA pediatrics, 2017. **171**(5): p. e170012-e170012.
1140. Beaulieu, D. and G. Godin, *Staying in school for lunch instead of eating in fast-food restaurants: results of a quasi-experimental study among high-school students.* Public Health Nutrition. **15**(12): p. 2310-9.
1141. Daniels, L., et al., *Evaluation of an intervention to promote protective infant feeding practices to prevent childhood obesity: outcomes of the NOURISH RCT at 14 months of age and 6 months post the first of two intervention modules.* International journal of obesity, 2012. **36**(10): p. 1292-1298.
1142. Daniels, L.A., et al., *The NOURISH randomised control trial: Positive feeding practices and food preferences in early childhood - A primary prevention program for childhood obesity.* BMC Public Health, 2009. **9**.
1143. Daniels, L.A., et al., *Child eating behavior outcomes of an early feeding intervention to reduce risk indicators for child obesity: The NOURISH RCT.* Obesity (19307381), 2014. **22**(5): p. E104-11.
1144. Daniels, L.A., et al., *Outcomes of an early feeding practices intervention to prevent childhood obesity.* Pediatrics, 2013. **132**(1): p. e109-e118.
1145. Ermetici, F., et al., *Association between a school-based intervention and adiposity outcomes in adolescents: The Italian "EAT" project.* Obesity (19307381), 2016. **24**(3): p. 687-695.
1146. Malavazos, A.E., et al., *The italian eat. project: Effectiveness of a multicomponent school-based health promotion study on measures of fatness and behavior in teenagers.* Acta Paediatrica, International Journal of Paediatrics, 2017. **106**: p. 20.
1147. Golan, M. and S. Crow, *Targeting parents exclusively in the treatment of childhood obesity: long-term results.* Obesity Research, 2004. **12**(2): p. 357-61.
1148. Golan, M., M. Fainaru, and A. Weizman, *Role of behaviour modification in the treatment of childhood obesity with the parents as the exclusive agents of change.* International Journal of Obesity & Related Metabolic Disorders: Journal of the International Association for the Study of Obesity. **22**(12): p. 1217-24.
1149. Golan, M., A. Weizman, and M. Fainaru, *Impact of treatment for childhood obesity on parental risk factors for cardiovascular disease.* Preventive Medicine, 1999. **29**(6 I): p. 519-526.
1150. Golan, M., V. Kaufman, and D.R. Shahar, *Childhood obesity treatment: targeting parents exclusively v. parents and children.* British Journal of Nutrition. **95**(5): p. 1008-15.

1151. Kitzman-Ulrich, H., et al., *An adolescent weight-loss program integrating family variables reduces energy intake.* Journal of the American Dietetic Association, 2009. **109**(3): p. 491-496.
1152. Koo, H.C., B.K. Poh, and R. Abd Talib, *The GReat-Child Trial: A Quasi-Experimental Intervention on Whole Grains with Healthy Balanced Diet to Manage Childhood Obesity in Kuala Lumpur, Malaysia.* Nutrients, 2018. **10**(2).
1153. Bonham, M.P., et al., *Evaluation of 'JenMe', a commercially-delivered weight management program for adolescents: A randomised controlled trial.* FASEB journal, 2017. **31**(1).
1154. Dordevic, A.L., et al., *Study protocol: evaluation of 'JenMe', a commercially-delivered weight management program for adolescents: a randomised controlled trial.* BMC public health, 2015. **15**: p. 563.
1155. Bonham, M.P., et al., *Evaluation of a Commercially Delivered Weight Management Program for Adolescents.* Journal of Pediatrics. **185**: p. 73-80.e3.
1156. Moschonis, G., et al., *Assessment of the Effectiveness of a Computerised Decision-Support Tool for Health Professionals for the Prevention and Treatment of Childhood Obesity. Results from a Randomised Controlled Trial.* Nutrients, 2019. **11**(3).
1157. Mirza, N.M., et al., *Effects of a low glycemic load or a low-fat dietary intervention on body weight in obese Hispanic American children and adolescents: a randomized controlled trial.* American Journal of Clinical Nutrition. **97**(2): p. 276-85.
1158. Polonsky, H.M., et al., *Effect of a Breakfast in the Classroom Initiative on Obesity in Urban School-aged Children: A Cluster Randomized Clinical Trial.* JAMA Pediatrics, 2019. **173**(4): p. 326-333.
1159. Sharma, S.V., et al., *Evaluating a school-based fruit and vegetable co-op in low-income children: A quasi-experimental study.* Preventive Medicine, 2016. **91**: p. 8-17.
1160. Siegel, R.M., M.S. Neidhard, and S. Kirk, *A Comparison of Low Glycemic Index and Staged Portion-Controlled Diets in Improving BMI of Obese Children in a Pediatric Weight Management Program.* Clinical Pediatrics, 2011. **50**(5): p. 459-461.
